# Sotorasib-impaired degradation of NEU1 contributes to cardiac injury by inhibiting AKT signaling

**DOI:** 10.1038/s41420-025-02431-x

**Published:** 2025-04-12

**Authors:** Mengting Cheng, Wentong Wu, Qing Li, Xinyu Tao, Feng Jiang, Jinjin Li, Nonger Shen, Fei Wang, Peihua Luo, Qiaojun He, Ping huang, Zhifei Xu, Yiwen Zhang

**Affiliations:** 1 Clinical Pharmacy Center, Department of Pharmacy, Zhejiang Provincial People’s Hospital, Affiliated People’s Hospital, Hangzhou Medical College, Hangzhou, China; 2 Center for Drug Safety Evaluation and Research of Zhejiang University, College of Pharmaceutical Sciences, Zhejiang University, Hangzhou, China; 3 School of Pharmaceutical Sciences, Zhejiang Chinese Medical University, Hangzhou, China; 4 Outpatient Pharmacy, Department of Pharmacy, Zhejiang Provincial People’s Hospital, Affiliated People’s Hospital, Hangzhou Medical College, Hangzhou, China; 5 Department of Pharmacology and Toxicology, Hangzhou Institute of Innovative Medicine, College of Pharmaceutical Sciences, Zhejiang University, Hangzhou, China; 6 Key Laboratory of Clinical Cancer Pharmacology and Toxicology Research of Zhejiang Province, Affiliated Hangzhou Cancer Hospital, Zhejiang University School of Medicine, Hangzhou, China; 7 Innovation Institute for Artificial Intelligence in Medicine of Zhejiang University, Hangzhou, China; 8 Zhejiang Provincial Clinical Research Center for Malignant Tumor, Hangzhou, People’s Republic of China; 9 Key Laboratory of Endocrine Gland Diseases of Zhejiang Province, Hangzhou, China

**Keywords:** Ubiquitylation, Non-small-cell lung cancer

## Abstract

Sotorasib, the inaugural targeted inhibitor sanctioned for the management of patients afflicted with locally advanced or metastatic non-small cell lung cancer presenting the KRAS G12C mutation, has encountered clinical application constraints due to its potential for cardiac injury as evidenced by safety trials. This investigation has elucidated that the heightened expression of neuraminidase-1 (NEU1) constitutes the principal etiology of cardiac damage induced by sotorasib. Mechanistically, sotorasib treatment inhibited the ubiquitinated degradation of NEU1, leading to its elevated expression, which induced downstream AKT signaling pathway inhibition and mitochondrial dysfunction leading to cardiomyocyte apoptosis. Meanwhile, in vivo and in vitro studies showed that D-pantothenic acid (D-PAC) alleviated sotorasib-induced cardiac damage by promoting NEU1 degradation. In conclusion, this study revealed that NEU1 is a key protein in sotorasib cardiotoxicity and that reducing the level of this protein is a critical strategy for the clinical treatment of sotorasib-induced cardiac injury.

**Figure:**
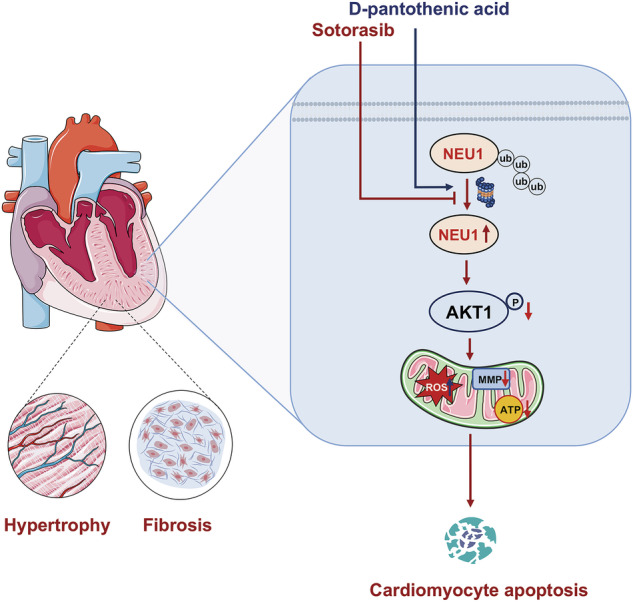
Schematic representation of a mechanism.

Schematic representation of a mechanism.

## Introduction

Sotorasib, a RAS GTPase family inhibitor for the treatment of solid tumors with KRAS G12C mutations, received approval from the Food and Drug Administration (FDA) in 2021 for treating adult patients with locally advanced or metastatic non-small cell lung cancer (NSCLC) carrying such mutations and who have undergone one or more prior systemic therapies [1]. Phase 2 clinical trials demonstrated that sotorasib treatment resulted in progression-free survival (PFS) of 6.3 months in NSCLC and 4.0 months in colorectal cancer (CRC), with disease control rates of 88.1% and 73.8%, respectively [2, 3]. Safety trials revealed the incidence of adverse events during sotorasib treatment, with 56.6% experiencing such events, of which 52.7% were grade 3 or higher. Notably, one instance of cardiac arrest was reported [4], suggesting potential cardiotoxicity associated with sotorasib use.

Cardiomyocytes, characterized by their high mitochondrial content, heavily rely on mitochondrial oxidative phosphorylation to generate adenosine triphosphate (ATP) necessary for maintaining cellular function [5]. Mitochondrial dysfunction disrupts cellular homeostasis, leading to structural abnormalities, impaired membrane potential, energy deficits, and oxidative stress, ultimately culminating in cellular dysfunction and apoptosis [6, 7]. Mitochondrial dysfunction stands as a primary contributor to cardiomyocyte apoptosis and consequent cardiac injury [8, 9]. The decrease of mitochondrial transmembrane potential (MMP) occurs in the early stage of apoptosis, and this process is irreversible [10]. Drug-induced mitochondrial dysfunction represents a significant mechanism underlying drug-mediated cardiotoxicity, which occurred via three principal pathways: direct inhibition of mitochondrial replication, interaction with the electron transport chain resulting in uncoupling of electron transport and ATP production, and stimulation of reactive oxygen species (ROS) and reactive nitrogen species (RNS) production, leading to oxidative/nitrative modification of mitochondrial proteins [5, 11, 12]. It has been reported in the literature that ATP-deficient mitochondrial dysfunction ultimately causes drug-induced cardiotoxicity through a train of downstream signaling pathways [13]. Oxidative damage causes mitochondrial and nuclear DNA mutations to damage the oxygen phosphorylation process, leading to further production of mitochondrial -ROS, thus forming a “vicious circle” of mitochondrial, ROS and genome instability [14–16].

Sialidases (SA), also referred to as neuraminidases (NA), are ubiquitous enzymes found across various organisms, with mammals expressing four distinct sialidase isoforms: NEU1, NEU2, NEU3, and NEU4. Among these, has been highlighted as a pivotal regulator in cellular processes, influencing signaling pathways and disease progression [17, 18]. NEU1 is expressed in lysosomes and regulates the extracellular secretion of lysosomes by modulating the shearing of various substrates [19, 20]. Recent studies have shown that NEU1 could enter the cell nucleus under stress stimulation and regulate the cardiac remodeling genes *Nppa* and *Nppb* in combination with the transcription factor GATA4 [21]. In addition, it has been demonstrated that NEU1 affects the function of mitochondria and regulates the transmission of the mitochondrial electron respiratory chain and the production of ROS, mainly through the SIRT1/PGC-1α axis [22]. NEU1 deficiency attenuates cardiac dysfunction, oxidative stress, fibrosis, and inflammation through the AMPK-SIRT3 signaling pathway in diabetic cardiomyopathy mice [23]. Furthermore, it has been demonstrated that NEU1 blocks the downstream PI3K/AKT signaling pathway, which is intimately linked to mitochondrial function [24, 25]. This evidence suggests that NEU1 is an essential target for the treatment of cardiovascular disease.

D-PAC, also known as vitamin B5, serves as a crucial precursor in coenzyme A synthesis, participating in various metabolic pathways within the human body [26, 27]. Dysregulation of coenzyme A-dependent metabolic pathways leads to chronic diseases such as inflammatory diseases, obesity, diabetes, cancer, and cardiovascular diseases [27]. Recent research has highlighted the critical role pantothenic acid plays in maintaining mitochondrial function [28]. Dexpanthenol, a derivative of D-PAC, has demonstrated efficacy in mitigating isoprenaline-induced cardiotoxicity in animal models, although its precise mechanism remains unclear [29, 30].

Our study sheds light on the potential cardiac injury of sotorasib and identifies the vital role of NEU1 protein levels in the maintenance of cardiac homeostasis, elucidating the molecular mechanism of sotorasib-induced cardiotoxicity. For the first time, the important role of D-PAC in lowering NEU1 and the regulation of NEU1 protein levels by ubiquitination were revealed.

## Results

### Sotorasib causes cardiac injury in mice

**Fig. 1:**
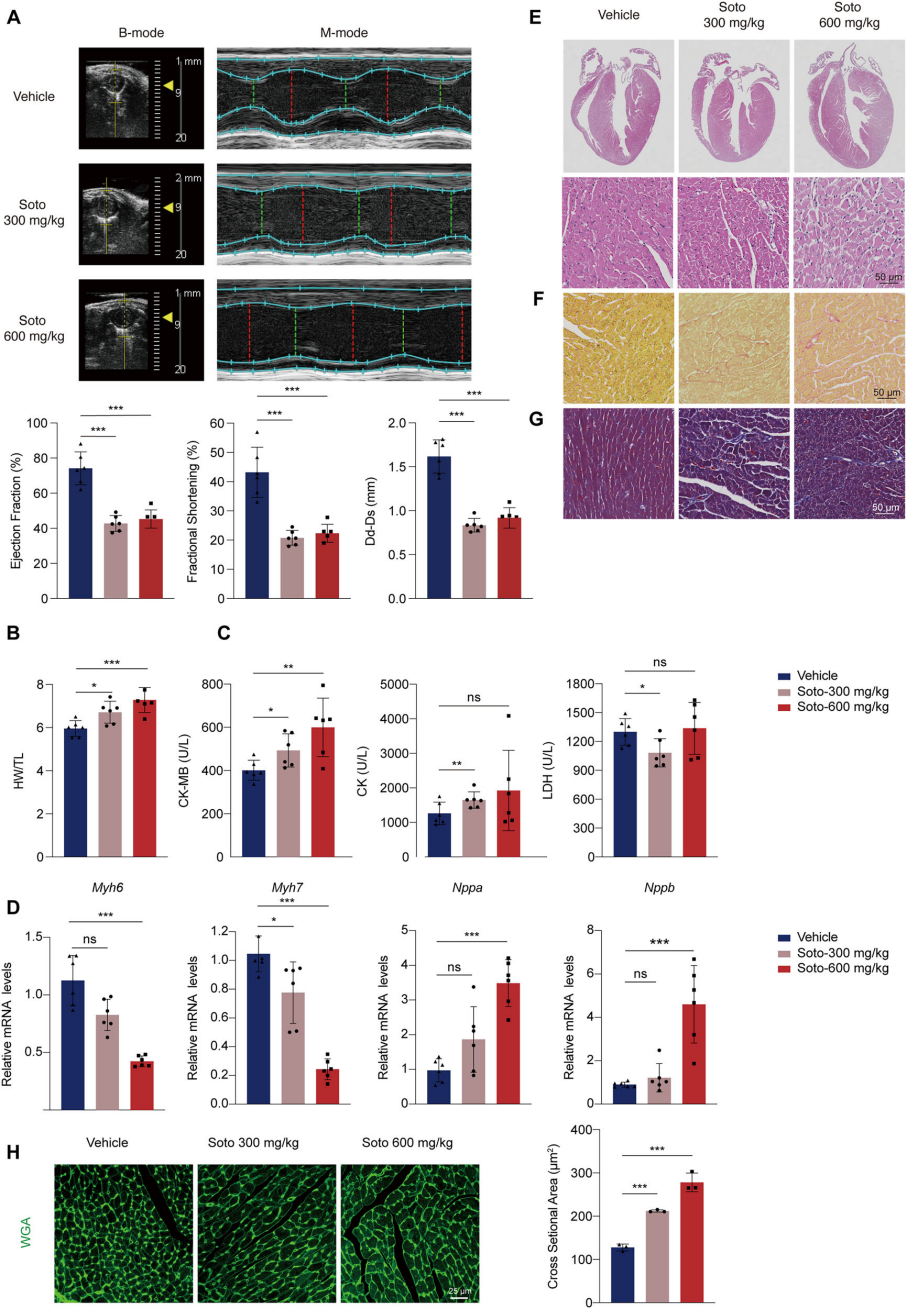
Sotorasib causes cardiac injury in mice. Male C57BL/6J mice were treated with 0.5% CMC-Na or sotorasib (300 mg/kg/d or 600 mg/kg/d) by gavage for 28 days. **A** Cardiac function was examined by echocardiography, showing the percentage of EF and FS (*n* = 6). **B** Ratio of heart weight to tibia length (*n* = 6). **C** Serum levels of CK-MB, CK and LDH were analyzed (*n* = 6). **D** mRNA levels of cardiac remodeling indicators *Myh6*, *Myh7*, *Nppa* and *Nppb* (*n* = 6). **E**–**G** Cardiac sections stained with H&E, Sirius Red and Masson (*n* = 3). Scale bars: 50 μm. **H** Cardiac sections stained with WGA (*n* = 3). Scale bars: 25 μm. Data expressed as mean ± SD, ***, *p* < 0.0001, **, *p* < 0.01, *, *p* < 0.05, ns, no significance (vs. Vehicle).

To clarify the cardiomyocyte death and toxicity of sotorasib, we established an animal model to simulate sotorasib-induced cardiotoxicity observed clinically. Male C57BL/6J mice were subjected to daily intragastric administration of 300 mg/kg/d or 600 mg/kg/d sotorasib for 28 days. Echocardiographic assessment revealed a decrease in ejection fraction (EF), fractional shortening (FS), and Dd-Ds shortening in sotorasib-treated mice (Fig. 1A), indicative of cardiac dysfunction. The ratio of heart weight (HW) to tibia length (TL) was significantly elevated in mice, suggesting that sotorasib may induce cardiac hypertrophy (Fig. 1B). Meanwhile, we found that sotorasib increased the levels of serum CK-MB in a dose-dependent manner, a crucial biochemical indicator of heart damage. A modest decrease in LDH was observed at 300 mg/kg/day of sotorasib. However, the sotorasib 600 mg/kg/d dose had no discernible effect on LDH levels. (Fig. 1C). Furthermore, there was a decrease in the mRNA level of *Myh6* (myosin, heavy peptide 6, myocardial α) and an increase in the mRNA level of *Nppa* and *Nppb* (natriuretic peptide TYPE A/B), indicating abnormal cardiac remodeling (Fig. 1D). Histologic analysis (including H&E, Masson Trichrome, and Sirius Red staining) showed that sotorasib-treated mice had slightly enlarged hearts with significant damage and collagen deposition, indicating the occurrence of cardiac fibrosis (Fig. 1E–G). Wheat germ agglutinin (WGA) staining showed noticeably increased cardiomyocyte size in the hearts of those treated with sotorasib (Fig. 1H). Collectively, our in vivo studies highlight significant cardiac injury associated with sotorasib treatment in mice, including potential myocardial dysfunction, myocardial injury, cardiac fibrosis, and cardiac hypertrophy.

### Sotorasib induces apoptosis and mitochondrial dysfunction in cardiomyocytes

**Fig. 2:**
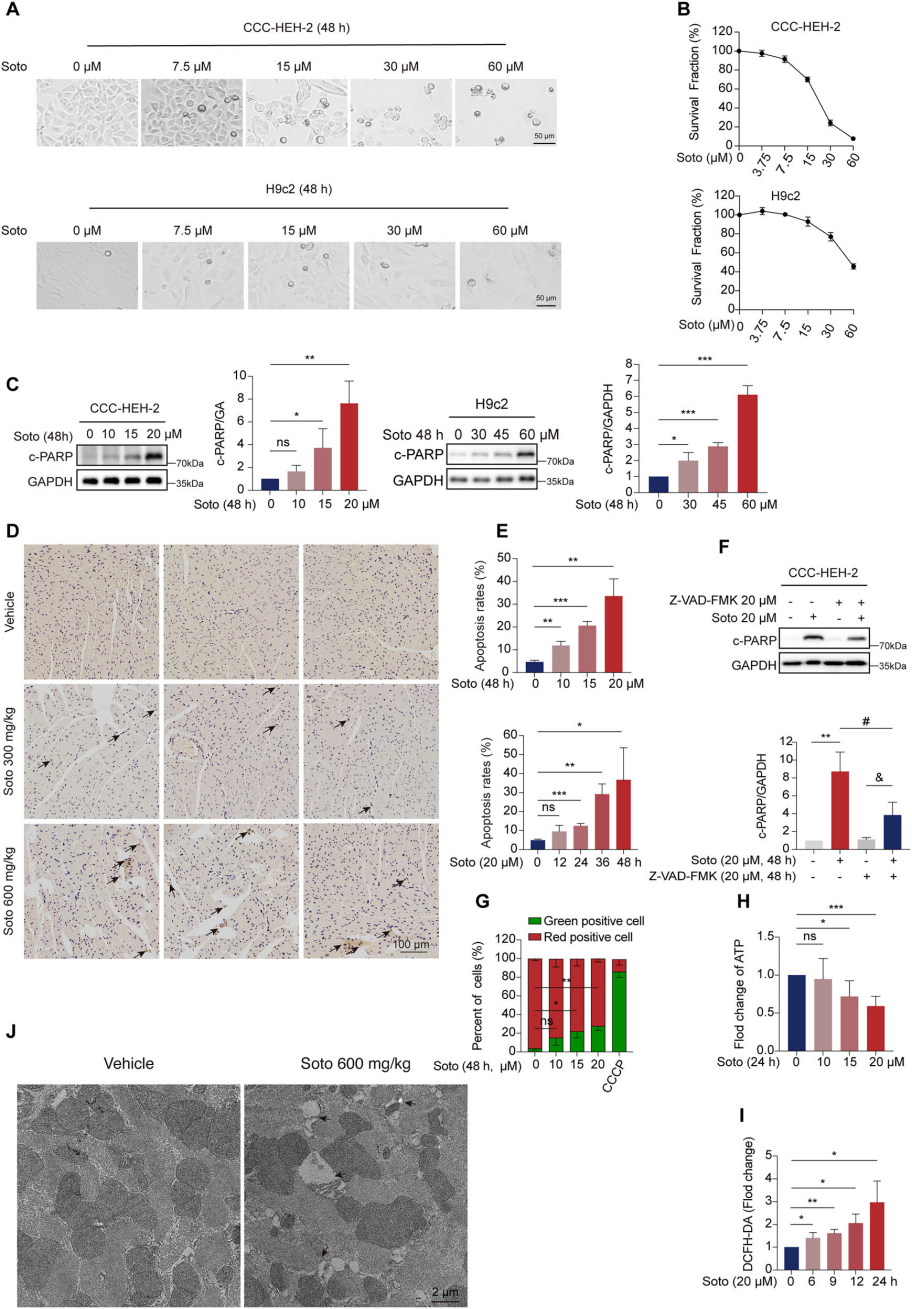
Sotorasib induces apoptosis and mitochondrial dysfunction in cardiomyocytes. **A** CC-HEH-2 cells were treated with 0, 7.5, 15, 30, 60 μM sotorasib for 48 h; H9c2 cells were treated with 0, 7.5, 15, 30, 60 μM sotorasib for 48 h (*n* = 3). Representative pictures. Scale bars: 50 μm. **B** CCC-HEH-2 cells were treated with 0, 3.75, 7.5, 15, 30, 60 μM sotorasib for 48 h; H9c2 cells were treated with 0, 3.75, 7.5, 15, 30, 60 μM sotorasib for 48 h. Survival fraction detection by SRB colorimetric assay (*n* = 3). **C** CCC-HEH-2 cells were exposed to different concentrations of 0, 10, 15, or 20 μM sotorasib for 48 h. H9c2 cells were exposed to different concentrations of 0, 30, 45, or 60 μM sotorasib for 48 h. Expression levels of c-PARP protein in CCC-HEH-2 and H9c2 cells. **D** Male C57BL/6J mice were treated with 0.5% CMC-Na or sotorasib (300 mg/kg/d or 600 mg/kg/d) by gavage for 28 days (*n* = 6). Representative image of IHC staining of cardiomyocytes c-CAPSP3 (*n* = 3). Scale bars: 100 μm. **E** CCC-HEH-2 were treated with 20 μM sotorasib for 0, 12, 24, 36, and 48 h and exposed to different concentrations of 0, 10, 15, or 20 μM sotorasib for 48 h. The apoptosis rate of CCC-HEH-2 cells (*n* = 3). **F** 20 μM sotorasib and/or 20 μM Z-VAD-FMK treated CCC-HEH-2 for 48 h. Expression level of c-PARP in CCC-HEH-2 cells (*n* = 3). **G** CCC-HEH-2 were exposed to different concentrations of 0, 10, 15, or 20 μM sotorasib for 48 h. MMP changes in CCC-HEH-2 cells (*n* = 3). **H** CCC-HEH-2 and were exposed to different concentrations of 0, 10, 15, or 20 μM sotorasib for 24 h. ATP levels in CCC-HEH-2 cells (*n* = 3). **I** 20 μM sotorasib treatment of CCC-HEH-2 cells for 0, 6, 9, 12 and 24 h. ROS levels in CCC-HEH-2 cells (*n* = 3). **J** Male C57BL/6J mice were treated with 0.5% CMC-Na or sotorasib (300 mg/kg/d or 600 mg/kg/d) by gavage for 28 days (*n* = 6). Transmission electron microscopy representative images. scale bars: 2 μm. Data expressed as mean ± SD, ***, *p* < 0.0001, **, *p* < 0.01, *, *p* < 0.05 (vs. Vehicle), #, *p* < 0.05 (vs. sotorasib), &, *p* < 0.05 (vs. Z-VAD-FMK), ns, no significance.

To further investigate the effects of sotorasib on cardiomyocytes, we utilized the cell line CCC-HEH-2, derived from human embryonic heart tissue, and observed a significant decrease in cell viability after sotorasib treatment, which was consistent with the findings in H9c2 rat cardiomyocytes (Fig. 2A, B). Western blot examination revealed that c-PARP (cleaved poly (ADP-ribose) polymerase family), markers of apoptosis, expression was dramatically enhanced in the CCHEH-2 and H9c2 cell lines and was in a dose- and time-dependent manner (Figs. 2C and S1A). This observation was further supported by immunofluorescence (IF) staining for cleaved- Caspase-3 (cleaved-CASP3) and Annexin V-PI staining using flow cytometry (Figs. S1B and 2E). Cleaved-CASP3 expression was further demonstrated to be upregulated by immunohistochemistry (IHC) (Fig. 2D). Treatment with the pan-Caspase inhibitor Z-VAD-FMK effectively rescued sotorasib-induced apoptosis in CCC-HEH-2 cells (Figs. 2F and S1C, D). These findings imply that sotorasib-mediated apoptosis in cardiomyocytes may result in heart failure.

To further investigate the effects of sotorasib on mitochondrial function in cardiomyocytes, we detected the MMP by JC-1 staining followed by flow cytometry, the findings demonstrated that sotorasib decreased MMP in a dose- and time-dependent manner, suggesting that sotorasib might damage the electron transport chain and thus caused mitochondrial dysfunction (Figs. 2G and S1E). Next, we evaluated ATP production and noted a dose-dependent decline in ATP levels upon sotorasib treatment (Fig. 2H). Employing flow cytometry, we quantified ROS production and detected a significant increase following sotorasib administration (Fig. 2I). Excessive accumulation of ROS caused mitochondrial damage eventually activating mitochondrial autophagy [15]. Transmission electron microscopy (TEM) results showed that mitochondrial autophagy increased after sotorasib treatment (Fig. 2J). In conclusion, our findings indicate that sotorasib is cardiotoxic, given that it causes apoptosis and mitochondrial dysfunction in cardiomyocytes.

### Sotorasib-mediated upregulation of NEU1 contributes to mitochondria-dependent cardiac injury

**Fig. 3:**
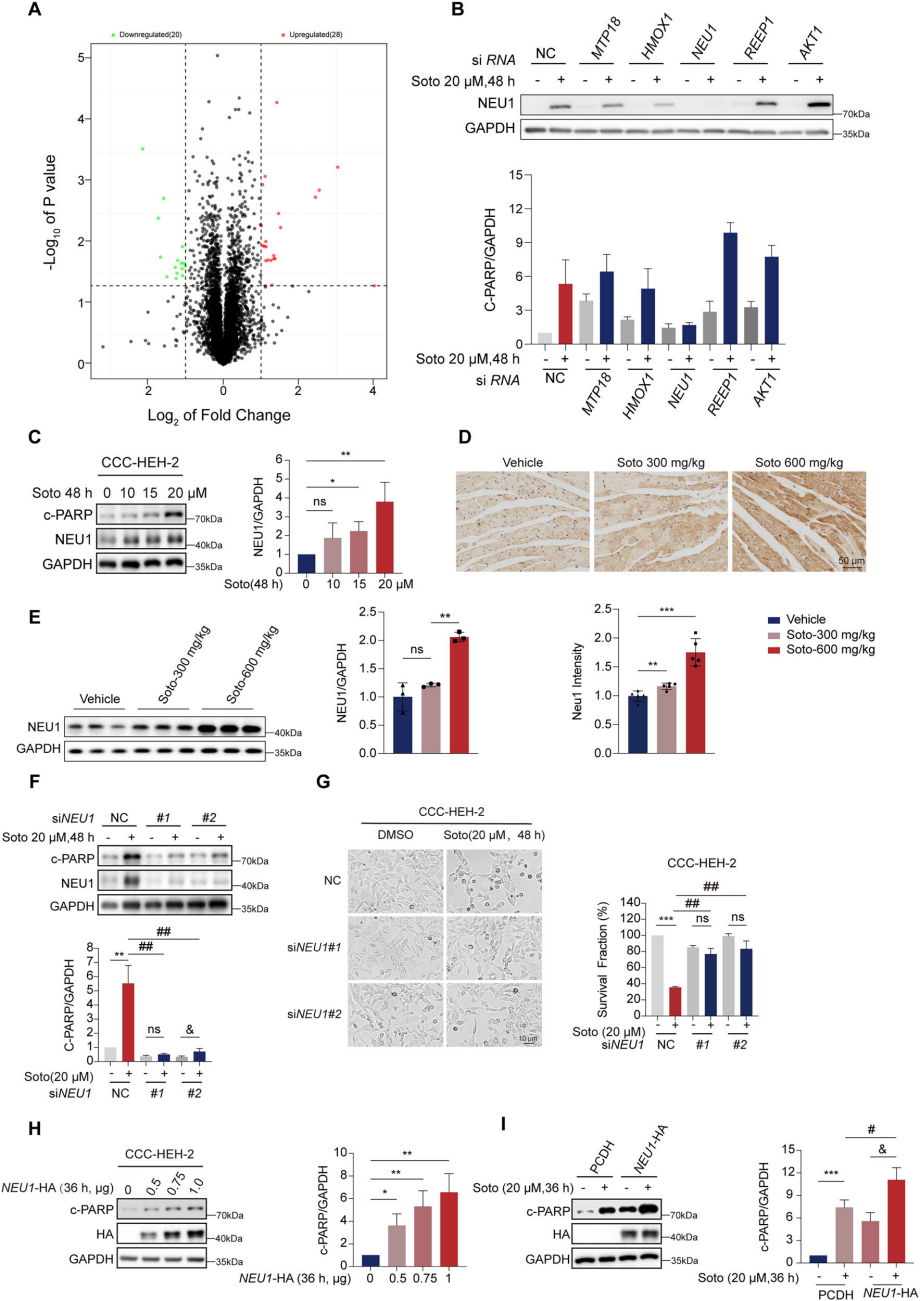
Sotorasib-mediated upregulation of NEU1 contributes to cardiomyocyte apoptosis. **A** Proteomic analysis of CCC-HEH-2 cell samples from control and sotorasib groups (*n* = 3). Volcano Plot. **B** CCC-HEH-2 cells transfected with NC or *MTP18*, *HMOX1*, *NEU1*, *REEP1*, and *AKT1* siRNA were treated with sotorasib for 48 h. Expression levels of c-PARP protein in CCC-HEH-2 cells (*n* = 3). **C** CCC-HEH-2 were exposed to different concentrations of 0, 10, 15, or 20 μM sotorasib for 48 h. c-PARP and NEU1 expression levels in CCC-HEH-2 cells (*n* = 3). Male C57BL/6J mice were treated with 0.5% CMC-Na or sotorasib (300 mg/kg/d or 600 mg/kg/d) by gavage for 28 days. **D** Representative image of NEU1 IHC in heart tissue (*n* = 3). Scale bar: 50 μm. **E** NEU1 protein expression levels in mouse tissues (*n* = 3). CCC-HEH-2 cells transfected with NC, *NEU1*#1 or *NEU1*#2 knockdown were treated with sotorasib for 48 h. **F** c-PARP and NEU1 expression levels in CCC-HEH-2 cells (*n* = 3). **G** Survival fraction and representative pictures of CCC-HEH-2 cells (*n* = 3). Scale bar: 10 μm. **H** CCC-HEH-2 cells were transfected with NEU1-HA plasmid overexpressing NEU1 at concentrations of 0, 0.5, 0.75 and 1.0 μg, respectively, for 48 h. HA and c-PARP expression levels in CCC-HEH-2 cells (*n* = 3). **I** CCC-HEH-2 cells were transfected with 0.5 μg PCDH or *NEU1*-HA plasmid after sotorasib treatment for 36 h. HA and c-PARP expression levels in CCC-HEH-2 cells (*n* = 3). Data expressed as mean ± SD, ***, *p* < 0.0001, **, *p* < 0.01, *, *p* < 0.05 (vs. Vehicle), #, *p* < 0.05 (vs. NC+sotorasib), &, *p* < 0.05 (vs. siNEU1#2 or NEU1-HA), ns, no significance.

To delve deeper into the mechanism underlying sotorasib-induced cardiomyocyte apoptosis, we subjected CCC-HEH-2 cells to sotorasib treatment for mass spectrometry (MS) analysis. We found that 28 proteins were significantly upregulated compared to the vehicle-treated cardiomyocytes, while 21 were downregulated (Figs. 3A, S2A and Table S1). Among these proteins, we focused on five proteins associated with cardiac dysfunction for further validation. Upon treating CCC-HEH-2 cells with scramble siRNA or specific siRNAs, western blot analysis revealed a marked reduction in c-PARP levels only upon NEU1 knockdown (Fig. 3B), suggesting a potential role of NEU1 in mediating sotorasib-induced cardiac damage. Western blot and IF analysis of CCC-HEH-2 cells indicated that NEU1 protein levels were elevated in a time- and dose-dependent manner, and that the elevation of this protein preceded c-PARP (Figs. 3C and S2B, C). Subsequently, to confirm this hypothesis, we used western blot and IHC to analyze the expression of NEU1 protein in sotorasib-treated mice, which showed that NEU1 expression increased in sotorasib-treated mouse heart tissue (Fig. 3D, E).

To elucidate the role of NEU1 in sotorasib-mediated cardiomyocyte apoptosis, we employed two siRNAs targeting NEU1. Remarkably, NEU1 knockdown increased the survival rate of sotorasib-treated cardiomyocytes, accompanied by a significant decrease in c-PARP protein levels (Figs. 3F, G and S2E). Next, NEU1 knockdown attenuated sotorasib-induced apoptosis (Fig. S2D). To further validate the involvement of NEU1 in cardiac injury, we conducted NEU1-HA overexpression experiments in CCC-HEH-2 cells. NEU1 overexpression induced increased apoptosis and decreased survival of cardiomyocytes. In addition to this cardiomyocyte overexpression of NEU1 followed by sotorasib treatment further exacerbated apoptosis (Figs. 3H, I and S2F, G).

Previous studies have underscored strong associations between NEU1 and oxidative stress, cardiac hypertrophy, and cardiac dysfunction [21, 23, 31]. To confirm the impact of NEU1 on mitochondrial injury, we overexpressed the NEU1-HA plasmid in CCC-HEH-2 cells, which demonstrated that the increased NEU1 protein expression in cardiomyocyte led to an increase in ROS production and a decrease in mitochondrial membrane potential (Fig. S3A, B). In order to further confirm the findings, we overexpressed NEU1 and subsequently treated the CCC-HEH-2 cells with sotorasib. We discovered that overexpression of NEU1 aggravated sotorasib-induced mitochondrial injury (Fig. S3C, D). We then showed that the NEU1 knockdown dramatically reduced the negative effects of sotorasib on mitochondrial membrane potential, ROS generation, and ATP production (Fig. S3E–G). In conclusion, our results suggest that NEU1 homeostasis is closely related to mitochondrial function in cardiomyocytes and that increased NEU1 protein induces mitochondrial dysfunction.

### NEU1 promotes sotorasib-induced cardiomyocyte injury by inhibiting the AKT signaling pathway

**Fig. 4:**
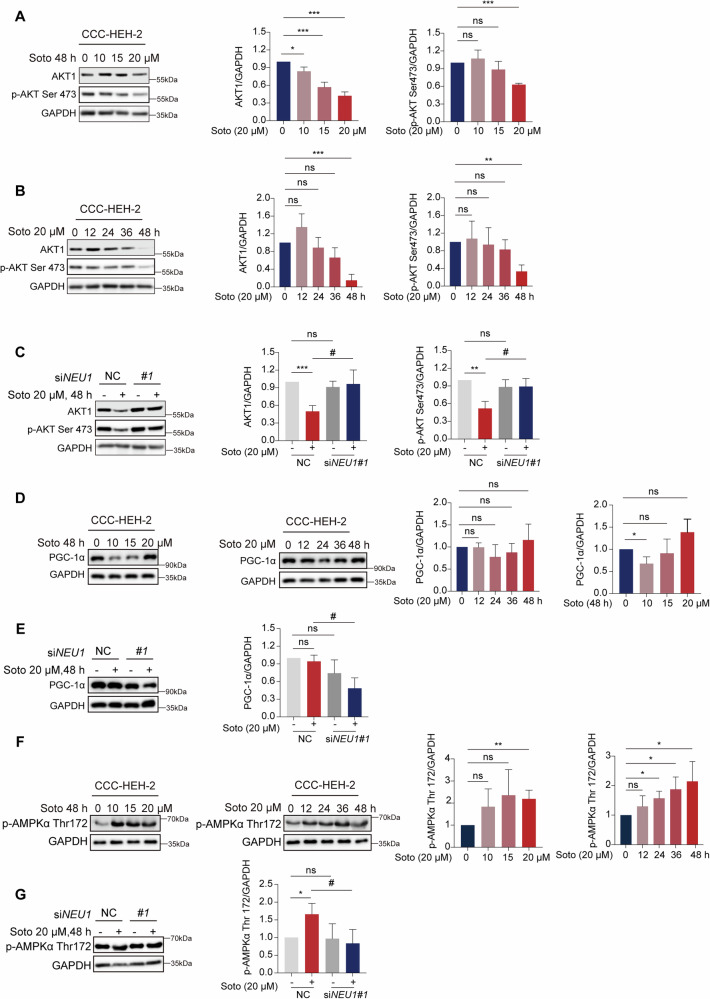
Accumulated NEU1 promotes cardiac injury by inhibiting AKT signaling. CCC-HEH-2 cells were treated with 20 μM sotorasib for 0, 12, 24, 36, and 48 h and exposed to different concentrations of 0, 10, 15, or 20 μM sotorasib for 48 h. **A**, **B** Expression levels of AKT1 and p-AKT Ser473 protein in CCC-HEH-2 cells (*n* = 3). **C** CCC-HEH-2 cells transfected with NC and NEU1#1 knockdown were treated with sotorasib for 48 h. Expression levels of AKT1 and p-AKT Ser473 protein in CCC-HEH-2 cells (*n* = 3). **D** CCC-HEH-2 cells were treated with 20 μM sotorasib for 0, 12, 24, 36, and 48 h and exposed to different concentrations of 0, 10, 15, or 20 μM sotorasib for 48 h. Expression levels of PGC-1α protein in CCC-HEH-2 cells (*n* = 3). **E** CCC-HEH-2 cells transfected with NC and NEU1#1 knockdown were treated with sotorasib for 48 h. Expression levels of PGC-1α protein in CCC-HEH-2 cells (*n* = 3). **F** CCC-HEH-2 cells were treated with 20 μM sotorasib for 0, 12, 24, 36, and 48 h and exposed to different concentrations of 0, 10, 15, or 20 μM sotorasib for 48 h. Expression levels of p-AMPK Thr172 protein in CCC-HEH-2 cells (*n* = 3). **G** CCC-HEH-2 cells transfected with NC and NEU1#1 knockdown were treated with sotorasib for 48 h. Expression levels of p-AMPK Thr172 protein in CCC-HEH-2 cells (*n* = 3). Data expressed as mean ± SD, ***, *p* < 0.0001, **, *p* < 0.01, *, *p* < 0.05 (vs. Vehicle), #, *p* < 0.05 (vs. sotorasib), ns, no significance.

We then investigate the mechanism by which NEU1 causes cardiomyocyte injury. We verified the known downstream signaling pathways of NEU1, as mentioned above. We examined AKT1 and p-AKT Ser473 levels by western blot, and the results indicate that both AKT1 and p-AKT1 Ser473 were inhibited by sotorasib in a time- and dose-dependent manner (Fig. 4A, B). To further validate the role played by NEU1 in this signaling pathway, we knocked down NEU1 and then treated CCC-HEH-2 cells with sotorasib, and the western blot results were consistent with the previous findings (Fig. 4C). It indicated that the AKT signaling pathway downstream of NEU1 was inhibited after sotorasib treatment, which led to cardiomyocyte death. Furthermore, we used western blot to detect both AMPK-SIRT3 and SIRT1/PGC-1α signaling pathways. The findings demonstrated that the protein level of PGC-1α in cardiomyocytes was unaffected by sotorasib treatment (Fig. 4D) and that NEU1 knockdown also had no influence on the level of PGC-1α in cardiomyocytes (Fig. 4E). p-AMPK-Thr172 expression kept unaltered by sotorasib treatment (Fig. 4F) or by NEU1 knockdown (Fig. 4G). In conclusion, our experiments suggest that sotorasib-induced NEU1 upregulation may regulate mitochondrial function and cardiomyocyte apoptosis through the PI3K/AKT signaling pathway, rather than the AMPK-SIRT3 or SIRT1/PGC-1α signaling pathways.

### Inhibition of ubiquitin-mediated degradation leads to the aberrant accumulation of NEU1 protein

**Fig. 5:**
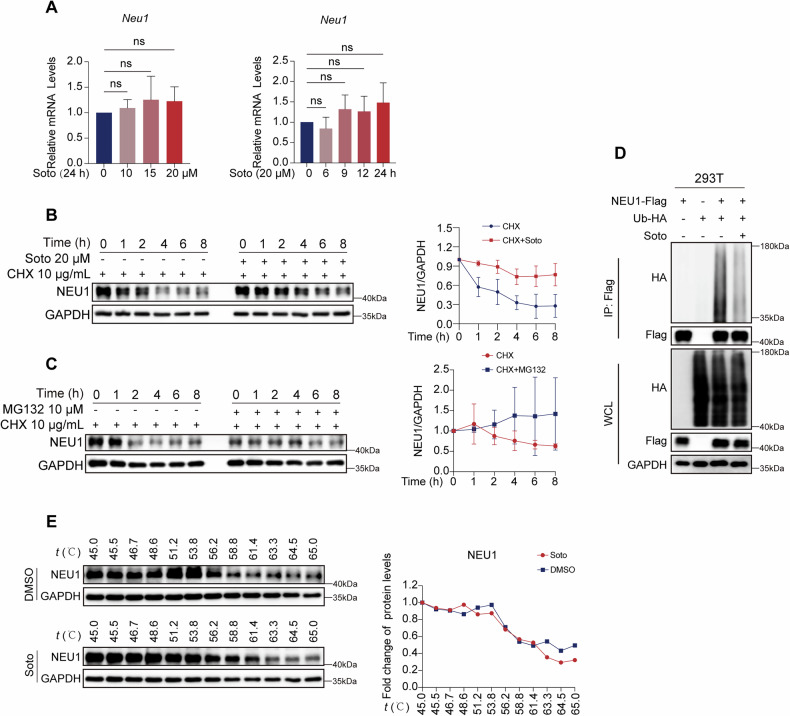
Sotorasib promotes the accumulation of NEU1 by blocking its ubiquitination degradation. **A** CCC-HEH-2 were treated with 20 μM sotorasib for 0, 6, 9, 12, or 24 h and exposed to different concentrations of 0, 10, 15, or 20 μM sotorasib for 24 h. CCC-HEH-2 NEU1mRNA level. (*n* = 3). **B**, **C** CHX (10 μg/mL), MG132 (10 μM), and /or sotorasib (20 μM) treated 0, 1, 2, 4, 6, 8 h. NEU1 protein expression level. **D** 293T cells were transfected with Ub-HA and/or NEU1-Flag and sotorasib was treated for 48 h. HA, Flag expression levels in 293T cells (*n* = 3). **E** CETSA, NEU1 expression level was verified in 293T cells. Data expressed as mean ± SD, ns, no significance.

Next, we endeavored to elucidate the mechanism underlying NEU1 accumulation following sotorasib treatment. Initially, we assessed alterations in NEU1 transcriptional levels subsequent to sotorasib administration using qRT-PCR. Our findings implied that *NEU1* transcriptional levels were not associated with sotorasib treatment (Fig. 5A). Next, we used cycloheximide (CHX), a protein synthesis inhibitor, to study protein degradation kinetics [32]. Western blot results revealed that sotorasib prolonged the half-life of NEU1, indicating that the increase in NEU1 protein level after sotorasib treatment was caused by the inhibition of its degradation. This effect was further corroborated by the combination of CHX and MG132, a protease inhibitor targeting various active proteases, which similarly prolonged NEU1 half-life [33], consistent with our previous observations, thus implicating the involvement of the ubiquitin-proteasome pathway in NEU1 degradation (Fig. 5B, C). In addition, the immunoprecipitation assay displayed that sotorasib significantly inhibited the ubiquitination of NEU1 (Fig. 5D). To ascertain whether sotorasib directly targeted NEU1 protein, we conducted CETSA experiments. However, our results from western blot analyses indicated that sotorasib did not directly interact with NEU1 (Fig. 5E). In summary, sotorasib-induced elevation of NEU1 induces cardiotoxicity not by affecting the transcriptional level of NEU1 but by inhibiting the ubiquitination-mediated degradation of this protein, which causes the aberrant accumulation of NEU1 in cardiomyocyte.

### D-PAC decreases NEU1 protein expression levels to rescue the cardiac injury induced by sotorasib

**Fig. 6:**
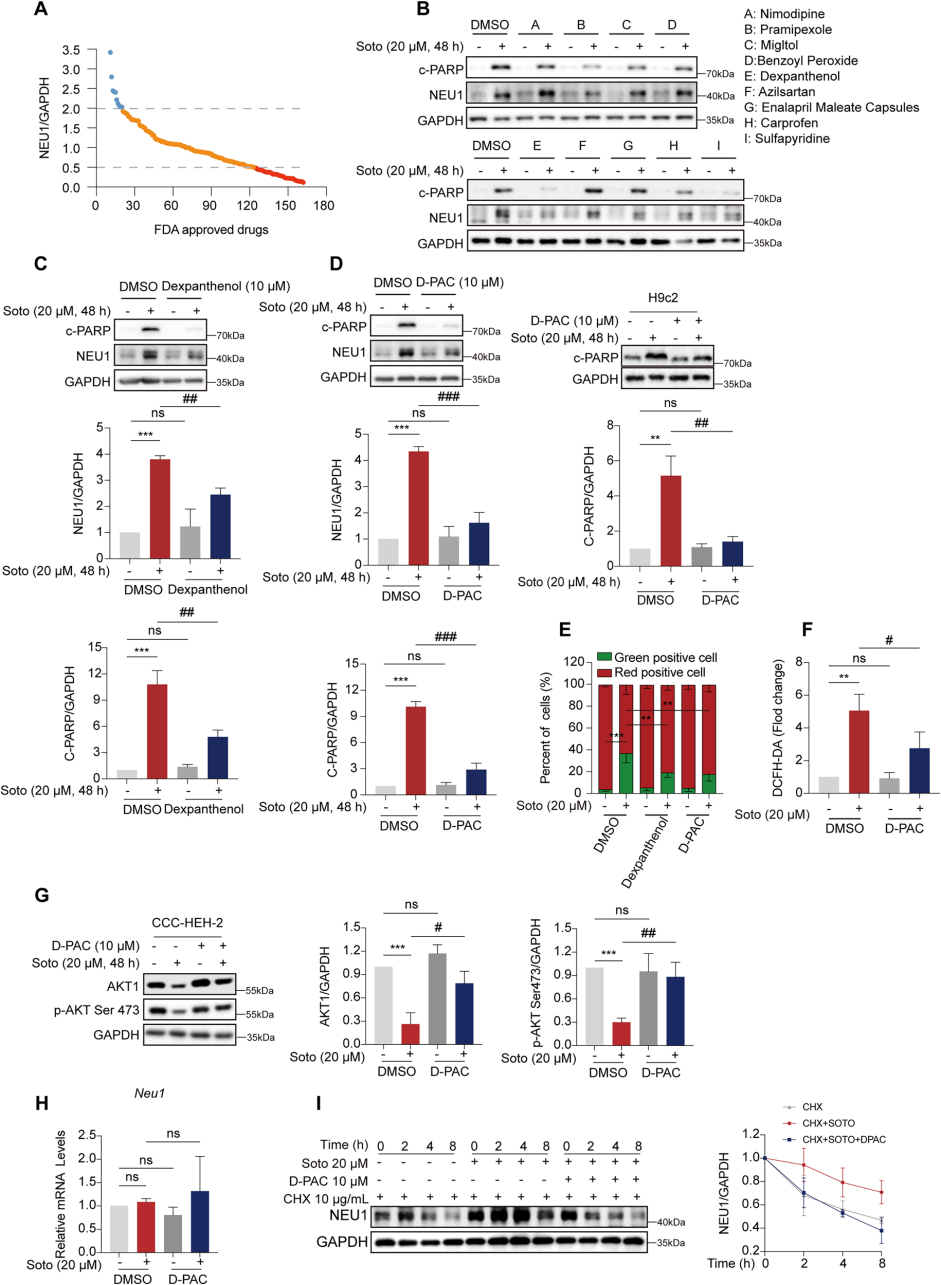
D-Pantothenic acid decreases NEU1 protein expression levels to rescue the cardiac injury induced by sotorasib. **A** NEU1 expression levels in CCC-HEH-2 cells treated with sotorasib 20 μM and/or FDA-approved drug 10 μM for 48 h. **B** CCC-HEH-2 cells were treated with potential 9 intervention strategies (Nimodipine, pramipexole, Benzoyl peroxide, Dexpanthenol, Azilsartan, Enalapril Maleate Capsules, Carprofen, Sulfadiazine) 10 μM and/or sotorasib 20 μM for 48 h. c-PARP and NEU1 protein expression levels were examined in cells (*n* = 3). CCC-HEH-2 cells and/or H9c2 cells were treated with D-PAC (10 μM) or dexpanthenol (10 μM) in combination with sotorasib 20 μM or 60 μM for 48 h. **C**, **D** c-PARP and NEU1 protein expression levels were examined in cells (n = 3). **E** CCC-HEH-2 cells were treated with D-PAC (10 μM) or dexpanthenol (10 μM) in combination with sotorasib 20 μM for 48 h. MMP changes in CCC-HEH-2 cells (*n* = 3). CCC-HEH-2 cells cells were treated with D-PAC (10 μM) in combination with sotorasib 20 μM for 48 h. **F** ROS levels in CCCHEH-2 cells (*n* = 3). **G** AKT1 and p-AKT Ser473 protein expression levels were examined in cells (*n* = 3). **H** CCC-HEH-2 NEU1mRNA level (*n* = 3). **I** CHX (10 μg/mL), sotorasib (20 μM), and/or D-PAC treated 0, 2, 4, 8 h. NEU1 protein expression level (*n* = 3). Data expressed as mean ± SD, ***, *p* < 0.0001, **, *p* < 0.01, (vs. Vehicle), ###, *p* < 0.001, ##, *p* < 0.01 #, *p* < 0.05 (vs. sotorasib), ns, no significance.

Based on the foregoing observations, it makes sense to conclude that NEU1 is a dependable target for the management of sotorasib cardiac damage. Employing western blot analysis, we evaluated the effects of 153 medications from the FDA-approved compound library on sotorasib-increased NEU1 protein level. The results showed that 39 medications significantly decreased NEU1 protein levels (NEU1/GAPDH < 0.5) (Fig. 6A). The 9 drugs with the greatest degree of downregulation were then rescreened, and the findings indicated that sulfapyridine, pramipexole, and dexpanthenol medicines were able to both lower c-PARP and reduce NEU1 to the normal level (Fig. 6B). Considering safety profiles and clinical combinatory strategies, dexpanthenol was selected for subsequent investigations. Previous studies have shown that dexpanthenol has protective and therapeutic effects on isoproterenol-induced cardiac injury in mice, albeit the precise underlying mechanisms remain elusive [29]. Building upon these antecedent findings, we hypothesized that dexpanthenol might have a cardioprotective effect by decreasing NEU1 expression and therefore reversing cardiomyocytes’ mitochondrial dysfunction. First, dexpanthenol significantly blocked the sotorasib-induced increase in NEU1 protein levels, while decreasing c-PARP levels in CCC-HEH-2 (Fig. 6C). Given the biological conversion of dexpanthenol to D-PAC, we further investigated the combinatory effects of D-PAC and sotorasib. Western blot analyses confirmed that D-PAC significantly reduced the expression levels of c-PARP and NEU1 in both CCC-HEH-2 and H9c2 cell lines (Fig. 6D). IF results demonstrated reduced NEU1 and cleaved-CASP3 expression in cardiomyocytes after dexpanthenol or D-PAC treatment, consistent with previous results (Fig. S4A). To further confirm the protective effect of dexpanthenol and D-PAC, we evaluated the survival rate of each treatment. Consistent with the previous results, dexpanthenol or D-PAC treatment could greatly rescue the cell morphological changes and apoptosis of cardiomyocytes induced by sotorasib (Fig. S4B–D). Surprisingly, we discovered that D-PAC or dexpanthenol rescued sotorasib-induced apoptosis in cardiomyocytes in a concentration-independent manner (Fig. S5A–E).

To investigate the specific mechanism of the protective effects of dexpanthenol and D-PAC, we used JC-1 staining and flow cytometry to detect changes in MMP after drug combination. Our findings revealed significant MMP reduction upondex panthenol or D-PAC treatment (Fig. 6E). Detection of ROS by flow cytometry revealed that D-PAC co-treatment with sotorasib reduced the sotorasib-mediated increase in ROS generation (Fig. 6F). These results suggest that in vitro conversion of dexpanthenol to D-PAC may rescue sotorasib-induced cardiomyocyte injury by reversing mitochondrial dysfunction.

Furthermore, to further validate whether D-PAC reverses sotorasib-induced cardiotoxicity through the AKT signaling pathway, we performed western blot analysis and found that D-PAC greatly ameliorated the sotorasib-induced reduction in AKT1 and p-AKT Ser 473 levels (Fig. 6G). To explore whether D-PAC modulates NEU1 protein levels, we performed qRT-PCR analysis, revealing no significant reduction in *NEU1* mRNA levels upon D-PAC treatment (Fig. 6H). However, combined findings with CHX demonstrated that D-PAC reversed sotorasib-induced inhibition of NEU1 degradation (Fig. 6I). These observations indicate that D-PAC mitigates sotorasib-induced cardiotoxicity by promoting NEU1 protein degradation rather than modulating *NEU1* mRNA transcript levels.

### Calcium pantothenate (Ca-PA) alleviates sotorasib-induced cardiac injury in mice

**Fig. 7:**
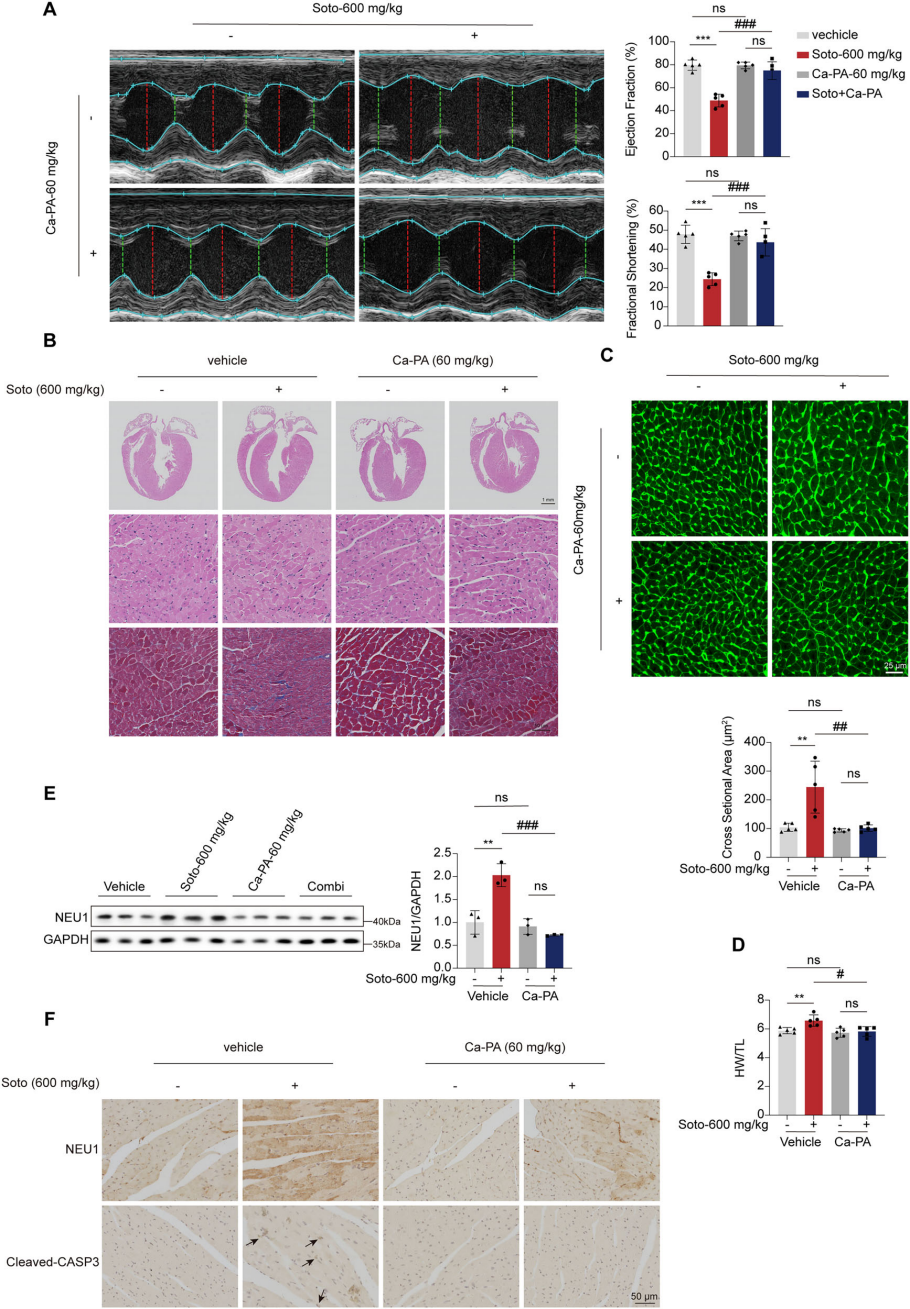
Ca-PA alleviates sotorasib-induced cardiac injury in mice. C57BL/6J male mice were treated with 600 mg/kg/d sotorasib and/or 60 mg/kg/d Ca-PA for 28 days. **A** Cardiac function was examined by echocardiography, showing the percentage of EF and FS (*n* = 5). **B** Representative images of mouse hearts stained for H&E, Sirius Red, and Masson. Scale bar 1 mm and 50 μm (*n* = 5). **C** Representative image and statistics of WGA staining. Scale bar: 25 μm (*n* = 5). **D** The levels of HW/TL of mice were analyzed (*n* = 5). **E** Expression levels of NEU1 in heart tissue (*n* = 3). **F** Representative images of IHC staining for cleaved-CASP3 and NEU1 in heart tissue. Scale bar: 50 μm (*n* = 5). Data expressed as mean ± SD, **, *p* < 0.01, (vs. Vehicle), ###, *p* < 0.0001, ##, *p* < 0.01 #, *p* < 0.05 (vs. sotorasib), ns, no significance.

The above work has demonstrated that D-PAC reduced cardiomyocyte apoptosis in vitro caused by sotorasib. We suggested conducting additional research to evaluate whether D-PAC reduced sotorasib’s cardiotoxicity in vivo. Given the instability of pantothenic acid, we proposed employing its more stable calcium salt, Ca-PA. Subsequent mouse cardiac ultrasound assessments revealed that Ca-PA co-administration significantly counteracted sotorasib-induced reductions in EF and FS (Fig. 7A). H&E staining results showed that Ca-PA reversed back sotorasib-induced cardiomyocyte injury. Masson staining indicated that Ca-PA ameliorated sotorasib-mediated cardiac fibrosis in mice (Fig. 7B). Notably, WGA staining further illustrated that combined Ca-PA treatment exhibited significant attenuation of cardiomyocyte hypertrophy compared to sotorasib monotherapy (Fig. 7C). HW/TL indicated that sotorasib-induced cardiac enlargement was reversed by Ca-PA (Fig. 7D). IHC and western blot analyses corroborated the synergistic effects of Ca-PA and sotorasib co-treatment, evidenced by reduced NEU1 protein expression levels and concurrent reversal of cleaved-CASP3 expression (Fig. 7E, F). In addition, diminished levels of *Nppa*, *Nppb*, and *Myh6* mRNA following co-treatment underscored the cardioprotective effects of Ca-PA (Fig. S6A). To ascertain the impact of Ca-PA on sotorasib’s anticancer activity, we evaluated the combination’s effects on A549 lung cancer cells, revealing no interference with sotorasib’s anticancer efficacy (Fig. S6B, C). In summary, Ca-PA can alleviate sotorasib-induced cardiotoxicity, and therefore Ca-PA may be a potential preventive strategy and therapeutic approach for the clinical management of sotorasib-induced cardiac damage.

## Discussion

In this study, we first revealed the critical role of NEU1 in the cardiac injury of sotorasib. We discovered that NEU1 caused myocardial injury by causing mitochondrial dysfunction. More importantly, we found that Ca-PA mitigated the cardiotoxicity of sotorasib by decreasing the protein levels of NEU1 and did not affect its antitumor activity.

Beyond its classical functions in lysosomal metabolism, NEU1 exhibits cell surface localization, contributing to the structural and functional modulation of cellular receptors. Previous studies have highlighted elevated NEU1 expression in cardiomyocytes, particularly in hypertrophic myocardium models in rodents and humans [21]. Our research extended these observations, revealing an association between increased NEU1 protein levels induced by sotorasib and the development of cardiac hypertrophy. In doxorubicin-induced cardiotoxicity, NEU1 enhances mitochondrial division and PINK1/Parkin-mediated mitochondrial autophagy through enhanced Drp1 expression [31]. However, in our study, we discovered that the cardiac damage of sotorasib was not due to enhanced mitochondrial autophagy but rather to mitochondrial dysfunction leading to lower MMP, decreased ATP generation, and increased ROS production. Previous mechanistic studies have shown that NEU1 undergoes nuclear translocation in response to the stimulation of pressure overload, selectively binds GATA4 after entering the nucleus, promotes the binding of GATA4 to the promoters of embryonic genes, the *Nppa* and *Nppb* genes, and activates their transcription and expression, which in turn induces myocardial injury [21]. In this study, we identified a novel mode of NEU1 accumulation, the ubiquitin-proteasome pathway. Instead of boosting transcription, we found that sotorasib blocks the processing of NEU1 ubiquitination and degradation, which leads to an excessive accumulation of NEU1 protein in cardiomyocyte. Thus, our study not only elucidated the mechanism of sotorasib cardiotoxicity but also enriched the regulatory system of NEU1 protein stability.

Recent research has revealed that NEU1 plays an important role in cardiovascular disease [34]. Literature reports that NEU1 inhibits downstream PI3K/AKT signaling [24]. We found that AKT1 and p-AKT Ser473 expression were reduced after sotorasib treatment. In addition, we further demonstrated that the reduction of AKT1 and p-AKT Ser 473 was due to the elevated expression of NEU1. The PI3K-AKT signaling pathway plays an important role in regulating cell growth and metabolic processes [35–37]. In addition to this, the activation of this signaling pathway also functions in cardioprotection and nerve regeneration [38]. Our study showed that the co-administration of D-PAC alleviated the sotorasib-induced reduction of AKT1 and p-AKT Ser473. However, the PI3K/AKT/mTOR pathway is one of the downstream pathways of RKAS [39, 40]. Thus, decreasing NEU1 protein levels may lead to activation of the AKT signaling pathway, which promotes tumor growth. We found that D-PAC did not reduce the therapeutic effect of sotorasib while activating the AKT signaling pathway in cardiomyocytes. Previous studies have shown that elevated NEU1 expression in myocardial infarction tissues leads to inhibition of the SIRT1/PGC-1α pathway, thereby leading to altered mitochondrial function causing cardiac dysfunction. However, our study identified that sotorasib treatment did not cause SIRT1/PGC-1α inhibition. In diabetic cardiomyopathy, AMPK1α/sirt3 inhibition is associated with upregulation of NEU1. AMPK is activated under low ATP conditions, inhibiting anabolism, promoting catabolism, and inhibiting cell growth while promoting ATP, thus maintaining homeostatic energy metabolism in the cell [41, 42]. The expression of p-AMPK Thr172 was slightly elevated after sotorasib administration, and NEU1 knockdown reversed the elevated expression levels of this protein, which may result from ATP depletion after sotorasib treatment.

The tumor microenvironment is closely related to tumor development. It has been reported that NEU1 modifies the tumor microenvironment and alters the tumor microenvironment by shearing the salivary acid modification of LAMP1, thereby regulating tumor development and drug resistance [43]. NEU1 varies in different cancer types. In bladder and gastric cancers, NEU1 overexpression promotes tumor cell apoptosis [44, 45]. In ovarian, pancreatic, hepatocellular and melanoma cancers, inhibition of NEU1 is a potential target for tumor therapy [45–48]. Notably, p53 mutant (p53-R273H) promotes NSCLC proliferation and migration by upregulating NEU1 [49]. This suggests that downregulation of NEU1 is a therapeutic strategy for NSCLC, which is not contrary to our view of NEU1 inhibitors as a co-administration strategy for sotorasib cardiotoxicity. Our investigation demonstrated that treatment of A549 with the NEU1 inhibitor D-PAC slightly inhibited the clone-forming ability of tumor cells, and that combination with sotorasib did not affect the antitumor activity of sotorasib further supports the above hypothesis.

Previous reports have shown that D-PAC has therapeutic and protective effects against isoproterenol-induced cardiotoxicity, but the specific mechanisms remain unclear. We demonstrated in vivo and in vitro that both dexpanthenol and D-PAC reverse cardiac mitochondrial dysfunction by decreasing NEU1, a key cardiac regulator, thereby exerting cardioprotective effects. We discovered that D-PAC did not reduce sotorasib-induced cardiotoxicity in a dose-dependent manner. At 5 μM, 10 μM, and 20 μM, there was no discernible difference in the rescue of cardiomyocyte injury between D-PAC and panthenol; however, at 40 μM, the rescue effect was slightly diminished, which may be related to the cytotoxicity of high degree of drug l. Notably, D-PAC, as a cardiotoxicity-protective agent, does not affect the cancer effects of sotorasib (Fig. 7).

KRAS serves as a therapeutic target for cancer, but there are only a handful of KRAS gene-targeted drugs due to their “undruggable”. Sotorasib, a KRAS G12C inhibitor, achieved disease control rates of 88.1% in the treatment of NSCLC and 84% in the treatment of pancreatic cancer [3, 4]. However, cardiotoxicity may not be widely recognized because sotorasib currently has a small number of clinical uses. By combining data mining analysis with clinical trials, we found that sotorasib results in pericardial effusion and cardiac arrest [50–52]. A number of conditions may come together to produce cardiac arrest. The most frequent causes are myocardial infarction, cardiomyopathy, heart failure, and arrhythmia. Pericardial effusion is also strongly associated with heart failure [53, 54]. According to our research, sotorasib decreases heart function markers such as EF and FS. Cardiomyocyte hypertrophy brought on by sotorasib may ultimately result in heart failure. Additionally, we discovered that mitochondrial dysfunction brought on by sotorasib plays a significant role in heart failure. Therefore, our study not only reveals the mechanism of sotorasib-induced cardiotoxicity but also provides a safe and effective protection strategy for the clinical use of sotorasib. However, the association between sotorasib-induced cardiomyocyte mitochondrial dysfunction and pericardial effusion was not clear in our study. Moreover, the precise mechanism by which pantothenic acid mitigates sotorasib’s cardiotoxicity remains incompletely understood. Thus, the two points mentioned above may be the subject of future research.

## Materials and methods

### Animals and drug treatment

Six- to eight-week male C57BL/6J mice were purchased from Beijing Vital River Laboratory. All experiments were performed according to the protocol approved by the Institute of Artificial Intelligence Medical Innovation Research of Zhejiang University (approval number DW202311092319). The mice were kept in the experimental animal house with constant humidity and temperature, a 12-h light-dark cycle, and free access to water and food. Mice were randomly grouped after one week of stabilization. Administer the drug according to the blind method. Sotorasib (CAS 2296729-00-3, Topscience, Shanghai, China) and calcium pantothenate tablets (Tianjin Lixiang Pharmaceutical Co., Ltd.) were dissolved in 0.5% sodium carboxymethylcellulose (CMC-Na) (419273, Sigma-Aldrich, St. Louis, MO, USA) to obtain the stock solution. In the sotorasib cardiotoxicity study, mice were administered 0.5% CMC-Na, 300 mg/kg/d and 600 mg/kg/d sotorasib per day for 28 days via intragastric administration; for the assessment of Ca-PA (a mixture of D and L types) in the sotorasib cardiotoxicity prophylaxis and protection study, mice were administered 600 mg/kg/d sotorasib per day via gastric administration and/or Ca-PA 60 mg/kg/d, and another group of mice was administered 0.5% CMC-Na by gavage for 28 days. After 28 days, mice were anesthetized with 2% isoflurane and killed by decapitation before removal of tissue. The sample size of each group was 10 mice, and the remaining mice were included in the statistical analysis by excluding mice that died during the course of the experiment or had accidents that were clearly unrelated to the experiment.

### Biochemistry assessment and histology

Under isoflurane anesthesia, blood was withdrawn from the retro-orbital plexus of mice and precipitated for 2 h, then centrifuged at 3500×*g* for 15 min. The supernatant was then collected for analysis. Creatine kinase isoenzyme (CK-MB), Creatine Kinase (CK) and lactate dehydrogenase (LDH) levels in the supernatant were detected using a chemical analyzer (XN-1000V, Sysmex). Cardiac tissues were retained in four chambers for processing and sent to Haoke Biotechnology Co. for hematoxylin and eosin (H&E), Masson staining and Sirius Red staining.

### Cell culture

CCC-HEH-2 human embryonic cardiac tissue-derived cell lines (RRID: CVCL_VU29) were established from male human embryonic tissues at 11 weeks by the National Infrastructure of Cell Line Resource of China, and H9c2 rat embryonic heart tissue-derived cell lines were purchased from Jennio Biological Technology (Guangzhou, China). Short tandem repeat (STR) analysis has been performed, and tested for mycoplasma contamination. The HEK293T was supplied by the Institute of Biochemistry and Cell Biology. A549 cells were obtained from the Cell Bank of China Science Academy (Shanghai, China). These cardiomyocytes and HEK293T were cultured in DMEM (10569010, Gibco) while A549 cells were maintained in RPMI-1640 (21870076, Gibco) containing 10% fetal bovine serum (FBS, 16140071, Gibco) and penicillin/streptomycin in a humidified atmosphere with 5% CO_2_ at 37 °C. Each set of cell experiments was replicated three times.

Materials: Sotorasib (T8684, Topscience, Shanghai, China), Z-VAD-FMK (T8541, Topscience, Shanghai, China), dexpanthenol (T1437, Topscience, Shanghai, China), D-PAC (T6480, Topscience, Shanghai, China), MG132 (M8699, Sigma-Aldrich). The FDA-approved drugs and natural products used for drug screening were sourced from Topscience (Shanghai, China) and Nantong Jingwei Biotechnology Co. Ltd. (Nantong, Jiangsu, China), respectively.

### Western blot analysis

Proteins were lysed with a lysis buffer mixture consisting of 150 mM NaCl, 50 mM Tris-HCl, 2 mM EGTA, 2 mM EDTA, 25 mM sodium glycerophosphate, 25 mM NaF, 0.3% Triton X-100, 0.3% NP-40, 0.3% Leupeptin, 0.1% NaVO3, and 0.1% PMSF. The lysis products were plated on SDS-PAGE gels at 8%, 10%, or 12%, then transferred to PVDF membranes provided by Millipore and incubated with 4% bovine serum albumin (BSA, B2064, Sigma-Aldrich) solution. Subsequently, the PVDF membrane was washed three times for 5 min each in PBS (T-PBS) containing 0.1% Tween-20. The membranes were then incubated for 1 h with HRP-labeled secondary antibodies corresponding to the properties of the secondary antibodies ((GAR007, GAM007, 1:1000) supplied by Liancor Bio). After washing three times in T-PBS as before, the bands were detected by exposure using the ECL-Plus kit (P2300, NCM Biotech) and visualized using an Amersham Imager 600 (General Electric). Protein quantification was performed using Image J (version 1.8.0). Relevant antibodies are shown in Table S2.

### Quantitative real-time polymerase chain reaction (qRT-PCR) analysis

After the cells were treated accordingly, the cells were collected, and RNA was isolated using the FastPure Cell/Tissue Total RNA Isolation Kit V2 (RC112, Vazyme Inc.) according to the kit instructions. The RNA was then uniformly reverse-transcribed into cDNA for subsequent qRT-PCR experiments. qRT-PCR amplification was performed in two steps, the first step was an initial phase of 3 min at 95 °C, followed by 39 cycles of 3 s at 95 °C and 31 s at 60 °C. Fold change in gene expression was determined by the comparative threshold cycling (Ct) method using the 2−(ΔΔCt) formula. The primer sequences provided by Youkang Biotechnology Co. are detailed in Table S3.

### Sulforhodamine B staining

For the study of cell viability, Sulforhodamine B (SRB; S1402, Sigma-Aldrich) was used. Cells inoculated in 96-well plates were treated accordingly cardiomyocyte in 96-well plates were fixed with 10% trichloroacetic acid (T104257, Aladdin), and then cells were treated with 4 mg/mL SRB for 30 min. The plates were then washed with 1% acetic acid (1000218, Sinopharm) configured in tap water. The 96-well plates were dried in an oven at 65 °C, and the residual SRB dye was dissolved in 10 mM unbuffered Tris base (1115KG001, Biofroxx) after the 96-well plates were cooled. The absorbance at 515 nm was measured using a multi-scan spectrometer (Tecan).

### Transfection of siRNA oligonucleotides

Transfect siRNA oligonucleotides using Oligofectamine™ Transfection Reagent (12252011, Invitrogen). After the cells had grown to approximately 50% fusion, removed the medium from the 12-well plate and replaced it with transfection solution containing Opti-MEM™ (31985070, Gibco). After 6 h of transfection in the incubator at 37 °C, the transfection medium was replaced with complete medium, and the cells were grown for 12 h. Cells were treated with drugs according to the experiments. siRNAs and their negative controls (NC) were purchased from GenePharma. siRNA sequences are shown in Table S4.

### Plasmid construction and transfection

pCMV-NEU1 was purchased from Miaolingbio (Wuhan, China), and cells were transfected with plasmids according to the manufacturer’s instructions using jetPRIME (101000046, Polyplus-transfection, Inc. Illkirch, France) for plasmid transfection of cells. Cells were seeded into specific dishes or plates, and cells were grown to ≈70% fusion. Cells were then transfected with transfection reagents. The ratio of the plasmid to reagent was 1 µg:2 μL, and after 6 h of transfection, the transfection reagent was discarded and replaced with a fresh culture medium to continue the culture or for drug administration.

### Flow cytometry analysis

The percentage of apoptotic cells was assayed with Apoptosis Detection Kit I (C1062L, Beyotime). Cells to be assayed were digested with trypsin and centrifuged, and the cells were stained with annexin V-PI for more than 20 min. 1 × 10^4^ cells in each sample were detected by flow cytometry (BD Biosciences). The gating strategy for this classification was recorded using BD CellQuest Pro software (version 5.1).

MMP was assessed with the JC-1 staining kit (C2005, Beyotime). Samples to be tested were digested with trypsin, and cells were collected and rinsed with PBS. Cells were then stained with 5 µM JC-1 for 30 min at 37 °C in the dark according to the kit instructions. For the positive control, 10 µM CCCP was applied 20 min before the assay, and the cells were also placed in the dark at 37 °C for 30 min. 1 × 10^4^ cells were prepared and measured using a flow cytometer (BD Biosciences).

Intracellular ROS generation and accumulation were detected using the Reactive Oxygen Species Assay Kit (S0033S, Beyotime). Samples to be tested were processed, cells were digested with trypsin and collected, and incubated for 20 min at 37 °C in the dark in serum-free medium containing the DCFH-DA probe, which was diluted at a ratio of 1:1000, according to the kit instructions. For the positive control group, 50 µg/mL of Rohypnol was added for 30 min prior to loading the DCFH-DA probe. Subsequently, 1 × 10^4^ cells were analyzed by ROS flow cytometry using a BD FACSCalibur™ flow cytometer (BD Biosciences).

### ATP detection

Intracellular ATP levels were assayed using the ATP Assay Kit (S0027, Beyotime). Cells were treated with sotorasib, the culture solution was discarded, rinsed with PBS, and the cells were lysed using the ATP lysis buffer provided in the kit. After centrifugation at 4 °C, the supernatant of the cell lysate was collected. The ATP assay solution was then prepared according to the kit instructions, and samples were added at room temperature and protected from light. Dispense 20 µL of each sample or standard into a black-bottomed 96-well plate and add to the prepared ATP Assay Solution (165305, Thermo Fisher Scientific). Quantify the relative light units (RLU) using a multifunctional microplate detector (Tecan).

### Immunofluorescence (IF)

Cardiomyocytes were fixed with 4% paraformaldehyde (PFA, P6148, Sigma-Aldrich) for 15 min and then permeabilized with 0.3% Triton X-100 (1139ML100, Biofroxx) for 10 min. PBS was rinsed 3 times and blocked with 4% BSA for 30 min. The primary antibody was then configured according to the instructions for the target antibody, and the samples and the target protein were incubated overnight at 4 °C. The samples were washed three times with PBS and then incubated at 4 °C for 10 min. After washing three more times with PBS, the samples were treated with Alexa Fluor 488 or Alexa Fluor 568-conjugated secondary antibodies (A21202, A10042, A31573, Thermo Fisher Scientific) for 1 h at room temperature, and then the nuclei were stained with DAPI (D212, Dojindo) for 7 min. Primary antibodies used included anti-cleaved-Caspase-3 (Asp175) (9664S, 1:300 Cell Signaling Technology) and anti-NEU1 (sc-166824, 1:00, Santa Cruz Biotechnology). Fluorescence images were taken using a fluorescence microscope (IX81-FV1000, Olympus).

### Immunohistochemistry staining (IHC)

Cardiac tissues were treated with fresh 4% PFA and sectioned after paraffin embedding. The paraffin-embedded sections were placed in an oven at 65 °C for 1 h, and then the tissue sections were de-paraffinized with xylene and rehydrated with different concentrations of ethanol. Antigen repair was completed after rehydration for 20 min. Sections were subsequently treated with 3% H_2_O_2_ (PV-6001, ZSGB-BIO) for 15 min in a rehydration chamber and blocked with 5% goat serum (16210064, Gibco) for 30 min. Sections were incubated overnight in the humidor with specific antibodies: NEU1 (sc-166824, Santa Cruz Biotechnology) and cleaved-Caspase-3 (Asp175) (9664S, Cell Signaling Technology), configured according to the antibody instructions. After 3 min, sections were further incubated with enzyme-linked secondary antibody (PV-6001, ZSGB-BIO) for 1 h. Staining was completed with DAB colorant (ZLI-9017, ZSGB-BIO) configured according to the instruction manual, and the nuclei were counterstained with hematoxylin (C0107, Beyotime) for a brief period of 7 s. Images were scanned with a light microscope (Olympus).

### Cellular thermal shift assay (CETSA)

HEK293T cells were lysed with RIPA lysate (C1053, Applygen, Beijing, China) on ice for 30 min and centrifuged at 13,000 rpm at 4 °C for 10 min. 80 μL of 293T cell lysate was mixed with DMSO, sotorasib, or D-PAC to give a final concentration of 10 μM. The mixture was incubated at different temperatures (45.0 °C, 45.5 °C, 46.7 °C, 48.6 °C, 51.2 °C, 53.8 °C, 56.2 °C, 58.8 °C, 61.4 °C, 64.5 °C, 65.0 °C) for 6 min. The supernatant was centrifuged at 12,000 rpm for 10 min. NEU1 expression in the supernatant was analyzed by SDS-PAGE.

### Echocardiography

To assess cardiac function in mice, two-dimensional echocardiography was performed in awake mice using a Vevo 1100 imaging system. Briefly, the area where the mouse heart was located was dehiscence red and then gently immobilized on a 37 °C thermostatic plate for cardiac echocardiography. The diastolic left ventricular internal diameters (LVIDd) or systolic left ventricular internal diameters (LVIDs), as well as end-diastolic volumes (EDV) or end-systolic volumes (ESV), were measured and analyzed using VisualSonics software. LVEF was calculated using the following formula: EF (%) = [(EDV − ESV)/EDV] × 100. LVFS was calculated using the following formula: FS (%) = [(LVIDd) / ESV] × 100 = [(LVIDd − LVIDs)/LVIDd] × 100.

### Proteomics

Cells were collected in cryopreservation tubes according to experimental requirements, and sample processing and information analysis were carried out by Beijing Novozymes. Samples were processed and analyzed by protein extraction, protease desalting, mass spectrometry and Lable-free quantitative proteomics. The screening of significantly differentially expressed proteins was carried out according to the criteria of 1.5-fold or more change in fold expression (upregulation greater than 1.5-fold or downregulation less than 0.67-fold) and *p*-value < 0.05.

### Clone formation assay

1000 cells per well were inoculated in a 6-well plate, and after the cells were attached to the wall, the drug was administered according to the experimental needs. The culture was incubated for 14 days, and the fresh culture medium was replaced every 3 days with drug administration treatment. At the end of the experiment, the cells were fixed with 4% PFA at 4 °C, washed the PFA and dried the water in the oven, then stained with SRB dye for 30 min after it cooled down and photographed with a digital camera.

### Statistical analysis

Data are expressed as mean ± standard deviation (SD). A significant difference was considered when the *p*-value was less than 0.05. Student’s *t*-test was used when assessing significant differences between two groups, while one-way ANOVA was used when comparisons involving three or more groups were made. Data analysis was performed using Microsoft Excel (version 1808), Image J (version 1.8.0) and GraphPad.

## Supplementary information

supplementary materials-WB

supplementary materials

## Data Availability

All data created or analyzed in this study are available from the corresponding authors upon reasonable request.

## References

[1] Blair HA. Sotorasib: first approval. Drugs. 2021;81:1573–79. 34357500 10.1007/s40265-021-01574-2 PMC8531079

[2] de Langen AJ, Johnson ML, Mazieres J, Dingemans A-MC, Mountzios G, Pless M, et al. Sotorasib versus docetaxel for previously treated non-small-cell lung cancer with KRASG12C mutation: a randomised, open-label, phase 3 trial. Lancet. 2023;401:733–46. 36764316 10.1016/S0140-6736(23)00221-0

[3] Strickler JH, Satake H, George TJ, Yaeger R, Hollebecque A, Garrido-Laguna I, et al. Sotorasib in KRAS p.G12C-mutated advanced pancreatic cancer. N Engl J Med. 2023;388:33–43. 36546651 10.1056/NEJMoa2208470 PMC10506456

[4] Hong DS, Fakih MG, Strickler JH, Desai J, Durm GA, Shapiro GI, et al. KRASG12C inhibition with sotorasib in advanced solid tumors. N Engl J Med. 2020;383:1207–17. 32955176 10.1056/NEJMoa1917239 PMC7571518

[5] Varga ZV, Ferdinandy P, Liaudet L, Pacher P. Drug-induced mitochondrial dysfunction and cardiotoxicity. Am J Physiol Heart Circ Physiol. 2015;309:H1453–67. 26386112 10.1152/ajpheart.00554.2015 PMC4666974

[6] Qi B, He L, Zhao Y, Zhang L, He Y, Li J, et al. Akap1 deficiency exacerbates diabetic cardiomyopathy in mice by NDUFS1-mediated mitochondrial dysfunction and apoptosis. Diabetologia. 2020;63:1072–87. 32072193 10.1007/s00125-020-05103-w

[7] Del Re DP, Amgalan D, Linkermann A, Liu Q, Kitsis RN. Fundamental mechanisms of regulated cell death and implications for heart disease. Physiol Rev. 2019;99:1765–817. 31364924 10.1152/physrev.00022.2018 PMC6890986

[8] Sun L, Wang H, Xu D, Yu S, Zhang L, Li X. Lapatinib induces mitochondrial dysfunction to enhance oxidative stress and ferroptosis in doxorubicin-induced cardiomyocytes via inhibition of PI3K/AKT signaling pathway. Bioengineered. 2022;13:48–60. 34898356 10.1080/21655979.2021.2004980 PMC8805895

[9] Kumar AA, Kelly DP, Chirinos JA. Mitochondrial dysfunction in heart failure with preserved ejection fraction. Circulation. 2019;139:1435–50. 30856000 10.1161/CIRCULATIONAHA.118.036259 PMC6414077

[10] Liu XY, Peng J, He F, Tursun X, Li SP, Xin XL, et al. Shabyar ameliorates high glucose induced retinal pigment epithelium injury through suppressing aldose reductase and AMPK/mTOR/ULK1 autophagy pathway. Front Pharmacol. 2022;13:852945. 35620285 10.3389/fphar.2022.852945 PMC9127207

[11] Wu L, Wang L, Du Y, Zhang Y, Ren J. Mitochondrial quality control mechanisms as therapeutic targets in doxorubicin-induced cardiotoxicity. Trends Pharmacol Sci. 2023;44:34–49. 36396497 10.1016/j.tips.2022.10.003

[12] Dhingra R, Rabinovich-Nikitin I, Rothman S, Guberman M, Gang H, Margulets V, et al. Proteasomal degradation of TRAF2 mediates mitochondrial dysfunction in doxorubicin-cardiomyopathy. Circulation. 2022;146:934–54. 35983756 10.1161/CIRCULATIONAHA.121.058411 PMC10043946

[13] Yan G, Han Z, Kwon Y, Jousma J, Nukala SB, Prosser BL, et al. Integrated stress response potentiates ponatinib-induced cardiotoxicity. Circ Res. 2024;134:482–501. 38323474 10.1161/CIRCRESAHA.123.323683 PMC10940206

[14] Cadenas S. Mitochondrial uncoupling, ROS generation and cardioprotection. Biochim Biophys Acta Bioenerg. 2018;1859:940–50. 29859845 10.1016/j.bbabio.2018.05.019

[15] Su L, Zhang J, Gomez H, Kellum JA, Peng Z. Mitochondria ROS and mitophagy in acute kidney injury. Autophagy. 2023;19:401–14. 35678504 10.1080/15548627.2022.2084862 PMC9851232

[16] Bao MH, Li JM, Zhou QL, Li GY, Zeng J, Zhao J, et al. Effects of miR‑590 on oxLDL‑induced endothelial cell apoptosis: roles of p53 and NF‑κB. Mol Med Rep. 2016;13:867–73. 26648441 10.3892/mmr.2015.4606

[17] Zhang JY, Chen QQ, Li J, Zhang L, Qi LW. Neuraminidase 1 and its inhibitors from Chinese herbal medicines: an emerging role for cardiovascular diseases. Am J Chin Med. 2021;49:843–62. 33827385 10.1142/S0192415X21500403

[18] Chen Y, Hu J, Chen Y. Platelet desialylation and TFH cells-the novel pathway of immune thrombocytopenia. Exp Hematol Oncol. 2021;10:21. 33722280 10.1186/s40164-021-00214-5 PMC7958461

[19] Lee C, Liu A, Miranda-Ribera A, Hyun SW, Lillehoj EP, Cross AS, et al. NEU1 sialidase regulates the sialylation state of CD31 and disrupts CD31-driven capillary-like tube formation in human lung microvascular endothelia. J Biol Chem. 2014;289:9121–35. 24550400 10.1074/jbc.M114.555888 PMC3979388

[20] Lillehoj EP, Hyun SW, Feng C, Zhang L, Liu A, Guang W, et al. NEU1 sialidase expressed in human airway epithelia regulates epidermal growth factor receptor (EGFR) and MUC1 protein signaling. J Biol Chem. 2012;287:8214–31. 22247545 10.1074/jbc.M111.292888 PMC3318723

[21] Chen QQ, Ma G, Liu JF, Cai YY, Zhang JY, Wei TT, et al. Neuraminidase 1 is a driver of experimental cardiac hypertrophy. Eur Heart J. 2021;42:3770–82. 34179969 10.1093/eurheartj/ehab347

[22] Guo Z, Fan D, Liu FY, Ma SQ, An P, Yang D, et al. NEU1 regulates mitochondrial energy metabolism and oxidative stress post-myocardial infarction in mice via the SIRT1/PGC-1 alpha axis. Front Cardiovasc Med. 2022;9:821317. 35548408 10.3389/fcvm.2022.821317 PMC9081506

[23] Guo Z, Tuo H, Tang N, Liu F-, Ma SQ, An P, et al. Neuraminidase 1 deficiency attenuates cardiac dysfunction, oxidative stress, fibrosis, inflammatory via AMPK-SIRT3 pathway in diabetic cardiomyopathy mice. Int J Biol Sci. 2022;18:826–40. 35002528 10.7150/ijbs.65938 PMC8741837

[24] Hyun SW, Imamura A, Ishida H, Piepenbrink KH, Goldblum SE, Lillehoj EP. The sialidase NEU1 directly interacts with the juxtamembranous segment of the cytoplasmic domain of mucin-1 to inhibit downstream PI3K-Akt signaling. J Biol Chem. 2021;297:101337. 34688655 10.1016/j.jbc.2021.101337 PMC8591358

[25] Yao L, Liang X, Liu Y, Li B, Hong M, Wang X, et al. Non-steroidal mineralocorticoid receptor antagonist finerenone ameliorates mitochondrial dysfunction via PI3K/Akt/eNOS signaling pathway in diabetic tubulopathy. Redox Biol. 2023;68:102946. 37924663 10.1016/j.redox.2023.102946 PMC10661120

[26] Corinti D, Chiavarino B, Scuderi D, Fraschetti C, Filippi A, Fornarini S, et al. Molecular properties of bare and microhydrated vitamin B5-calcium complexes. Int J Mol Sci. 2021;22:692. 33445631 10.3390/ijms22020692 PMC7826572

[27] Dibble CC, Barritt SA, Perry GE, Lien EC, Geck RC, DuBois-Coyne SE, et al. PI3K drives the de novo synthesis of coenzyme A from vitamin B5. Nature. 2022;608:192–98. 35896750 10.1038/s41586-022-04984-8 PMC9352595

[28] Depeint F, Bruce WR, Shangari N, Mehta R, O’Brien PJ. Mitochondrial function and toxicity: role of the B vitamin family on mitochondrial energy metabolism. Chem Biol Interact. 2006;163:94–112. 16765926 10.1016/j.cbi.2006.04.014

[29] Kalkan F, Parlakpinar H, Disli OM, Tanriverdi LH, Ozhan O, Polat A, et al. Protective and therapeutic effects of dexpanthenol on isoproterenol-induced cardiac damage in rats. J Cell Biochem. 2018;119:7479–89. 29775243 10.1002/jcb.27058

[30] Wang H, Duan C, Keate RL, Ameer GA. Panthenol citrate biomaterials accelerate wound healing and restore tissue integrity. Adv Healthc Mater. 2023;12:2301683. 37327023 10.1002/adhm.202301683 PMC11468745

[31] Qin Y, Lv C, Zhang X, Ruan W, Xu X, Chen C, et al. Neuraminidase1 inhibitor protects against doxorubicin-induced cardiotoxicity via suppressing Drp1-dependent mitophagy. Front Cell Dev Biol. 2021;9:802502. 34977042 10.3389/fcell.2021.802502 PMC8719652

[32] Li J, Cai Z, Vaites LP, Shen N, Mitchell DC, Huttlin EL, et al. Proteome-wide mapping of short-lived proteins in human cells. Mol Cell. 2021;81:4722–35.e5. 34626566 10.1016/j.molcel.2021.09.015 PMC8892350

[33] Han YH, Moon HJ, You BR, Park WH. The effect of MG132, a proteasome inhibitor on HeLa cells in relation to cell growth, reactive oxygen species and GSH. Oncol Rep. 2009;22:215–21. 19513526

[34] Sharma M, Bhatt LK. Emerging therapeutic targets for heart failure. Curr Cardiol Rep. 2022;24:1737–54. 36194359 10.1007/s11886-022-01789-z

[35] Gong GQ, Bilanges B, Allsop B, Masson GR, Roberton V, Askwith T, et al. A small-molecule PI3Kα activator for cardioprotection and neuroregeneration. Nature. 2023;618:159–68. 37225977 10.1038/s41586-023-05972-2 PMC7614683

[36] Zheng Z, Nan B, Liu C, Tang D, Li W, Zhao L, et al. Inhibition of histone methyltransferase PRMT5 attenuates cisplatin-induced hearing loss through the PI3K/Akt-mediated mitochondrial apoptotic pathway. J Pharm Anal. 2023;13:590–602. 37440906 10.1016/j.jpha.2023.04.014 PMC10334280

[37] Qu L, Liu Y, Deng J, Ma X, Fan D. Ginsenoside Rk3 is a novel PI3K/AKT-targeting therapeutics agent that regulates autophagy and apoptosis in hepatocellular carcinoma. J Pharm Anal. 2023;13:463–82. 37305788 10.1016/j.jpha.2023.03.006 PMC10257150

[38] Glaviano A, Foo ASC, Lam HY, Yap KCH, Jacot W, Jones RH, et al. PI3K/AKT/mTOR signaling transduction pathway and targeted therapies in cancer. Mol Cancer. 2023;22:138. 37596643 10.1186/s12943-023-01827-6 PMC10436543

[39] Ma Y, Schulz B, Trakooljul N, Al Ammar M, Sekora A, Sender S, et al. Inhibition of KRAS, MEK and PI3K demonstrate synergistic anti-tumor effects in pancreatic ductal adenocarcinoma cell lines. Cancers. 2022;14:4467. 36139627 10.3390/cancers14184467 PMC9497071

[40] Liu C, Zheng S, Wang Z, Wang S, Wang X, Yang L, et al. KRAS-G12D mutation drives immune suppression and the primary resistance of anti-PD-1/PD-L1 immunotherapy in non-small cell lung cancer. Cancer Commun. 2022;42:828–47. 10.1002/cac2.12327 PMC9456691 35811500

[41] Hardie DG, Ross FA, Hawley SA. AMPK: a nutrient and energy sensor that maintains energy homeostasis. Nat Rev Mol Cell Biol. 2012;13:251–62. 22436748 10.1038/nrm3311 PMC5726489

[42] Jiang P, Ren L, Zhi L, Yu Z, Lv F, Xu F, et al. Negative regulation of AMPK signaling by high glucose via E3 ubiquitin ligase MG53. Mol Cell. 2021;81:629–37.e5. 33400924 10.1016/j.molcel.2020.12.008

[43] Yogalingam G, Bonten EJ, van de Vlekkert D, Hu H, Moshiach S, Connell SA, et al. Neuraminidase 1 is a negative regulator of lysosomal exocytosis. Dev Cell. 2008;15:74–86. 18606142 10.1016/j.devcel.2008.05.005 PMC2664108

[44] Jiang K, Zhi XH, Ma YY, Zhou LQ. Long non-coding RNA TOB1-AS1 modulates cell proliferation, apoptosis, migration and invasion through miR-23a/NEU1 axis via Wnt/b-catenin pathway in gastric cancer. Eur Rev Med Pharmacol Sci. 2019;23:9890–99. 31799657 10.26355/eurrev_201911_19554

[45] Zhou X, Zhai Y, Liu C, Yang G, Guo J, Li G, et al. Sialidase NEU1 suppresses progression of human bladder cancer cells by inhibiting fibronectin-integrin α5β1 interaction and Akt signaling pathway. Cell Commun Signal. 2020;18:44. 32164705 10.1186/s12964-019-0500-x PMC7066847

[46] Ren LR, Zhang LP, Huang SY, Zhu YF, Li WJ, Fang SY, et al. Effects of sialidase NEU1 siRNA on proliferation, apoptosis, and invasion in human ovarian cancer. Mol Cell Biochem. 2016;411:213–19. 26463994 10.1007/s11010-015-2583-z

[47] Wu Z, He L, Yang L, Fang X, Peng L. Potential role of NEU1 in hepatocellular carcinoma: a study based on comprehensive bioinformatical analysis. Front Mol Biosci. 2021;8:651525. 34513919 10.3389/fmolb.2021.651525 PMC8427823

[48] Kato T, Wang Y, Yamaguchi K, Milner CM, Shineha R, Satomi S, et al. Overexpression of lysosomal-type sialidase leads to suppression of metastasis associated with reversion of malignant phenotype in murine B16 melanoma cells. Int J Cancer. 2001;92:797–804. 11351298 10.1002/ijc.1268

[49] Lv T, Lv H, Fei J, Xie Y, Lian D, Hu J, et al. p53-R273H promotes cancer cell migration via upregulation of neuraminidase-1. J Cancer. 2020;11:6874–82. 33123278 10.7150/jca.44718 PMC7591995

[50] Ding Y, Su H, Shu Y, Chen J. Post-marketing safety concerns of sotorasib: a disproportionality analysis based on FDA adverse event reporting system. Heliyon. 2024;10:30437. 10.1016/j.heliyon.2024.e30437 PMC11079084 38726179

[51] Chen M, Huang Y, Jiang S, Ke C. Safety assessment of KRAS (G12C) inhibitors based on the FDA Adverse Event Reporting System (FAERS) database: a real-world pharmacovigilance study. Lung Cancer. 2024;196:107966. 39342769 10.1016/j.lungcan.2024.107966

[52] Wu L, Xu M, Li X, Aierken D, Yu J, Qin T. A real-world pharmacovigilance study of KRAS G12C mutation inhibitors based on the food and drug administration adverse event reporting system. Front Pharmacol. 2024;15:1418469. 39263575 10.3389/fphar.2024.1418469 PMC11387170

[53] Santas E, Sandino J, Chorro FJ, Méndez J, Miñana G, Núñez E, et al. Prognostic implications of pericardial effusion in acute heart failure: does size matter? Int J Cardiol. 2015;184:259–61. 25726903 10.1016/j.ijcard.2015.02.052

[54] Wu C, Zhang Z, Zhang W, Liu X. Mitochondrial dysfunction and mitochondrial therapies in heart failure. Pharmacol Res. 2022;175:106038. 34929300 10.1016/j.phrs.2021.106038

