# Supplementary material for: Sotorasib-impaired degradation of NEU1 contributes to cardiac injury by inhibiting AKT signaling
Source: Cell Death Discov. 2025 Apr 12;11:169. doi: 10.1038/s41420-025-02431-x (PMC11993734; doi:10.1038/s41420-025-02431-x)
Supplement: Supplementary file 1 — supplementary materials-WB [file 41420_2025_2431_MOESM1_ESM.docx]

Fig.2-C and Fig. S-A


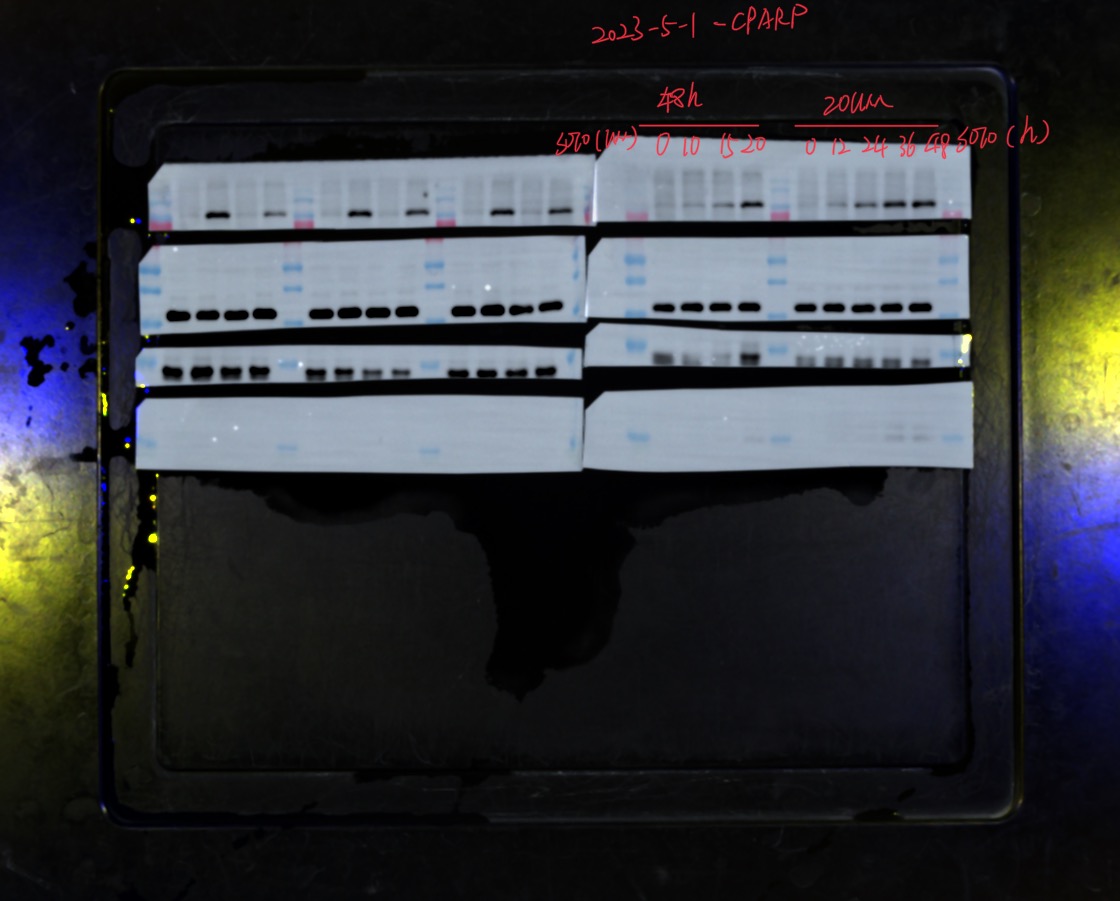

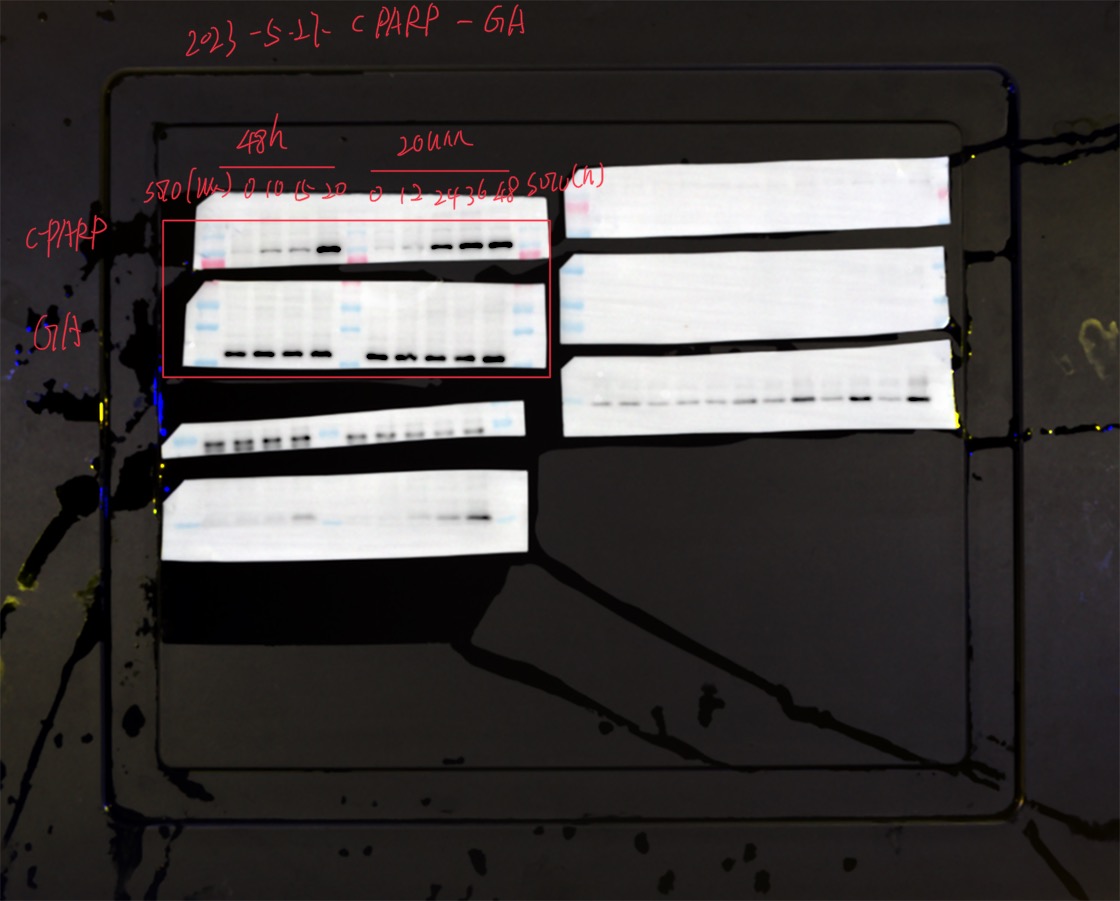

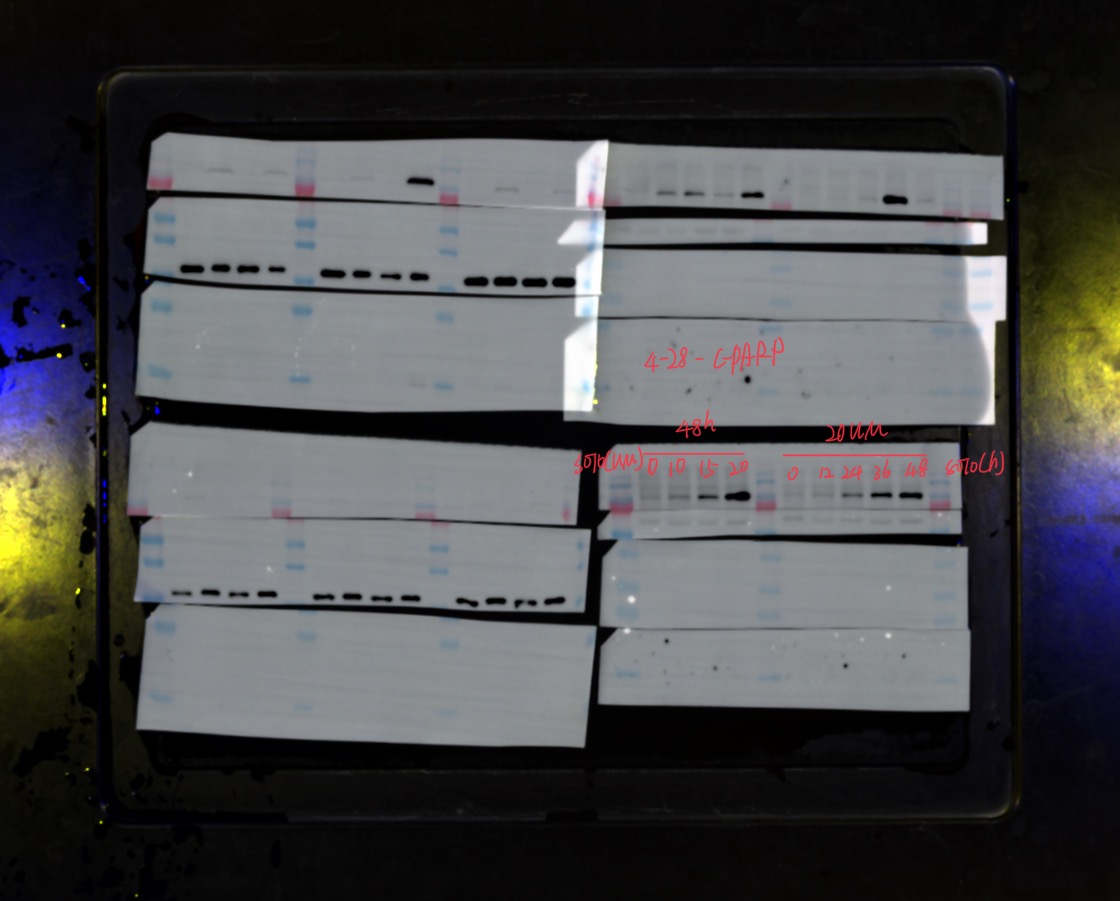


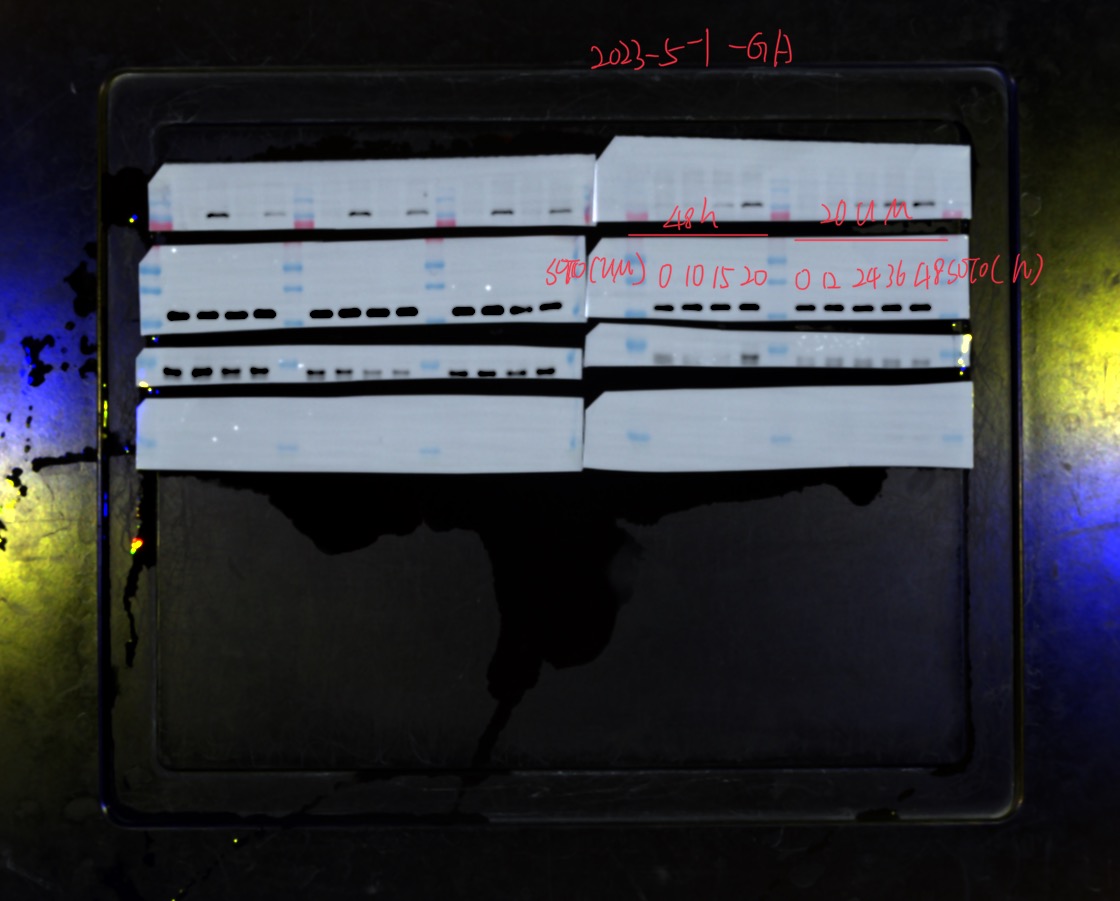

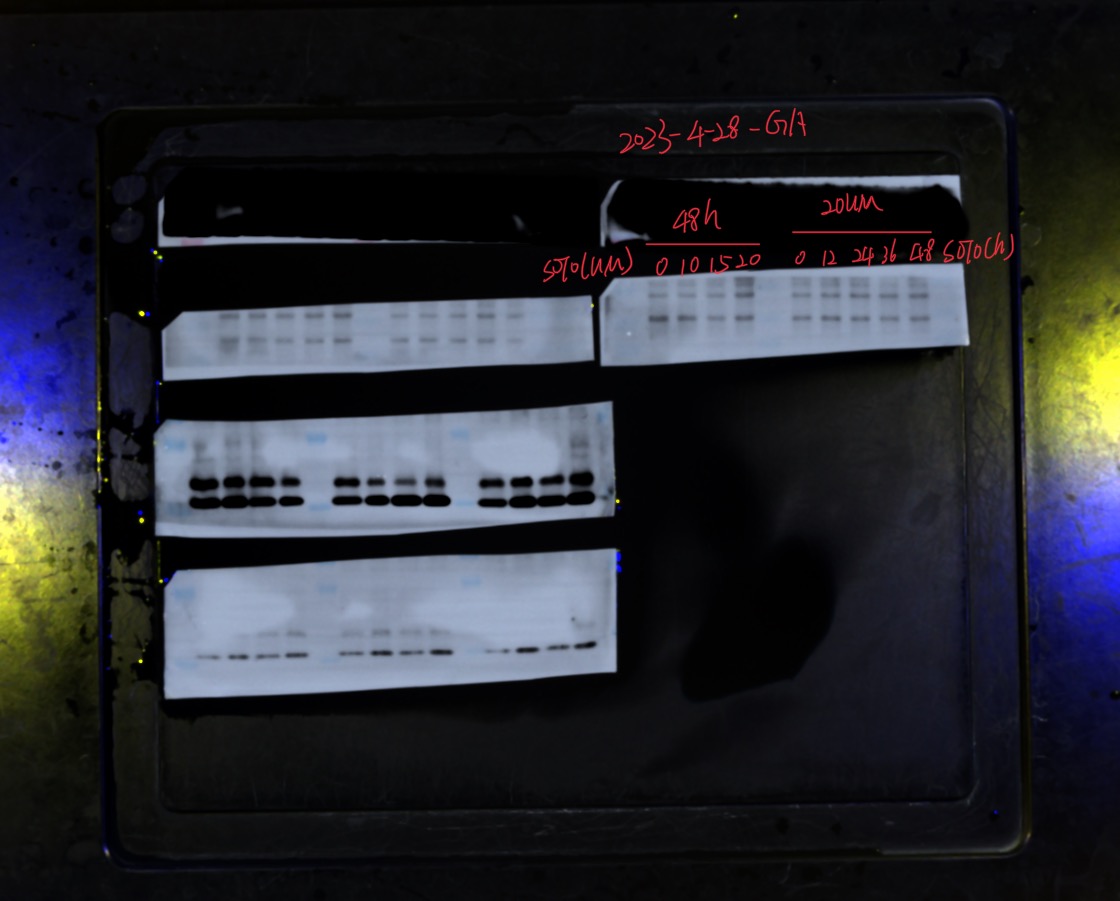


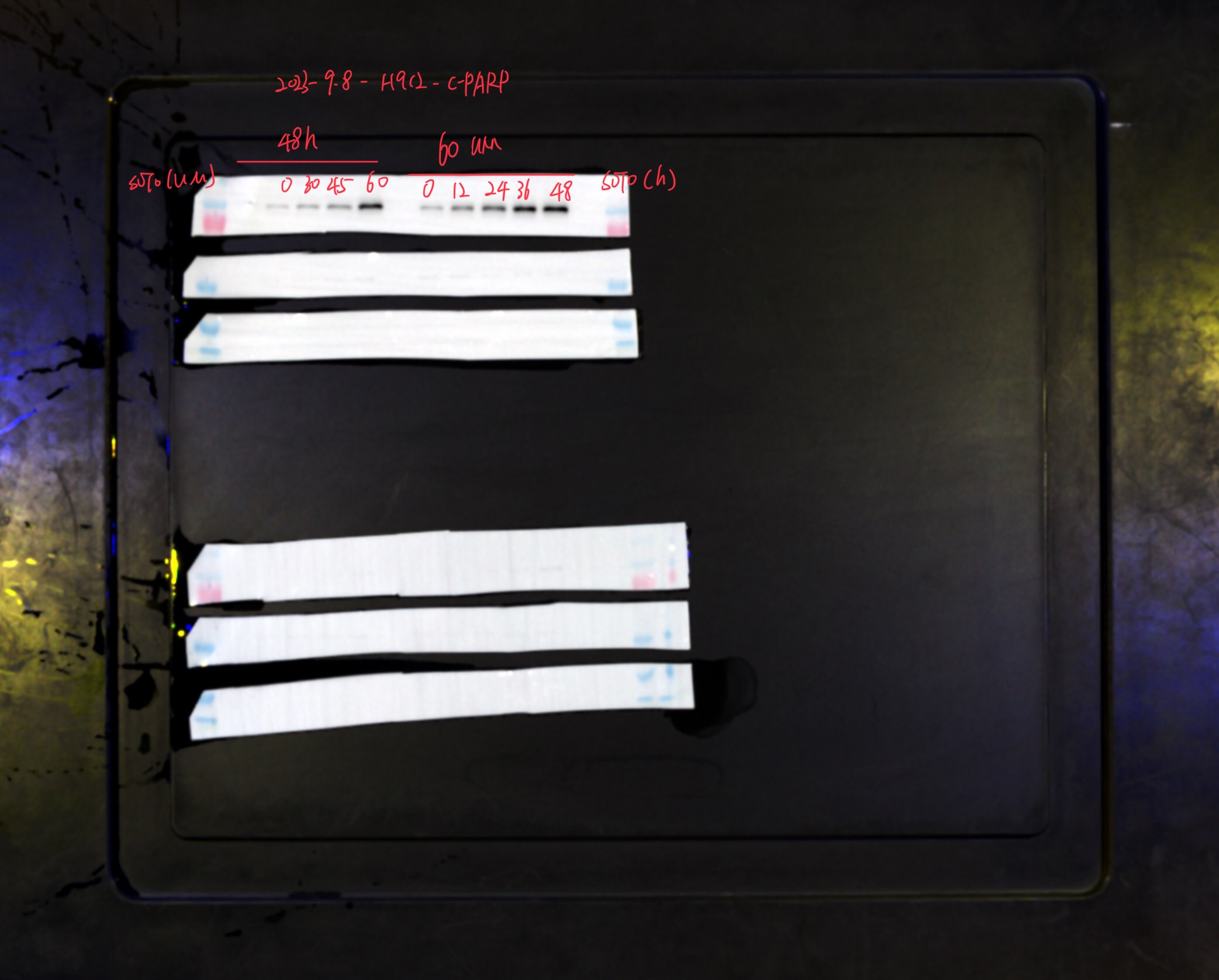

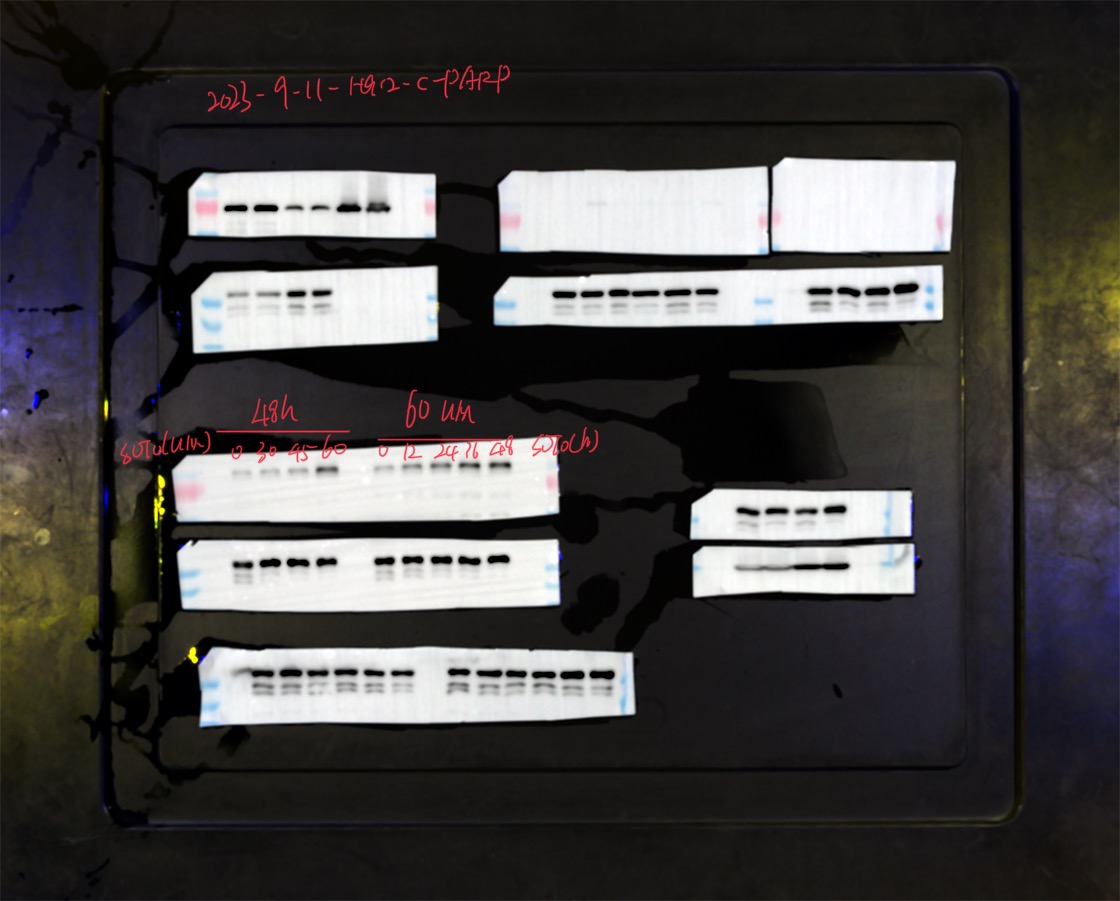

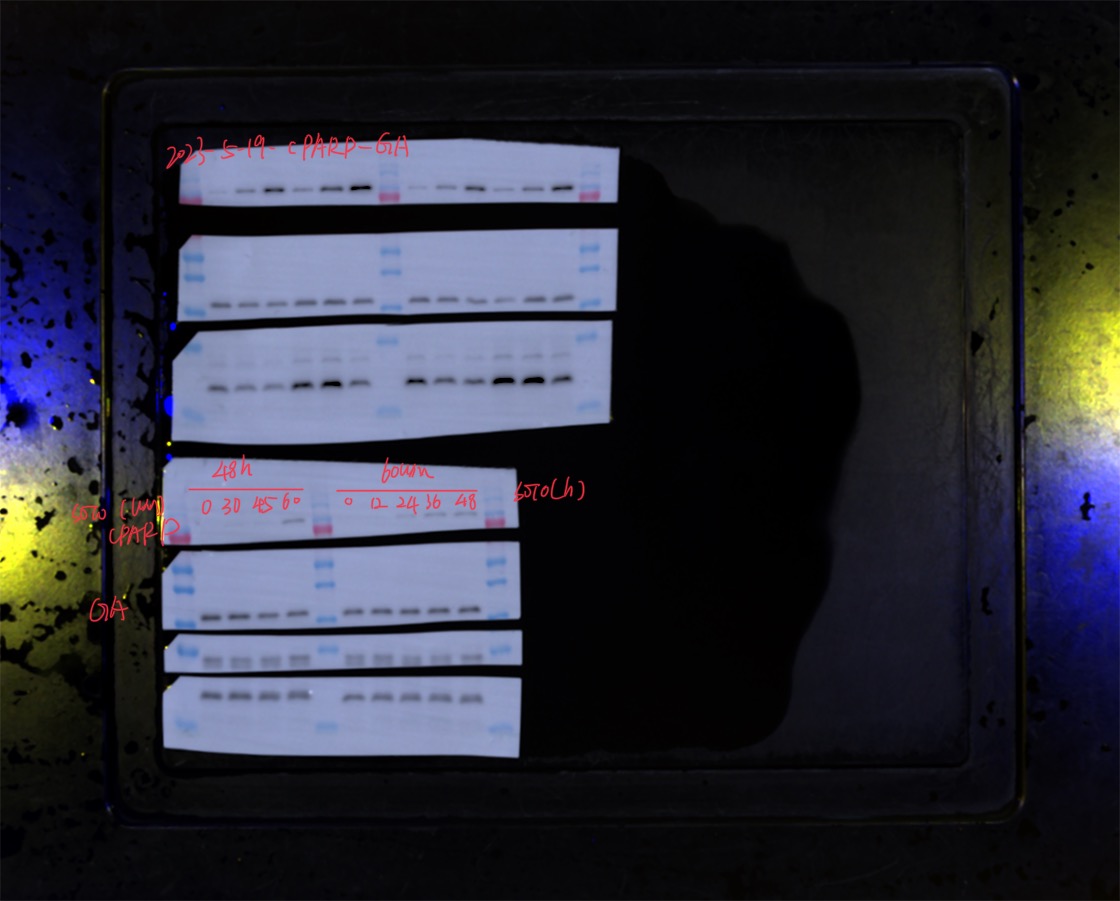


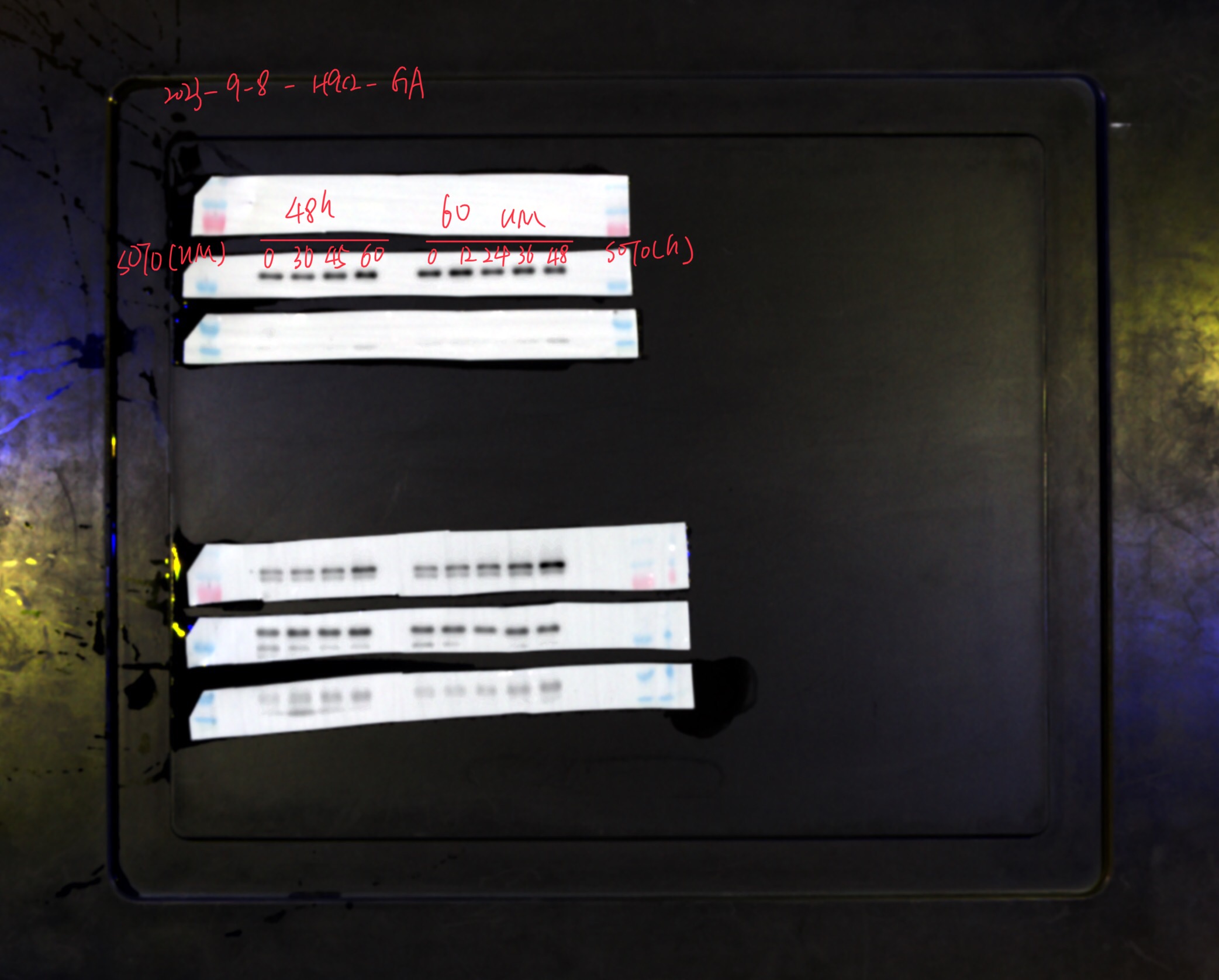

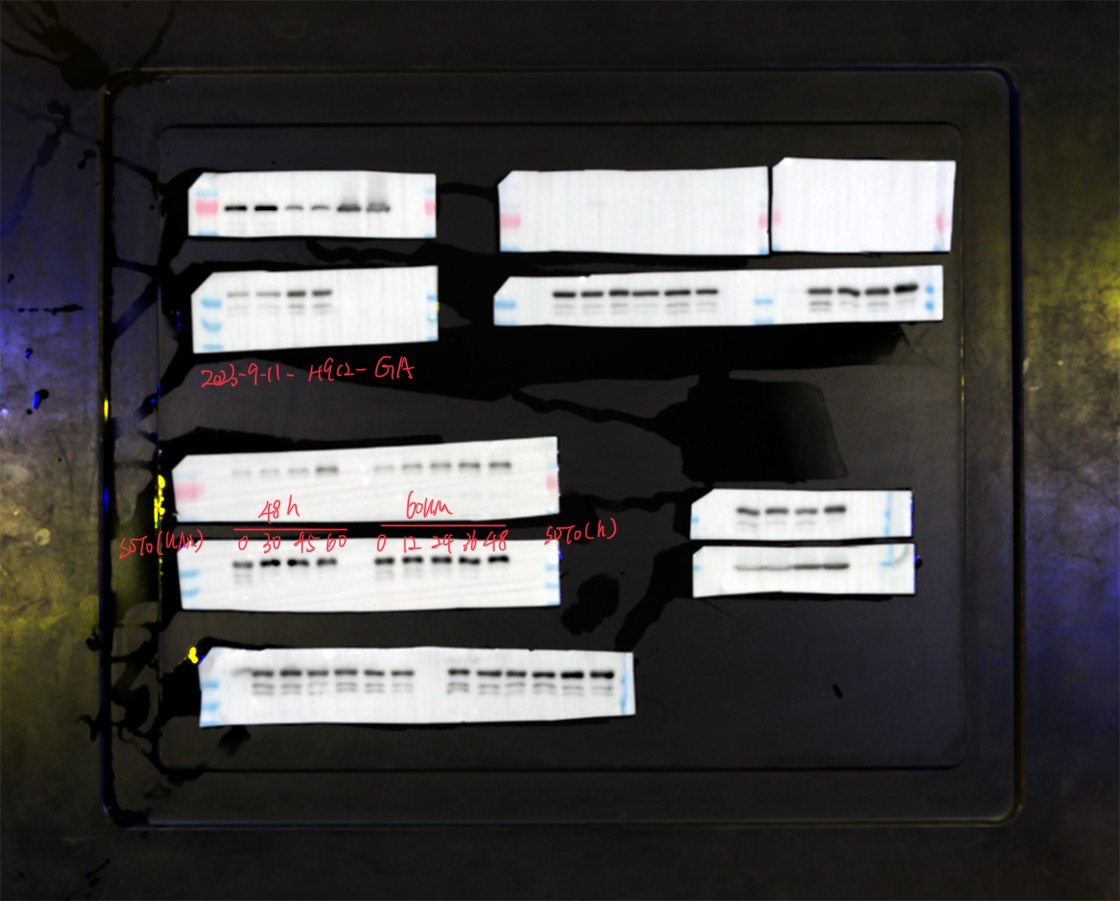


Fig. 3-B


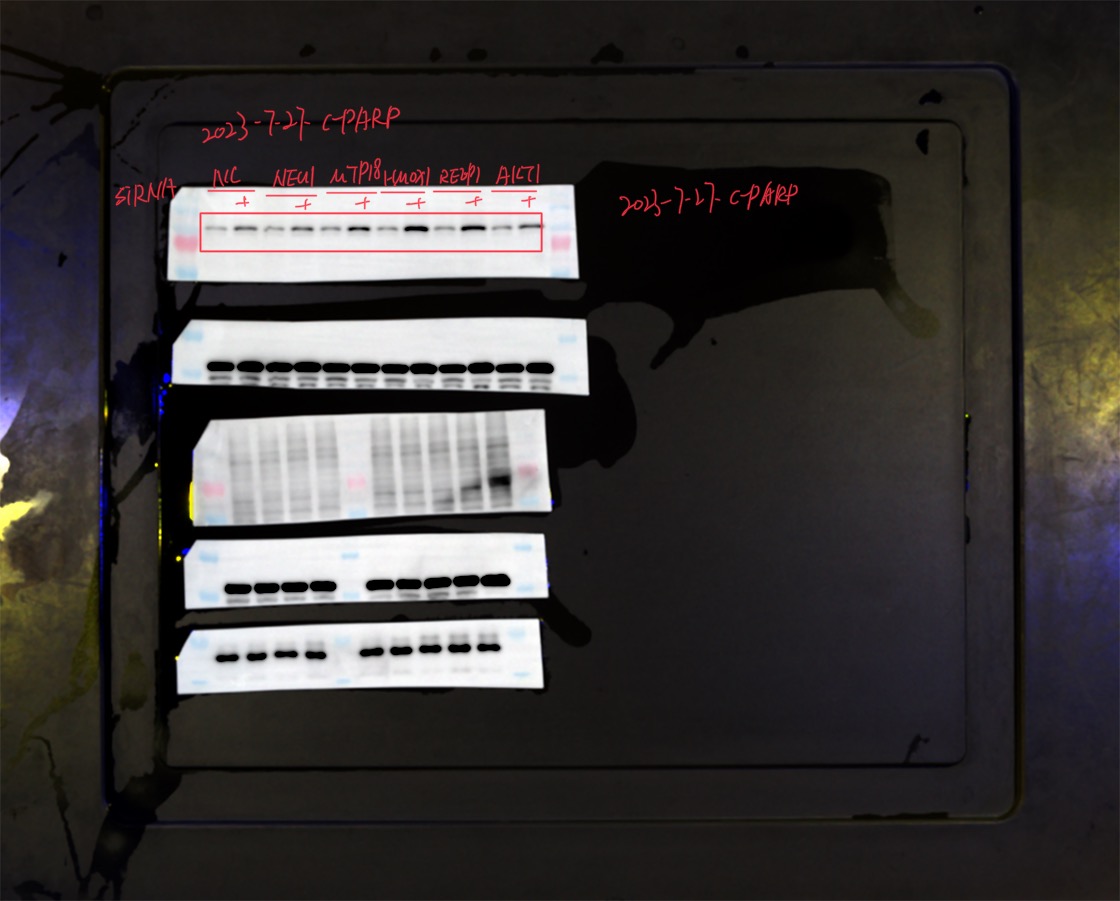

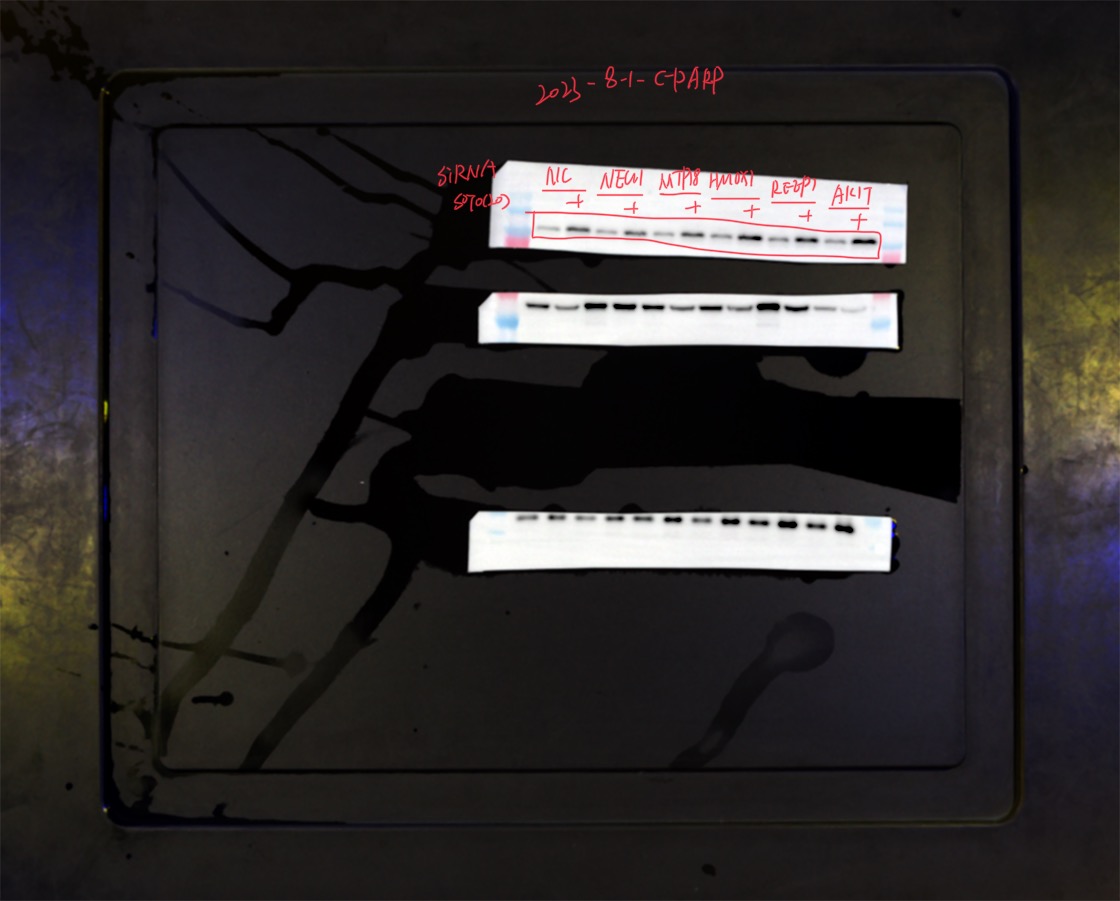

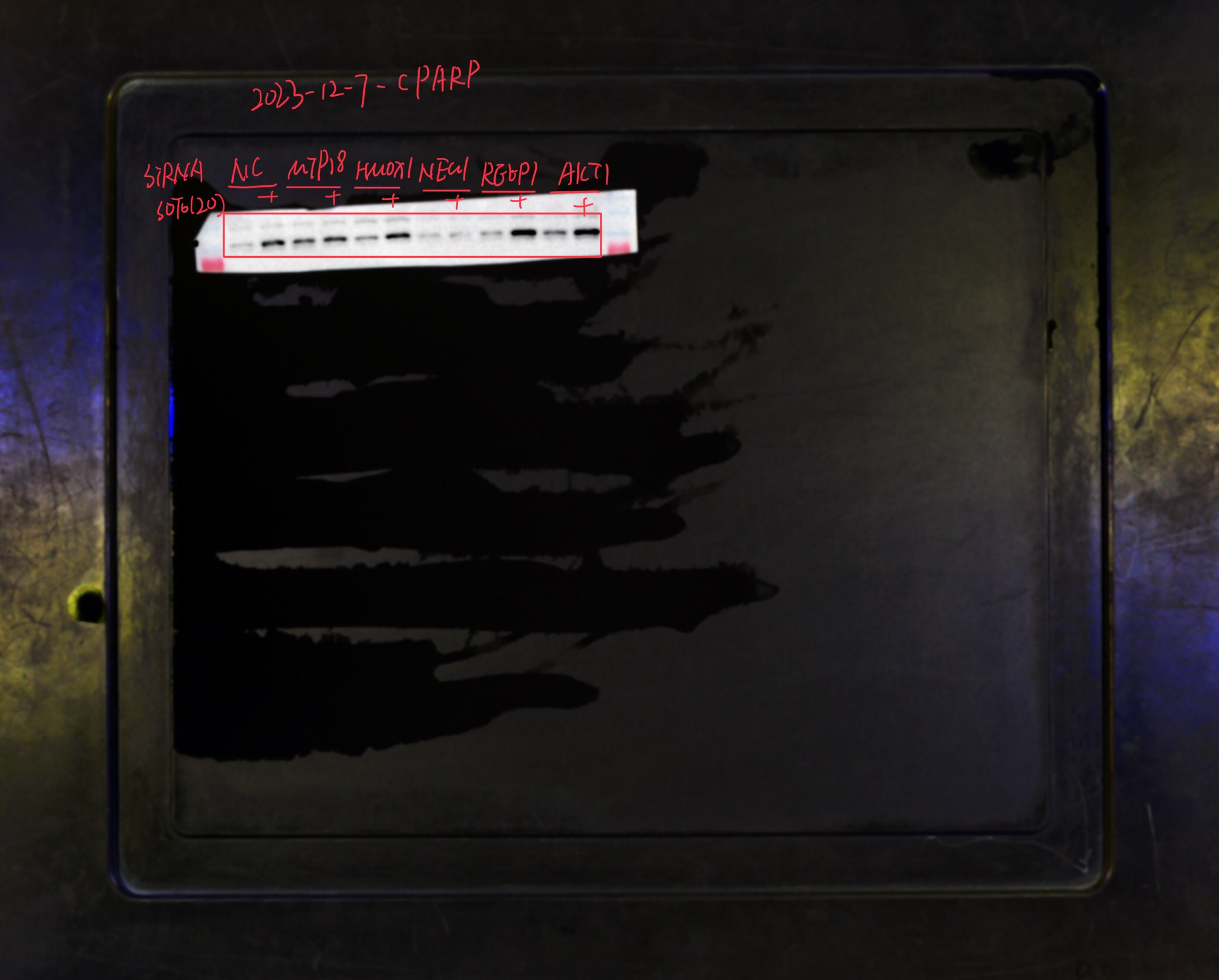


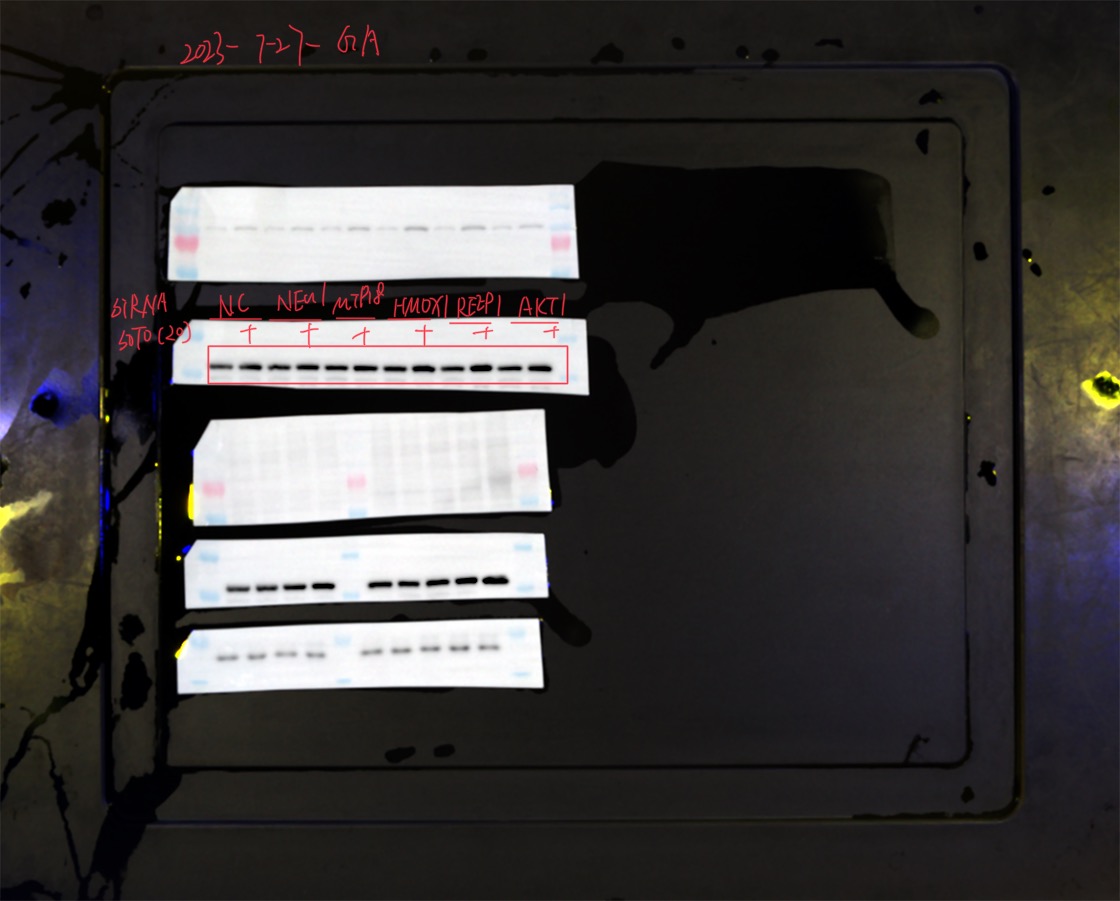

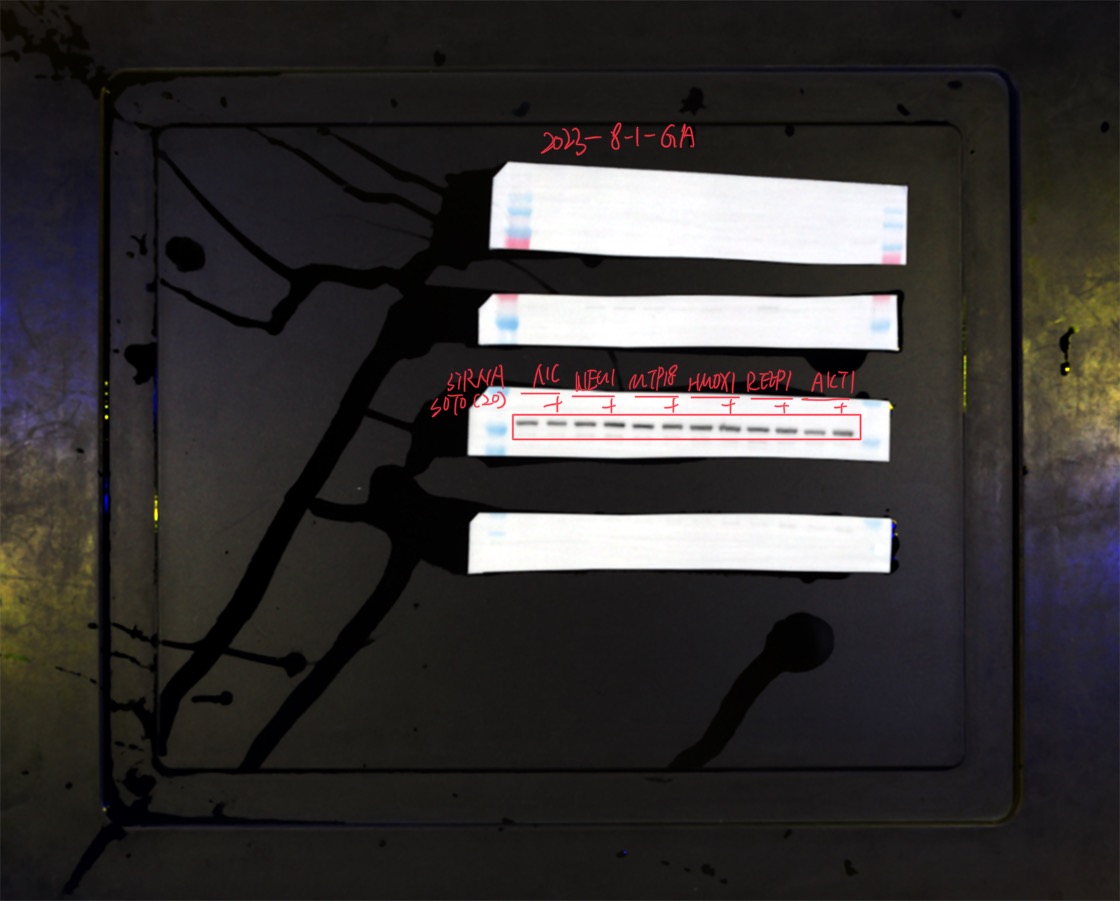

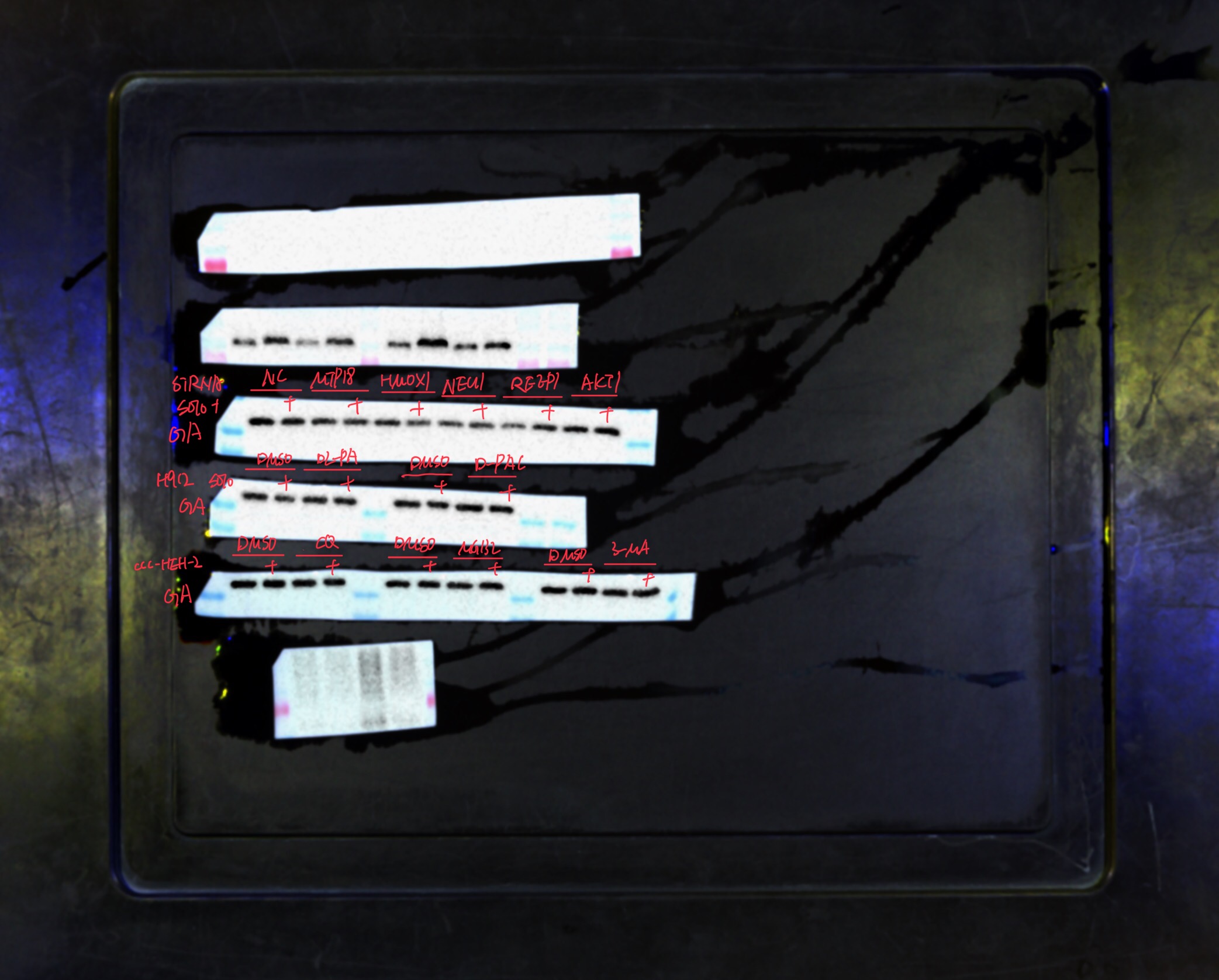


Fig. 3-C and Fig. S2-B


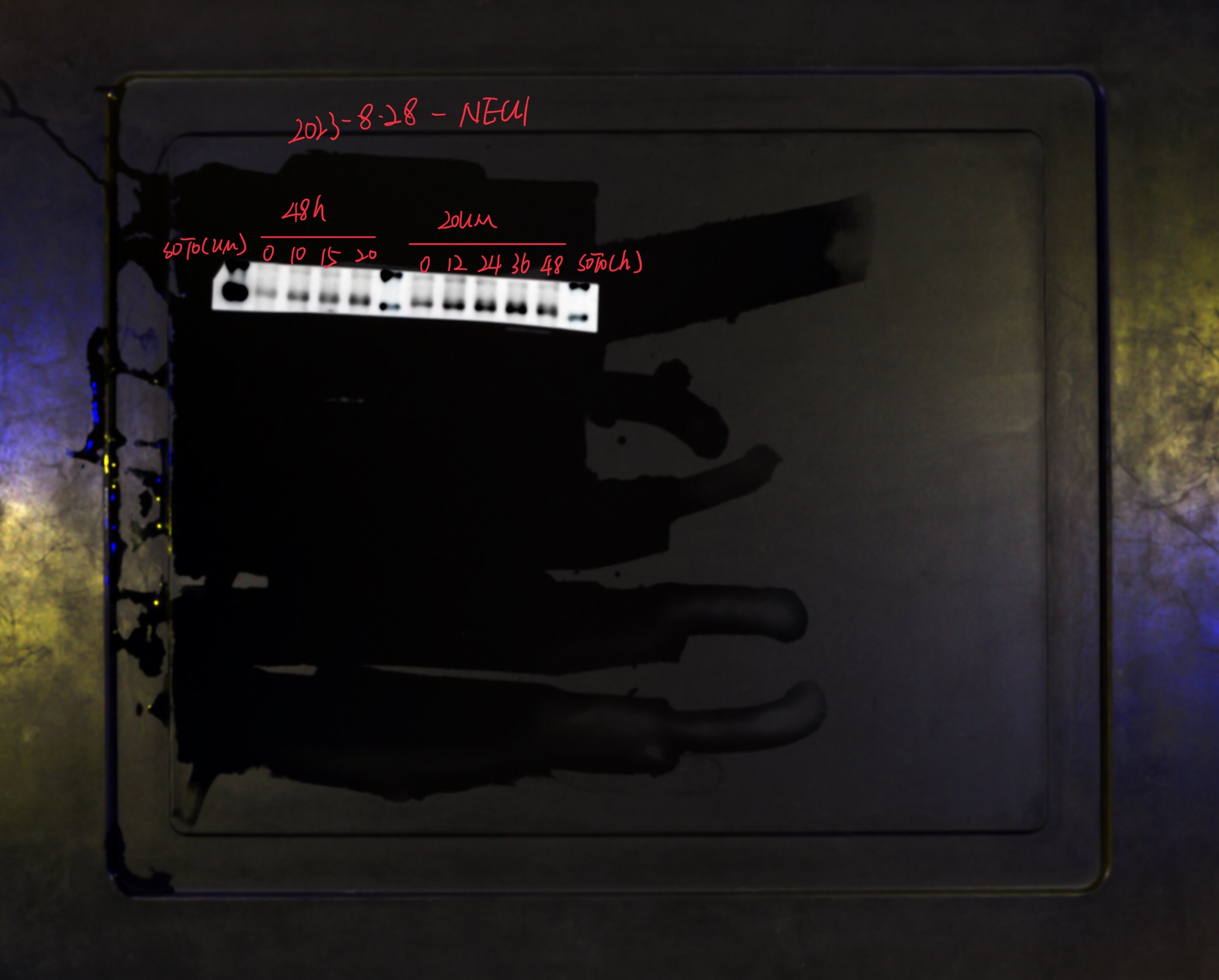


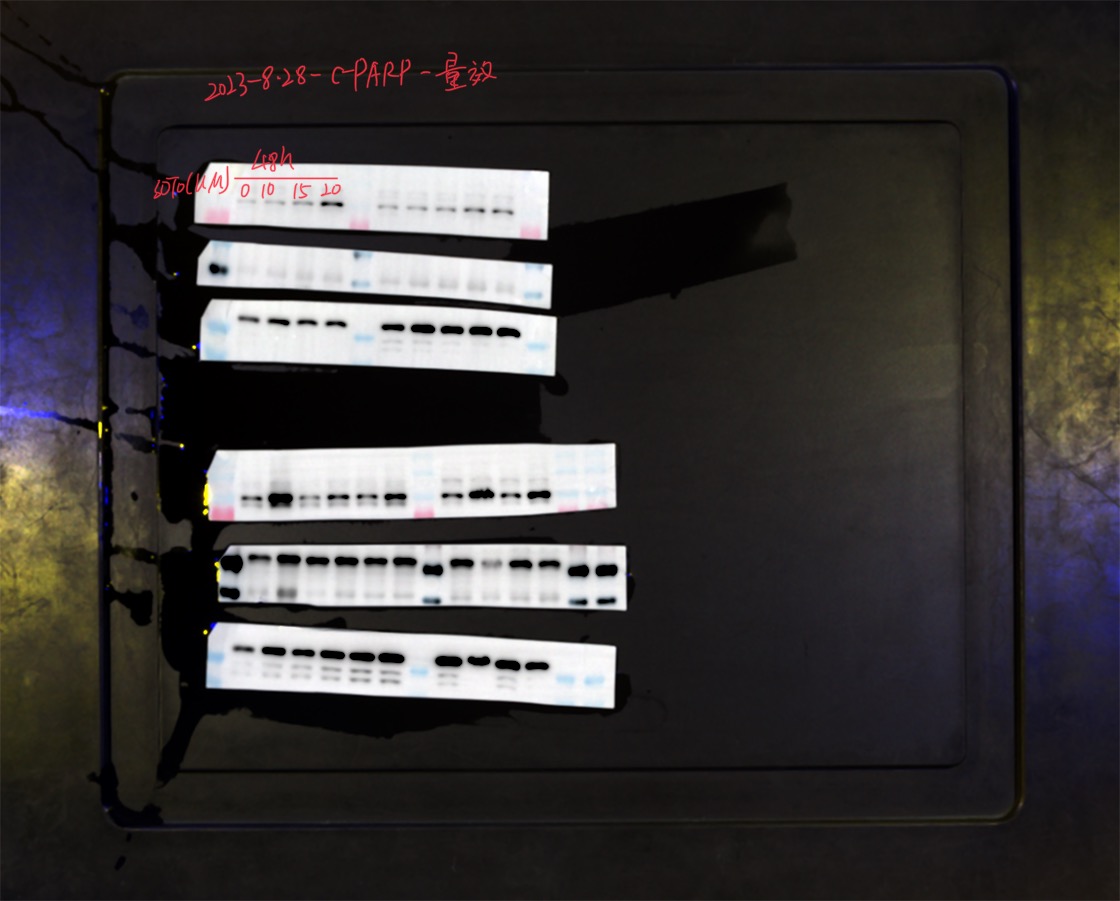

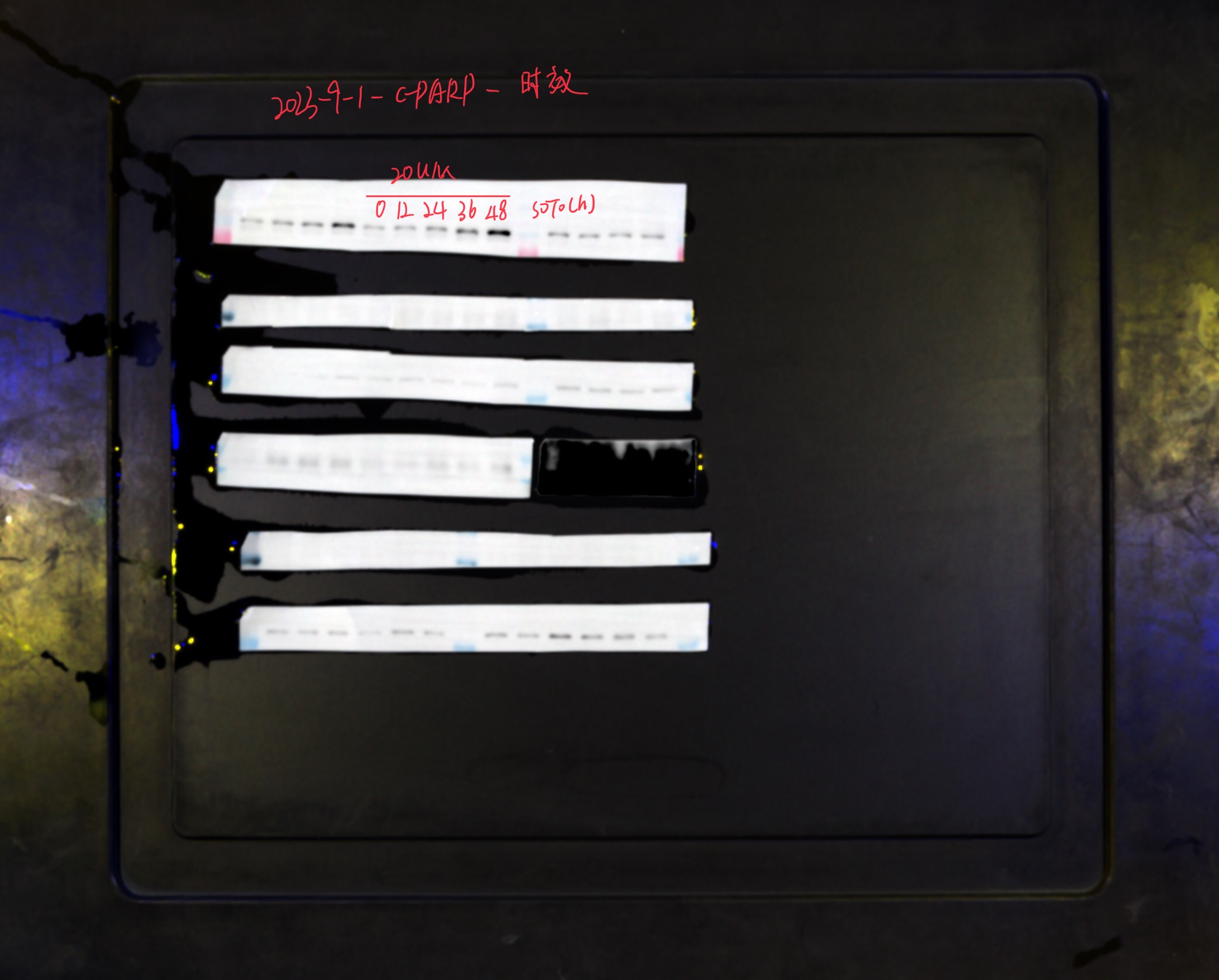

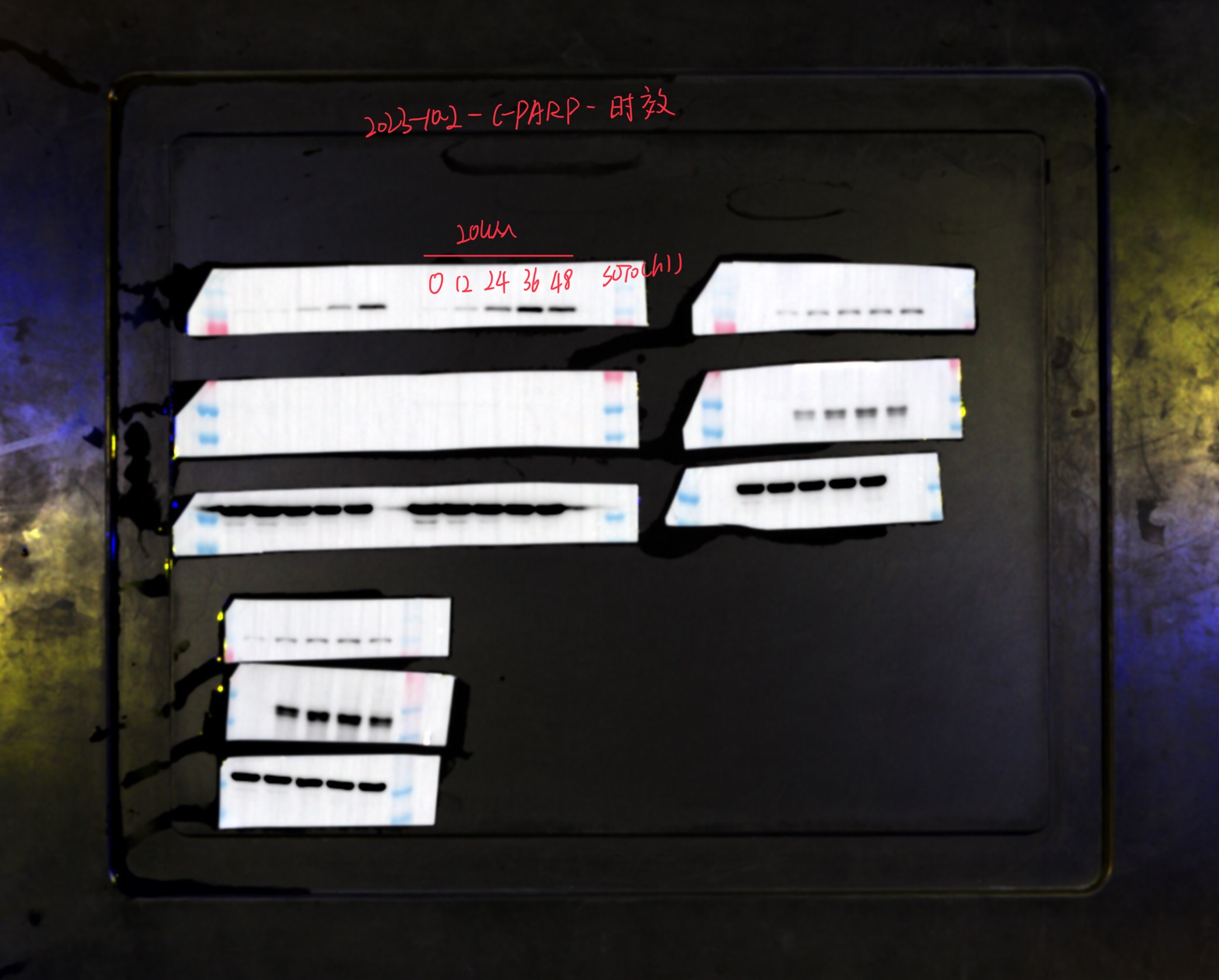


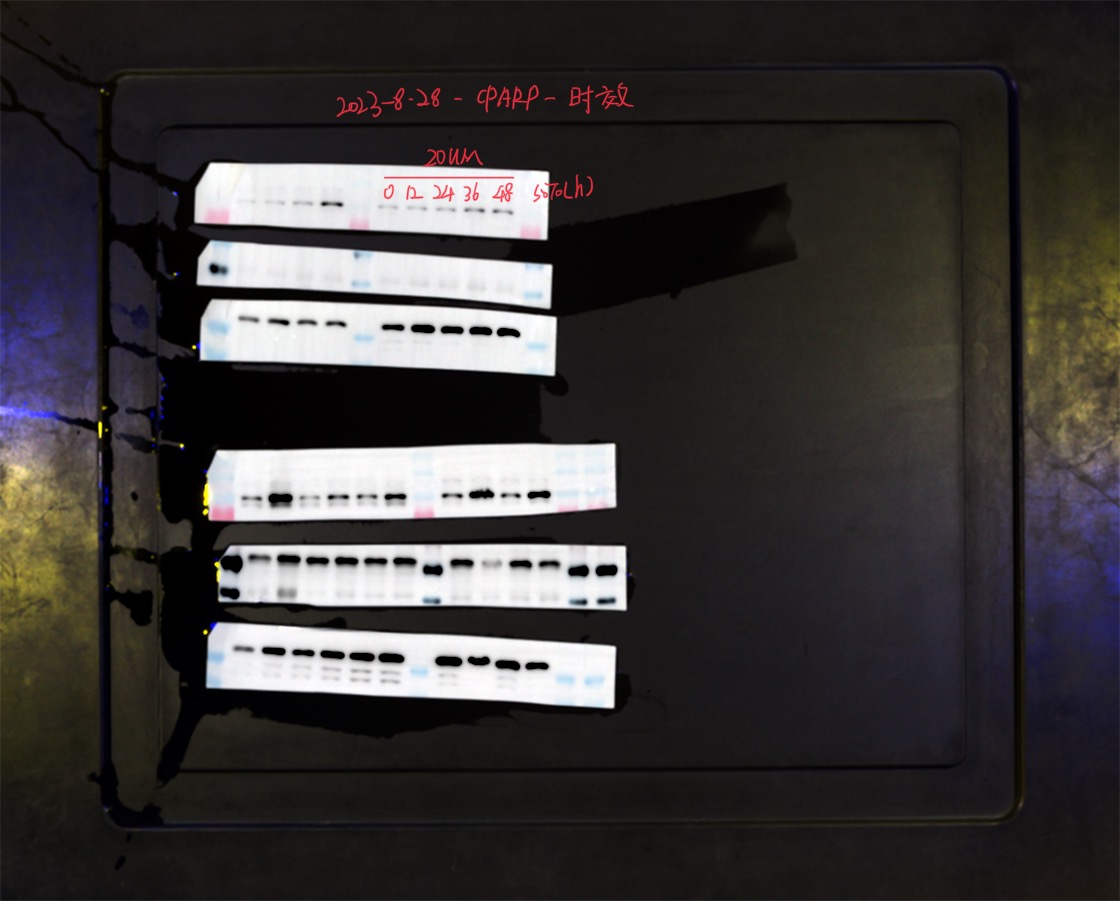

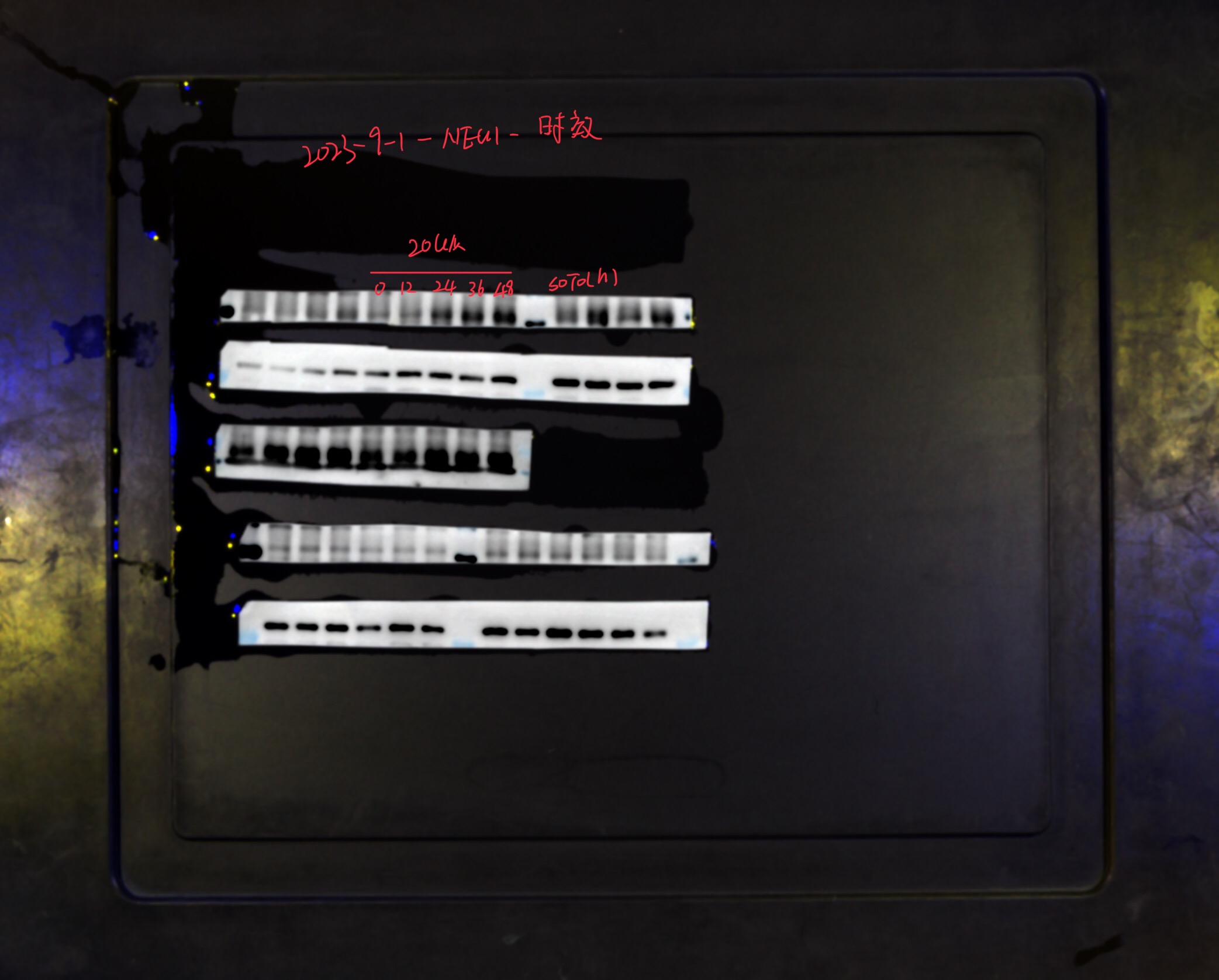

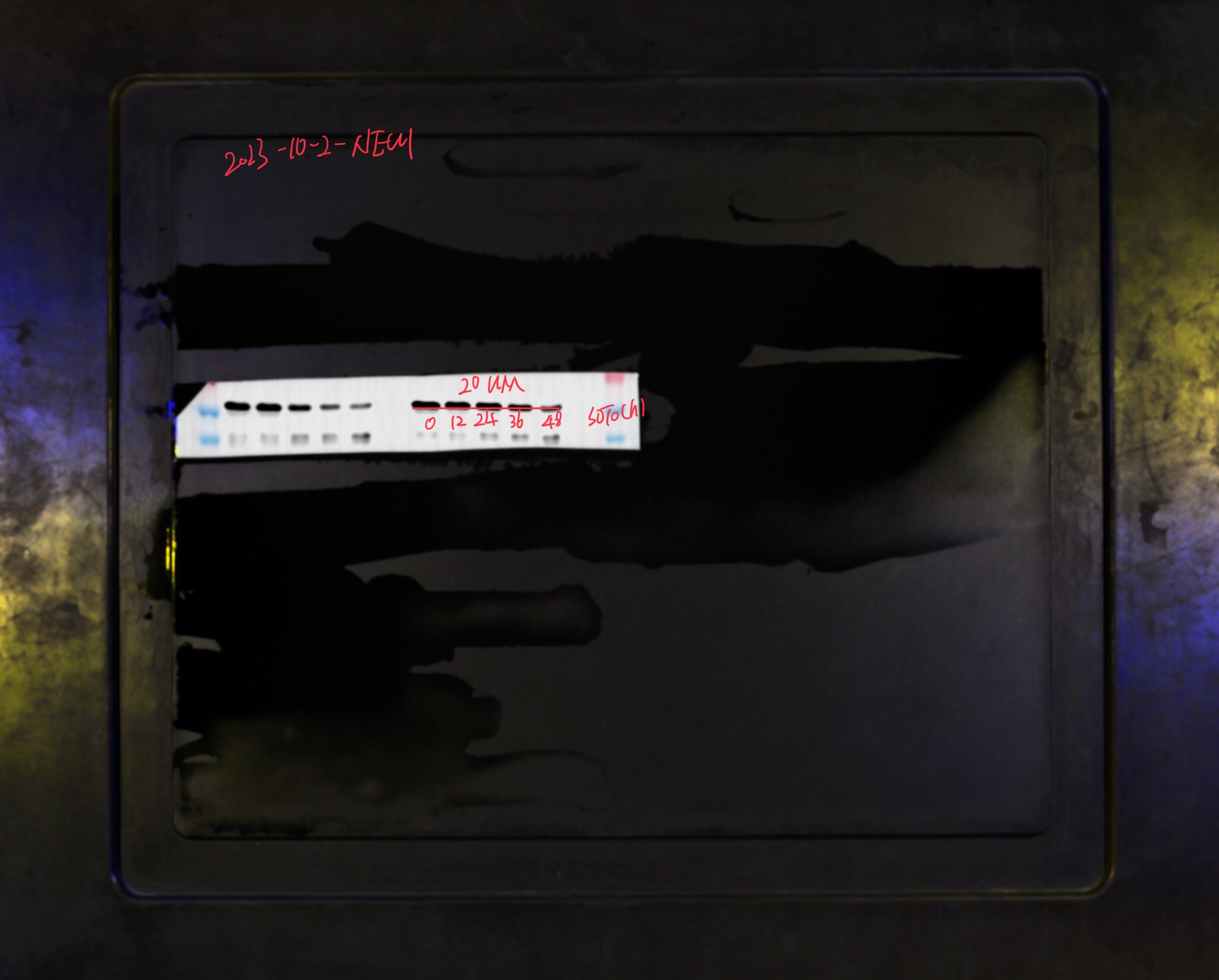


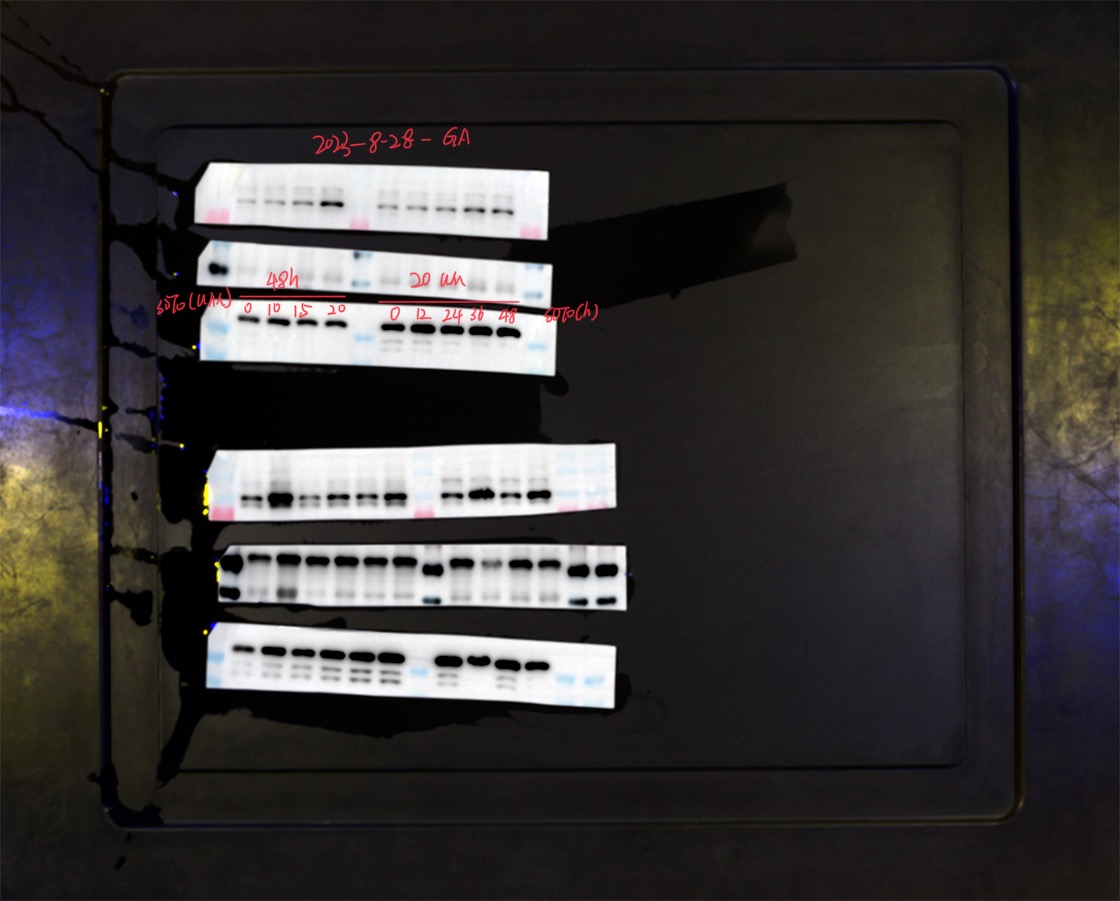

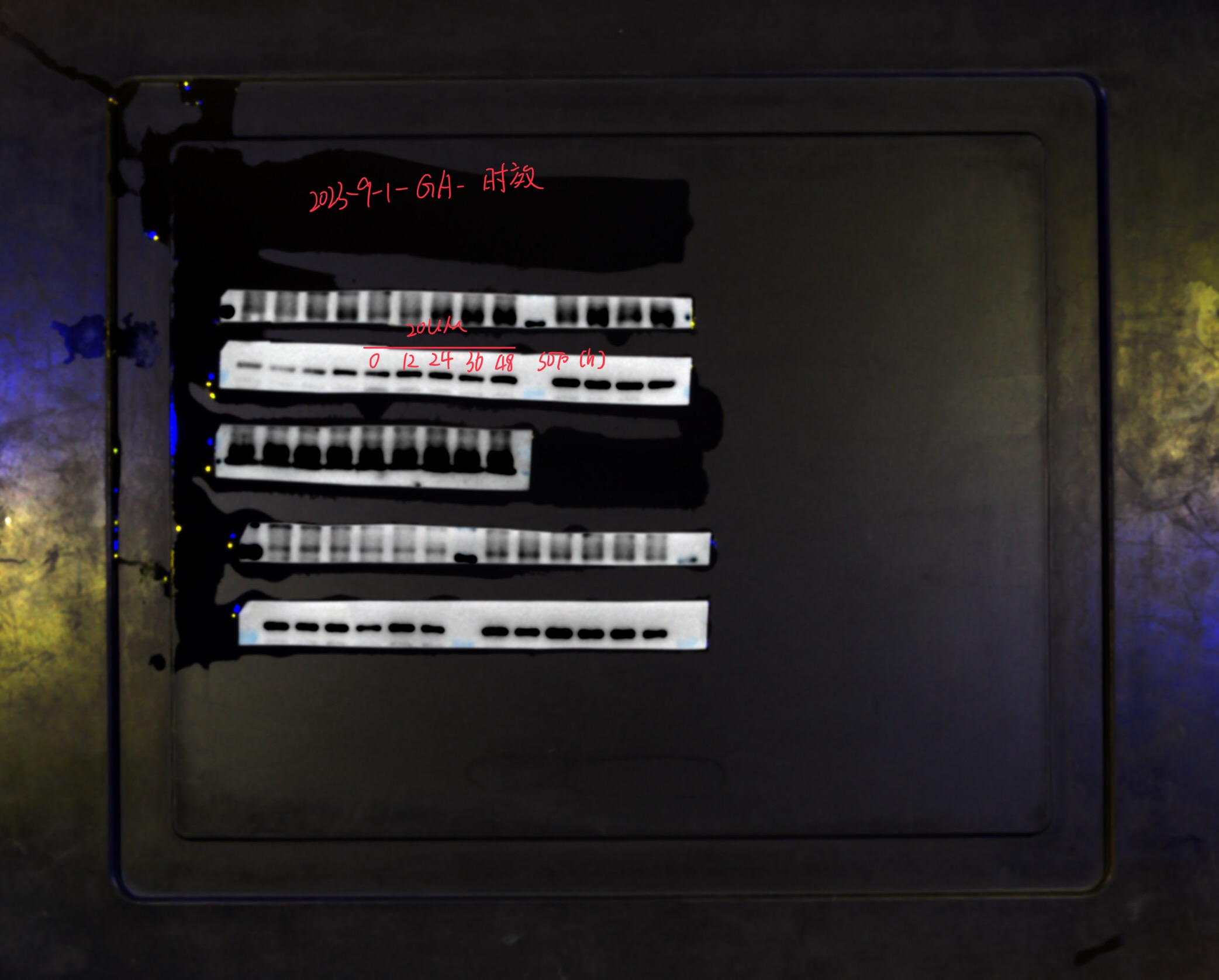

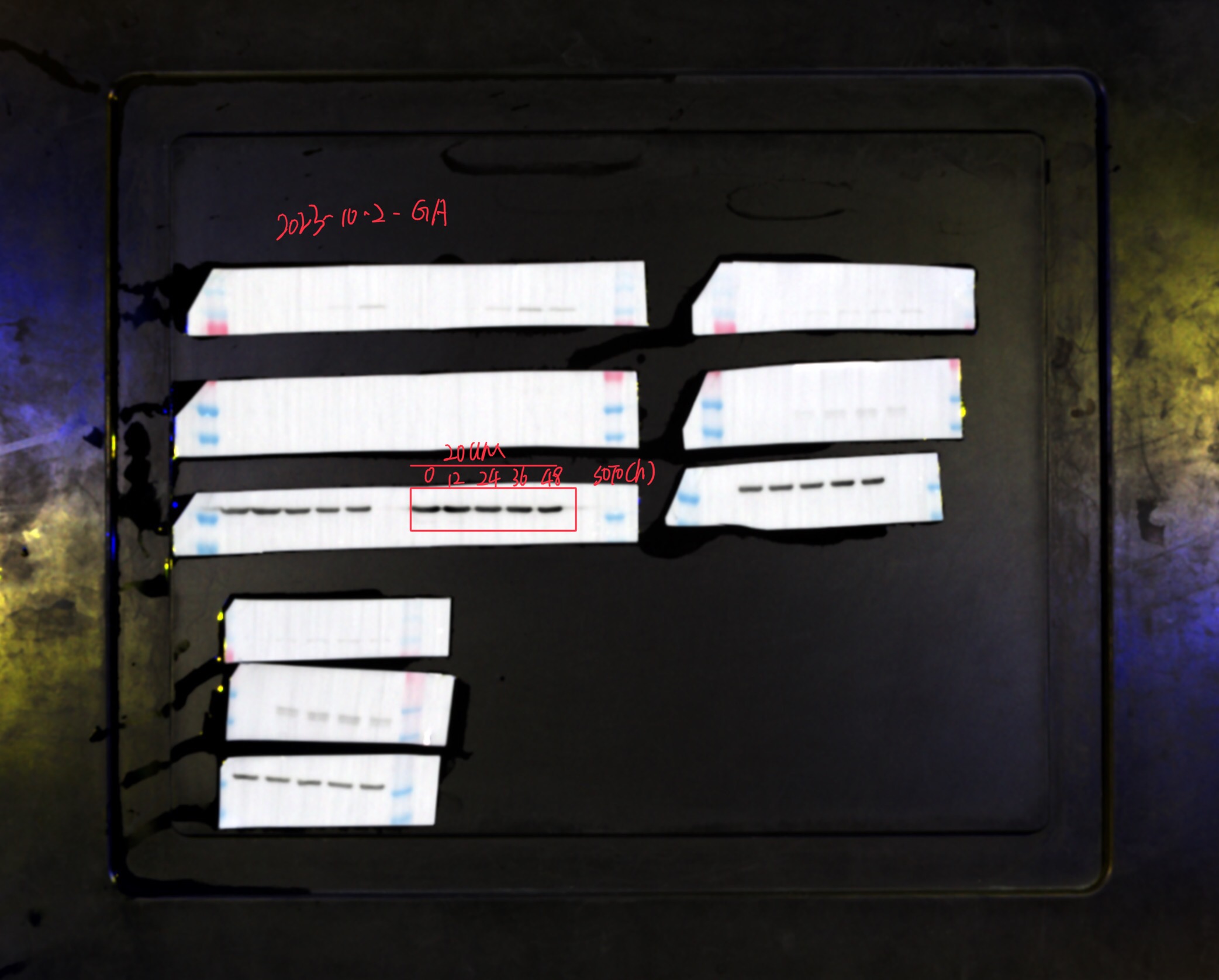


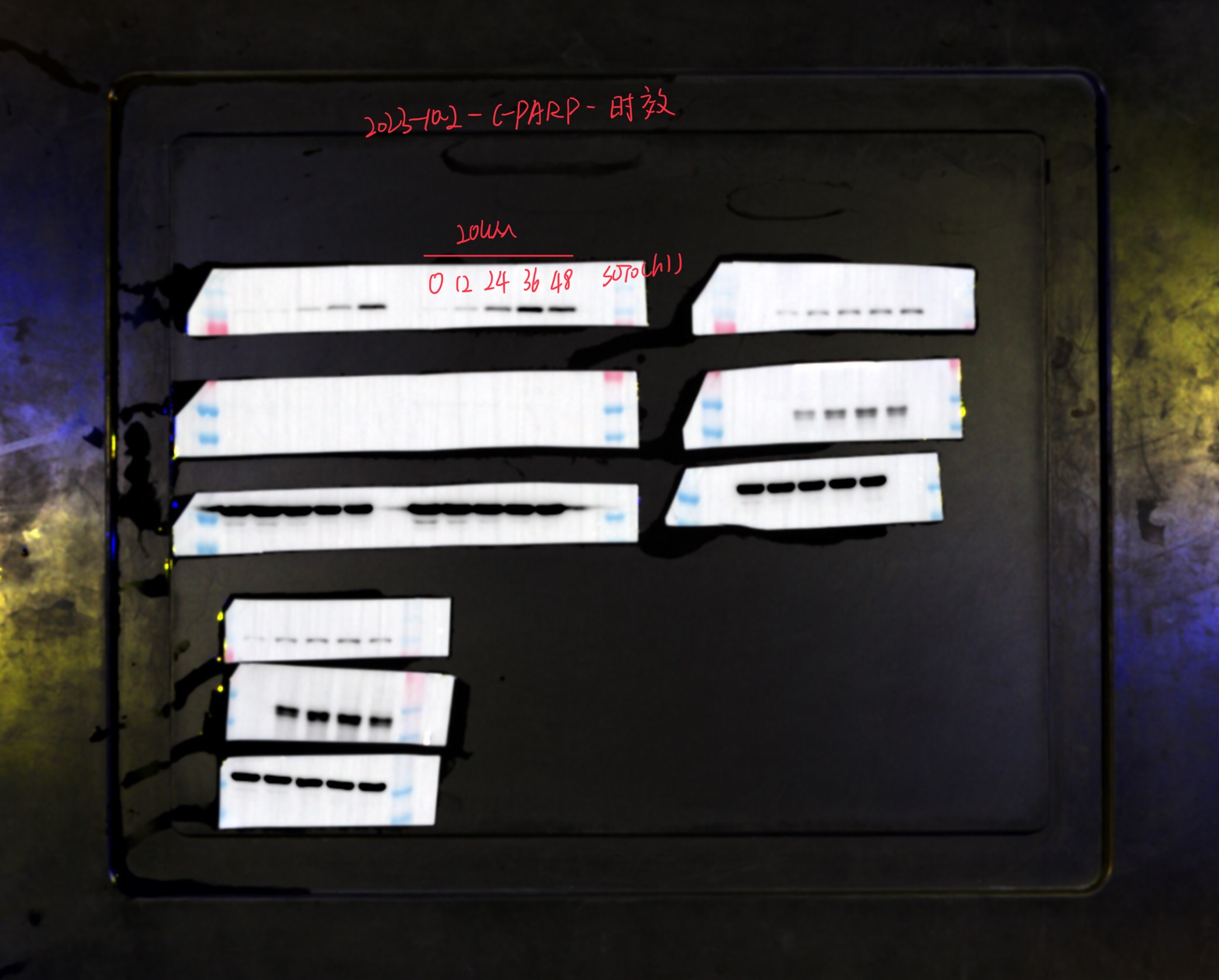

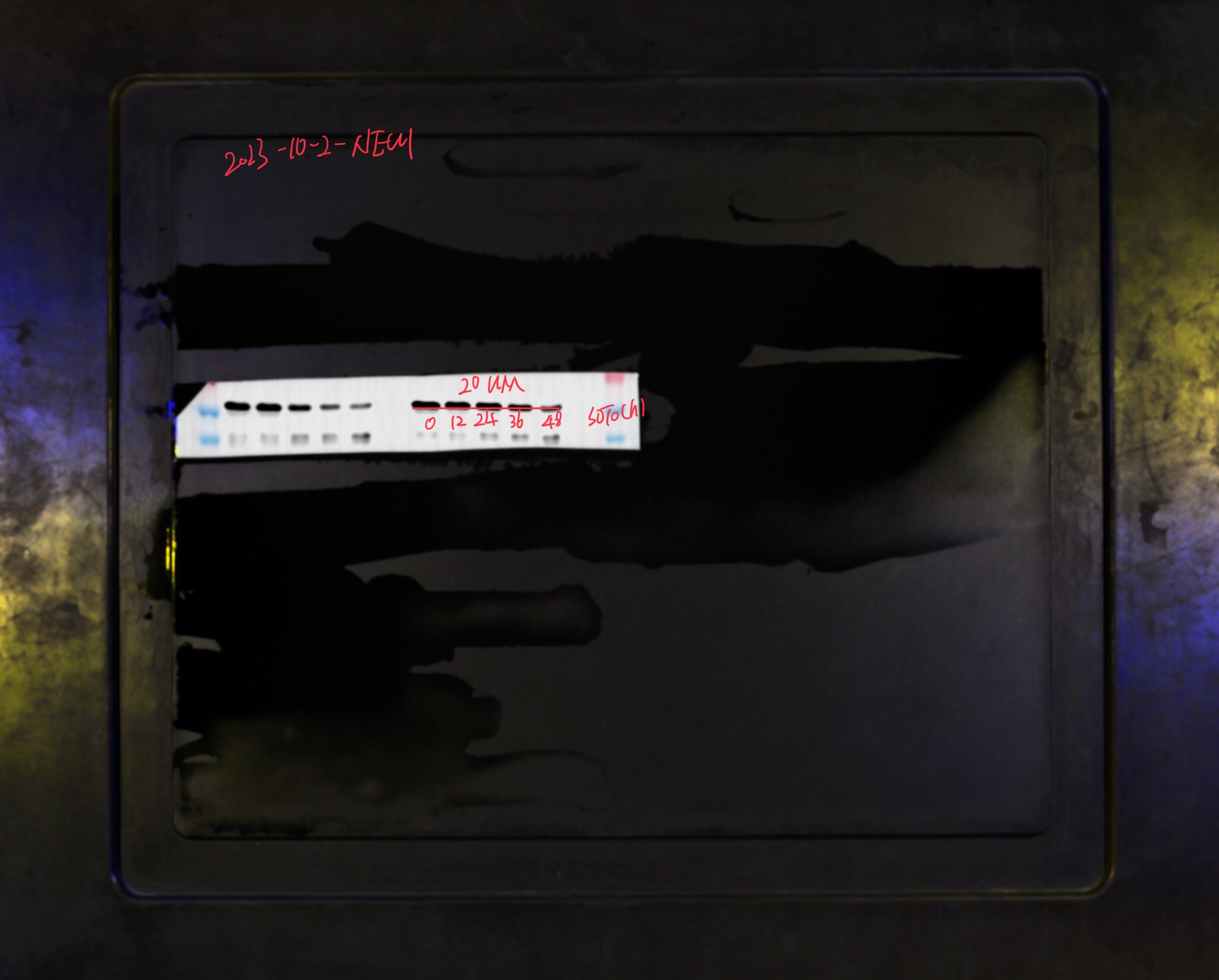

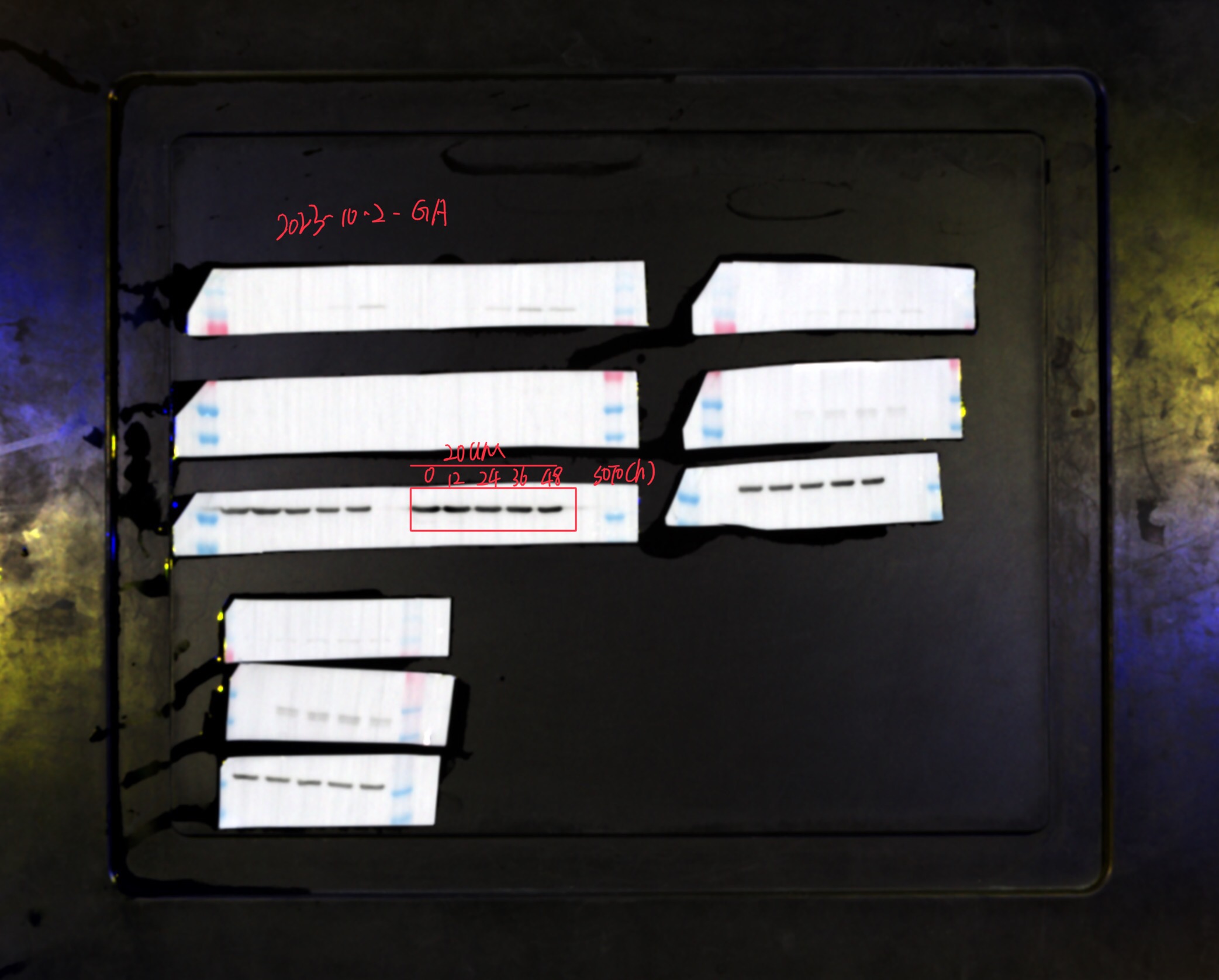


Fig. 3-E


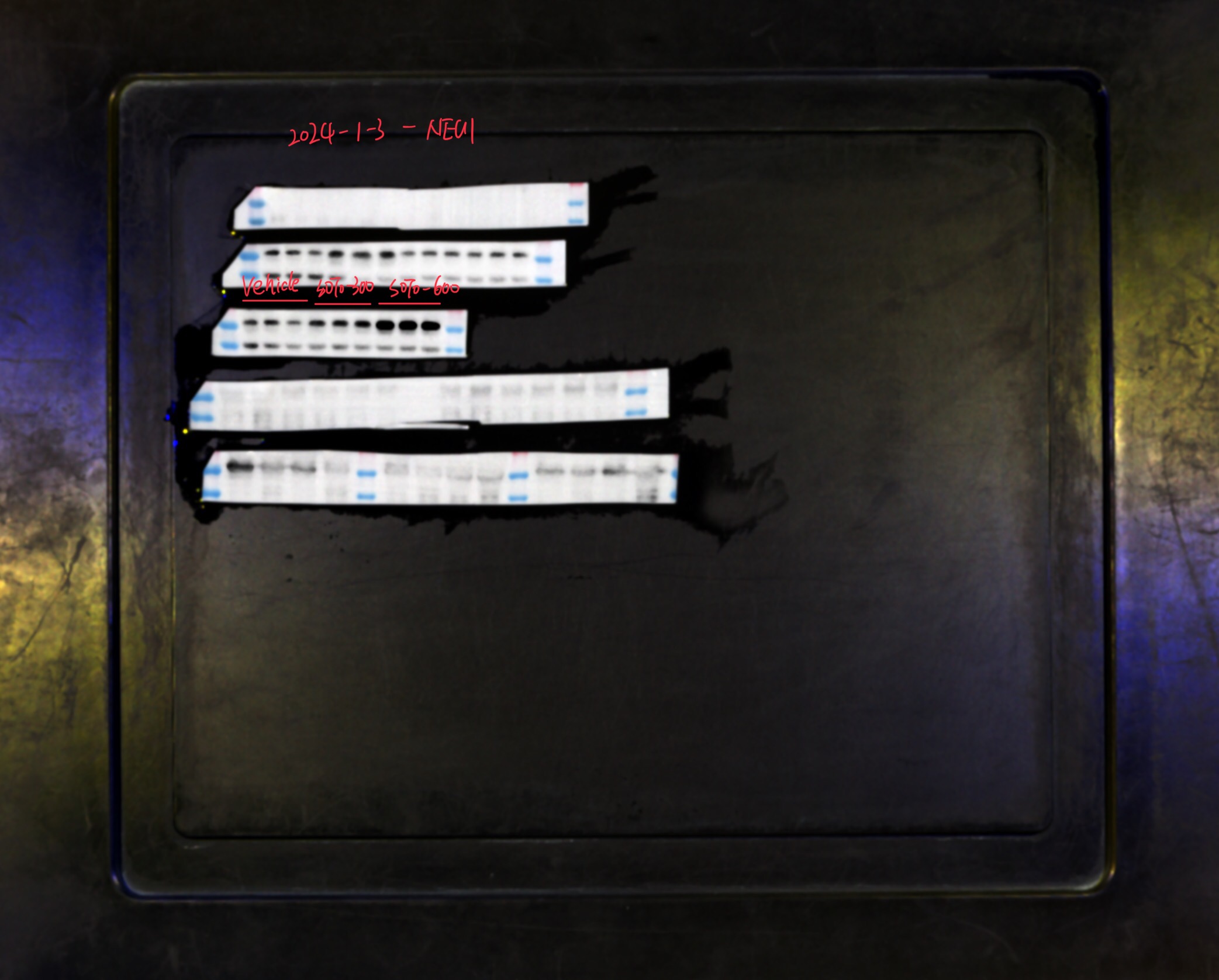

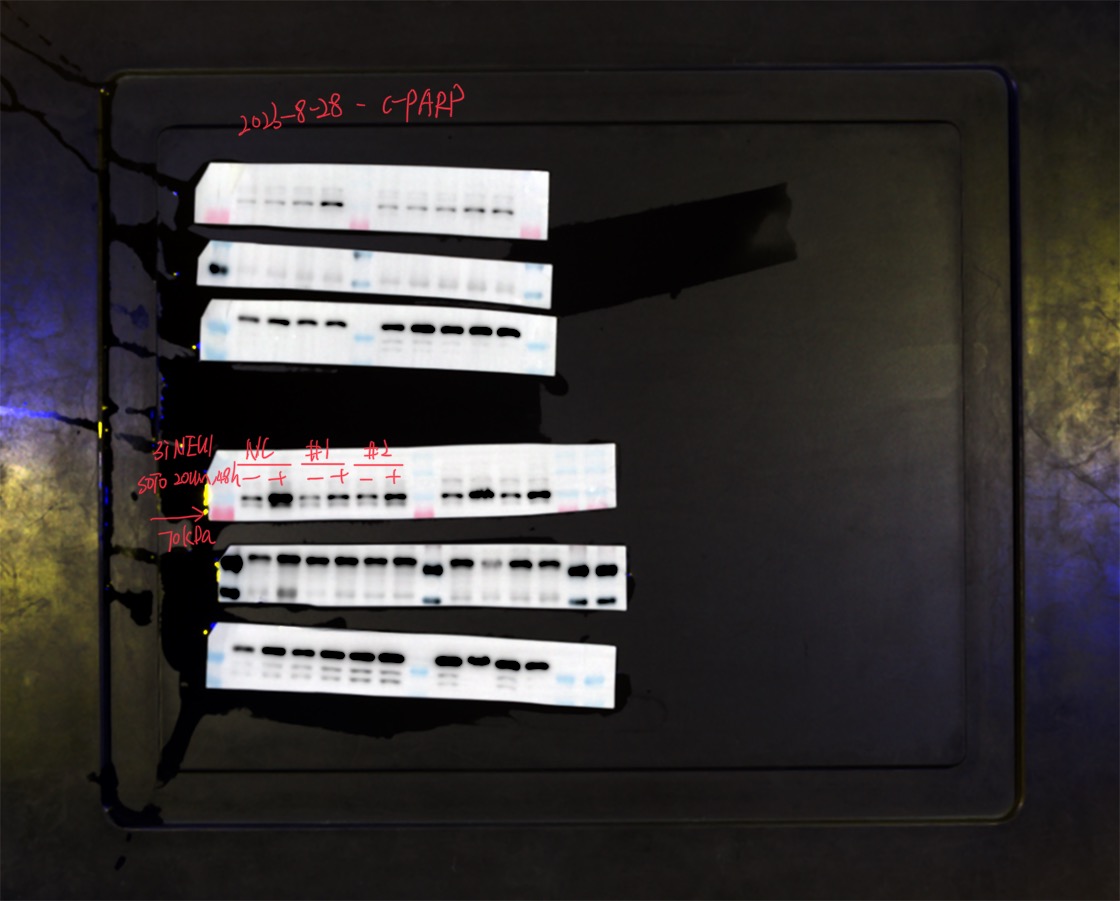


Fig. 3-F


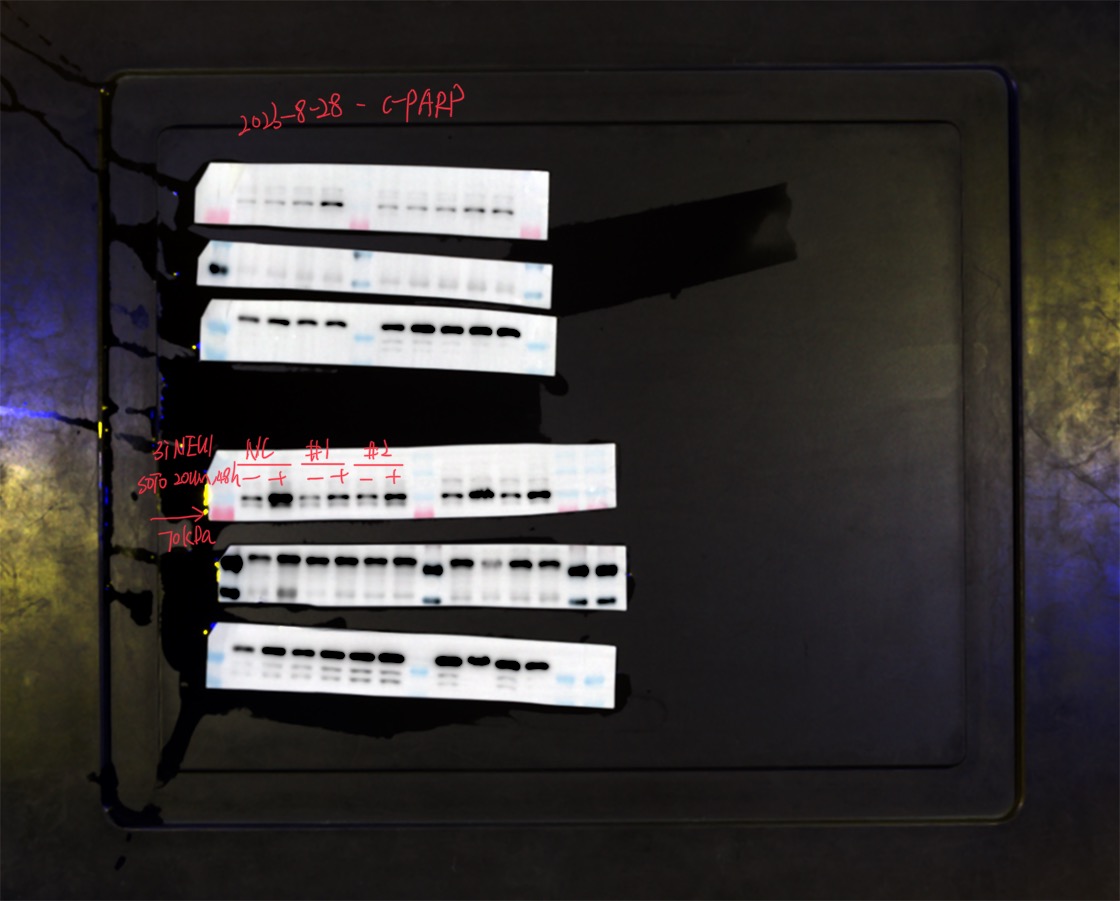

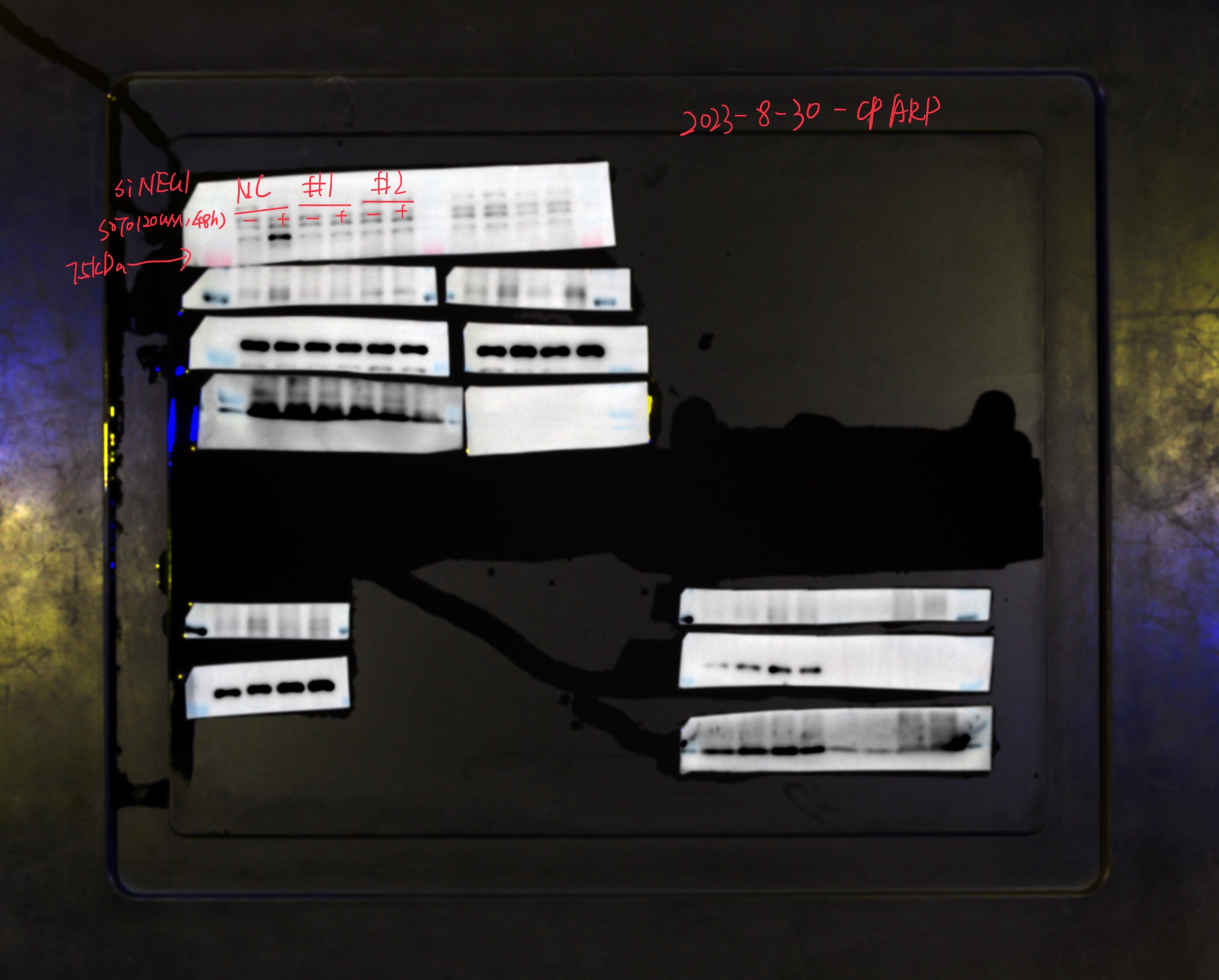

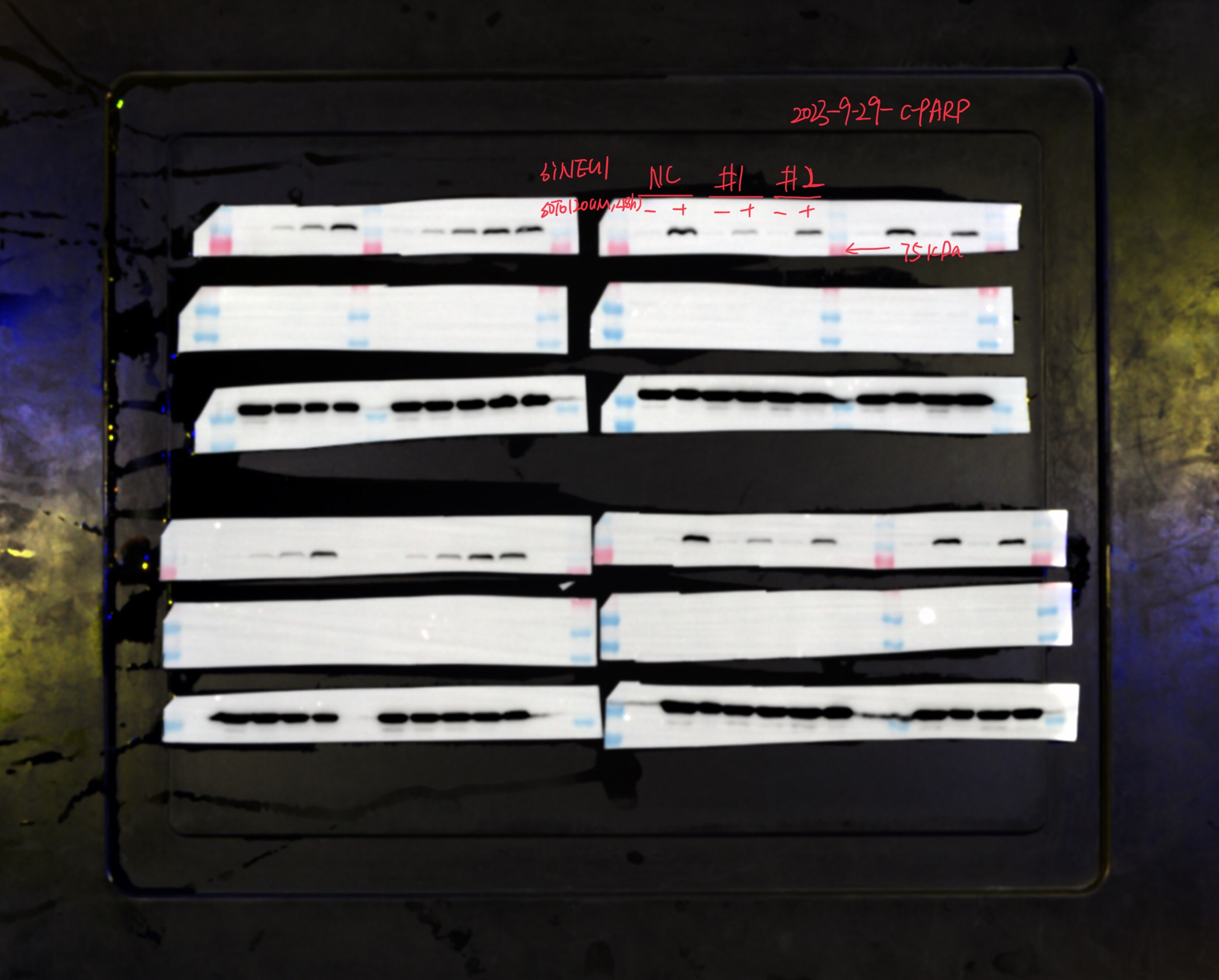


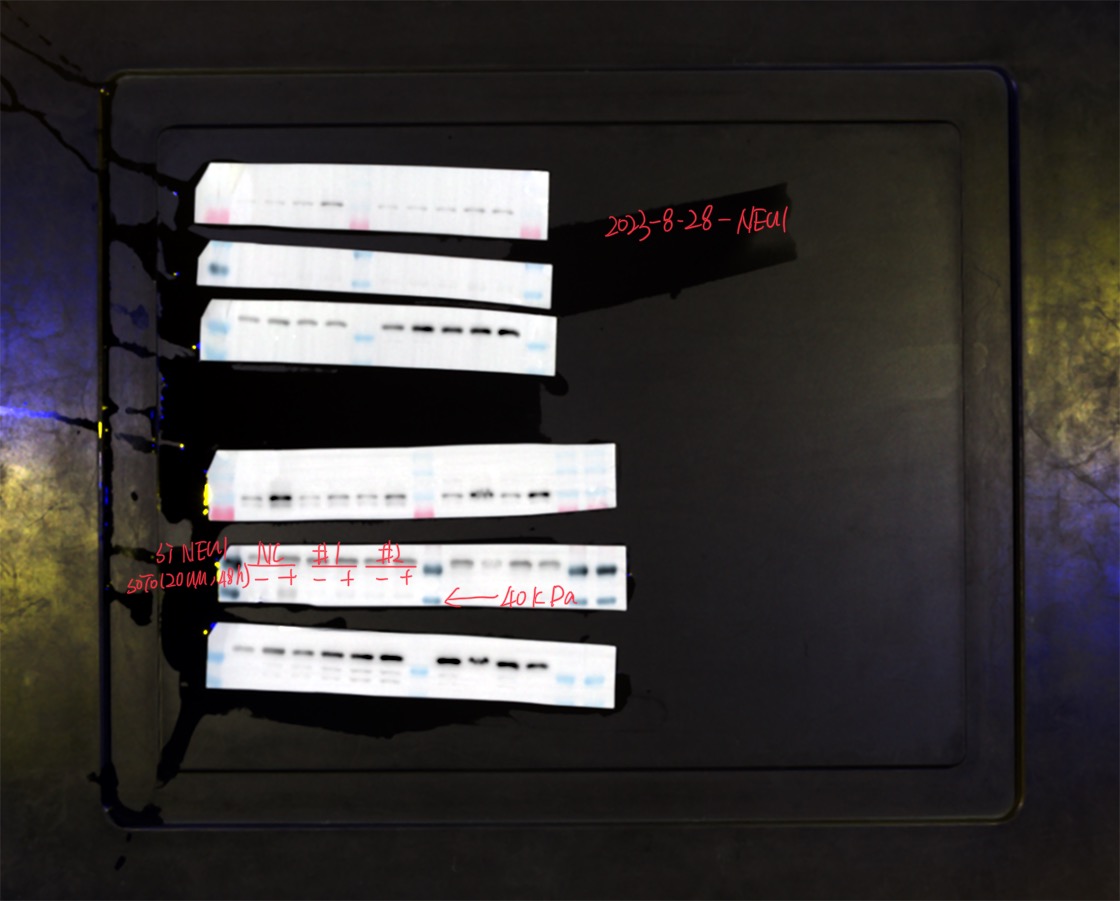

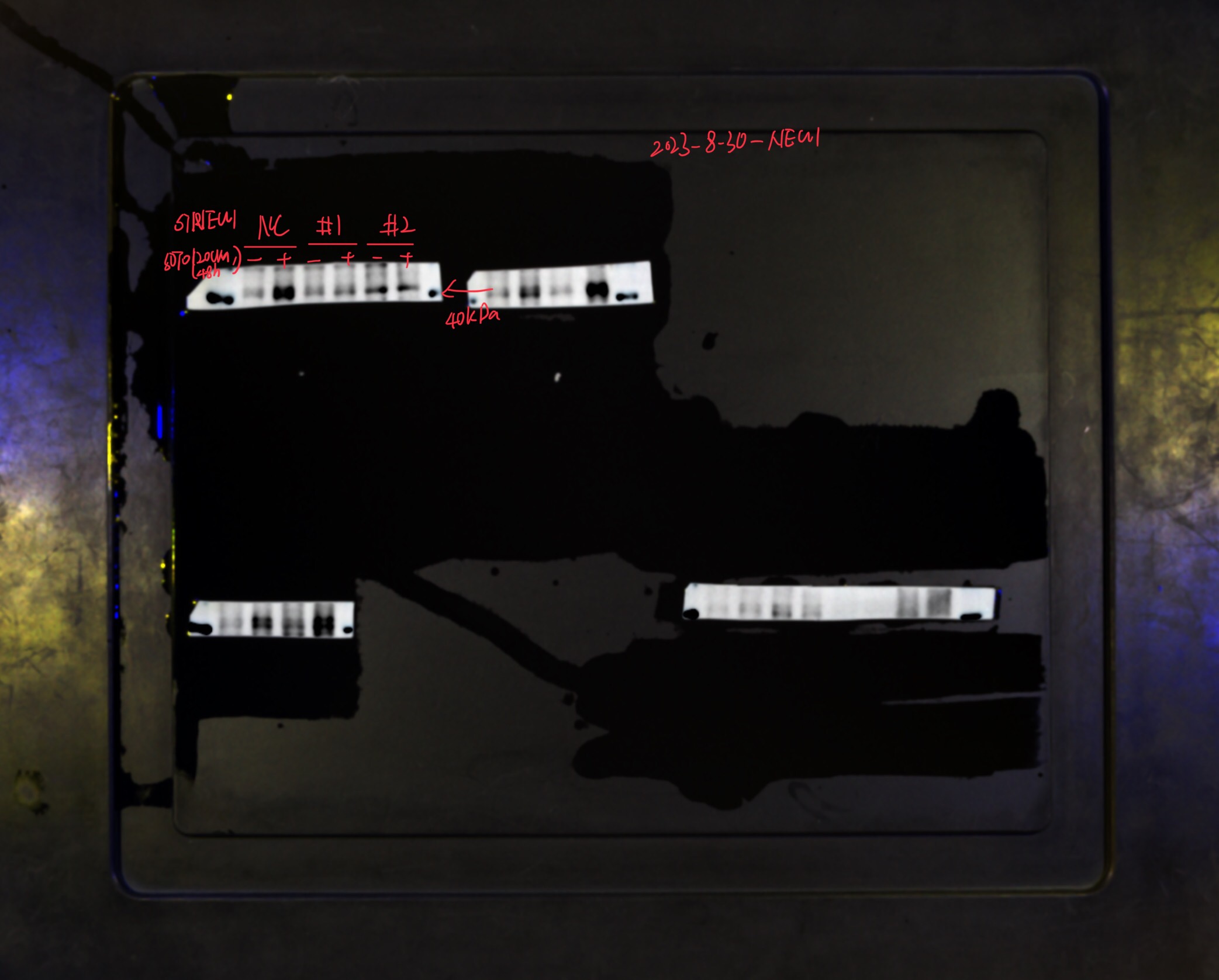

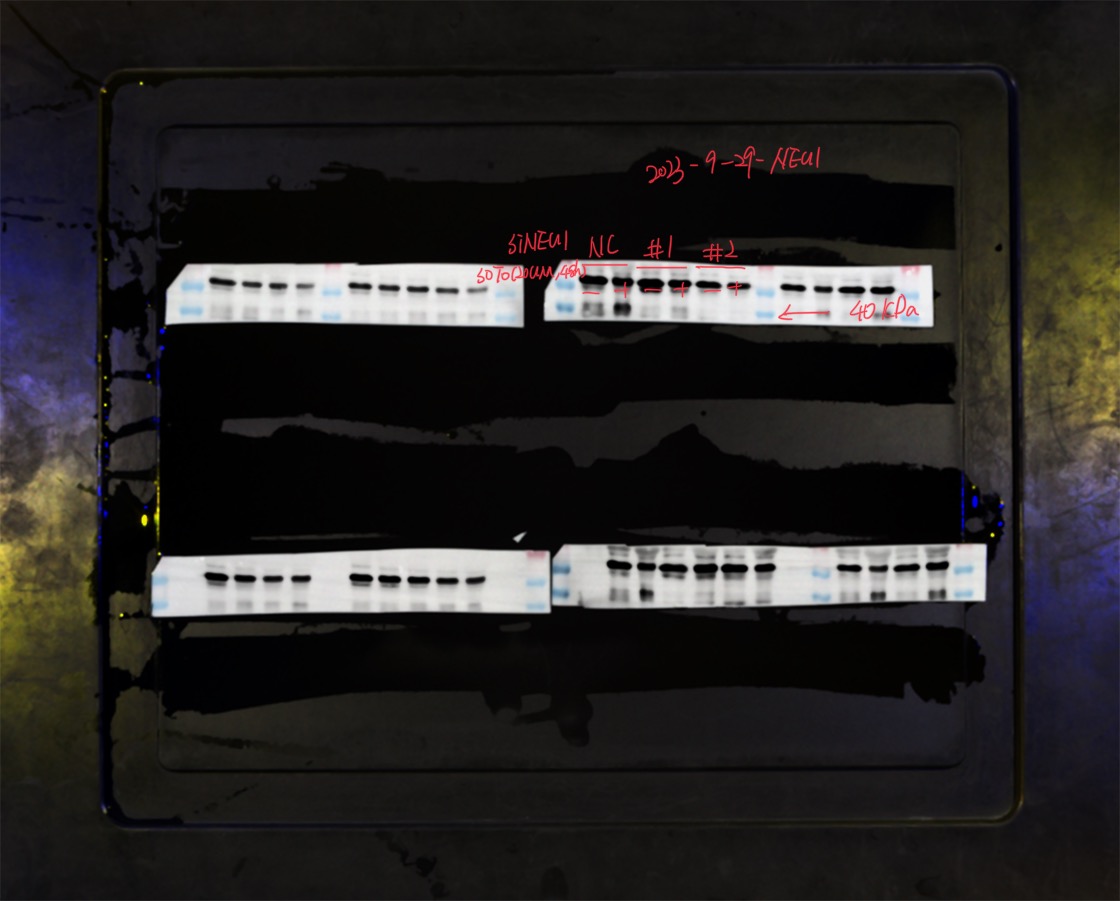


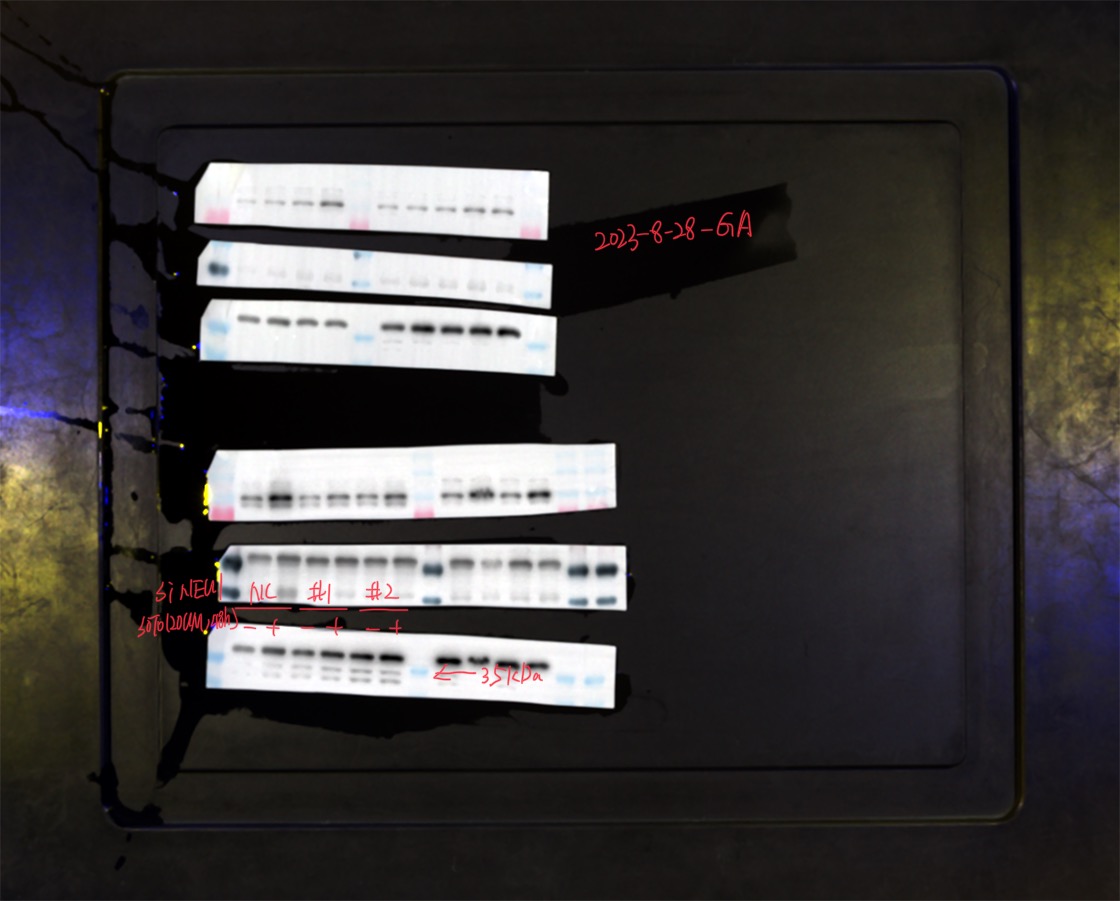

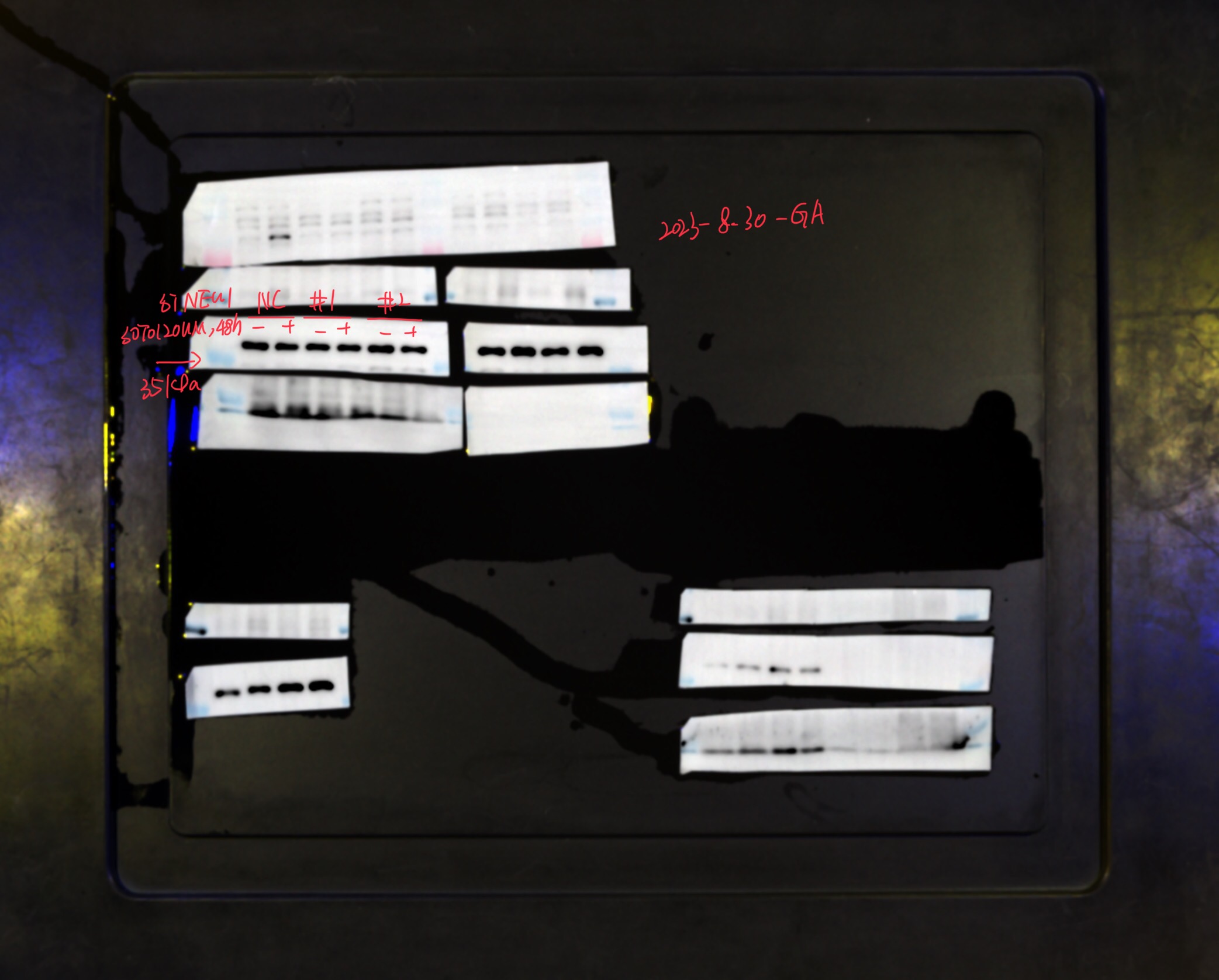

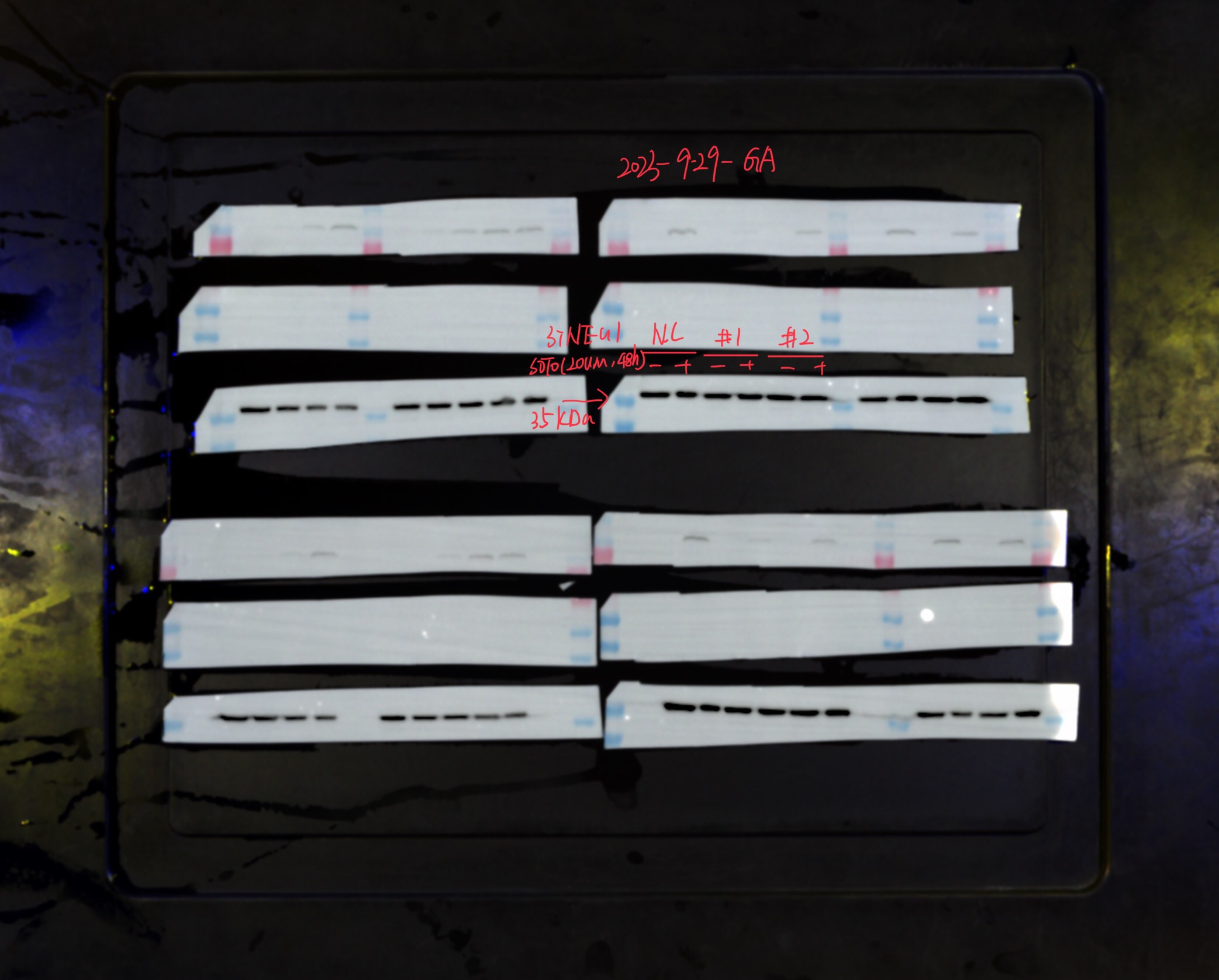


Fig. 3-H


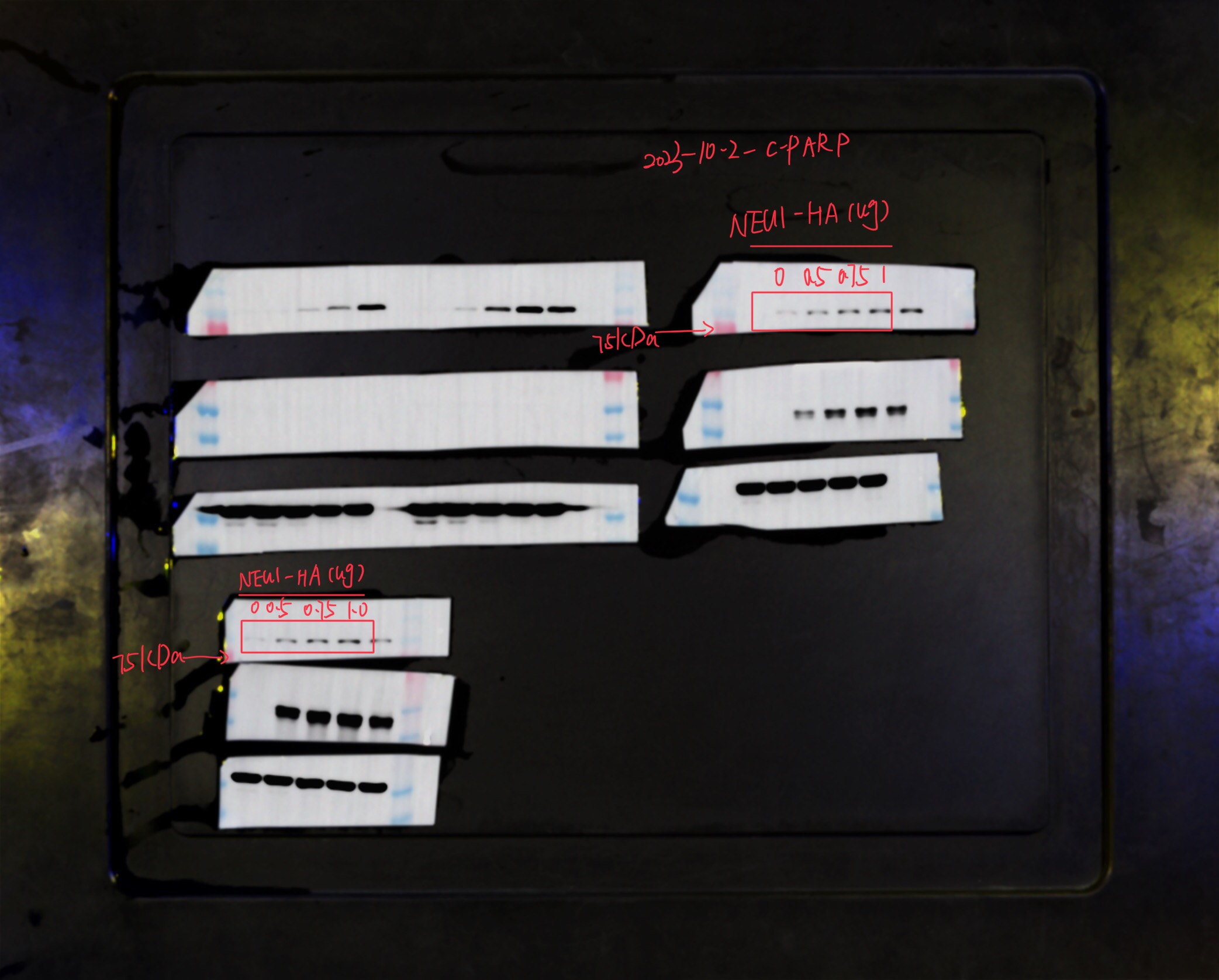

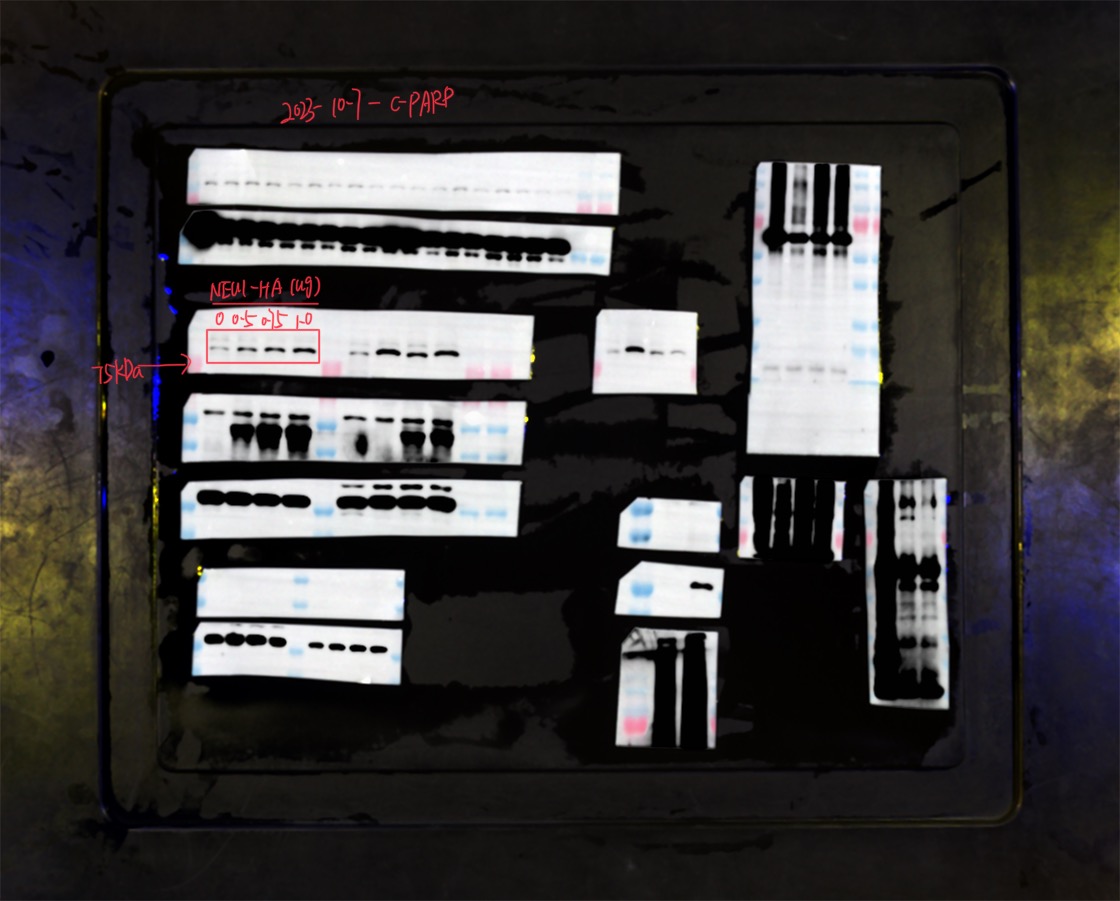


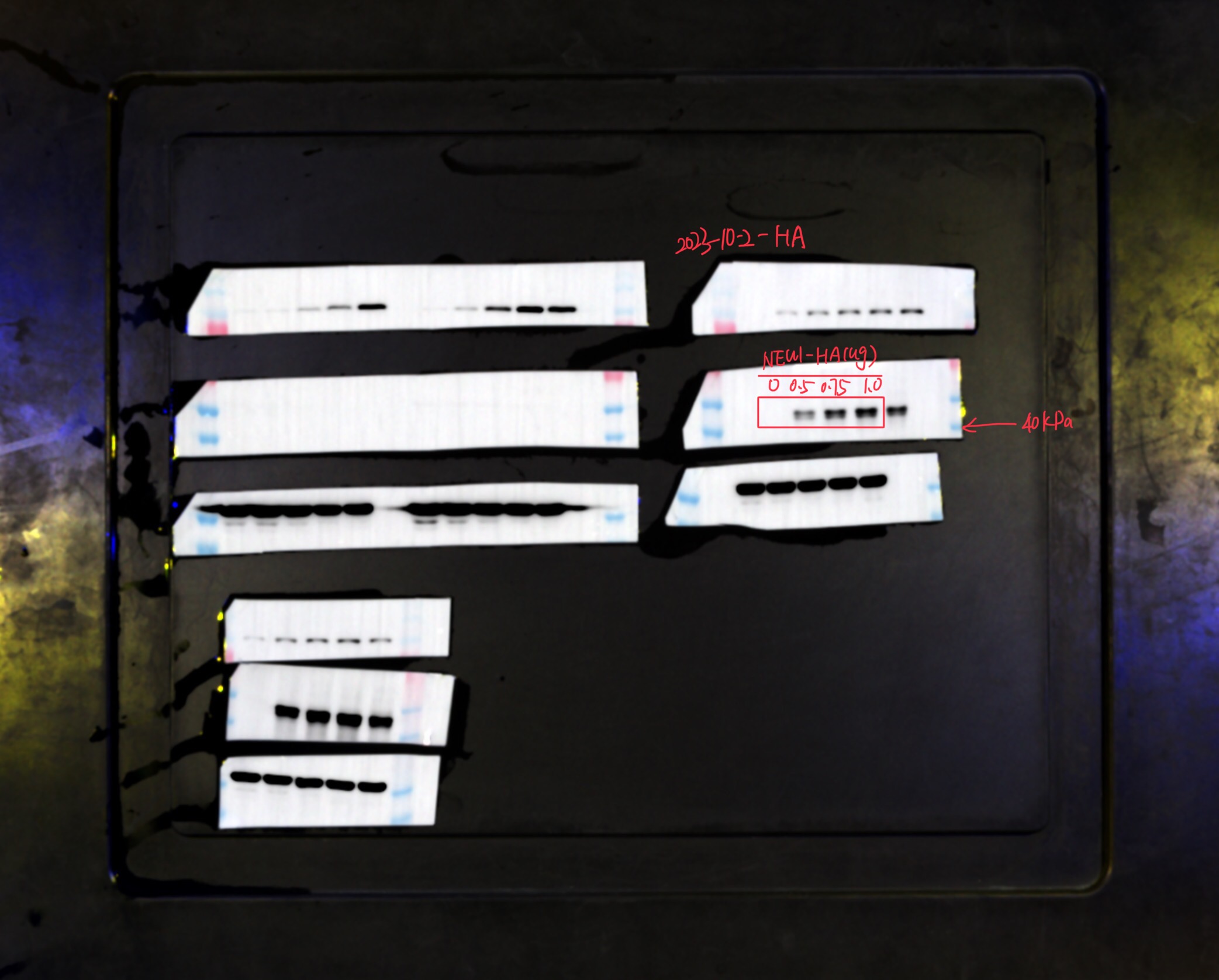

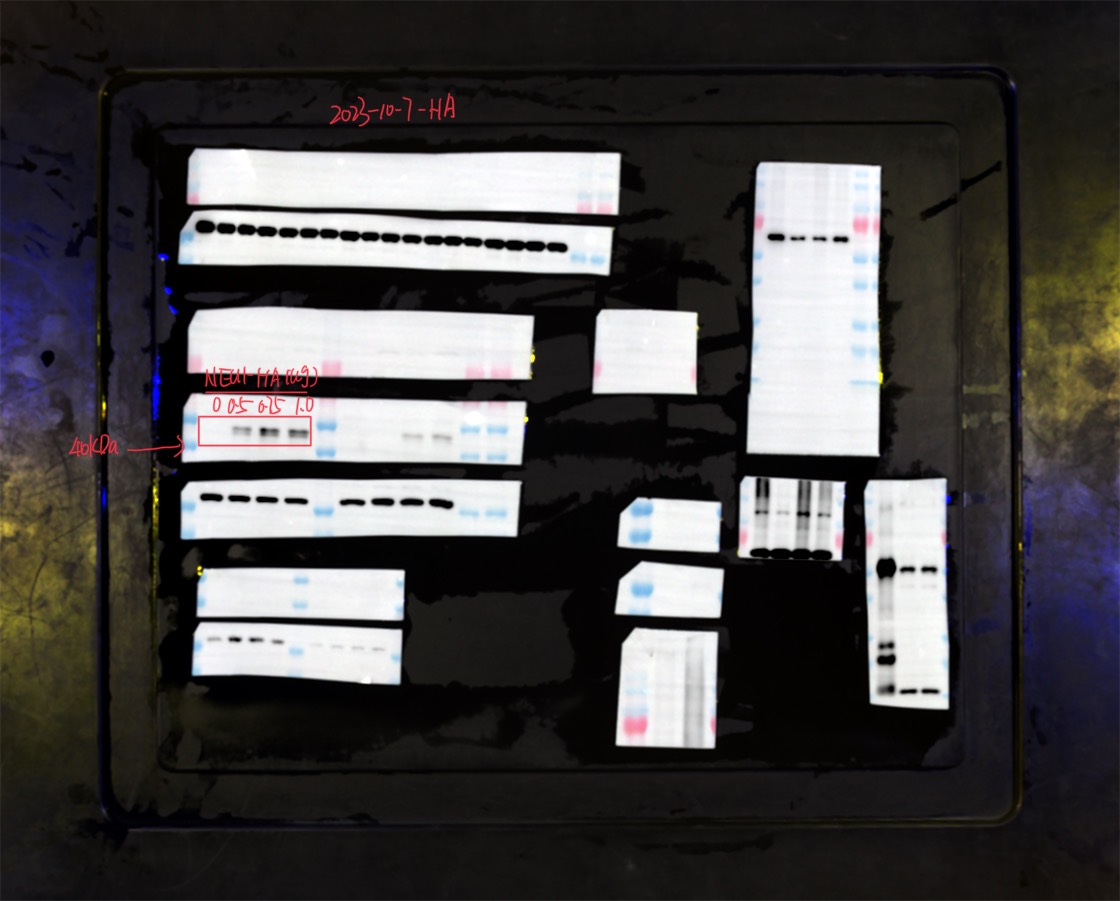


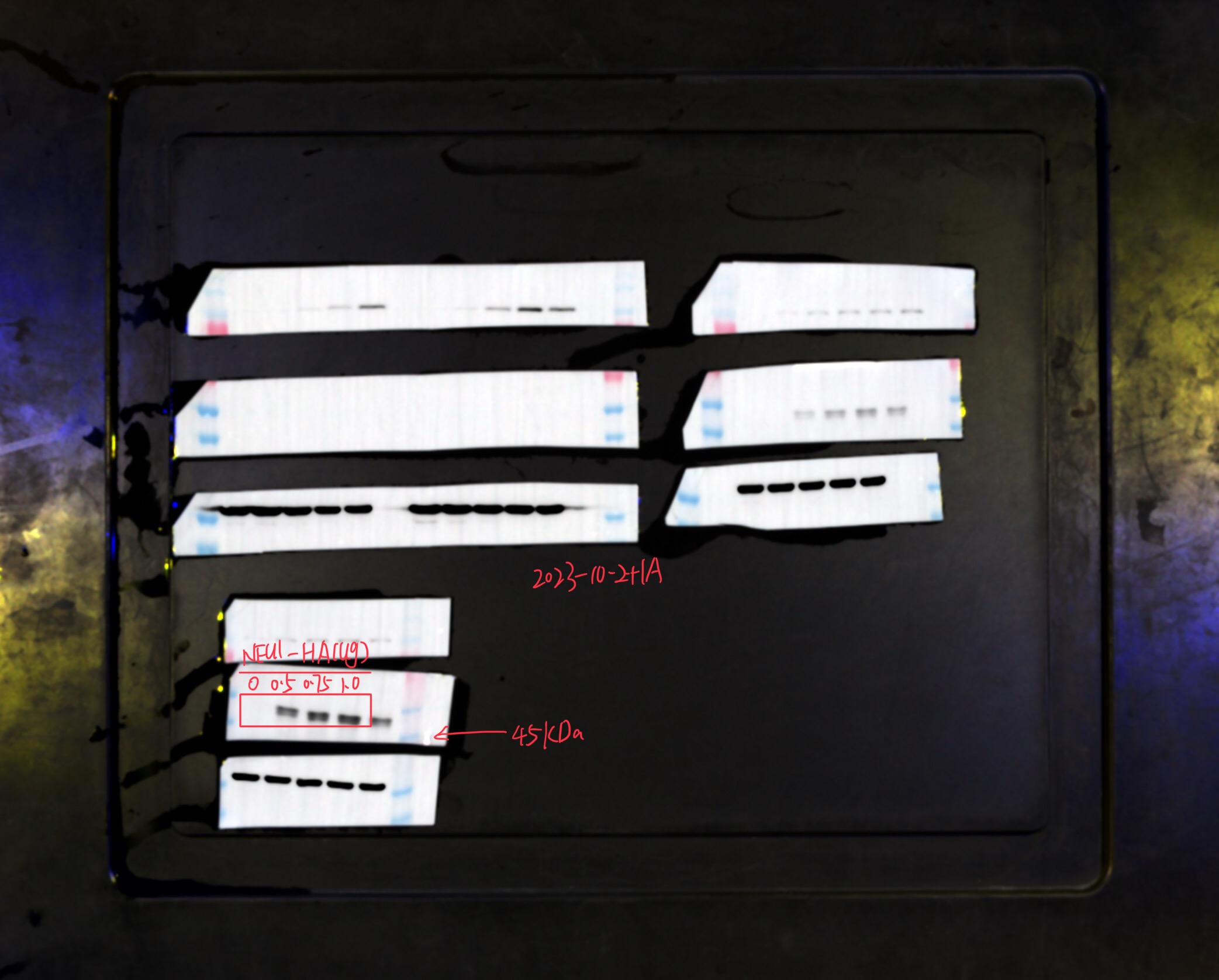

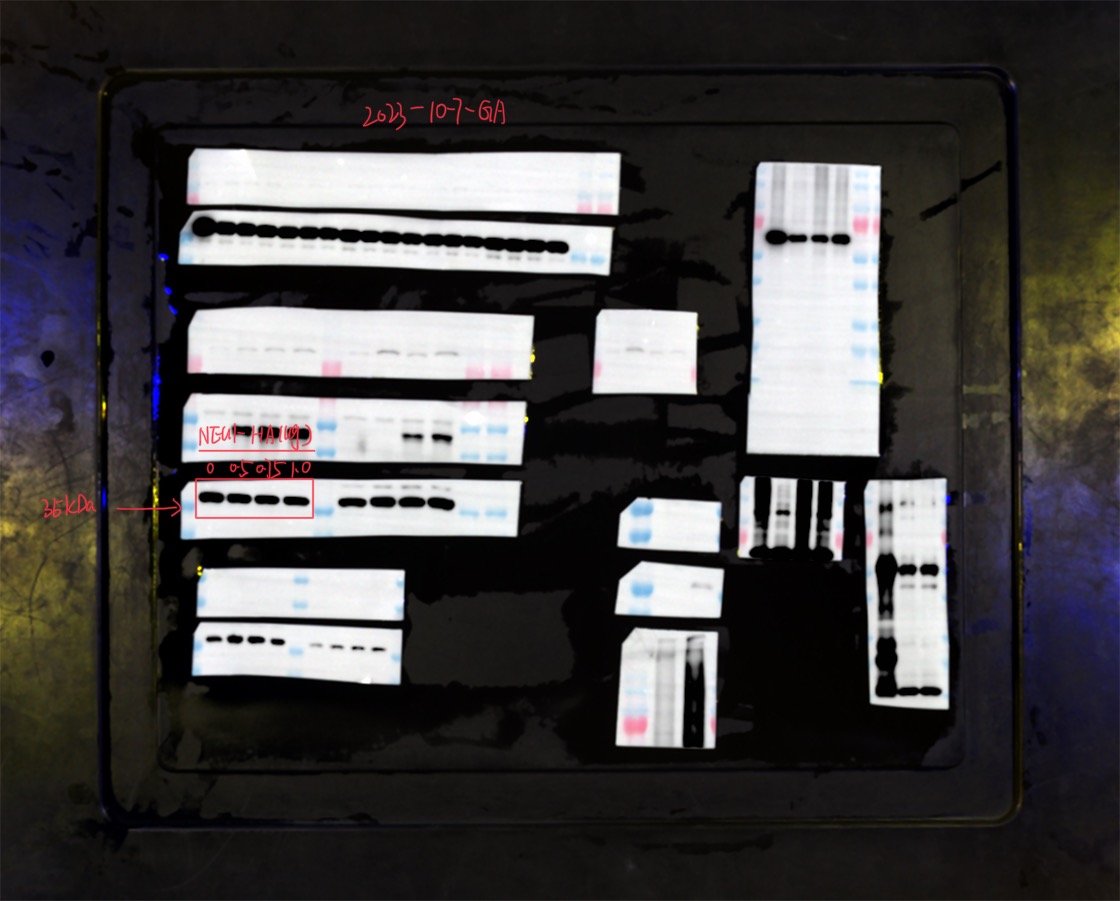


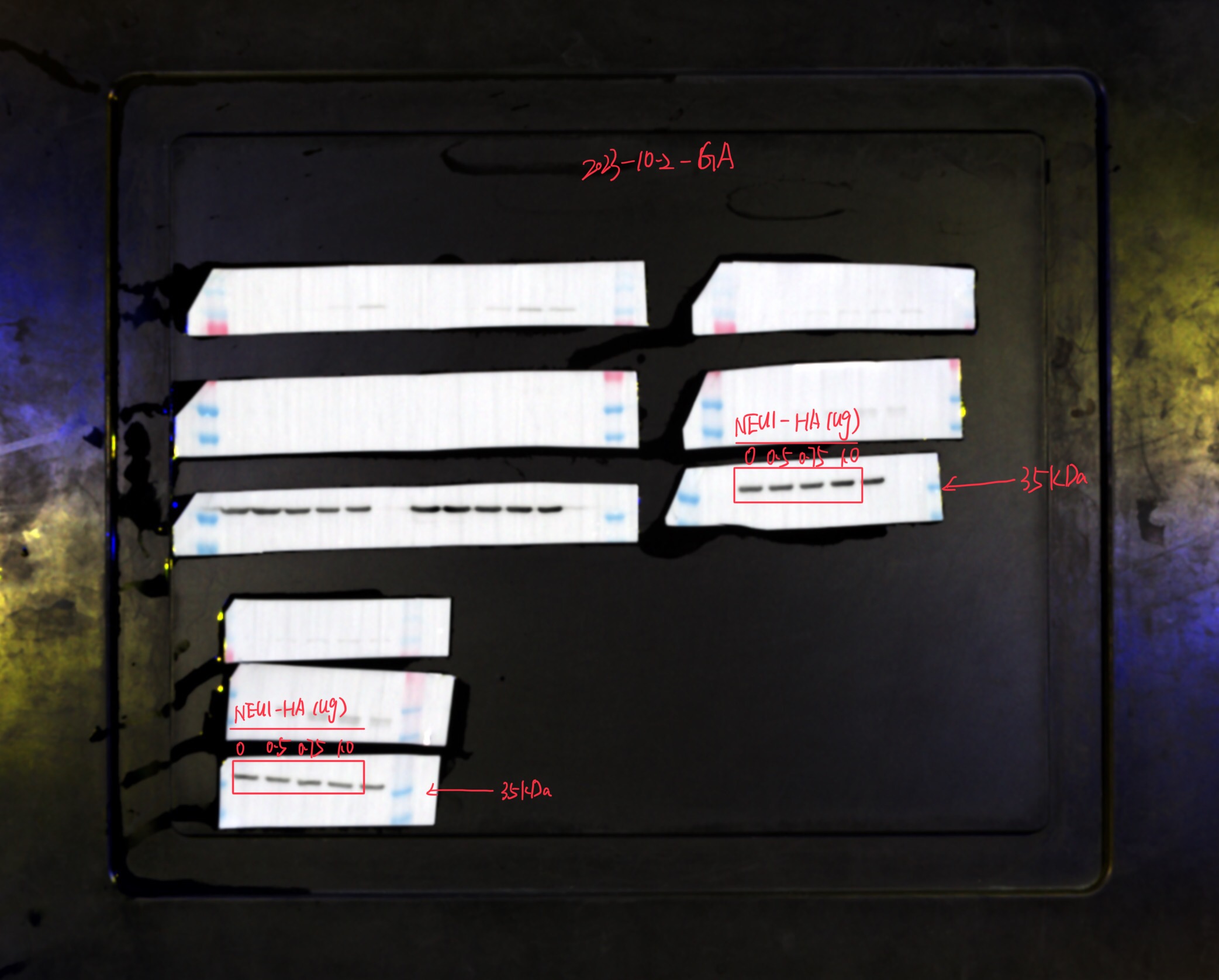


Fig. 3-I


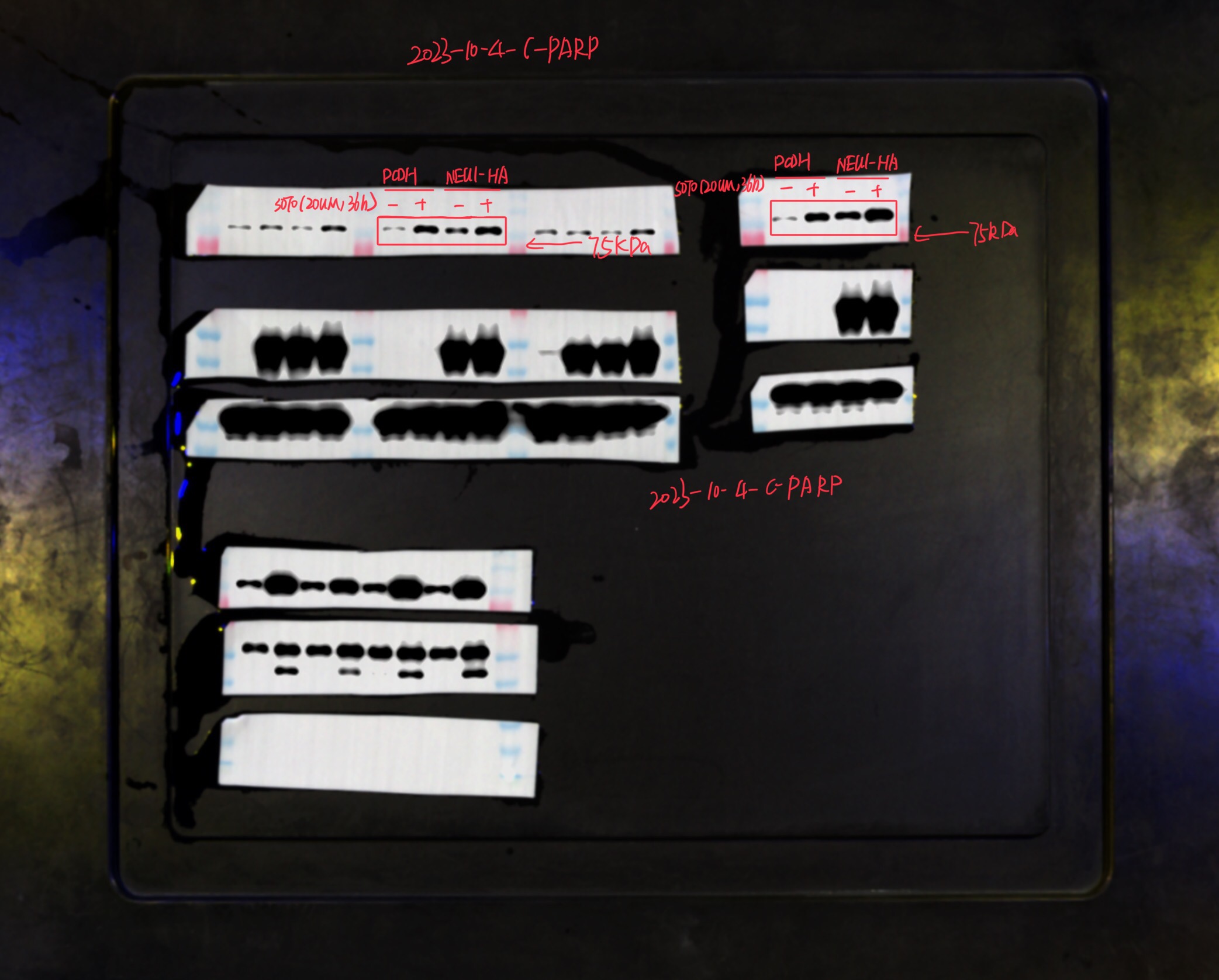

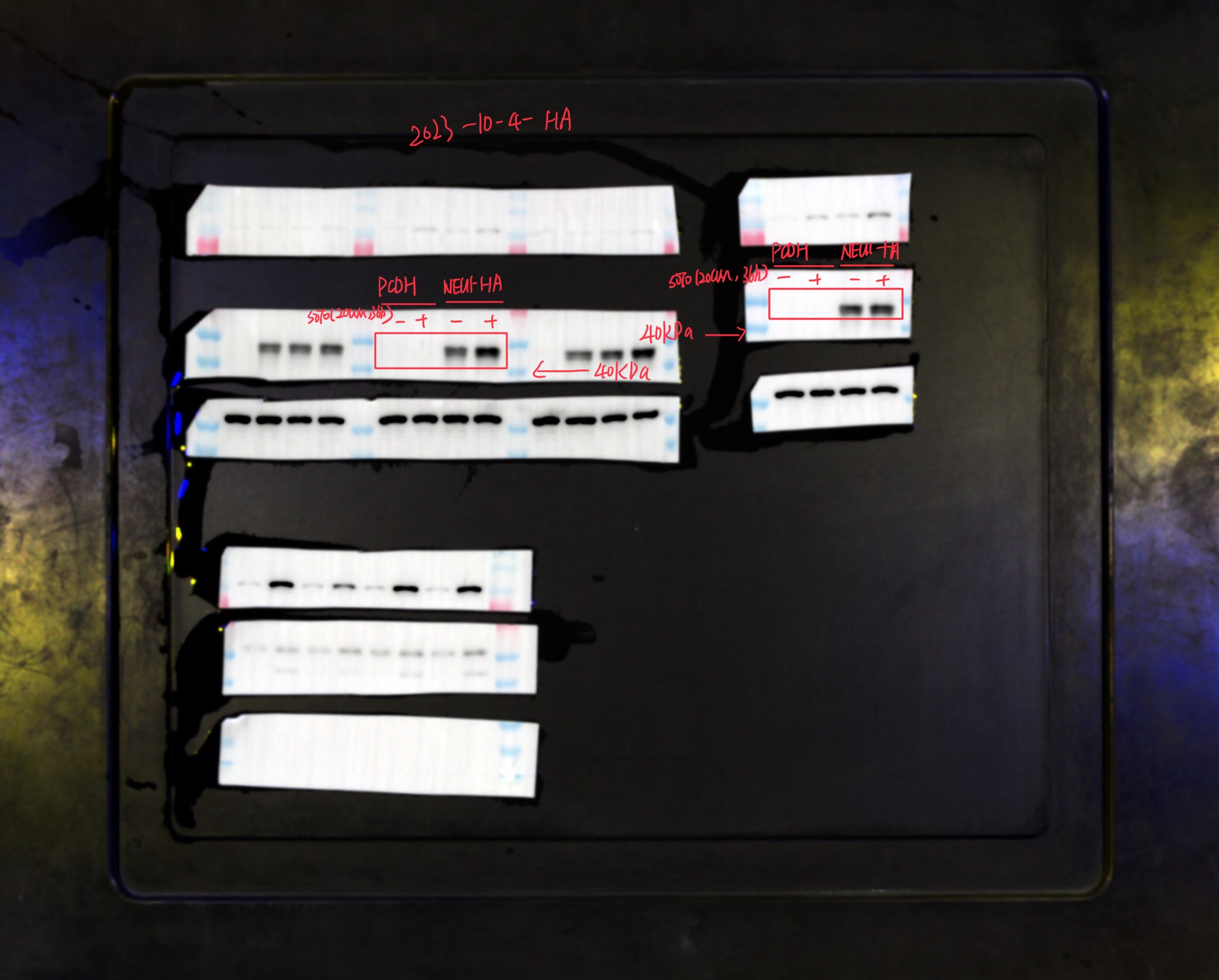


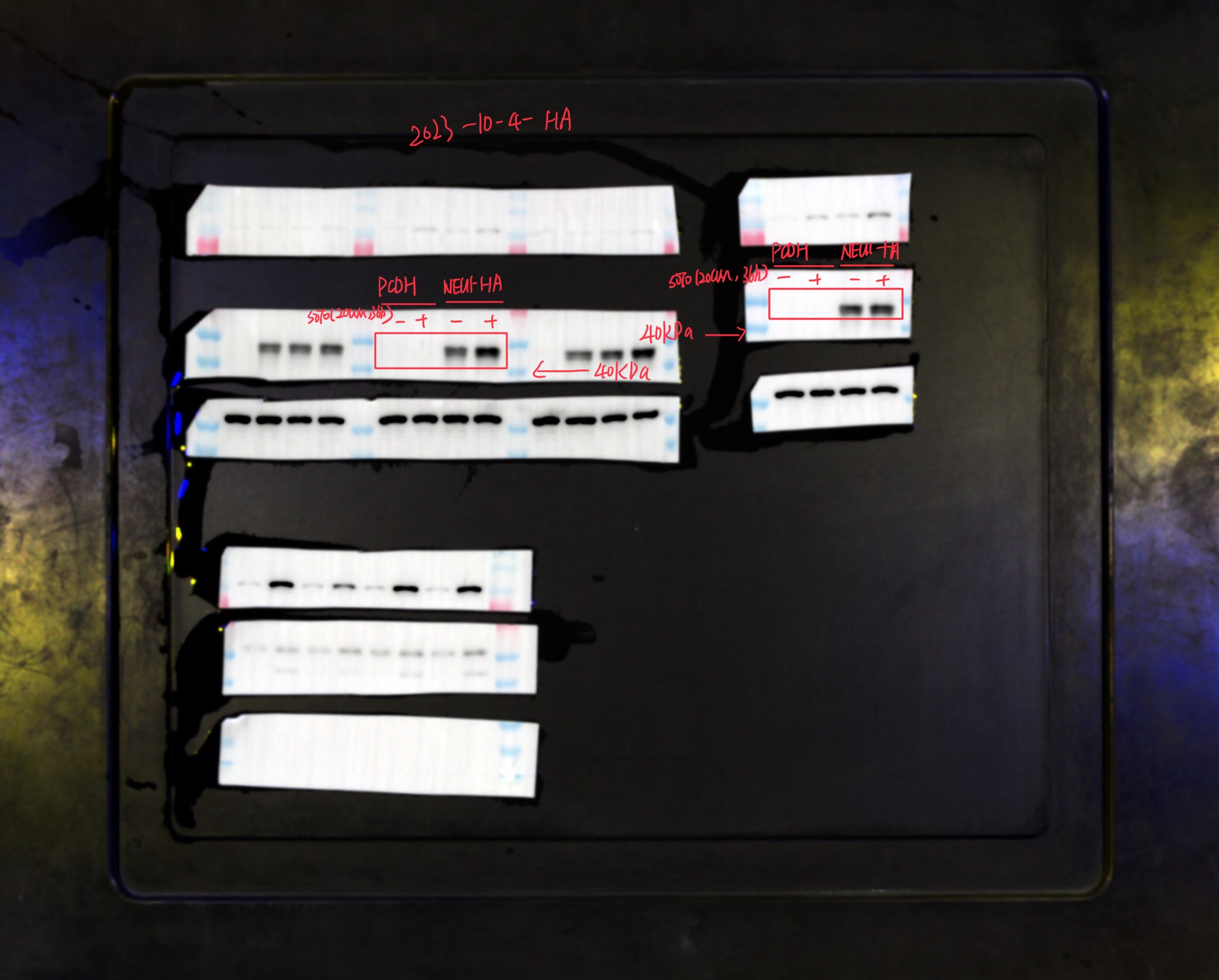

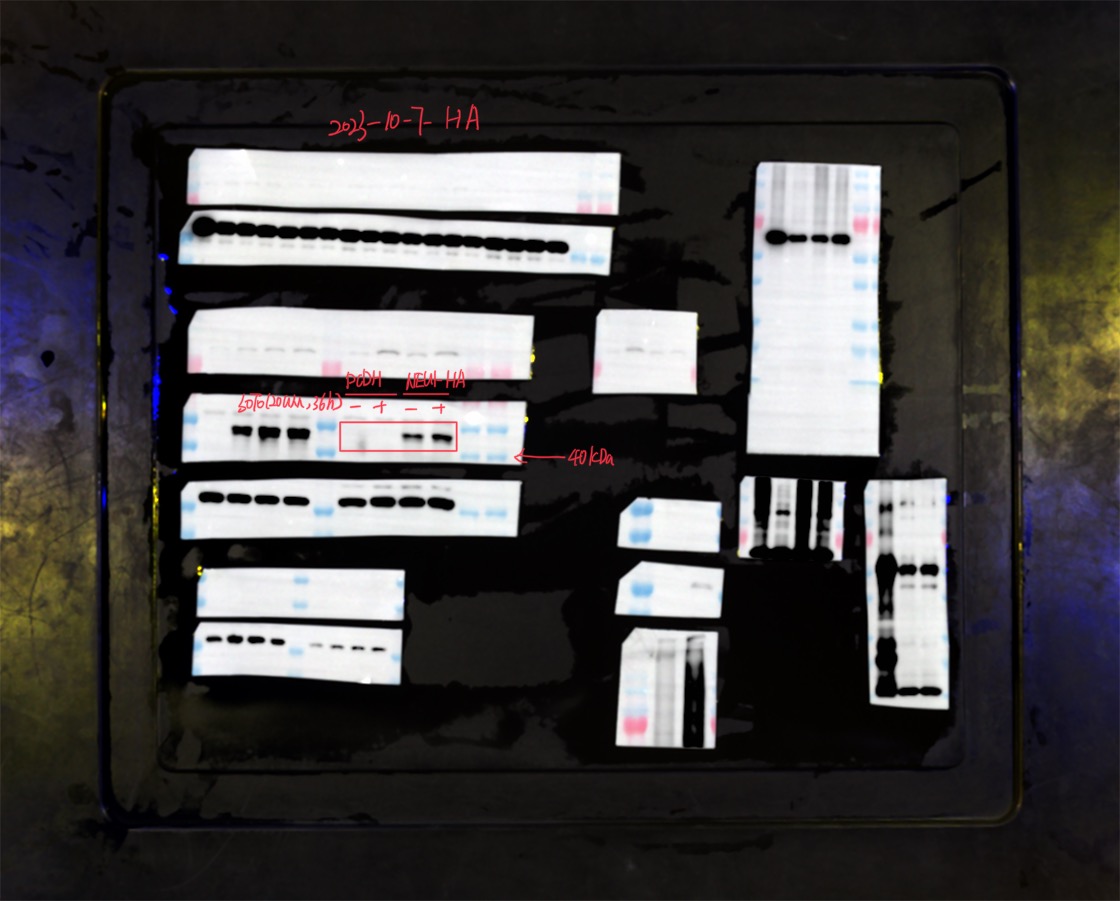


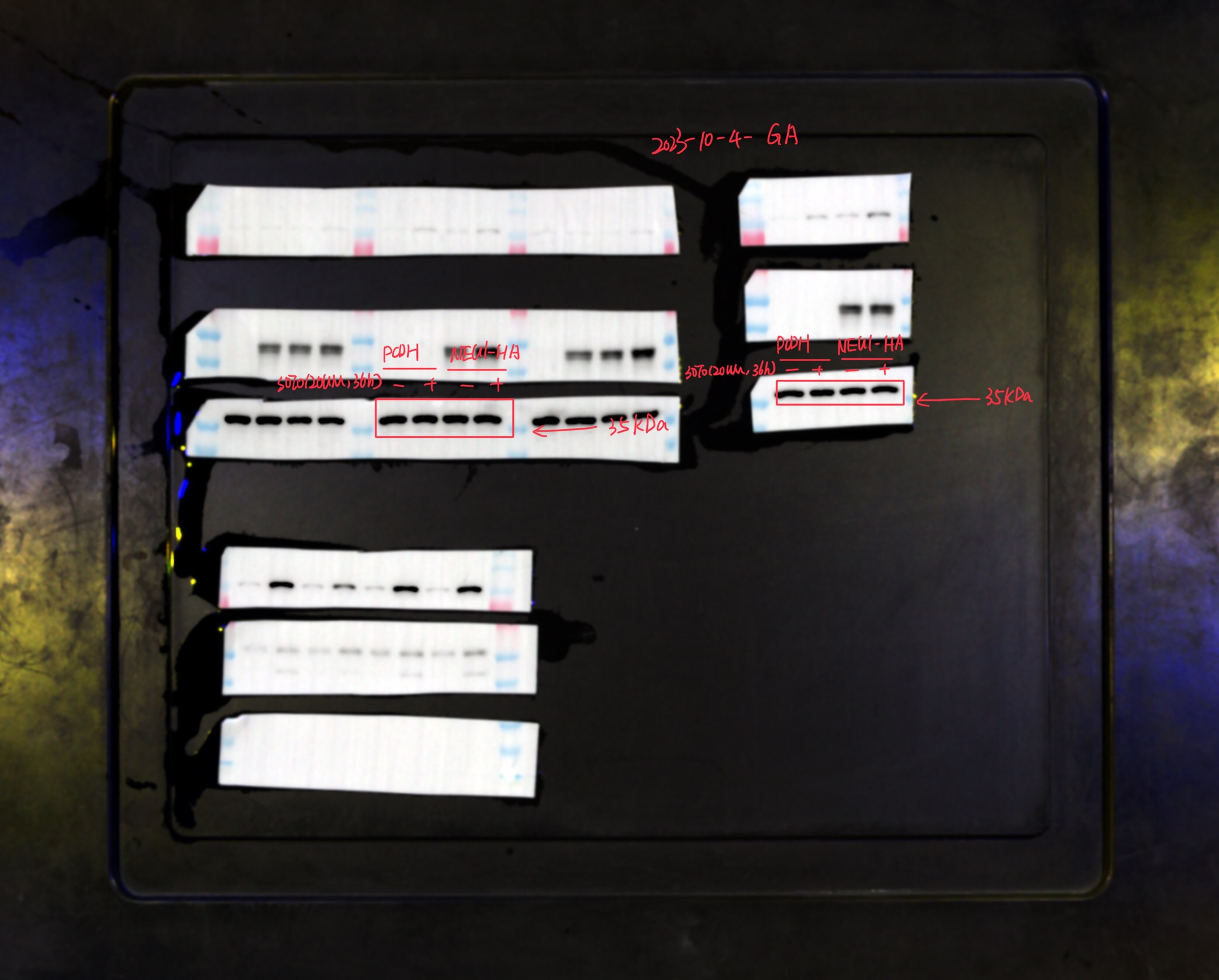

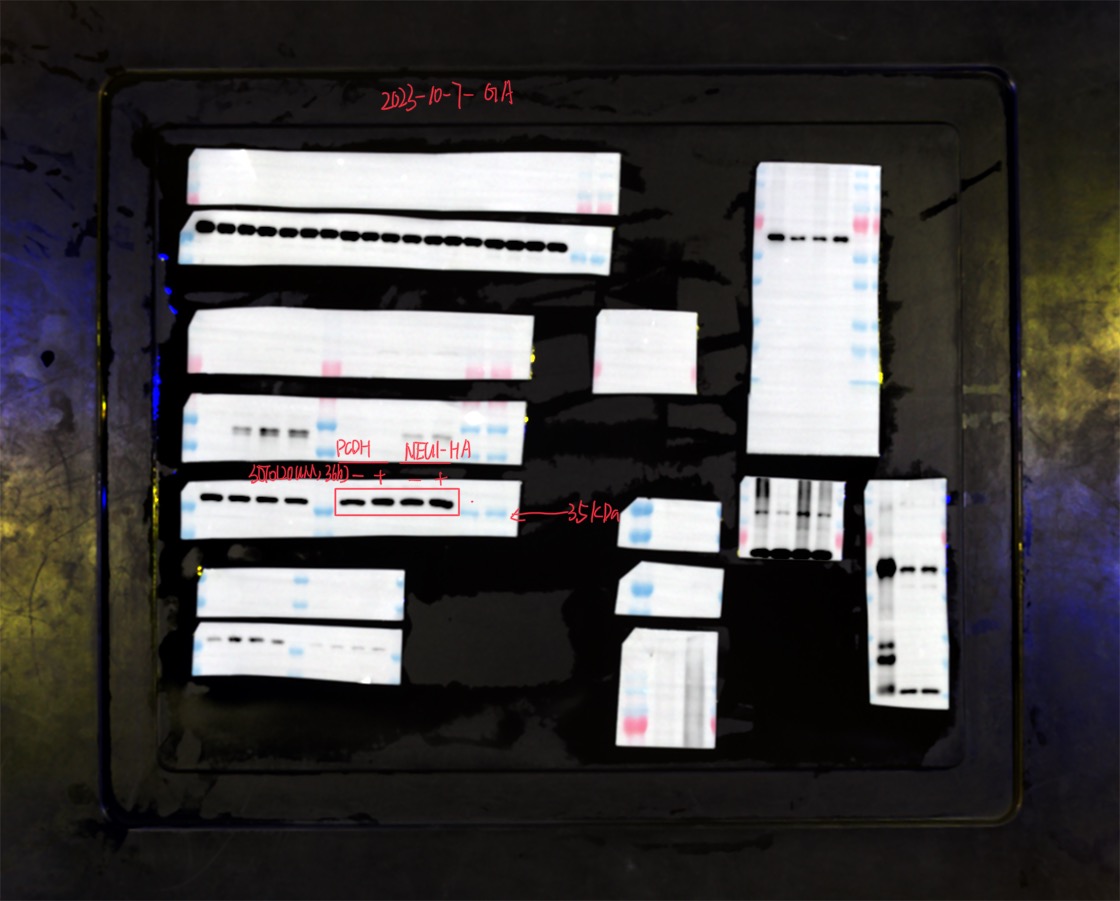


Fig. 4-A-D


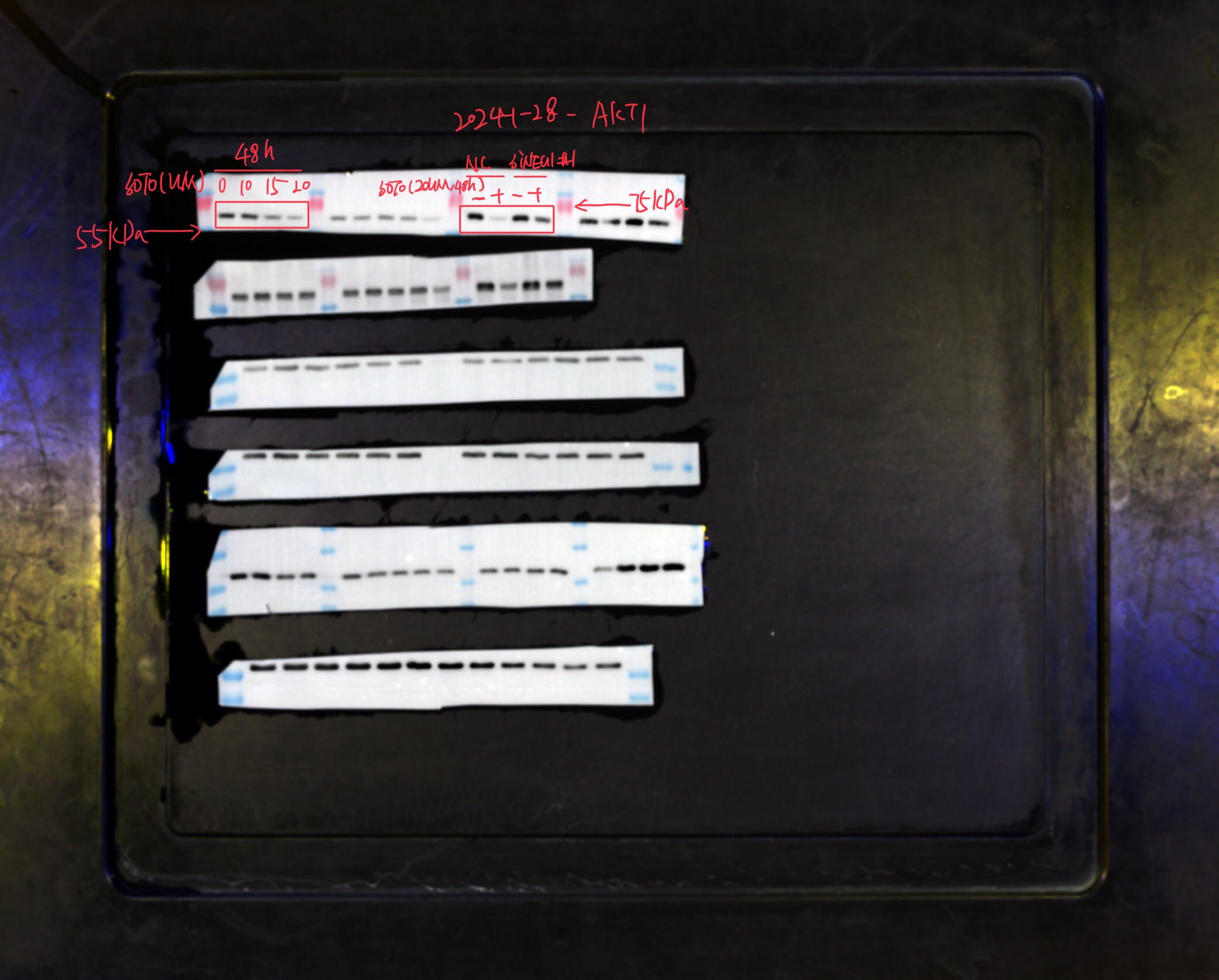

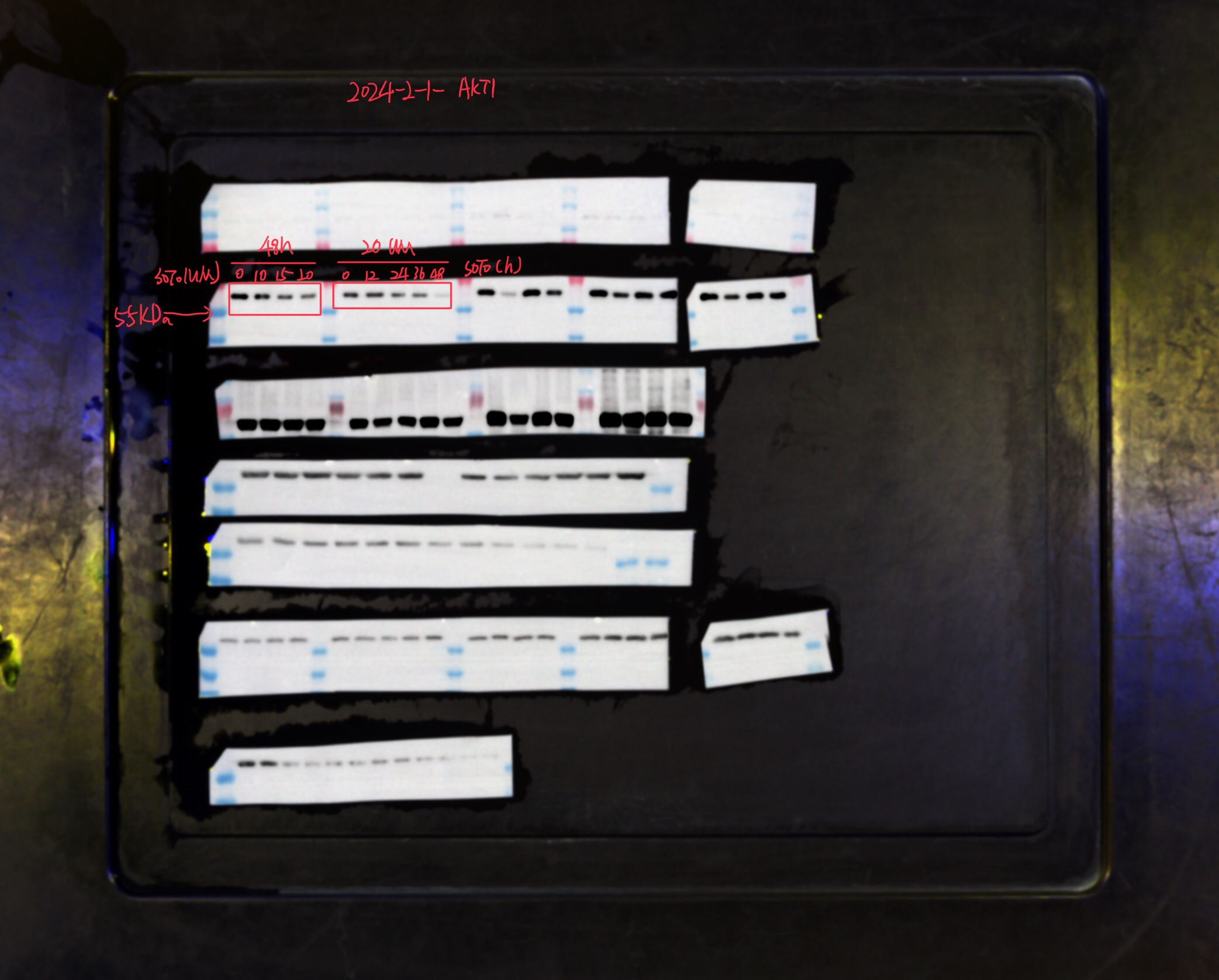


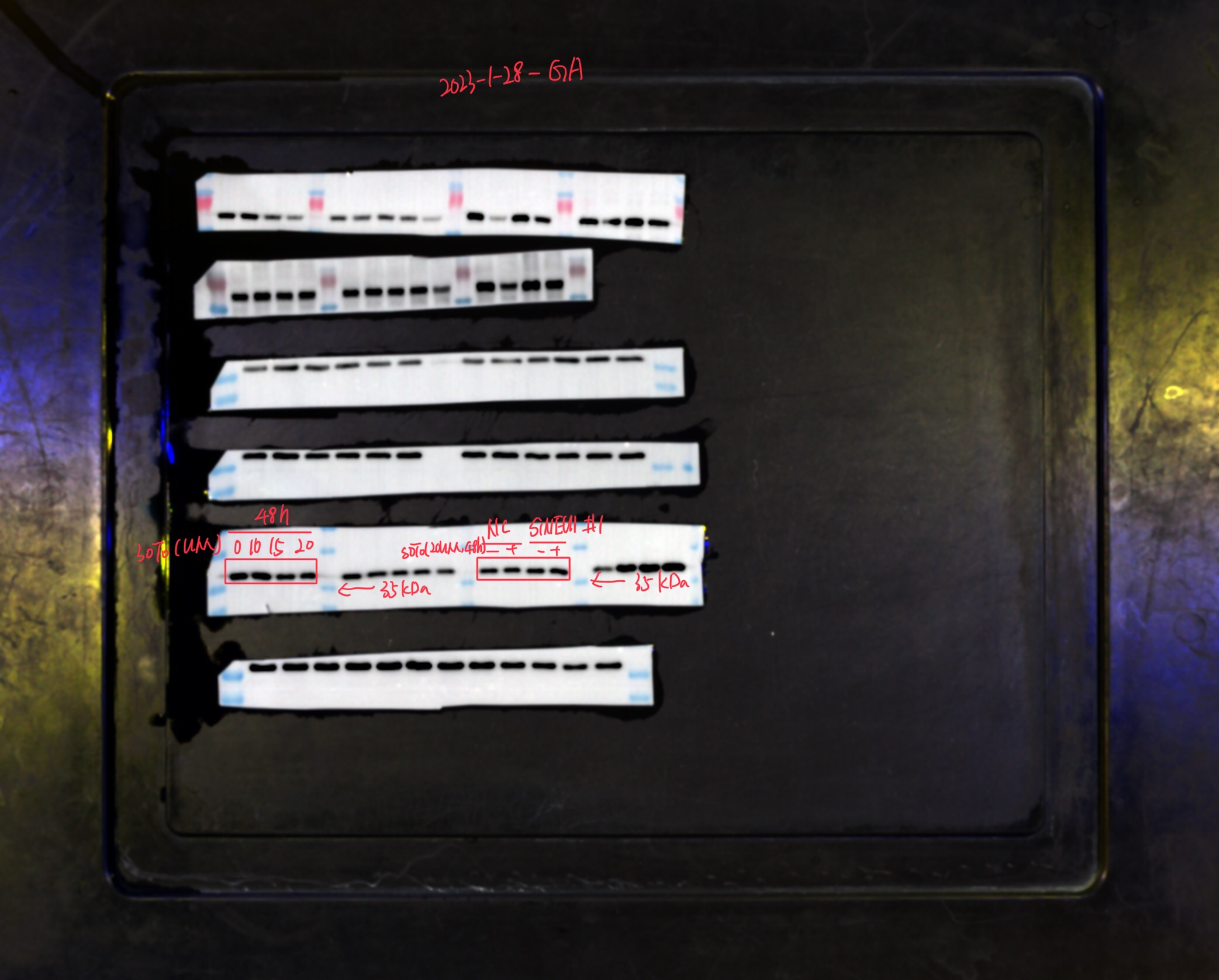

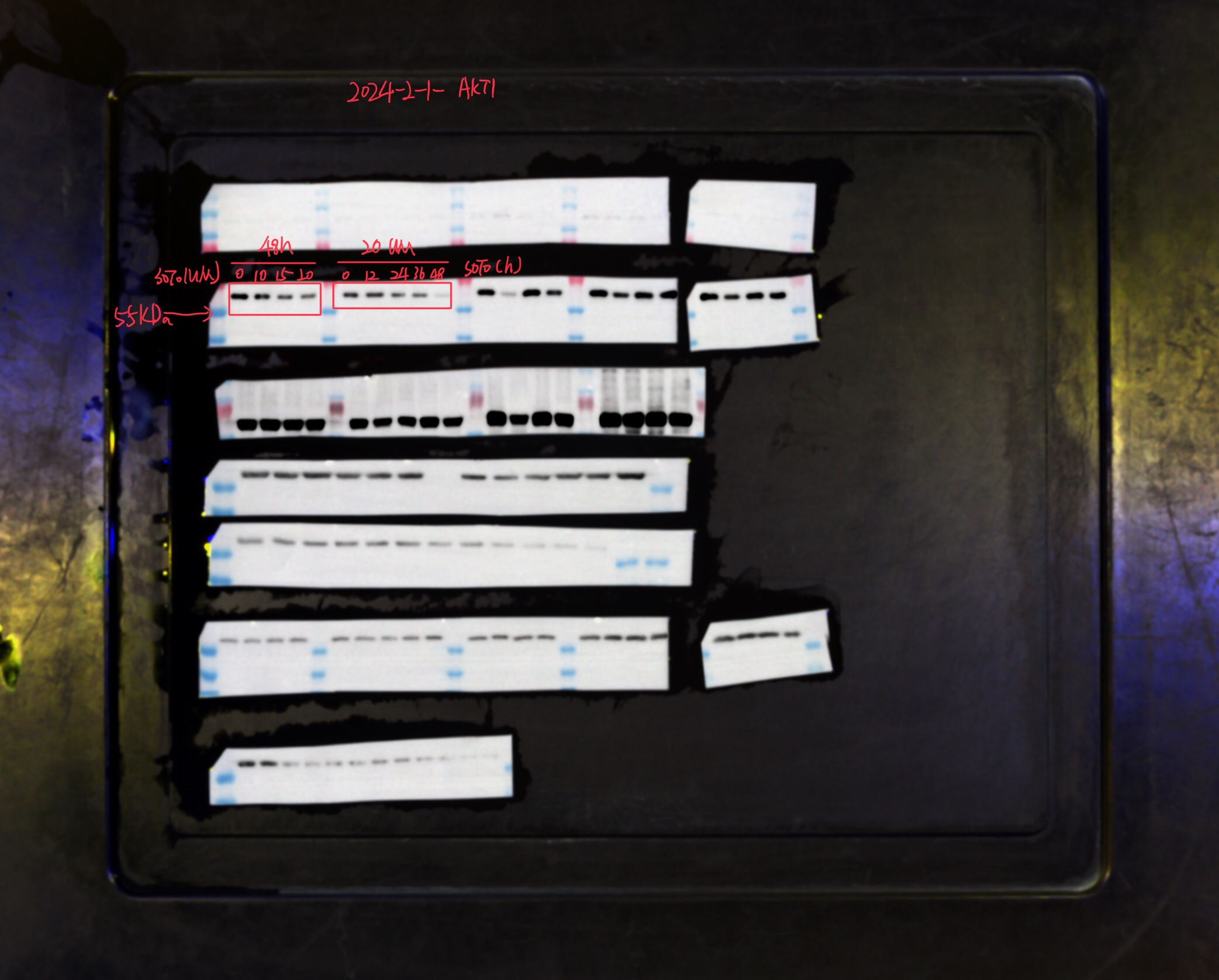


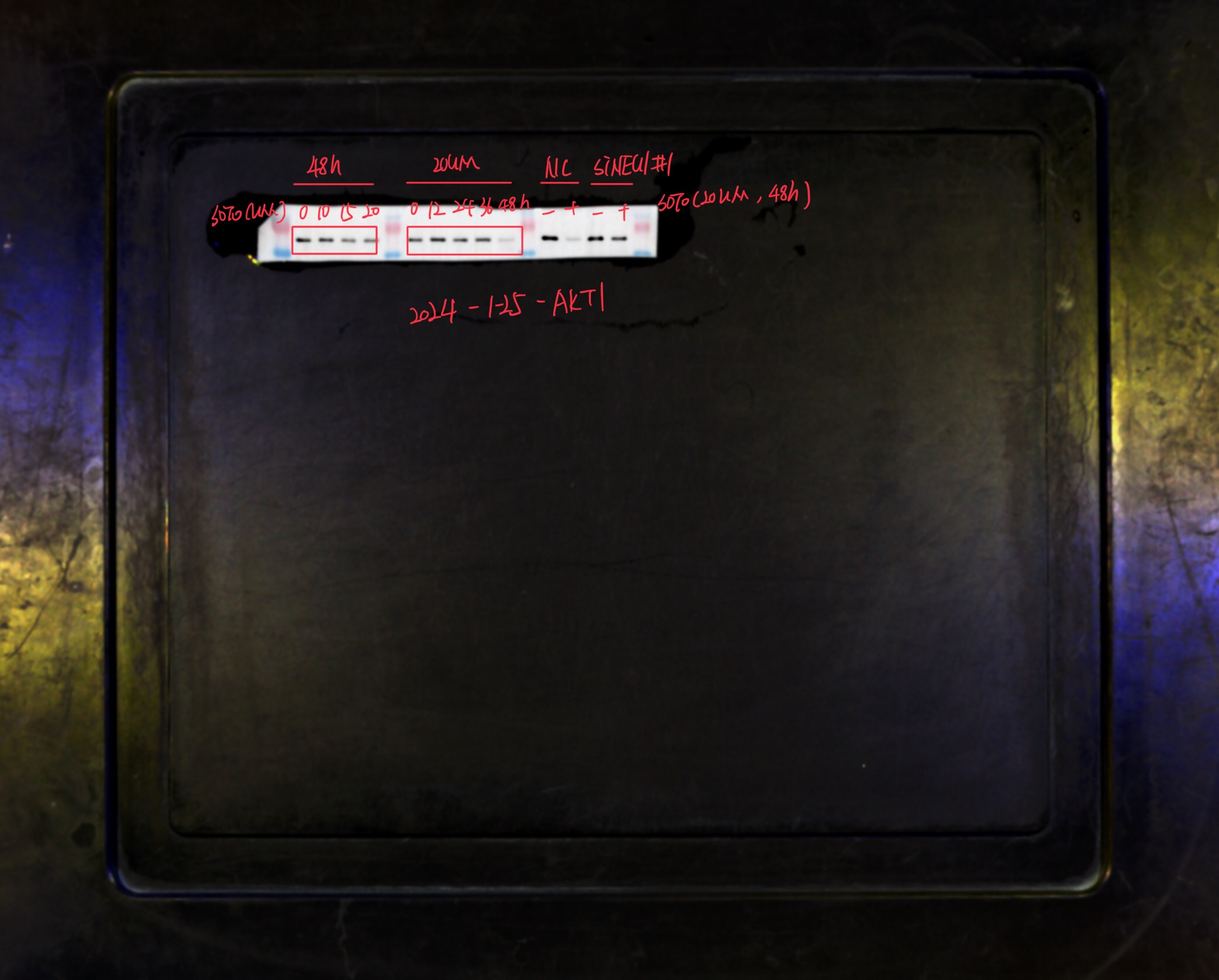

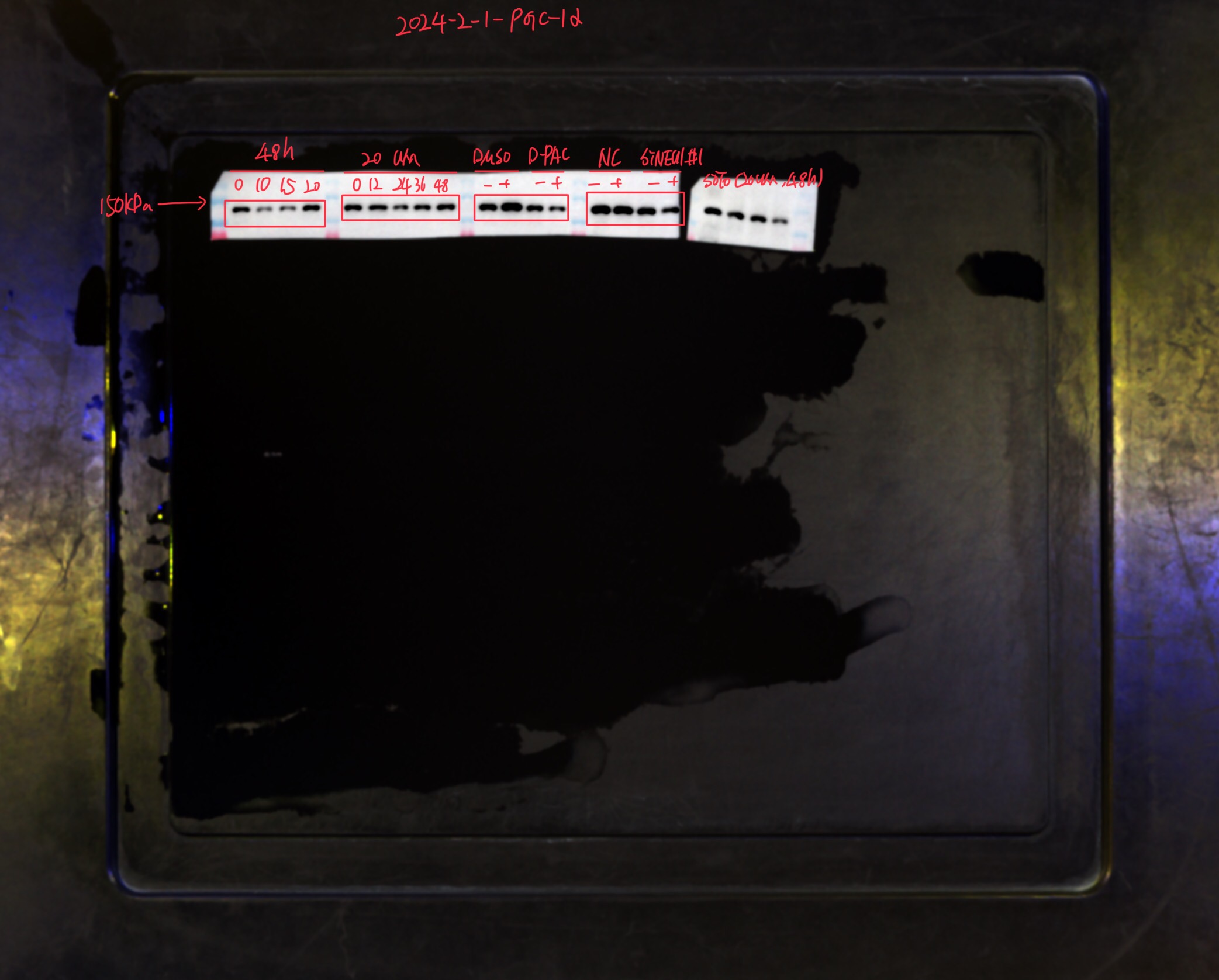

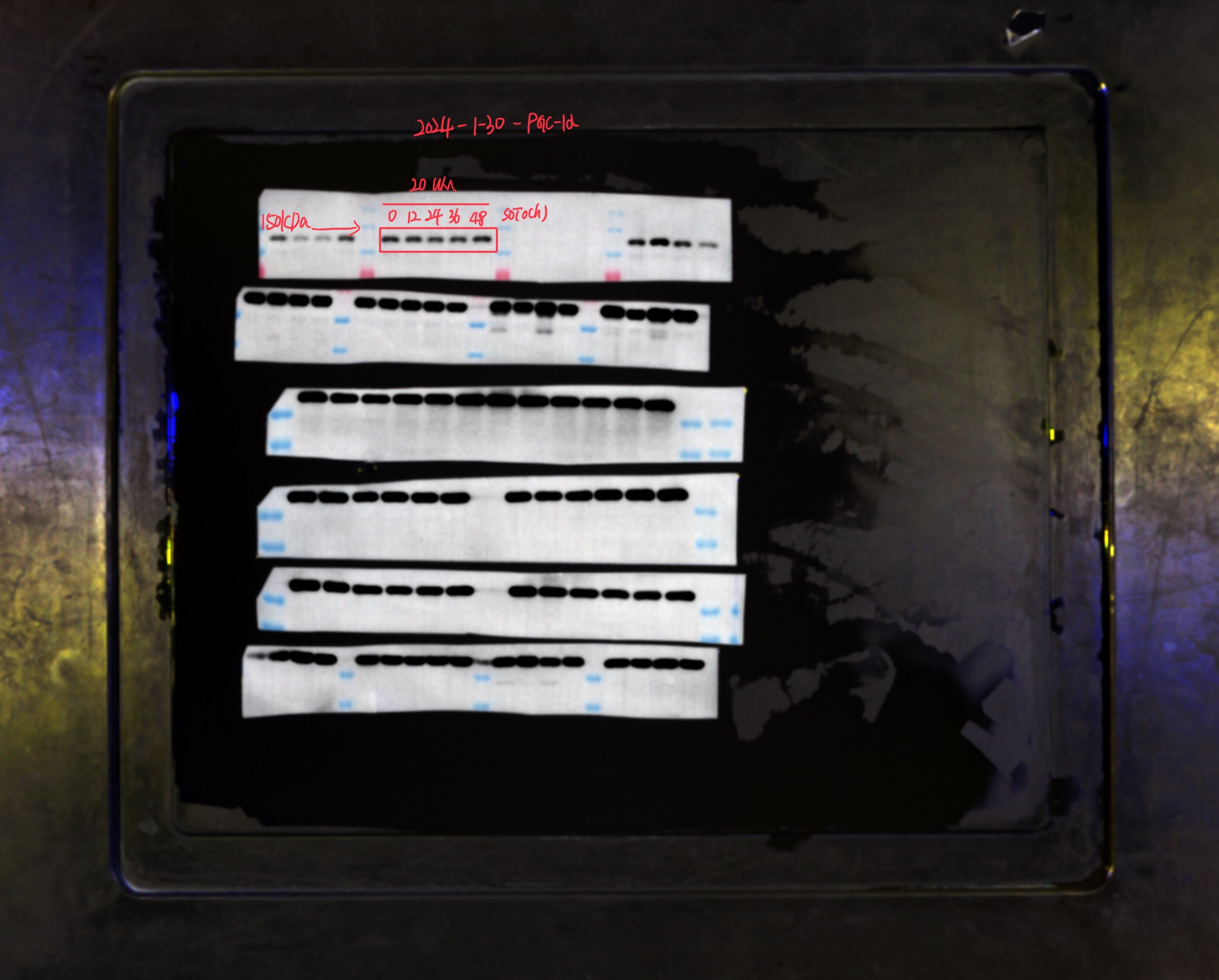


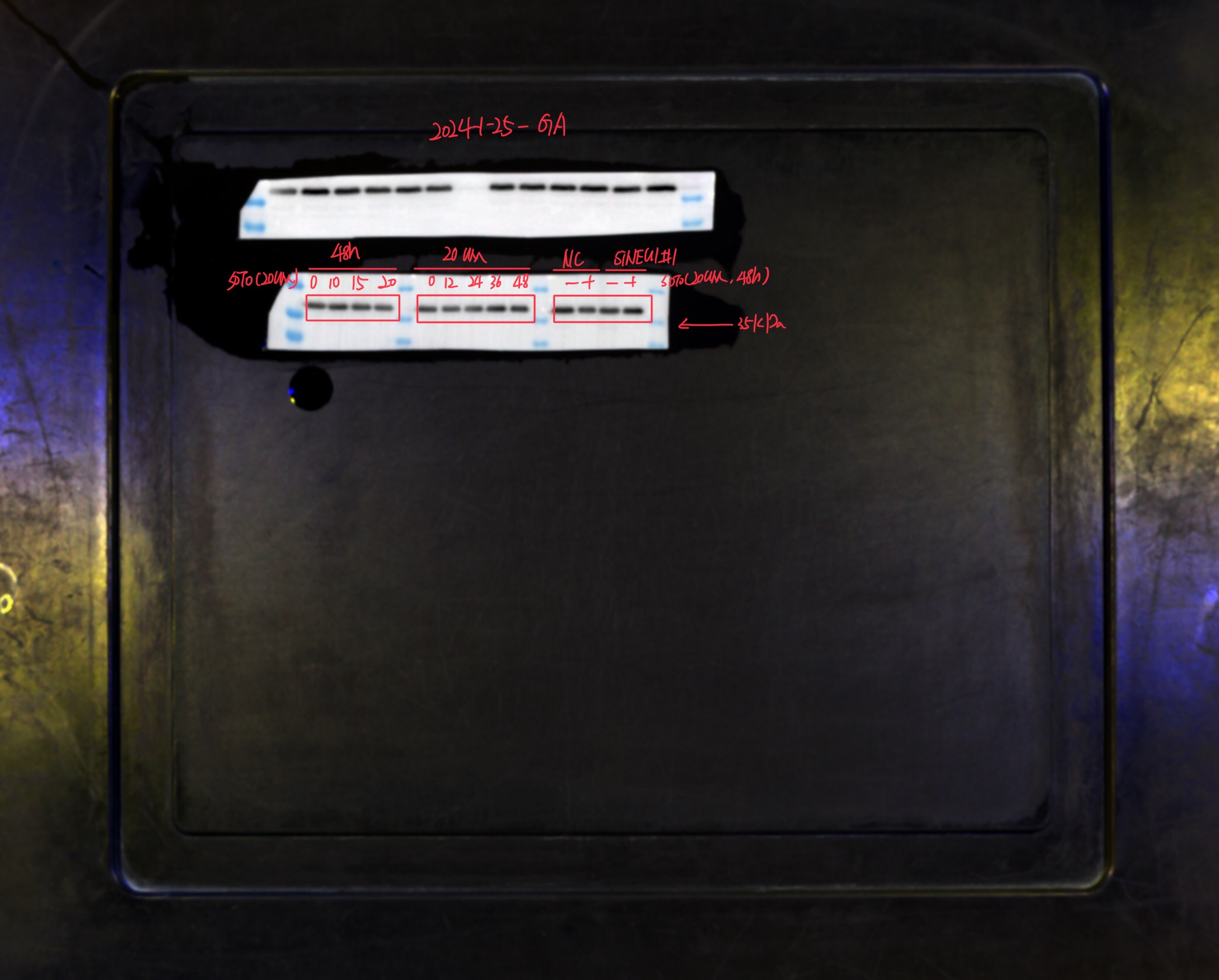

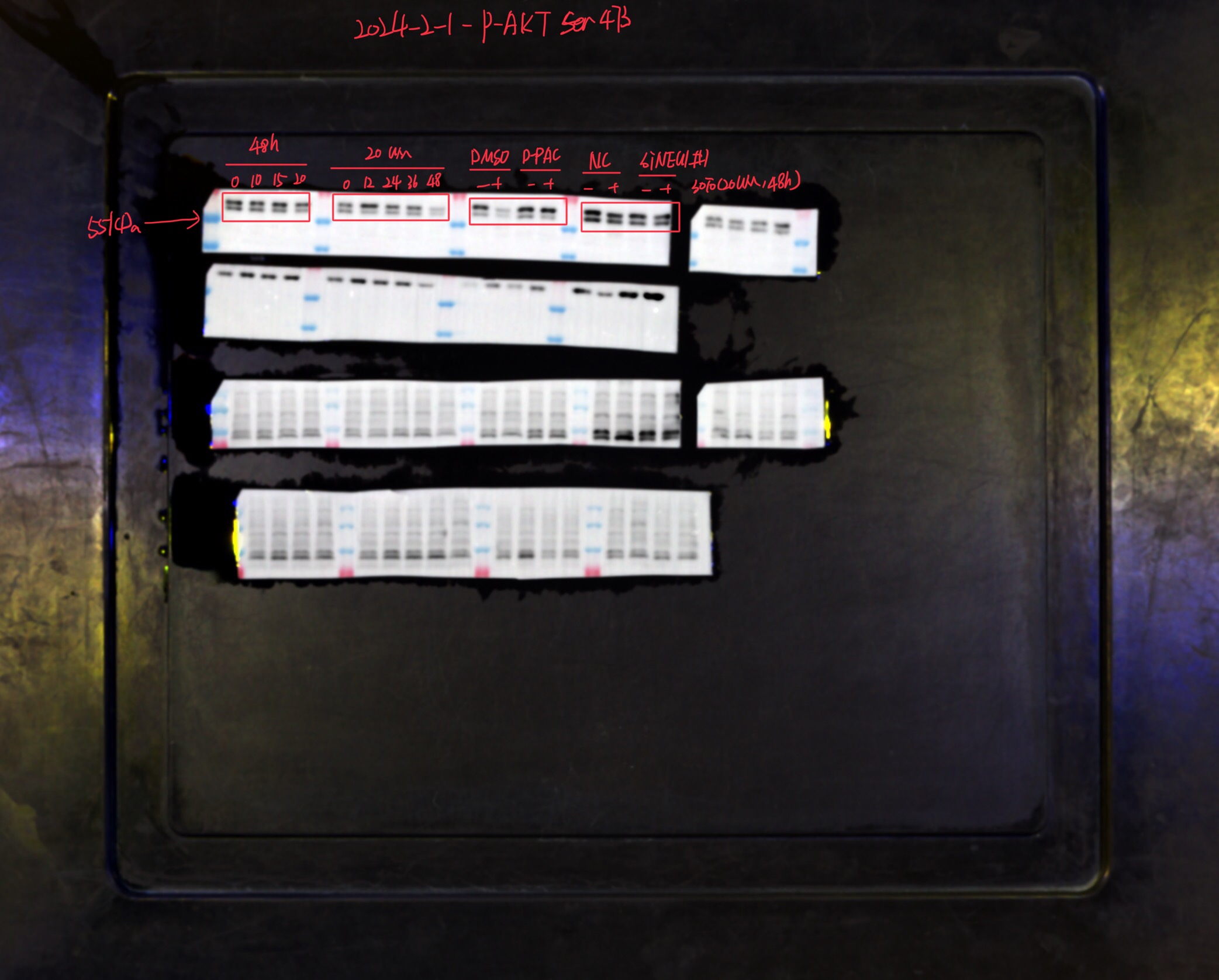

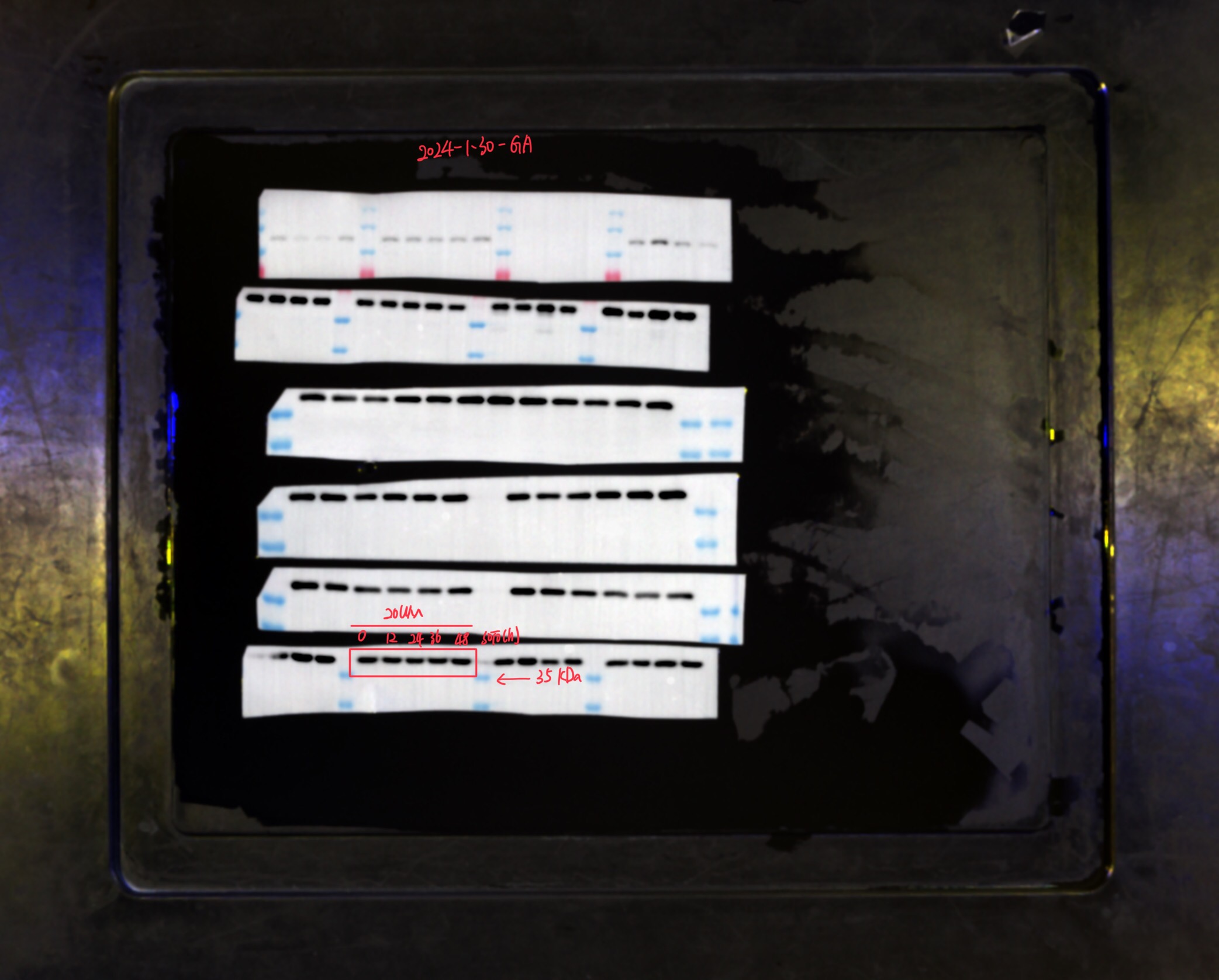


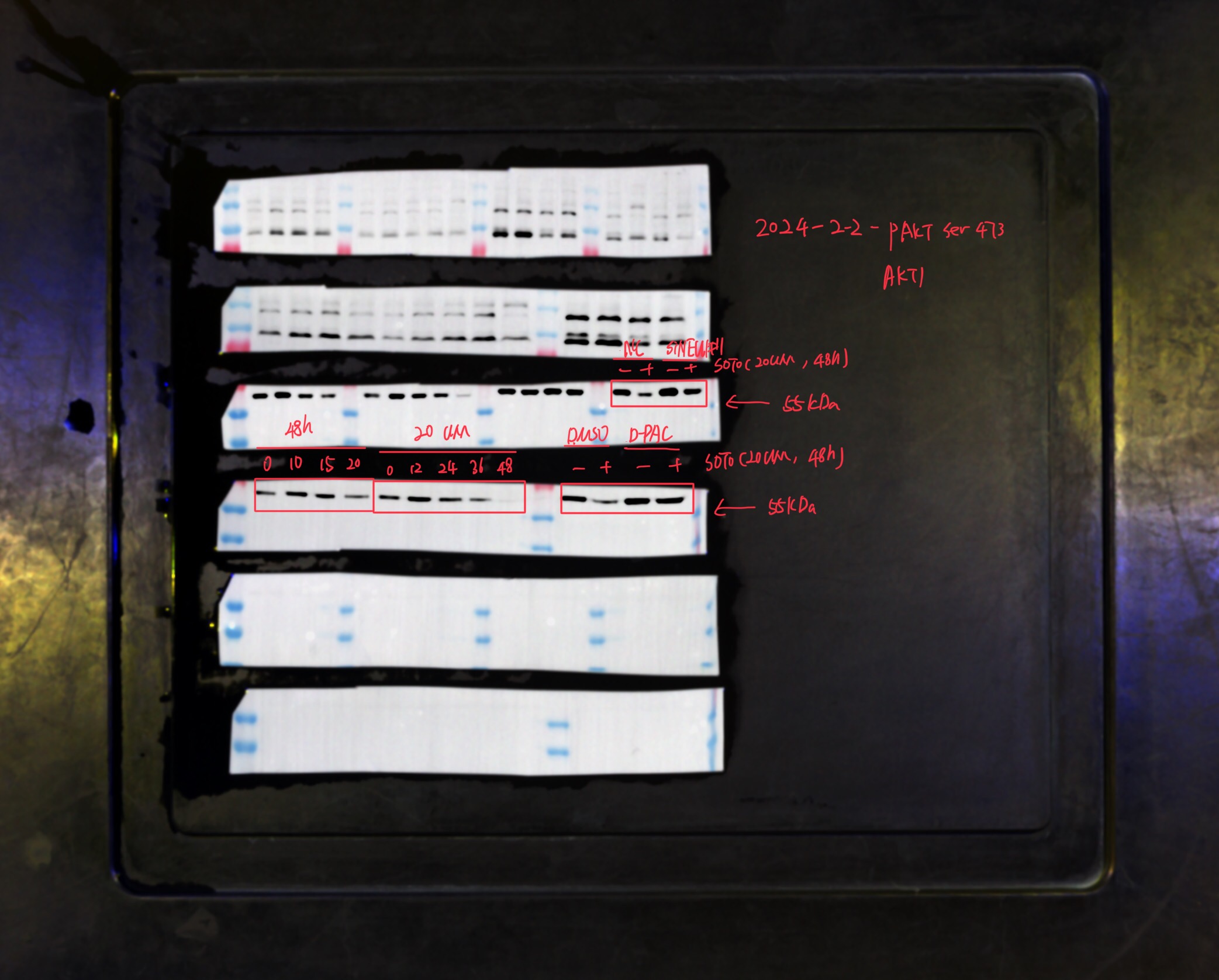

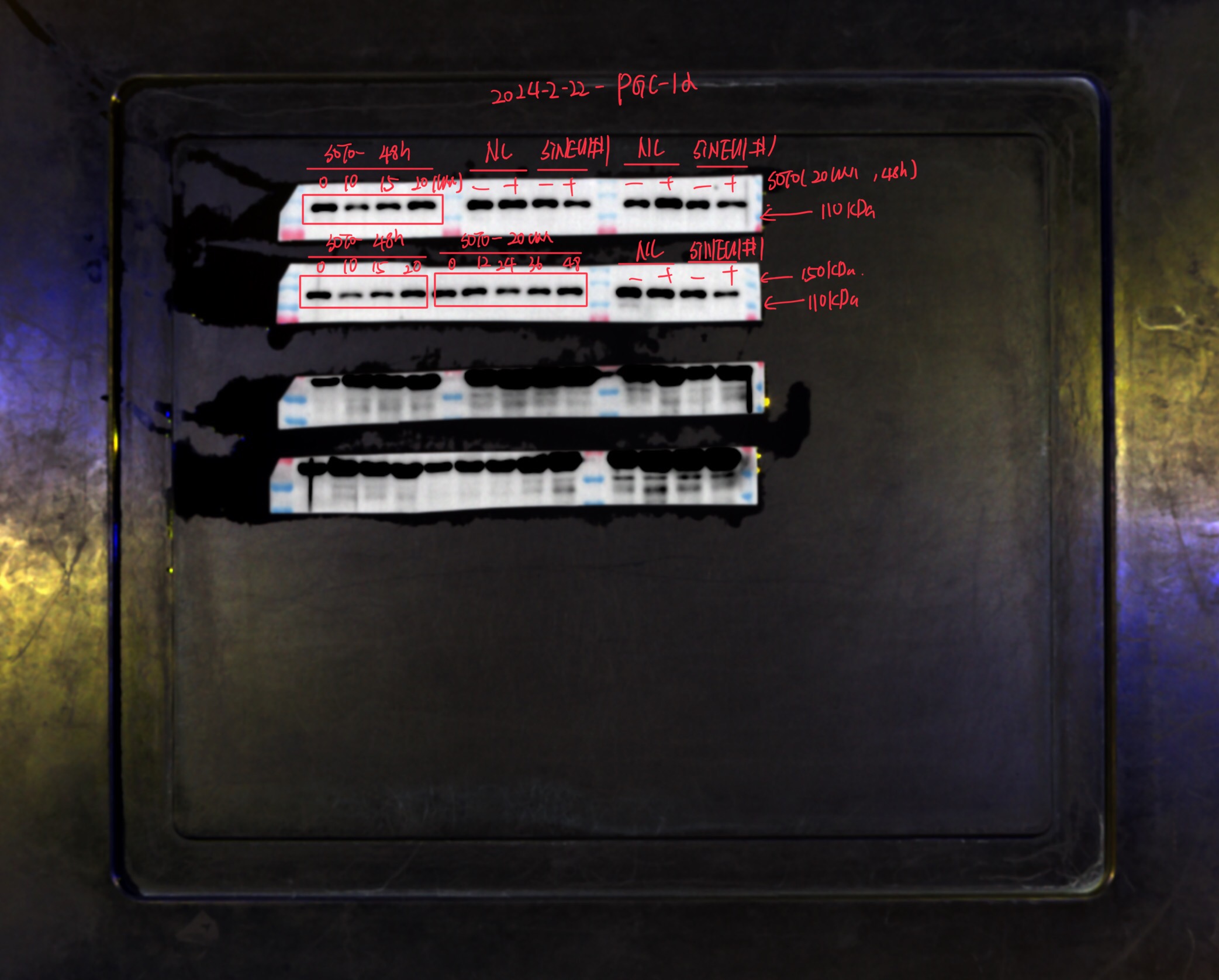


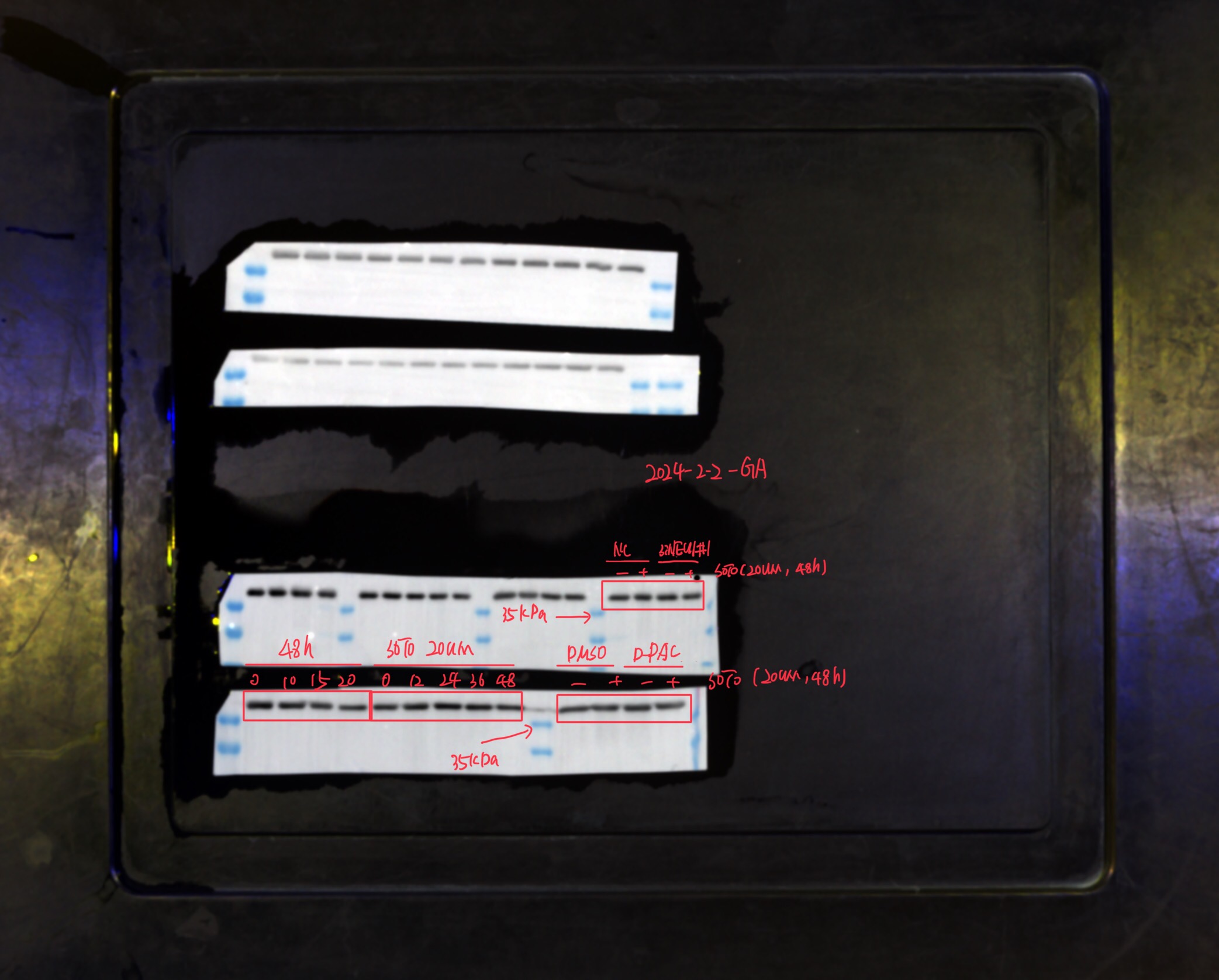

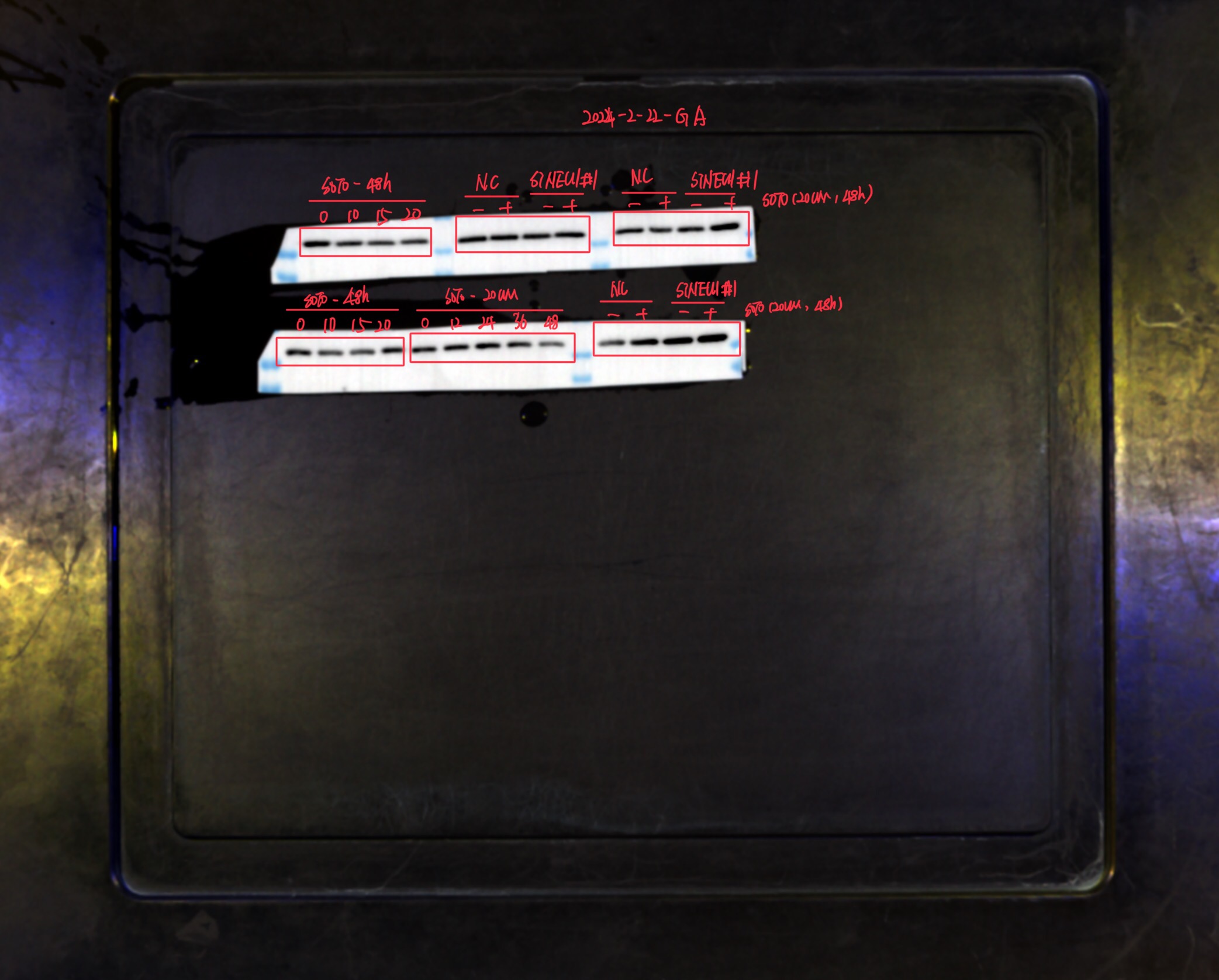


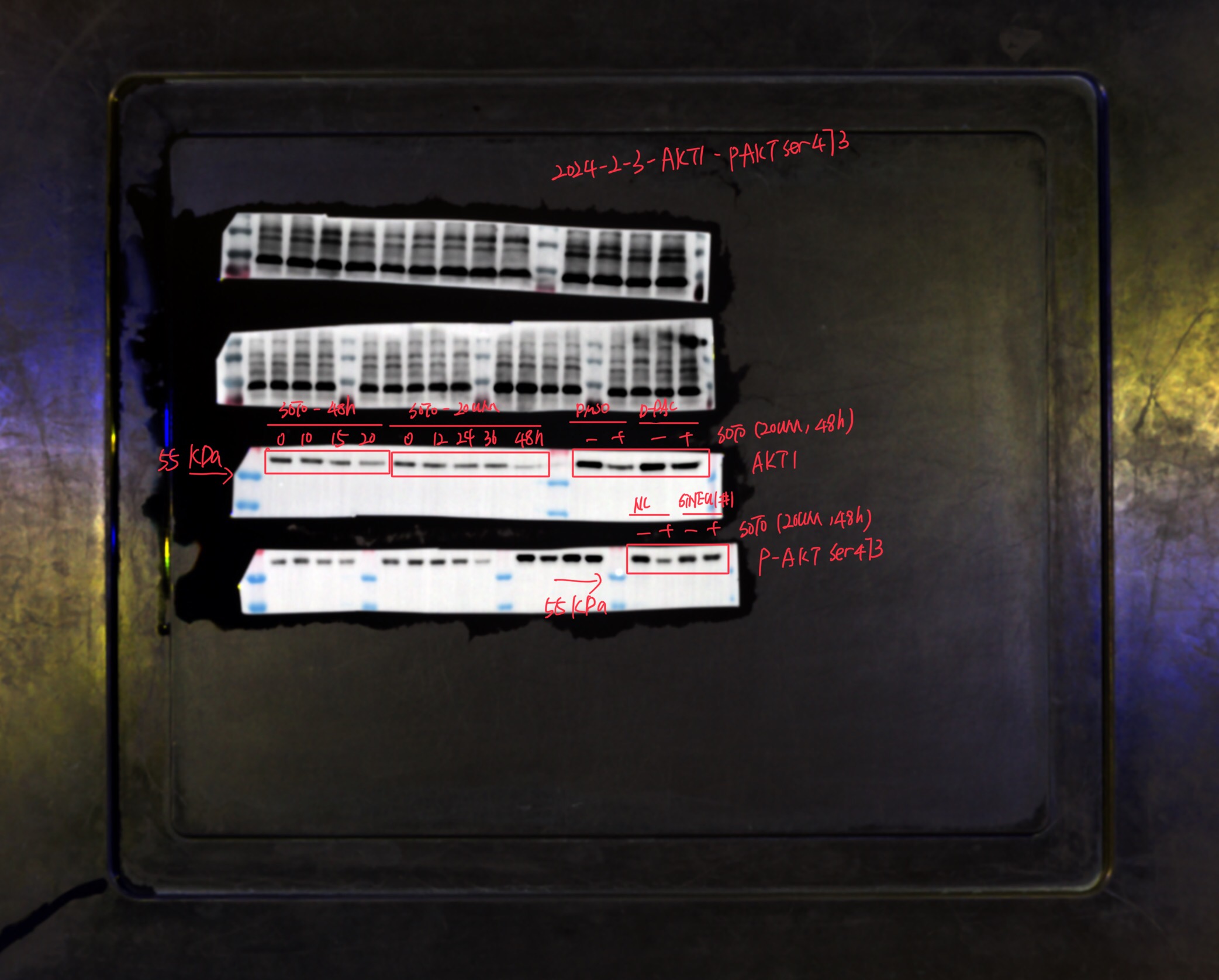


Fig. 4-F-G


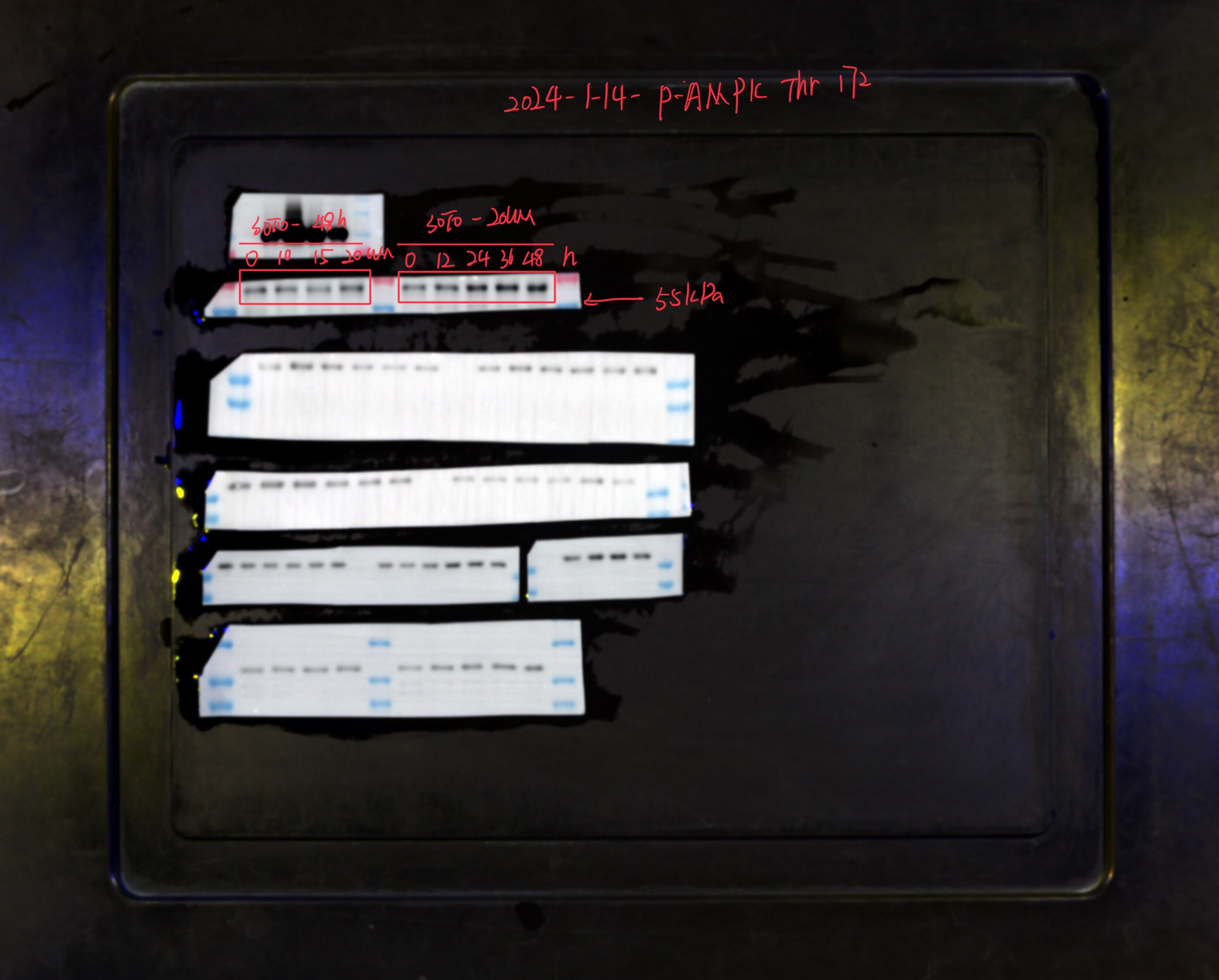

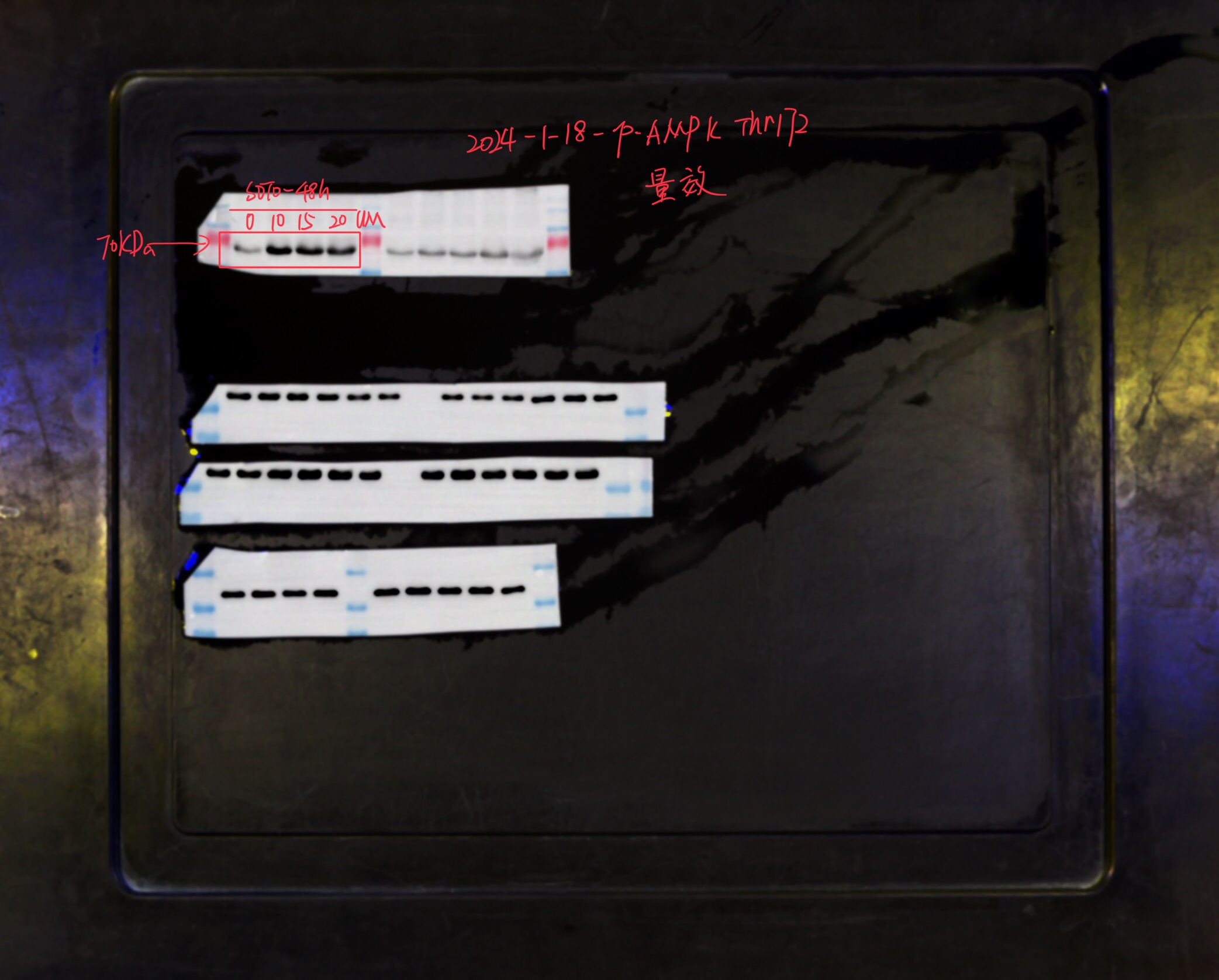

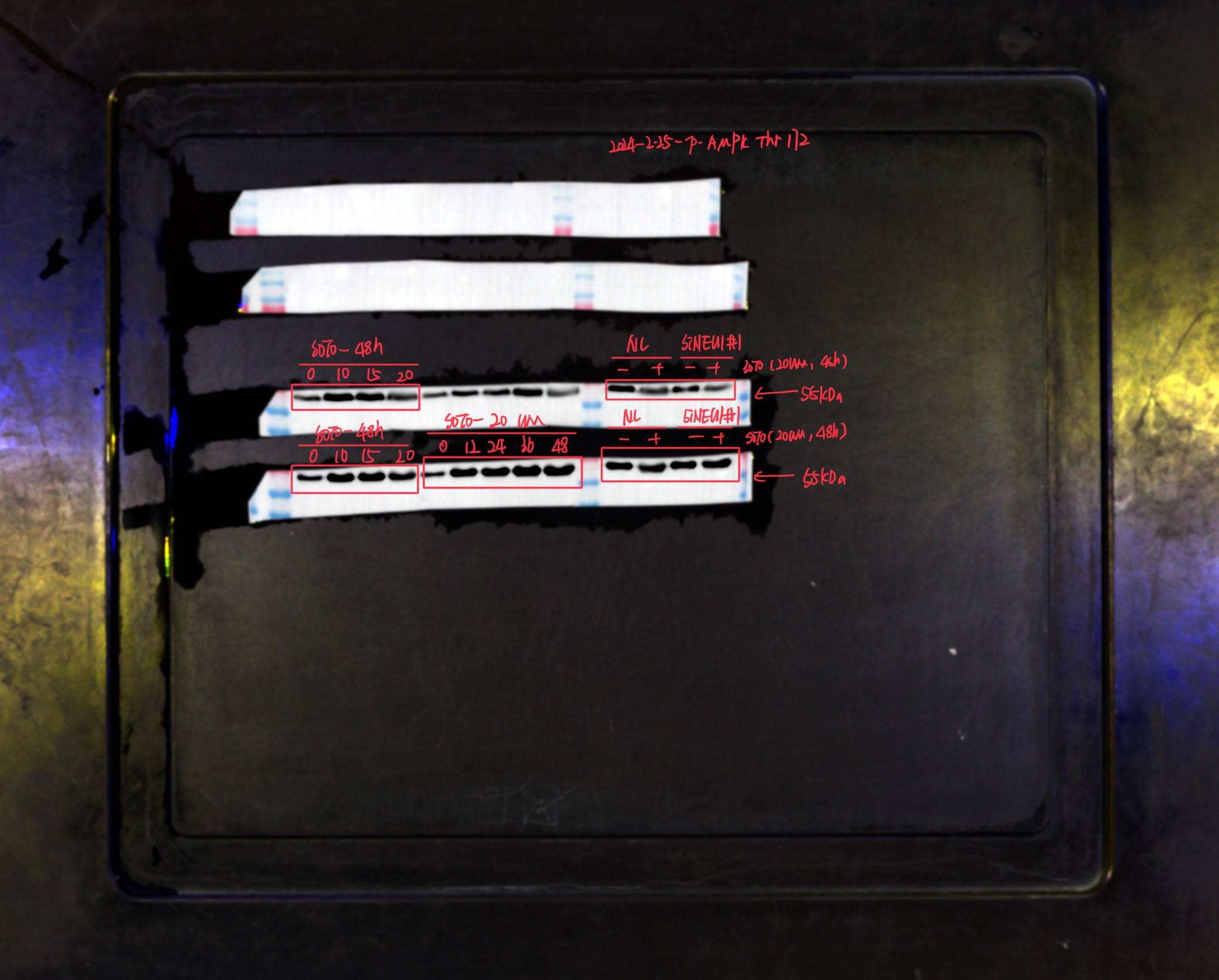


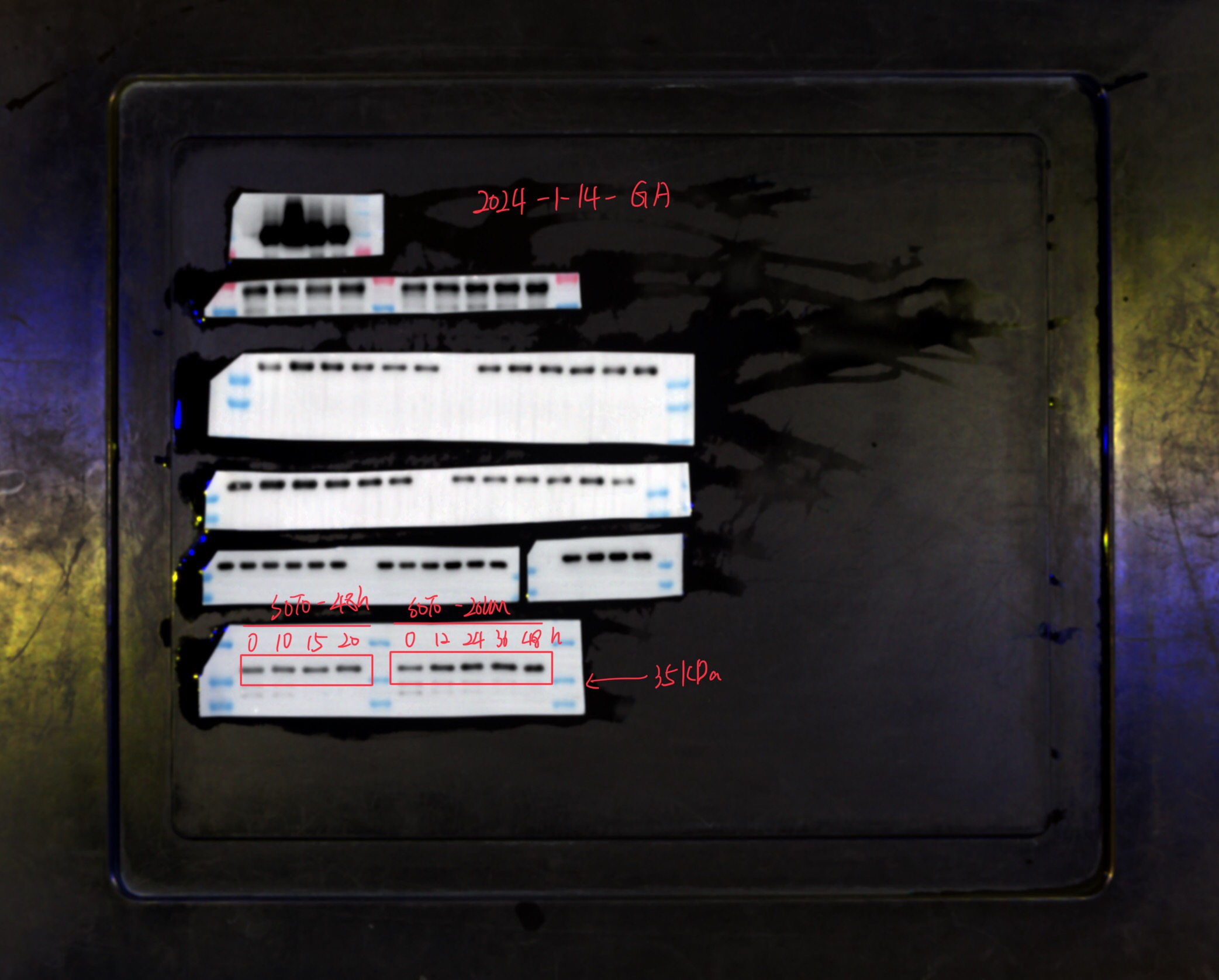

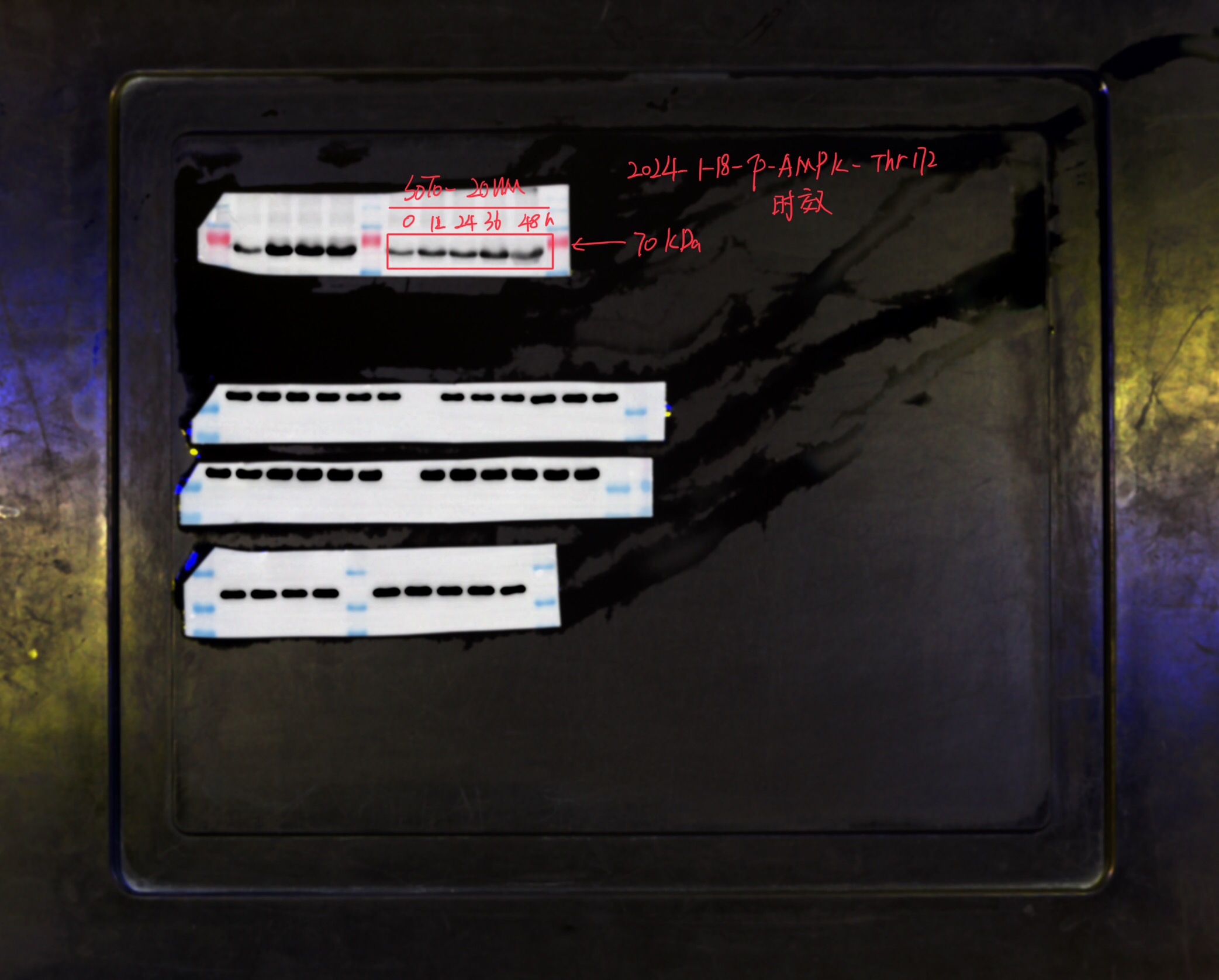

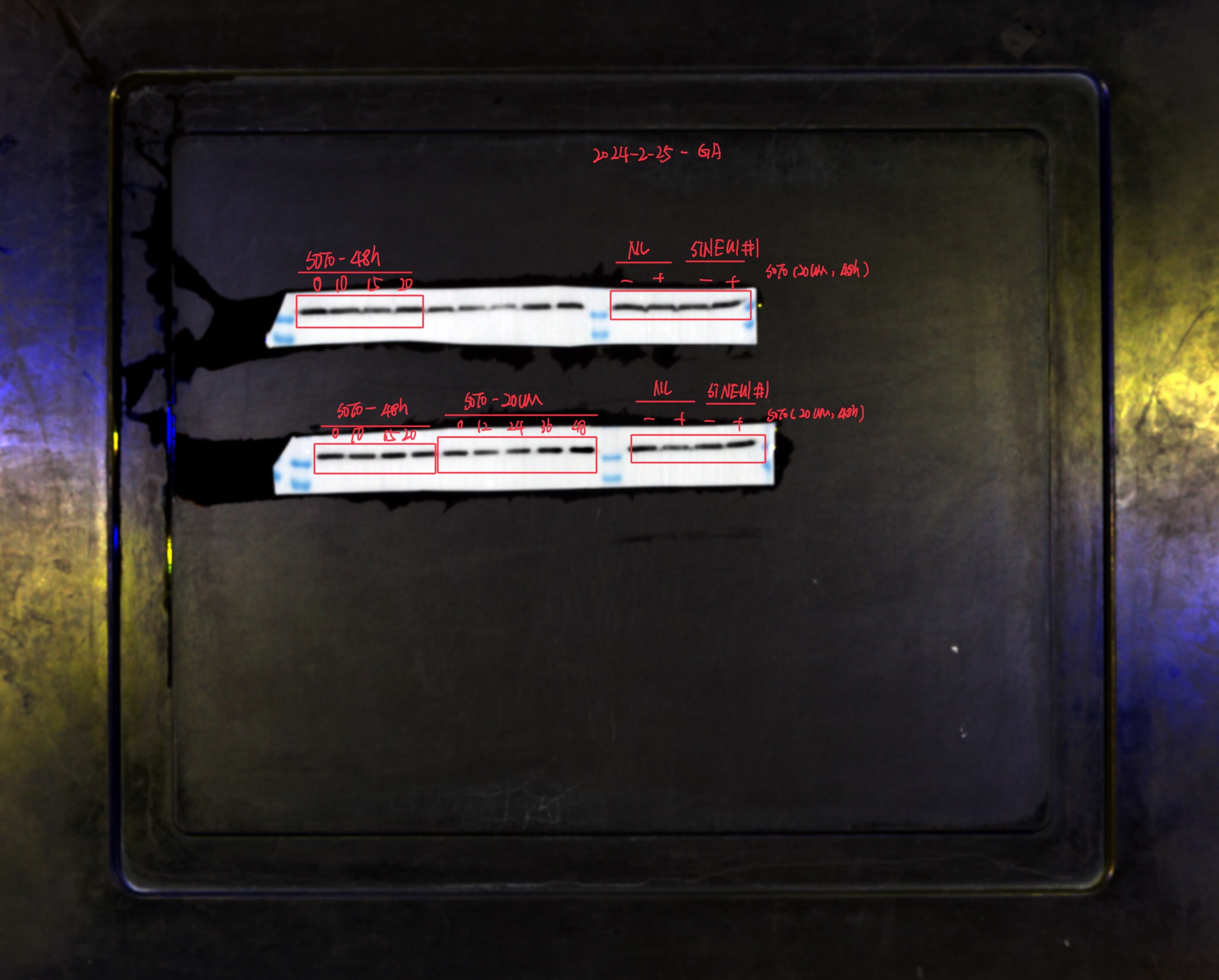


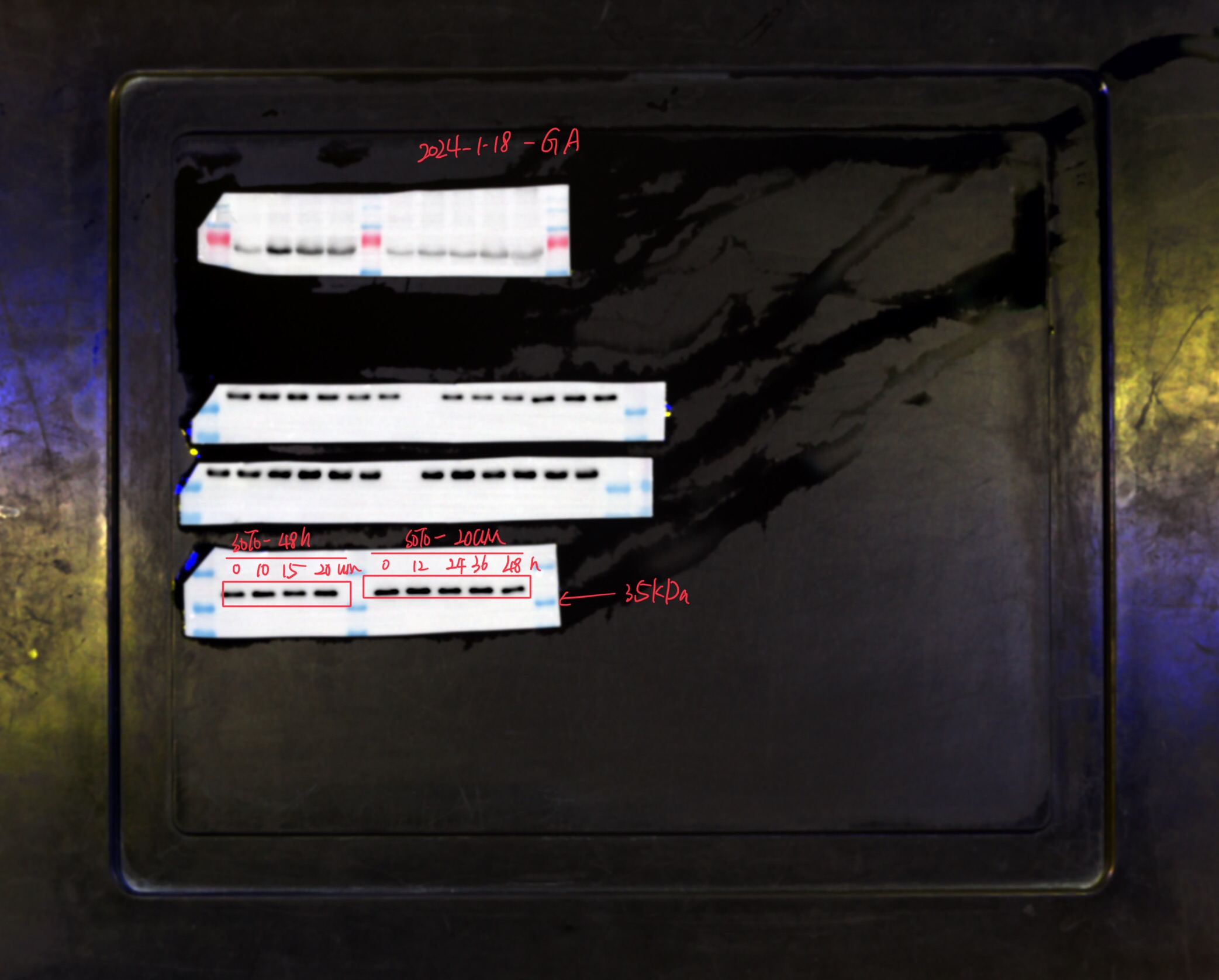


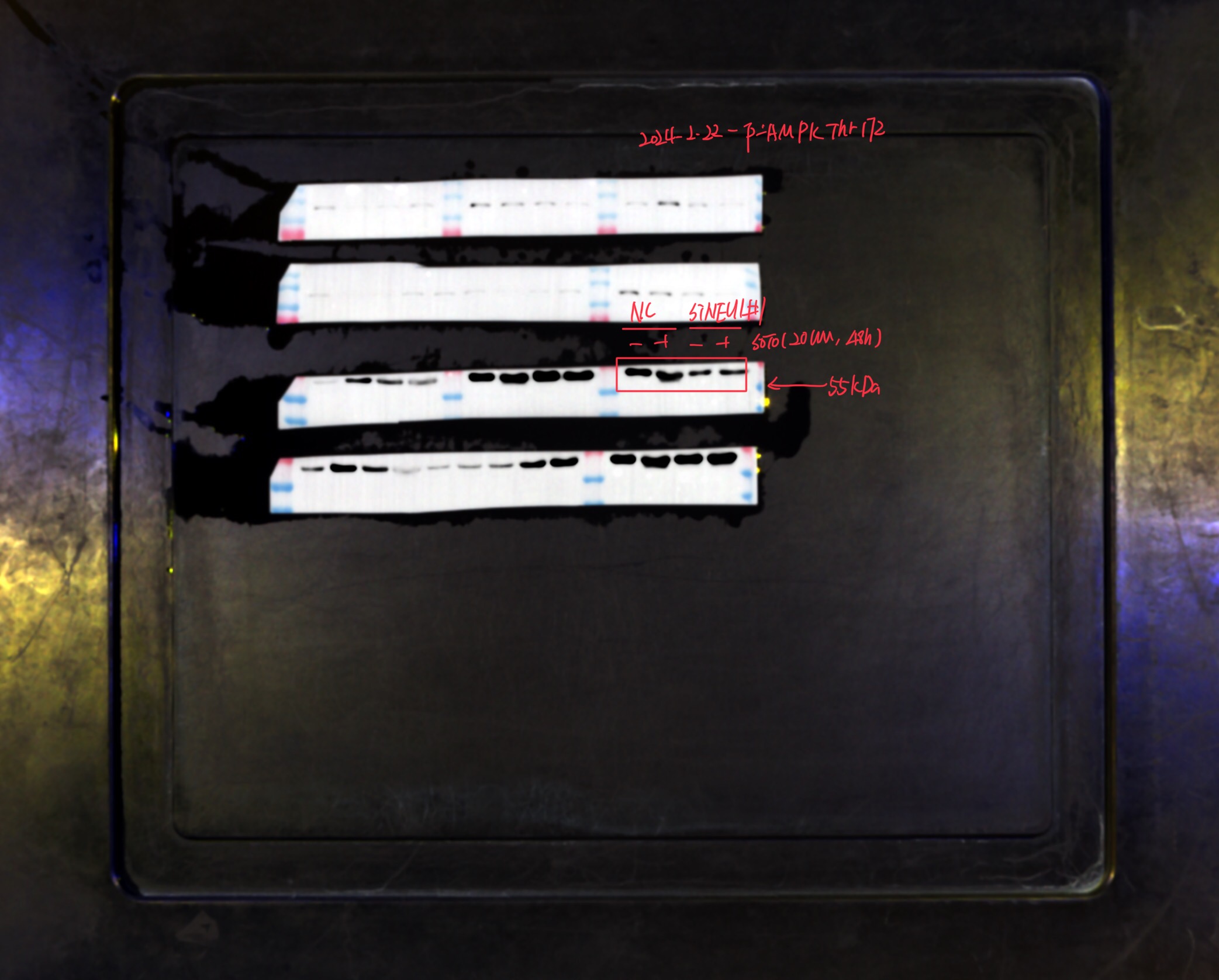

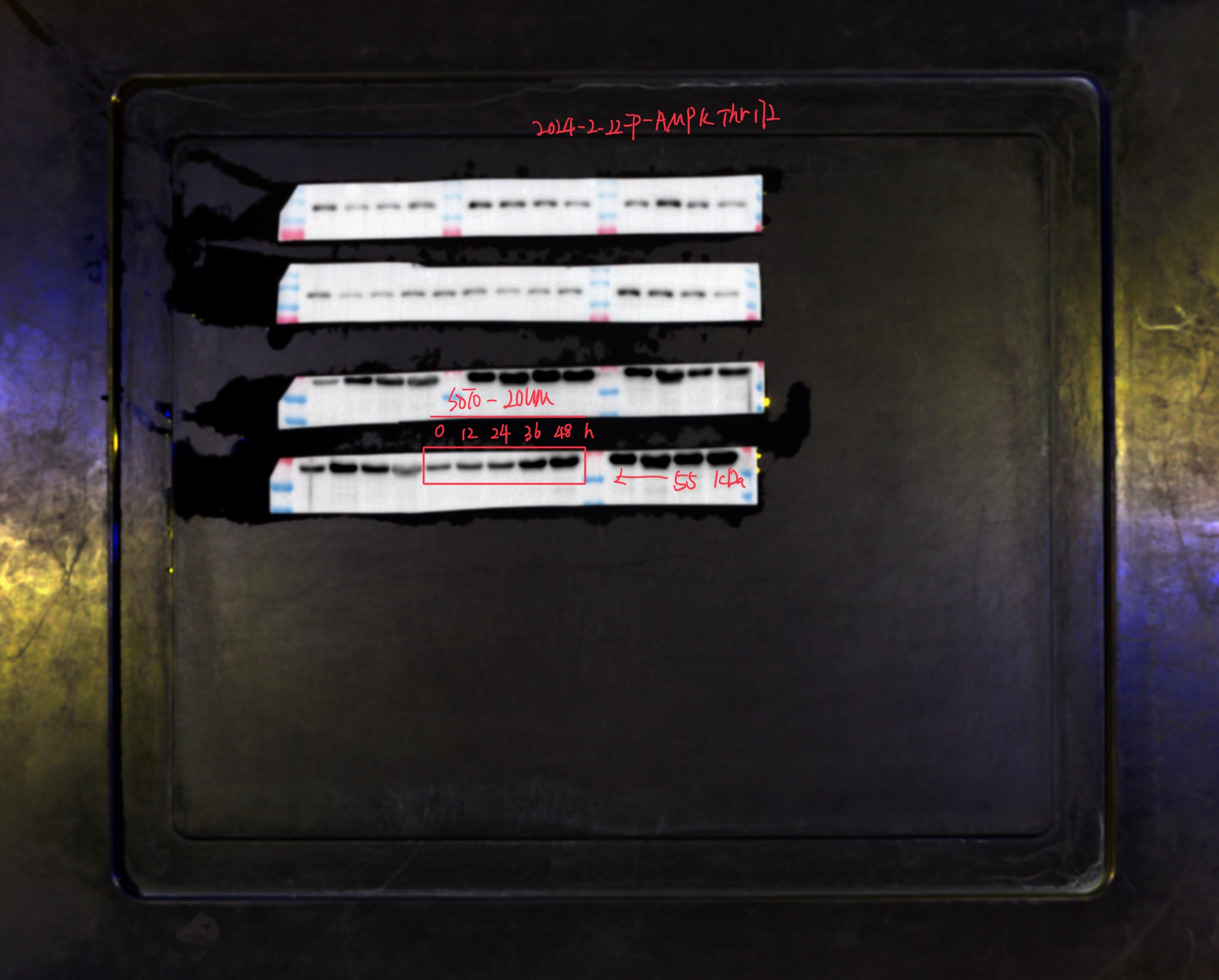

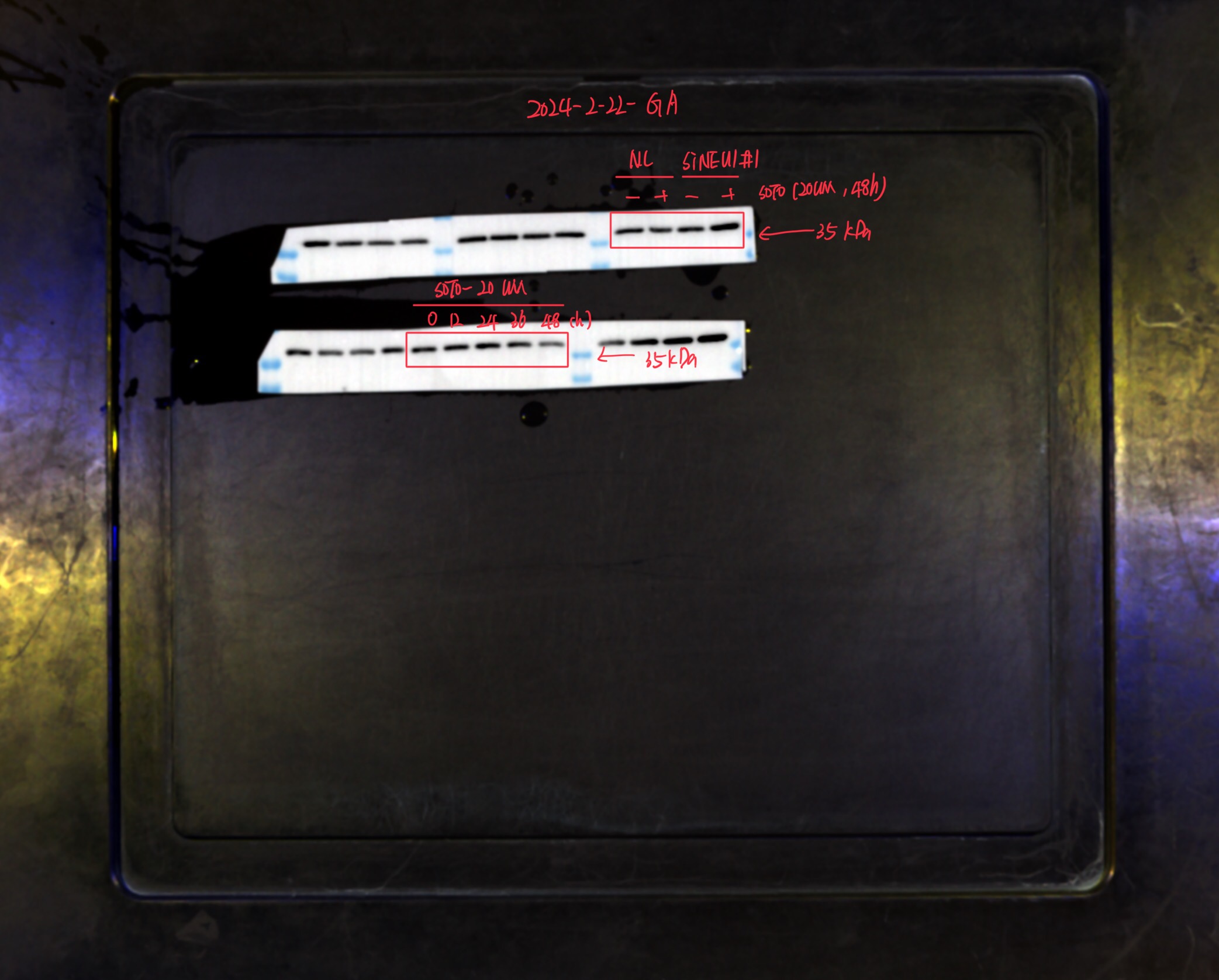


Fig. 5-B


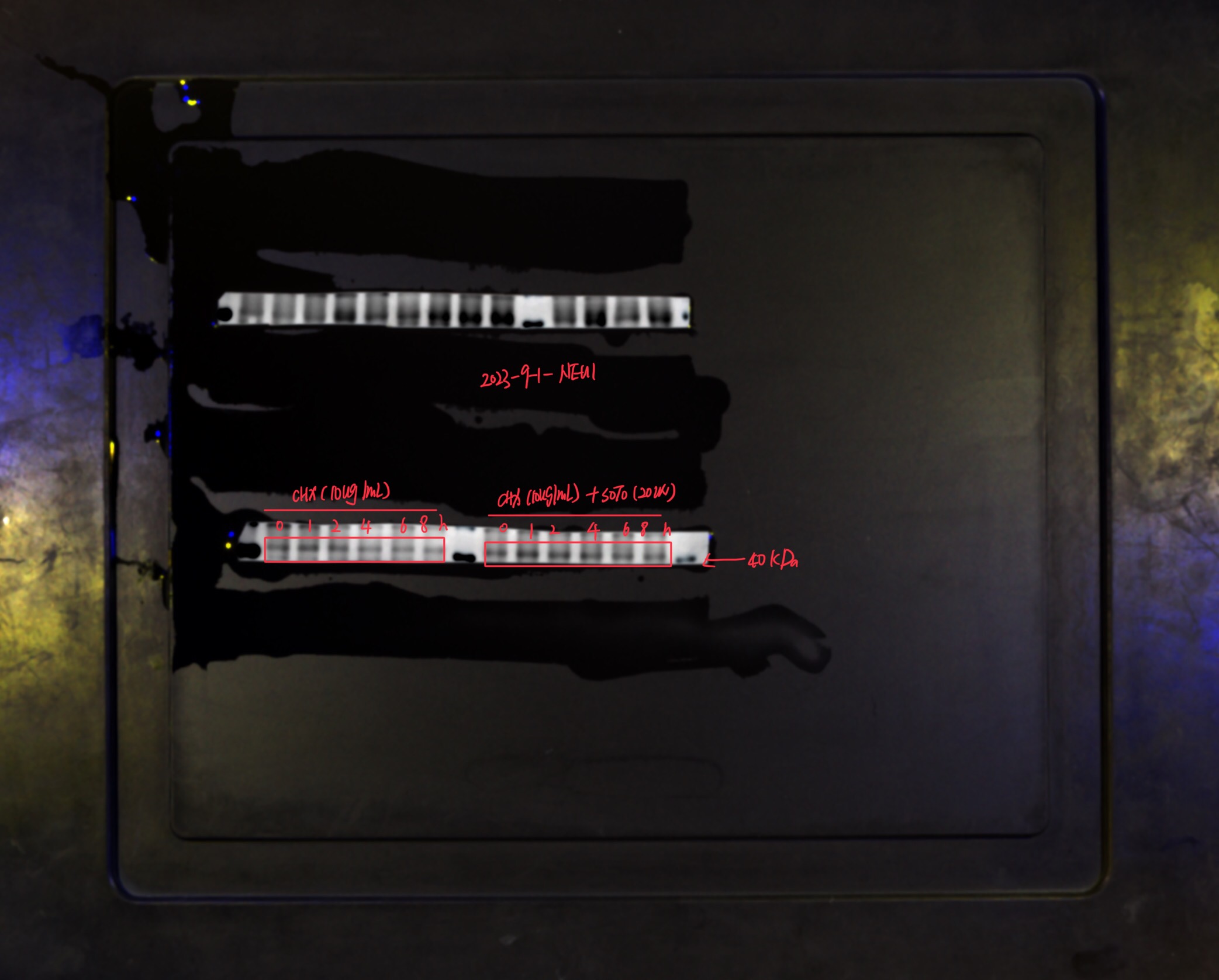

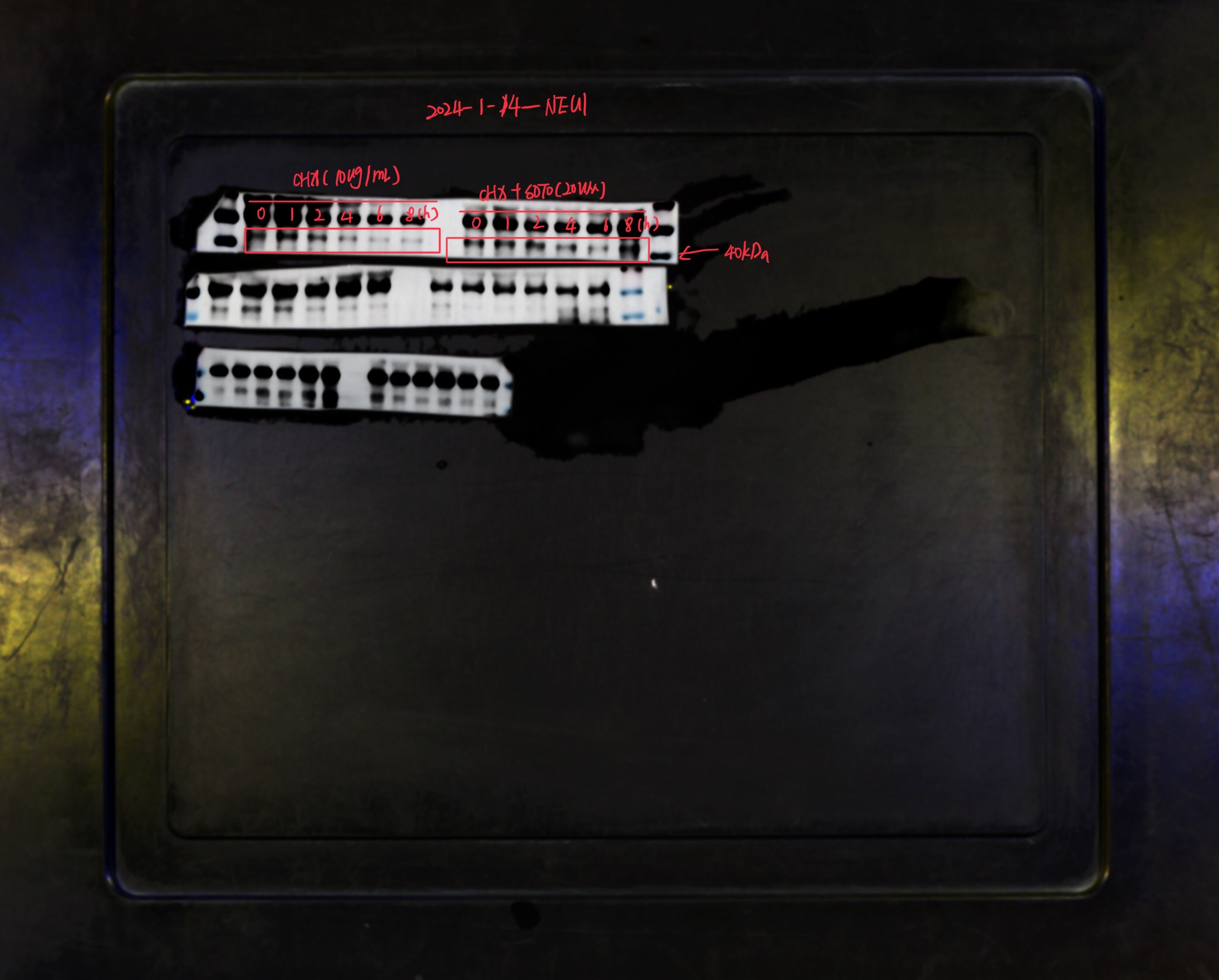

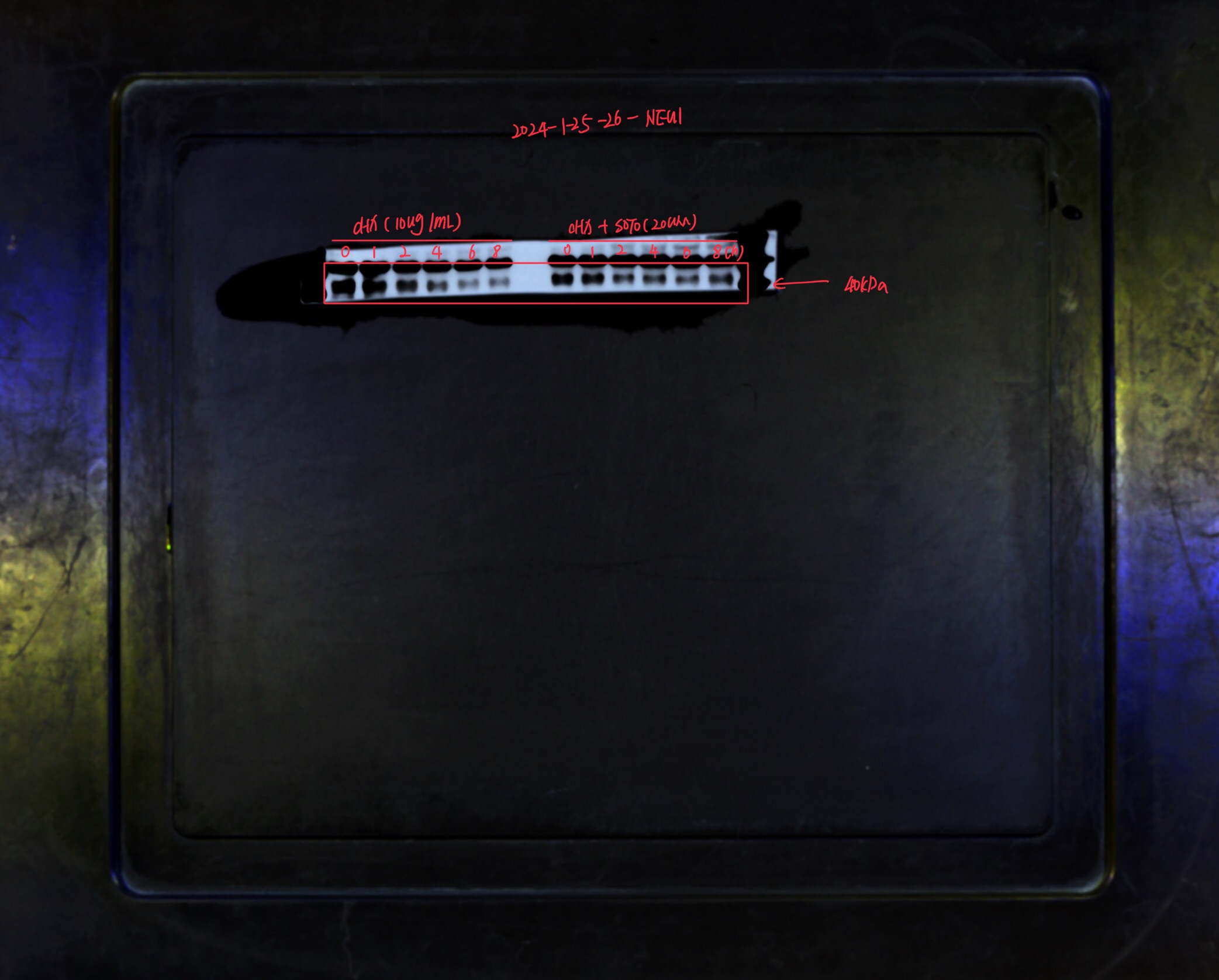


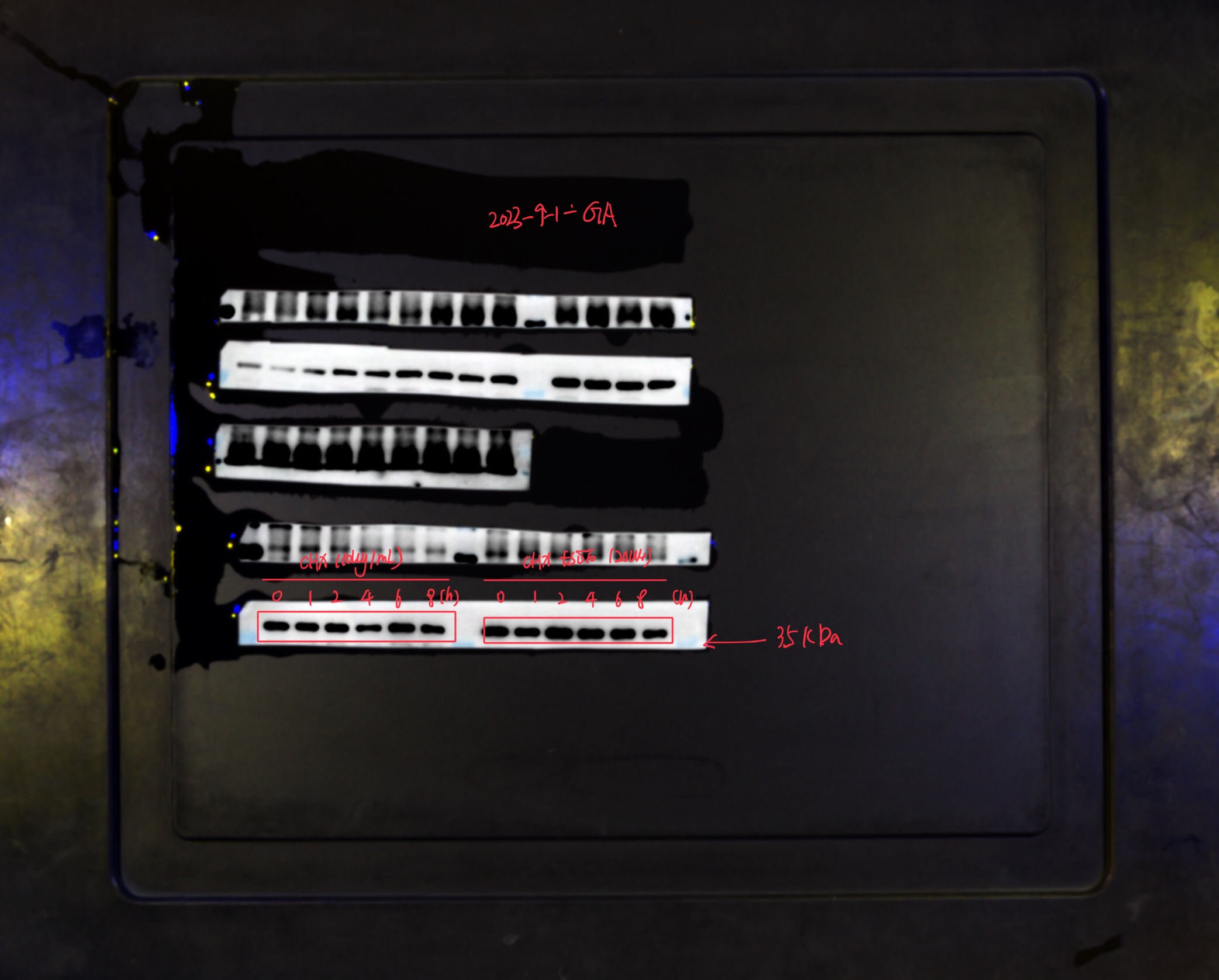

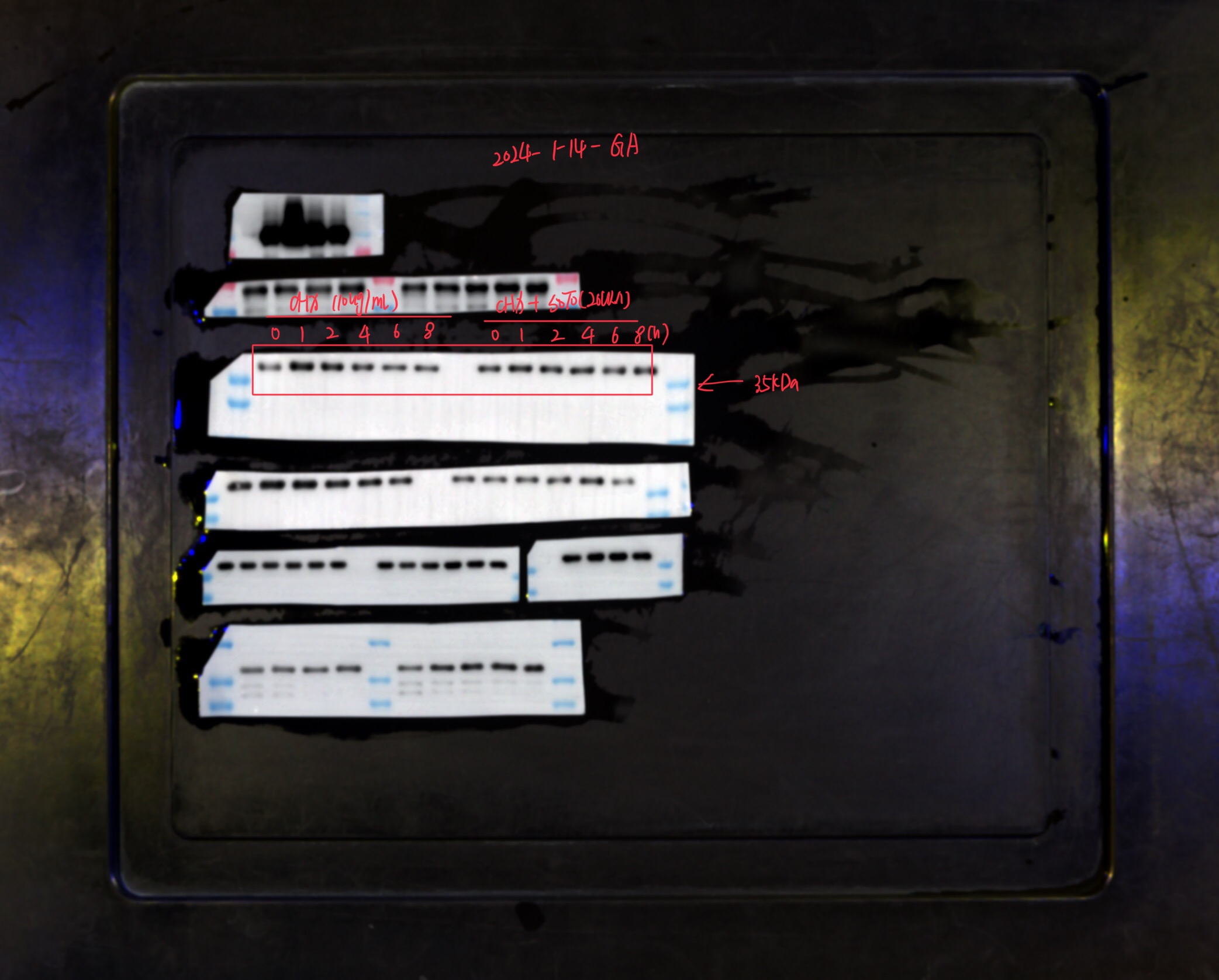

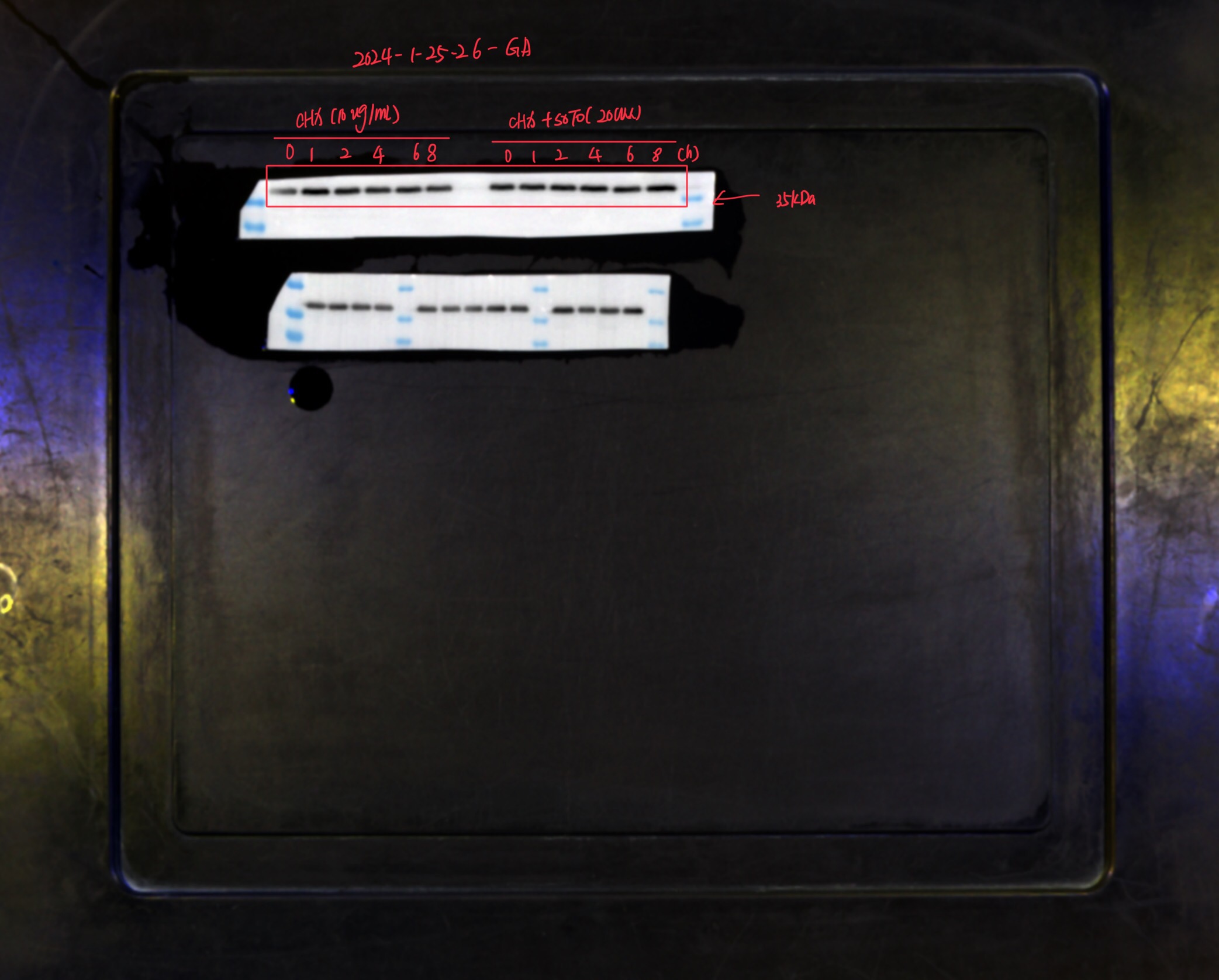


Fig. 5-C


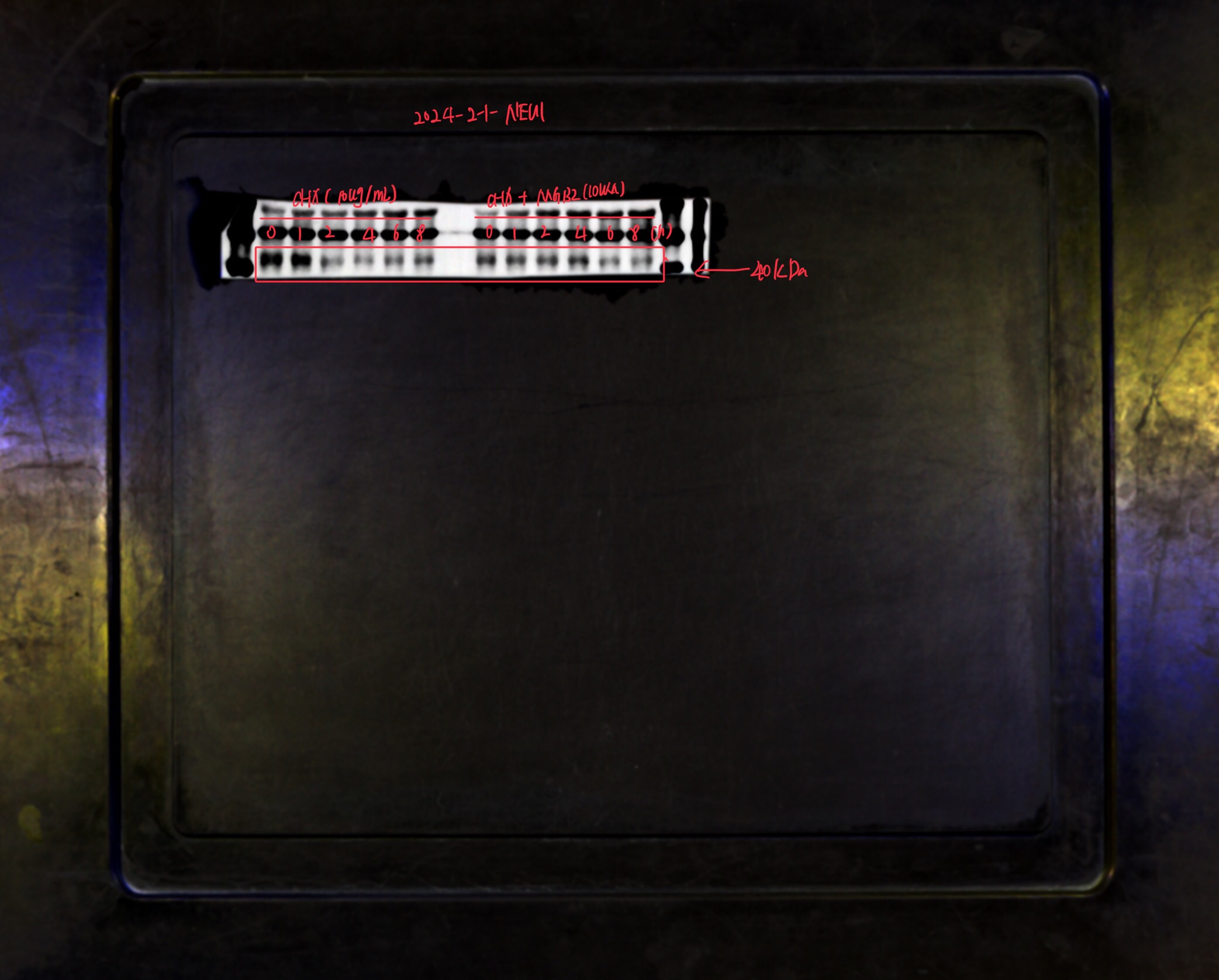

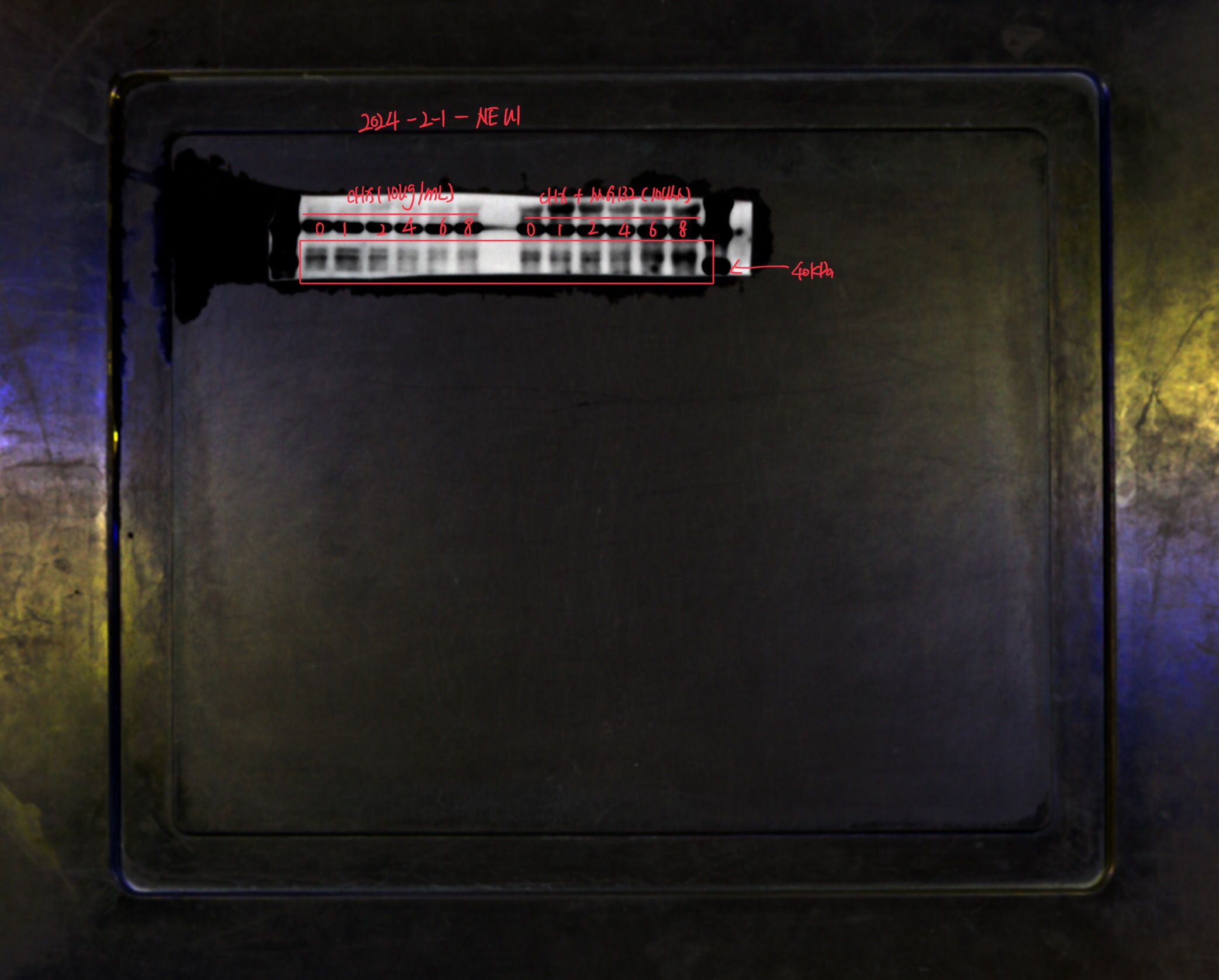

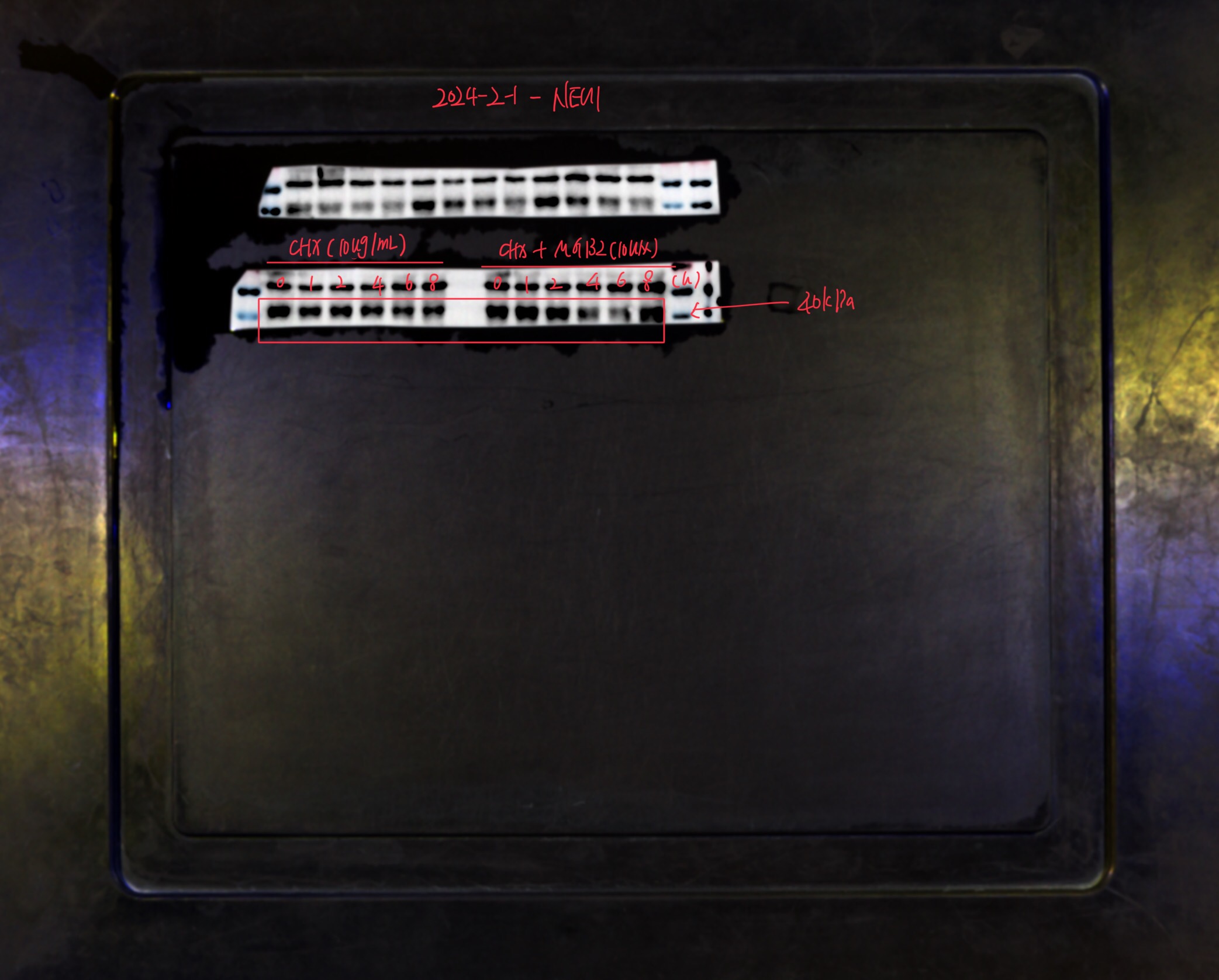


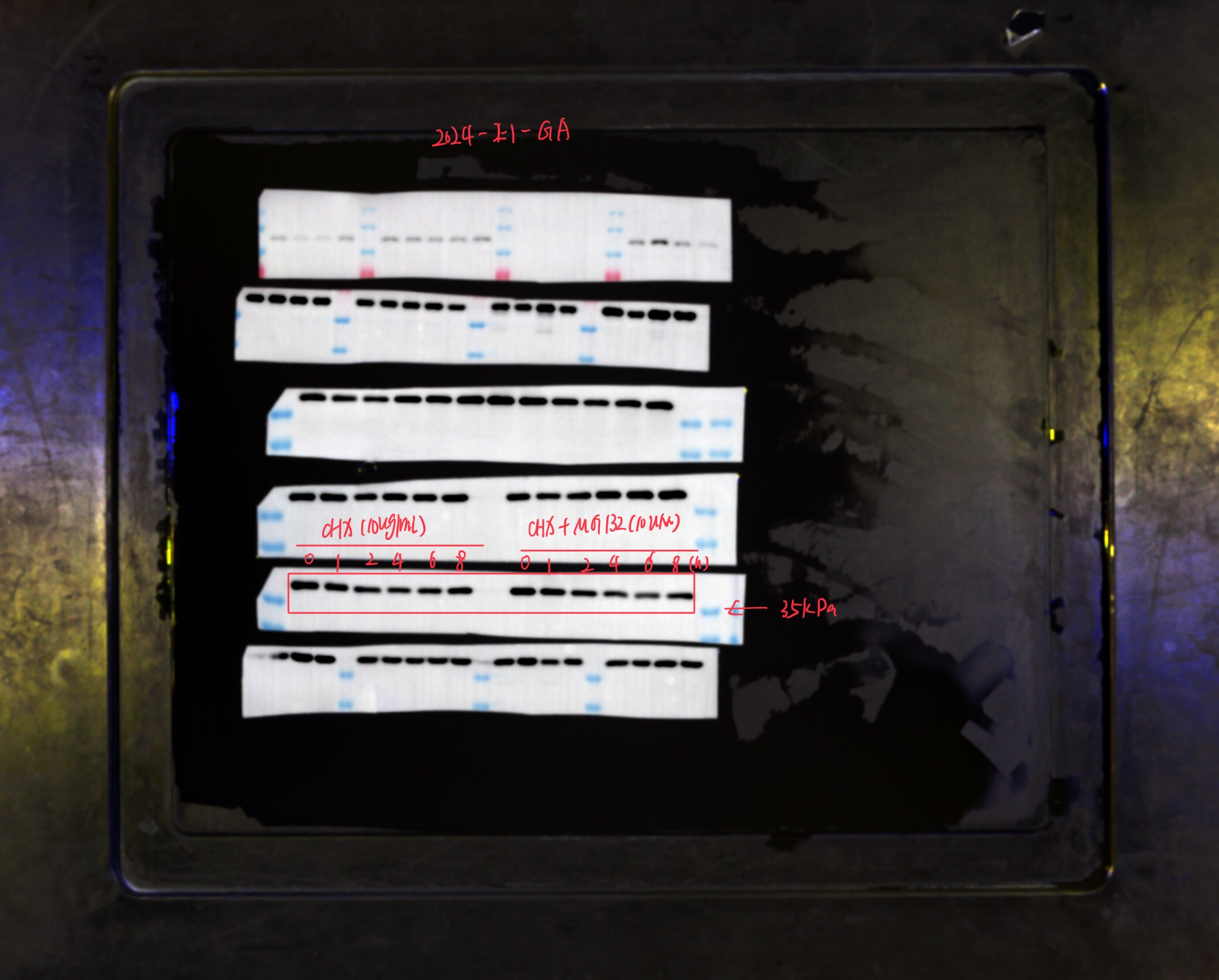

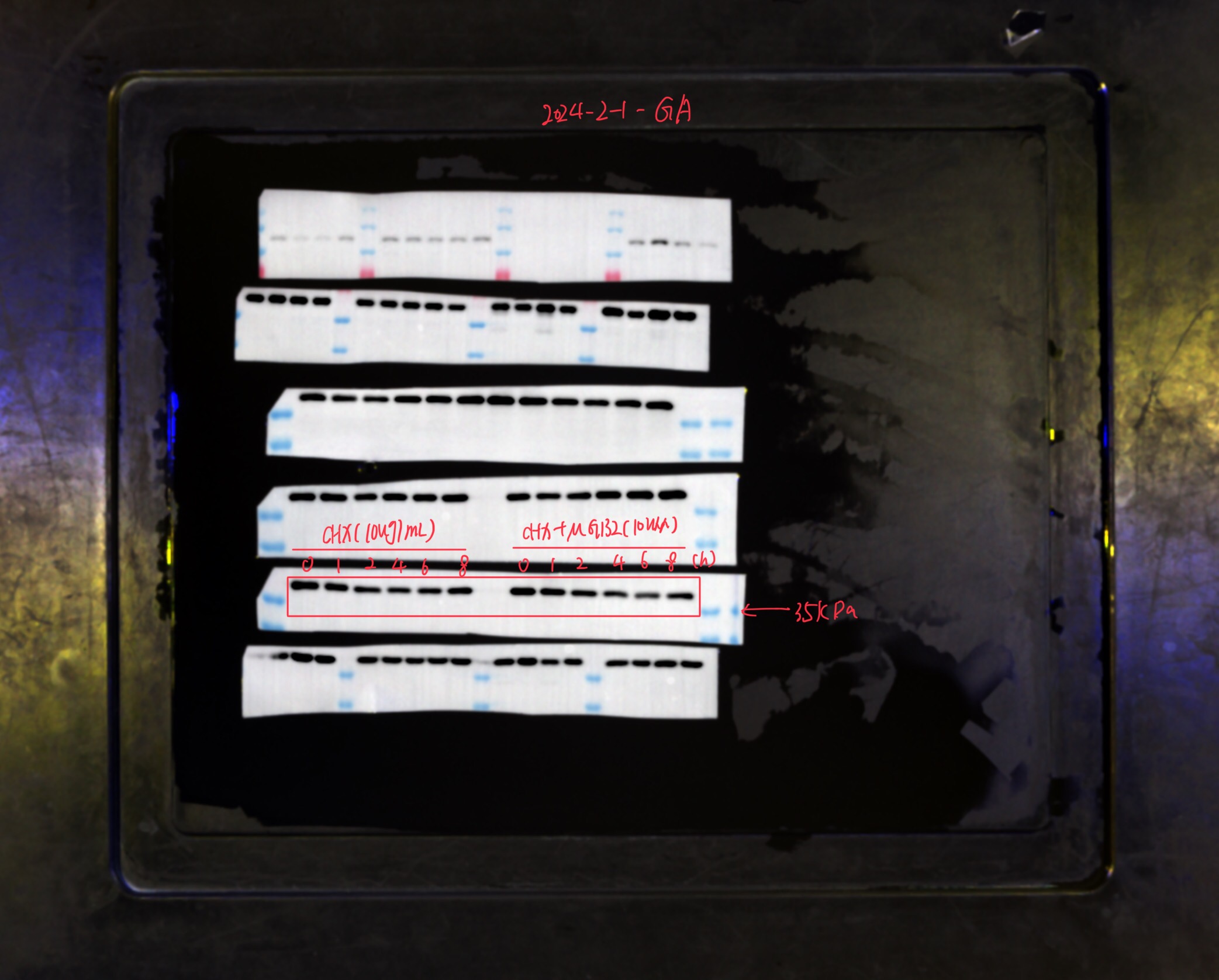

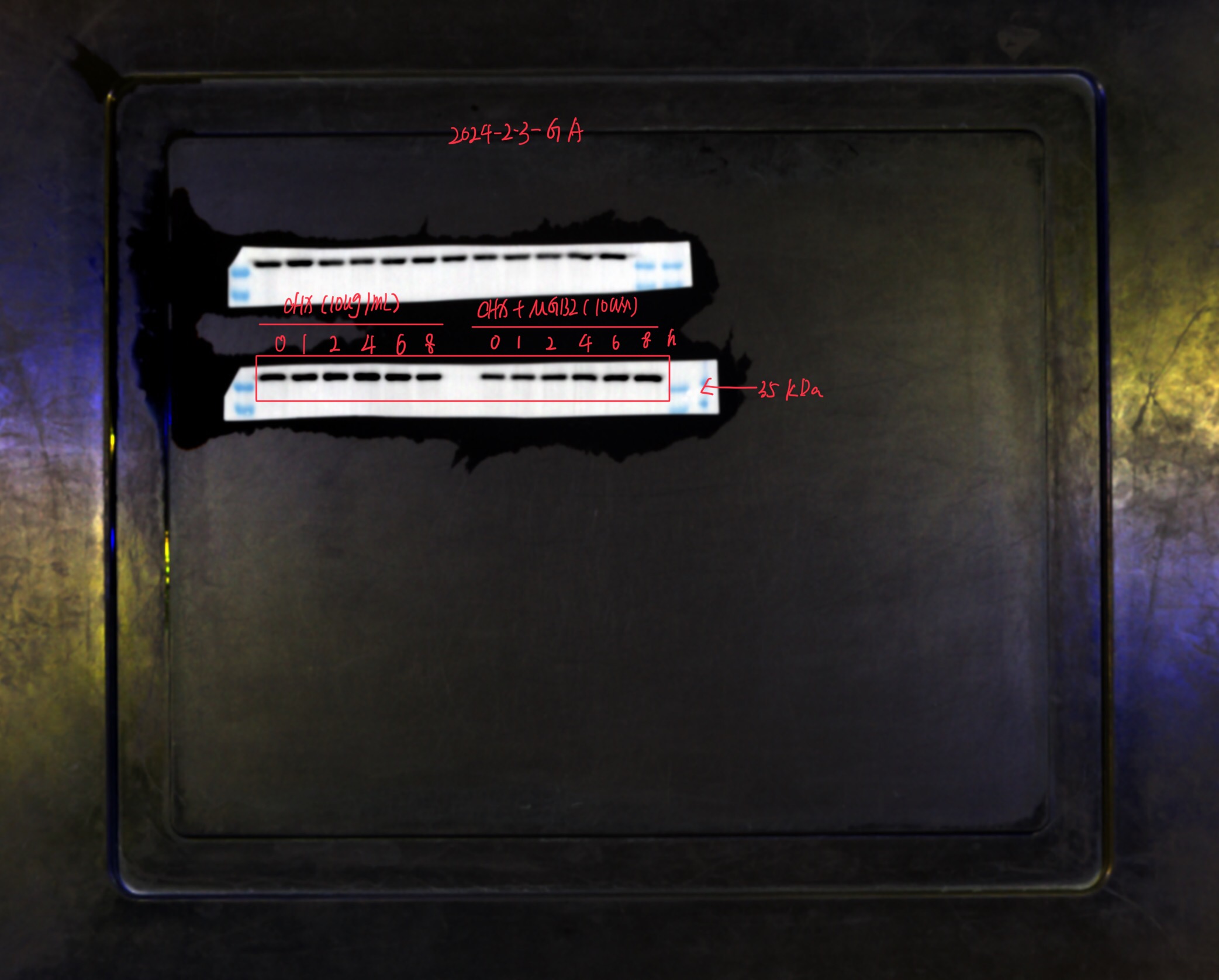


Fig. 5-D


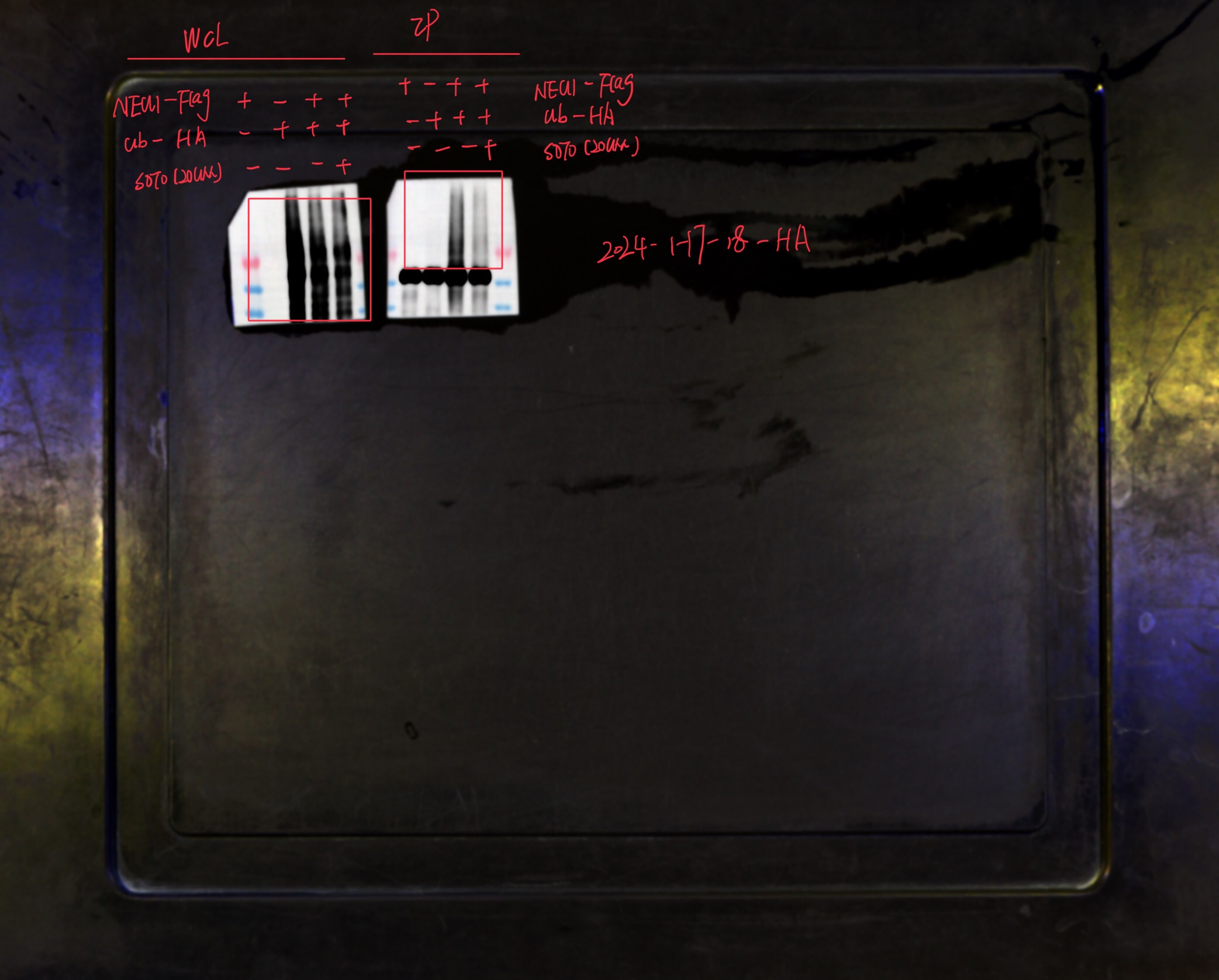


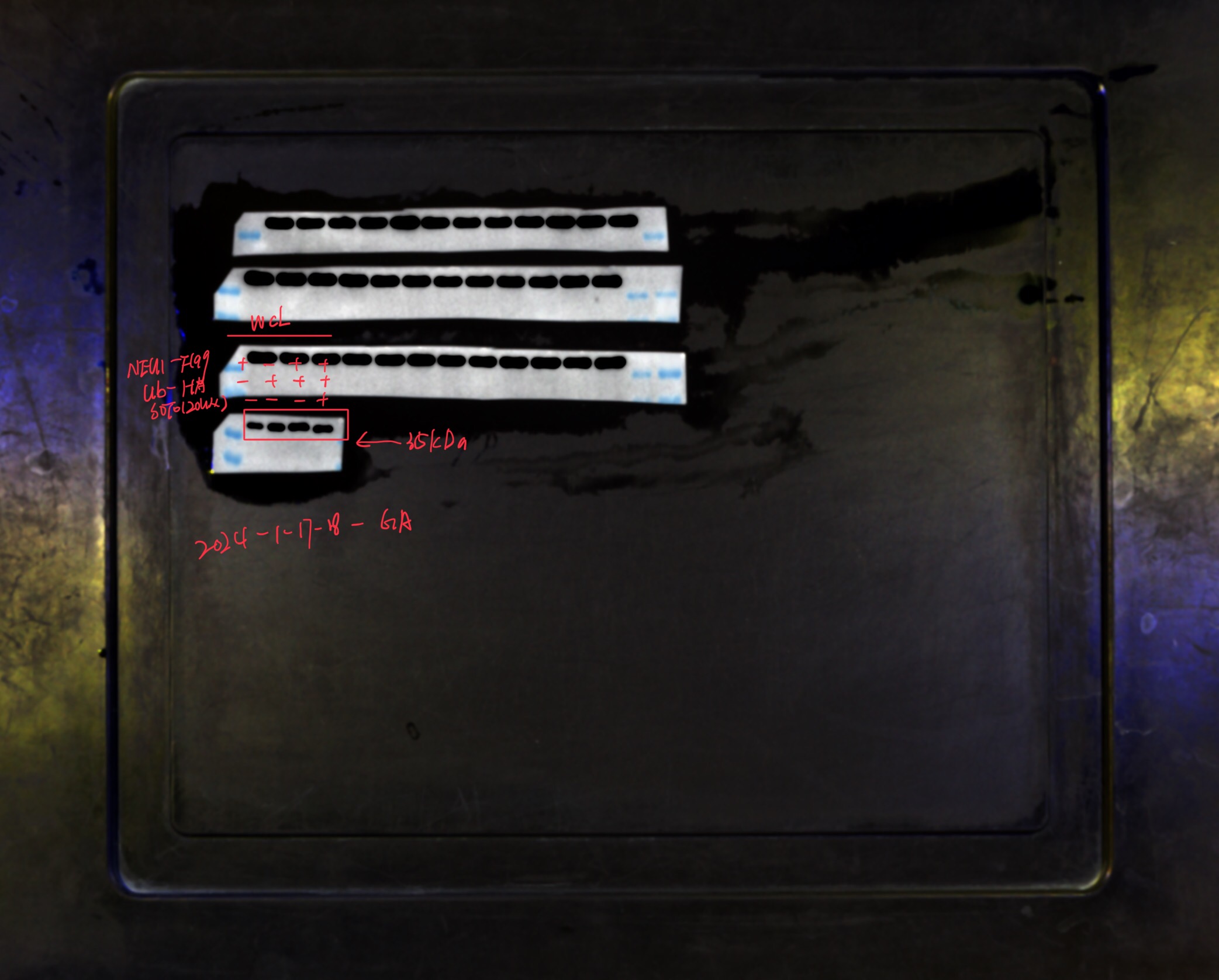


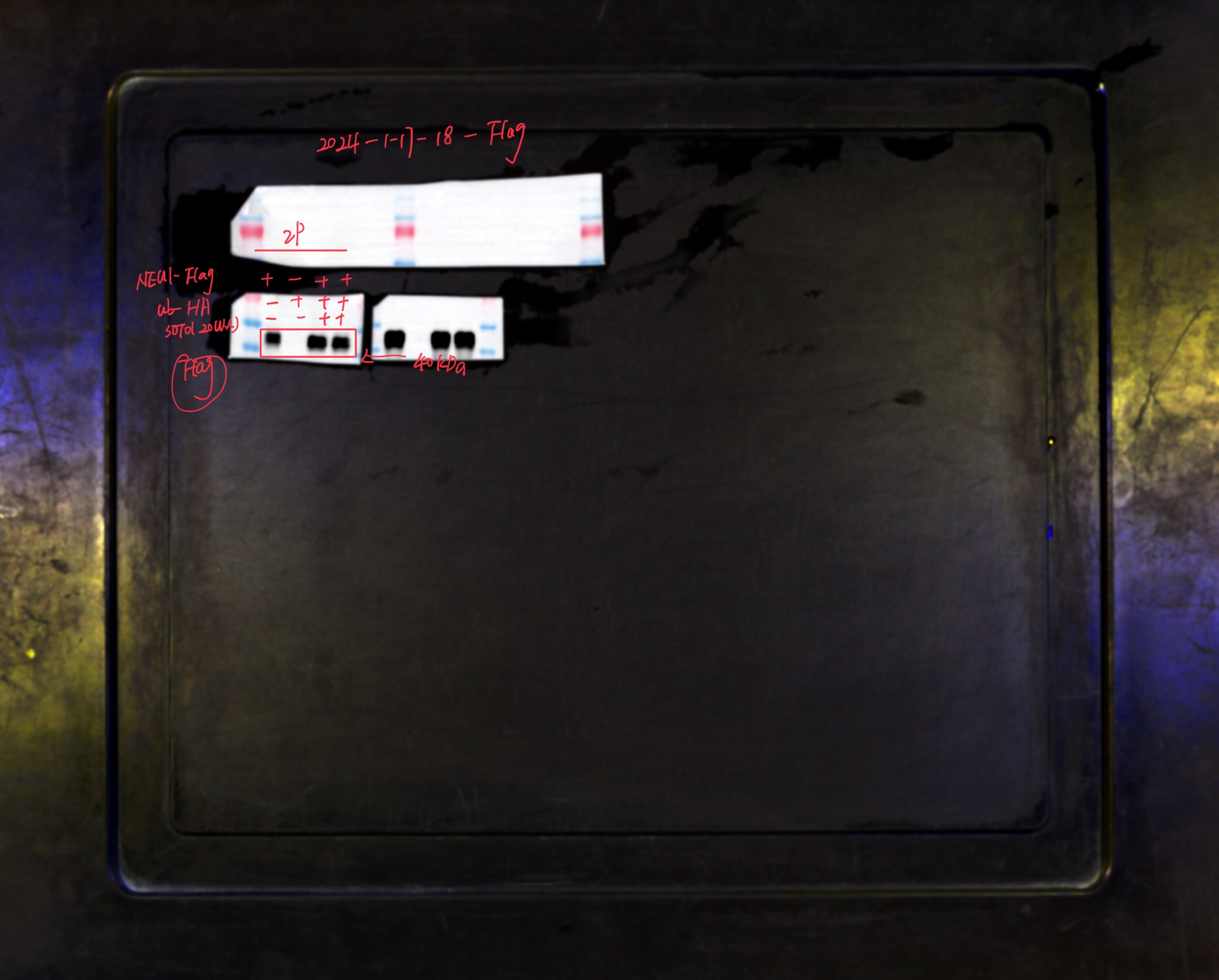


Fig. 5-E


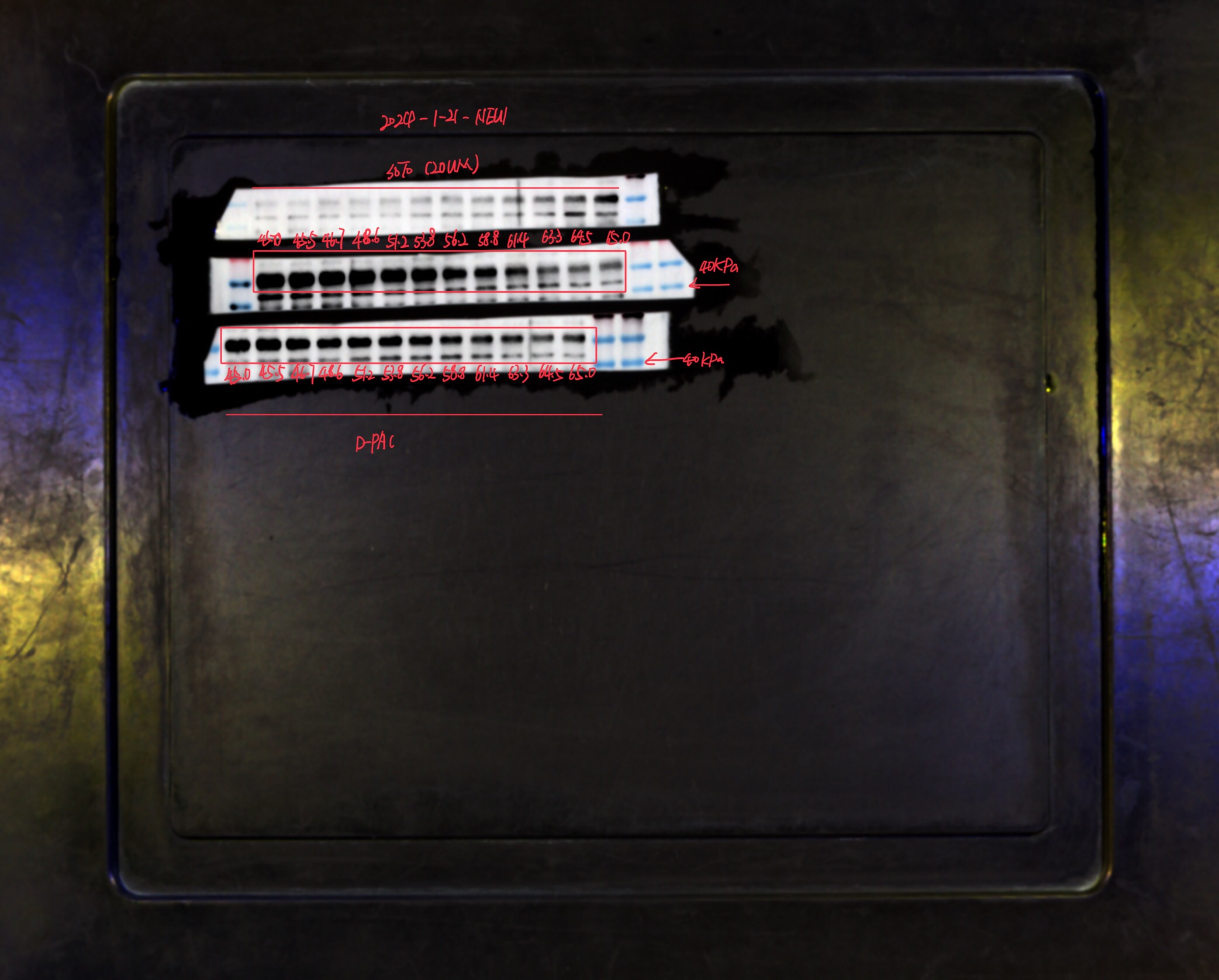


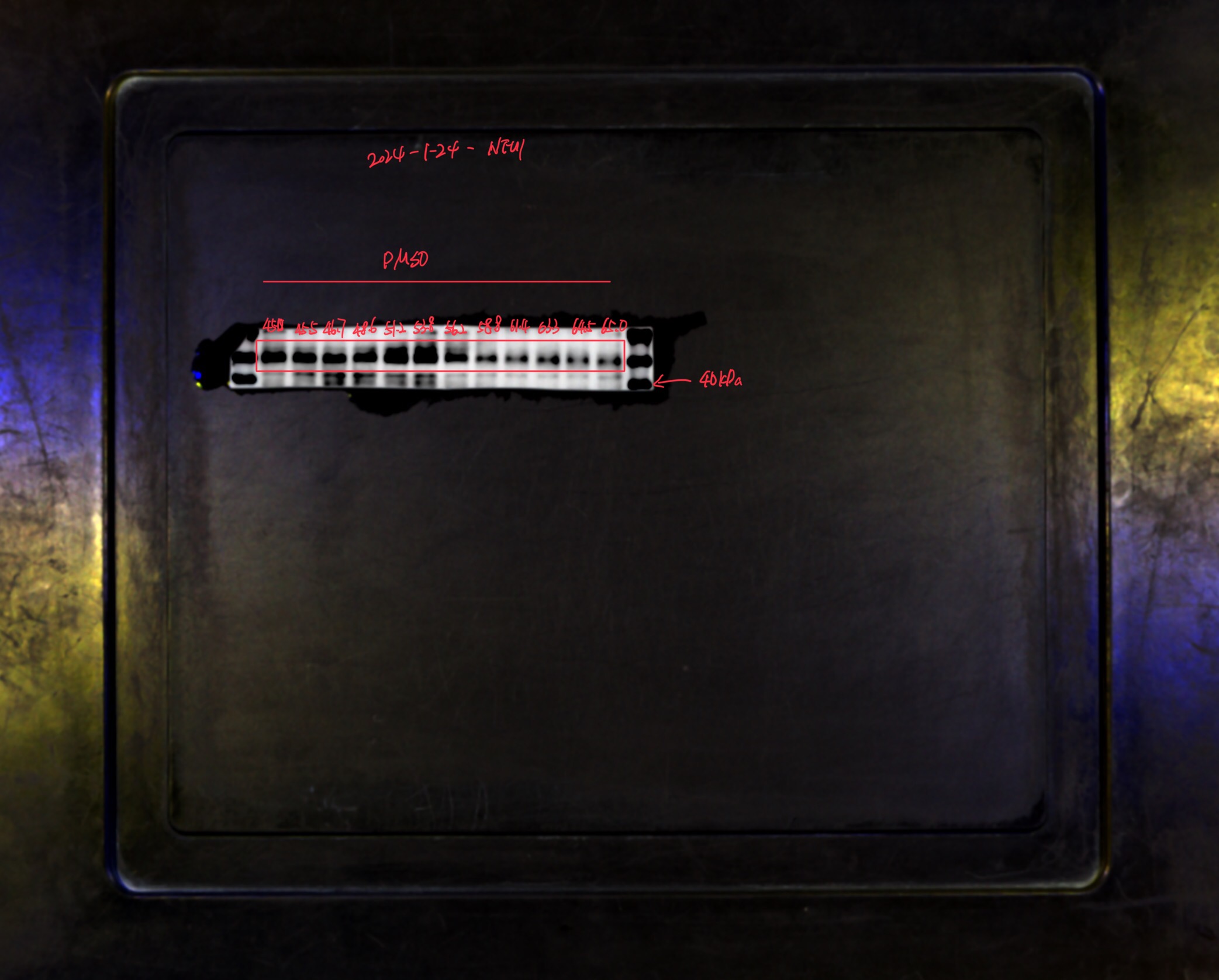


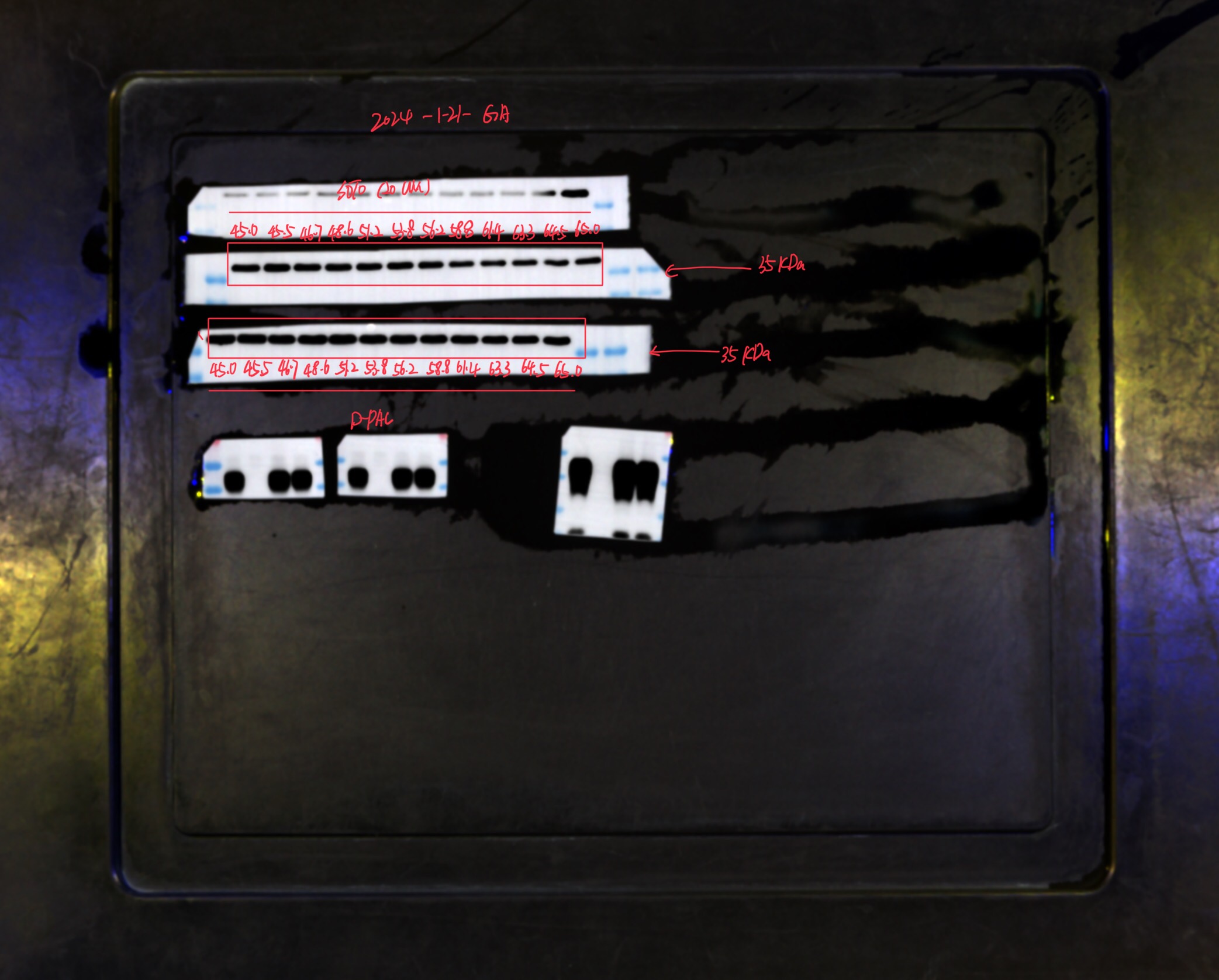


Fig. 6-B


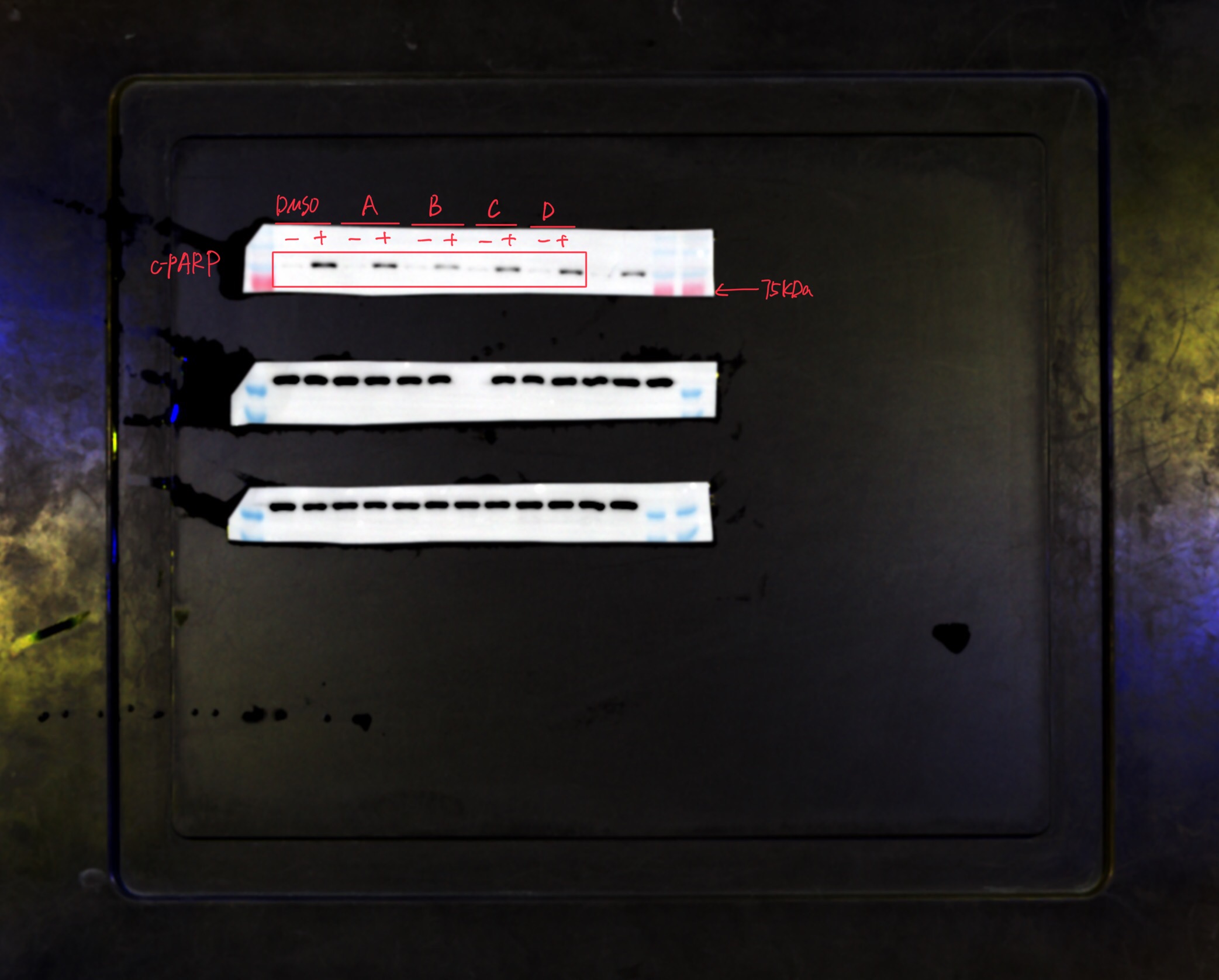

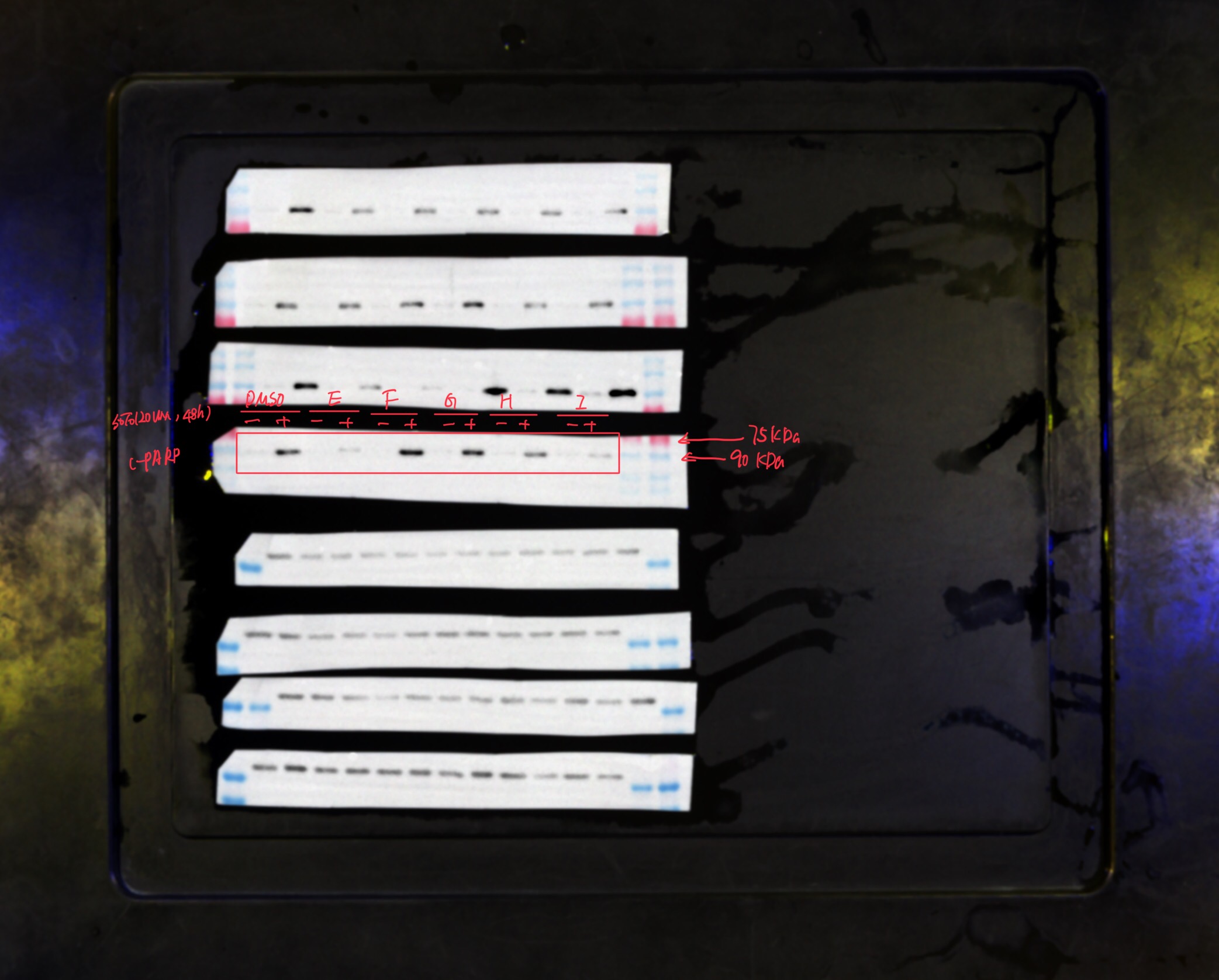


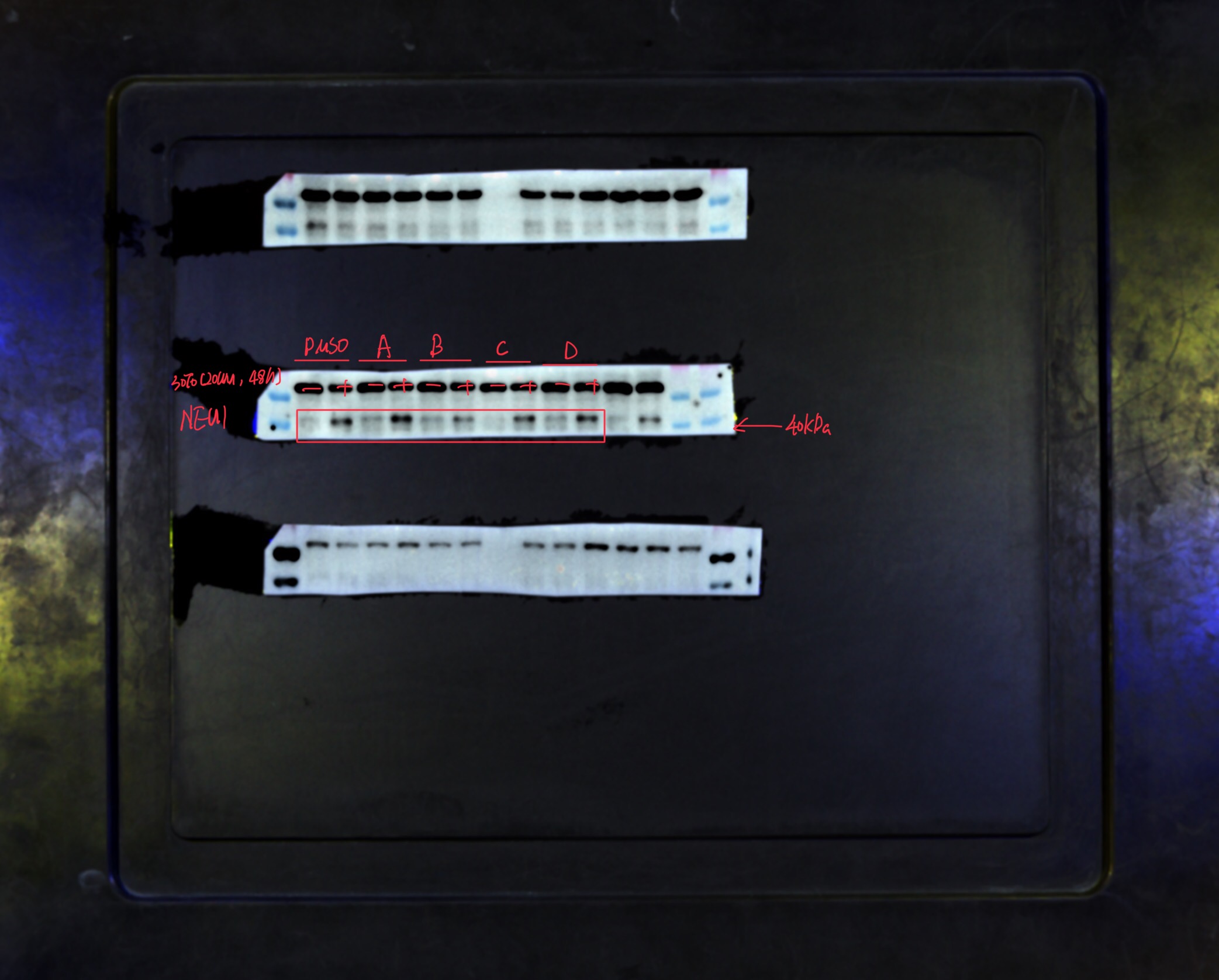

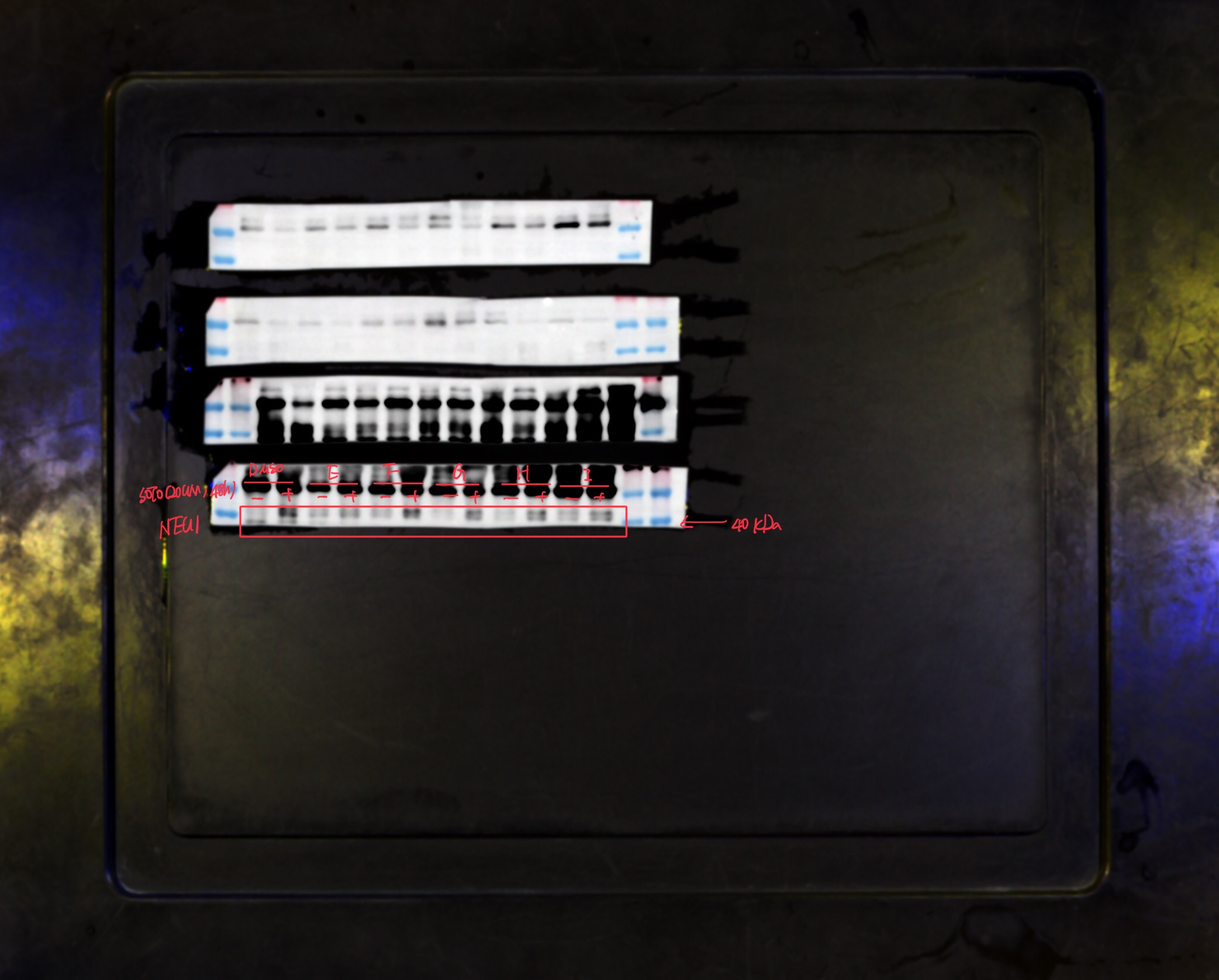


Fig. 6-C-D

Fig. 6-I

Fig 7-E

Fig S5 E
