# Supplementary material for: Sotorasib-impaired degradation of NEU1 contributes to cardiac injury by inhibiting AKT signaling
Source: Cell Death Discov. 2025 Apr 12;11:169. doi: 10.1038/s41420-025-02431-x (PMC11993734; doi:10.1038/s41420-025-02431-x)
Supplement: Supplementary file 2 — supplementary materials [file 41420_2025_2431_MOESM2_ESM.docx]

**Sotorasib-impaired degradation of NEU1 contributes to cardiac injury by inhibiting AKT signaling**

Mengting Cheng^1^, Wentong Wu^2^, Qing Li^1^, Xinyu Tao^3^, Feng Jiang^2^, Jinjin Li^2^, Nonger Shen^1^, Fei Wang^4^, Peihua Luo^2, 5, 6^, Qiaojun He^2, 7^, Ping huang^1, 8^, Zhifei Xu^2*^, Yiwen Zhang^1, 8, 9*^

^1^ Clinical Pharmacy Center, Department of Pharmacy, Zhejiang Provincial People's Hospital, Affiliated People's Hospital, Hangzhou Medical College, Hangzhou 310014, Zhejiang, China

^2^ Center for Drug Safety Evaluation and Research of Zhejiang University, College of Pharmaceutical Sciences, Zhejiang University, Hangzhou 310058, China

^3^ School of Pharmaceutical Sciences, Zhejiang Chinese Medical University, Hangzhou, 310053, Zhejiang, China

^4^ Outpatient Pharmacy, Department of Pharmacy, Zhejiang Provincial People's Hospital, Affiliated People's Hospital, Hangzhou Medical College, Hangzhou 310014, Zhejiang, China

^5^ Department of Pharmacology and Toxicology, Hangzhou Institute of Innovative Medicine, College of Pharmaceutical Sciences, Zhejiang University, Hangzhou 310018, China

^6^ Key Laboratory of Clinical Cancer Pharmacology and Toxicology Research of Zhejiang Province, Affiliated Hangzhou Cancer Hospital, Zhejiang University School of Medicine, Hangzhou 310002, China

^7^ Innovation Institute for Artificial Intelligence in Medicine of Zhejiang University, Hangzhou 310018, China

^8^ Zhejiang Provincial Clinical Research Center for malignant tumor, 310014, Hangzhou, Zhejiang, People's Republic of China

^9^ Key Laboratory of Endocrine Gland Diseases of Zhejiang Province, Hangzhou, China

* Correspondence to:

Clinical Pharmacy Center, Department of Pharmacy, Zhejiang Provincial People's Hospital, Affiliated People's Hospital of Hangzhou Medical College, 158 Shangtang Road, Gongshu District, Hangzhou 310014, China.

Center for Drug Safety Evaluation and Research of Zhejiang University, College of Pharmaceutical Sciences, Zhejiang University, Hangzhou 310058, China

Email: [zhangyiwen@hmc.edu.cn](mailto:zhangyiwen@hmc.edu.cn); xzfzjut@zju.edu.cn.


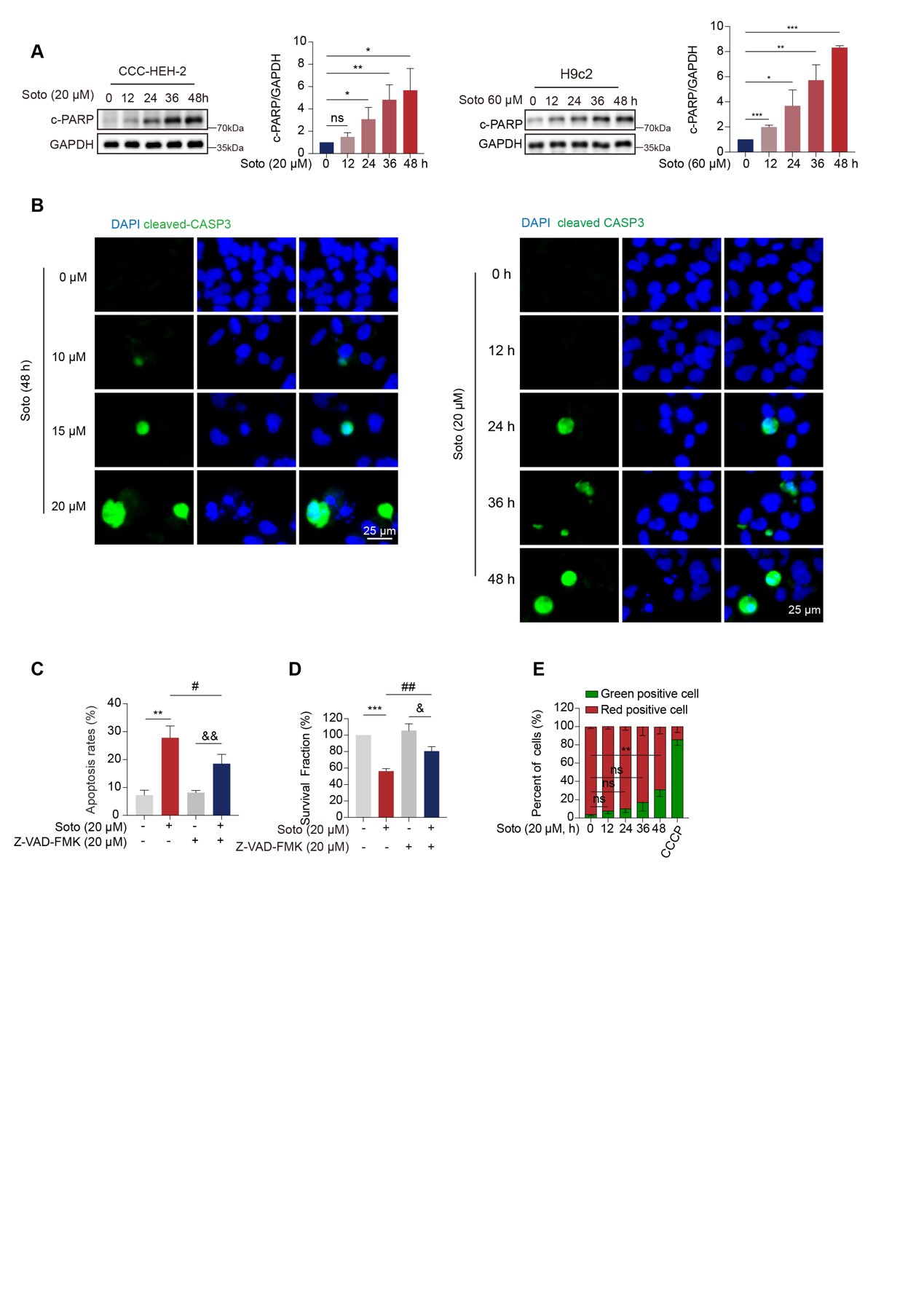


**Fig. S1 Sotorasib induces apoptosis and mitochondrial dysfunction in cardiomyocytes.**

(A) 20 μM or 60 μM sotorasib treatment of CCC-HEH-2 or H9c2 cells for 12, 24, 36 and 48 h. Expression levels of c-PARP protein in CCC-HEH-2 and H9c2 cells (n = 3). (B) CCC-HEH-2 or H9c2 cells were treated with 20 μM or 60 μM sotorasib for 0, 12, 24, 36, and 48 h and exposed to different concentrations of 0, 10, 15, or 20 μM sotorasib for 48 h. Representative image of cleaved-CASP3 IF (n = 3). Scale bars: 25 μm. 20 μM sotorasib and/or 20 μM Z-VAD-FMK treated CCCHEH-2 for 48 hours. (C) The apoptosis rate of CCC-HEH-2 cells (n = 3). (D) CCC-HEH-2 cell survival rate. (E) 20 μM sotorasib treatment of CCC-HEH-2 cells for 0, 12, 24, 36 and 48 h. MMP changes in CCC-HEH-2 cells (n = 3). Data expressed as mean ± SD, ***, *p*＜0.001, **, *p*＜0.01, *, *p*＜0.05 (vs. Vehicle), ##, *p*＜0.01 #, *p*＜0.05(*vs*. sotorasib), &&, *p*＜0.01, &, *p*＜0.05 (*vs*. Z-VAD-FMK), ns, no significance.


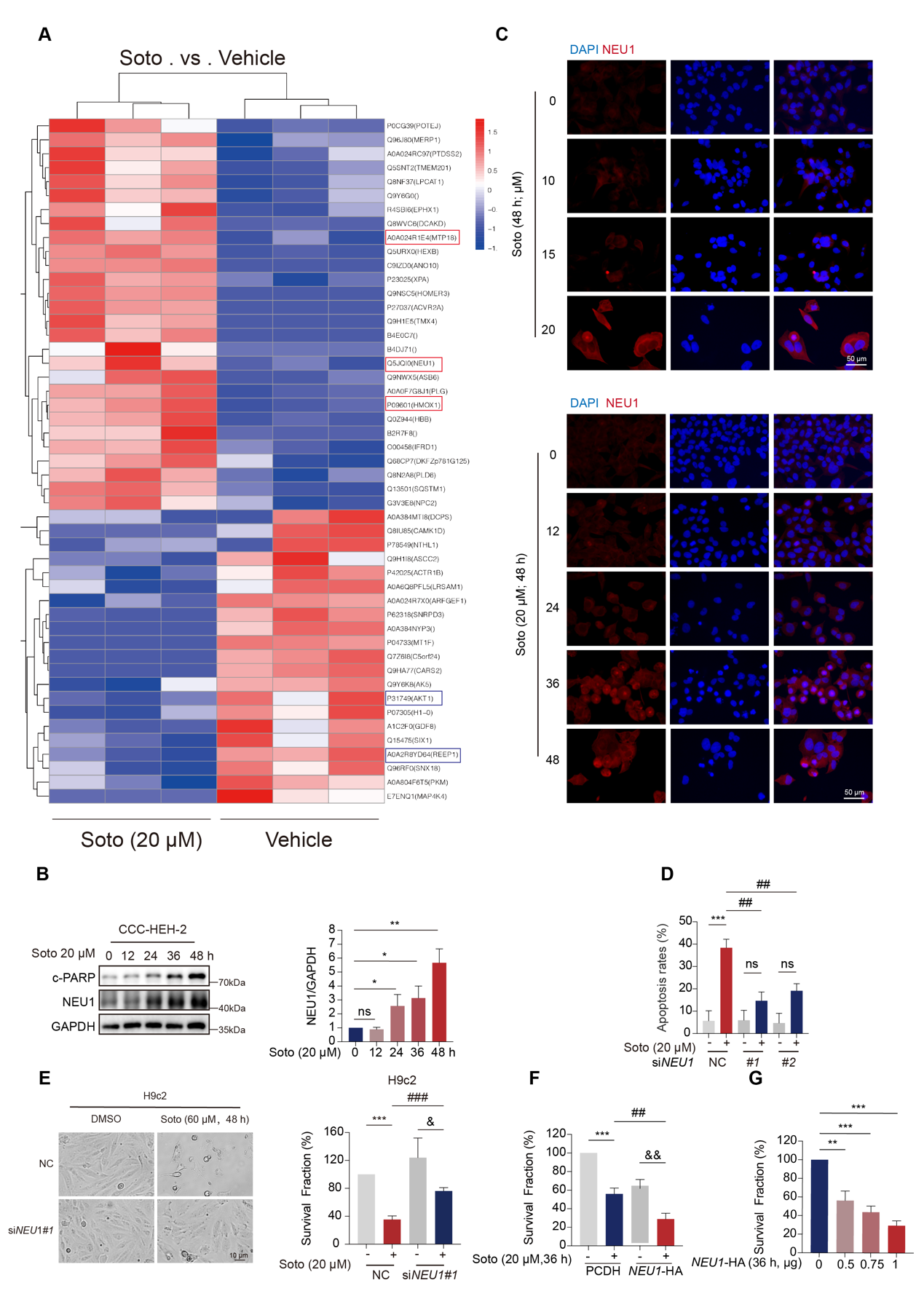


**Fig. S2 Sotorasib mediated upregulation of NEU1 contributes to myocardial cell apoptosis.**

(A) Proteomic analysis of CCC-HEH-2 cell samples from control and sotorasib groups (n = 3). Heatmap showing differentially expressed proteins. (B) 20 μM sotorasib treatment of CCC-HEH-2 cells for 0, 12, 24, 36 and 48 h. Expression levels of c-PARP protein in CCC-HEH-2 cells (n = 3). (C) CCC-HEH-2 cells were treated with 20 μM sotorasib for 0, 12, 24, 36, and 48 h and exposed to different concentrations of 0, 10, 15, or 20 μM sotorasib for 48 h. Representative image of NEU1 IF (n = 3). Scale bars: 25 μm. (D) CCC-HEH-2 cells transfected with NC, *NEU1*#1 or *NEU1*#2 knockdown were treated with sotorasib for 48 h. CCC-HEH-2 cell apoptosis rate (n = 3). (E) H9c2 cells transfected with NC or *NEU1*#1 knockdown were treated with sotorasib for 48 h. Survival fraction and representative pictures of H9c2 cells (n=3). (F) CCC-HEH-2 cells were transfected with 0.5 μg PCDH or *NEU1*-HA plasmid after sotorasib treatment for 36 h. Survival fraction and representative pictures of CCC-HEH-2 cells (n=3). (G) CCC-HEH-2 cells were transfected with *NEU1*-HA plasmid overexpressing NEU1 at concentrations of 0, 0.5, 0.75 and 1 μg, respectively, for 36 h. Survival fraction and representative pictures of CCC-HEH-2 cells (n=3). Data expressed as mean ± SD, ***, *p*＜0.0001, **, *p*＜0.01, *, *p*＜0.05 (vs. Vehicle), ###, *p*＜0.001 ##, *p*＜0.01 (*vs*. sotorasib), &&, *p*＜0.01, &, *p*＜0.05 (*vs*.si*NEU1*#1 or *NEU1*-HA) ns, no significance.


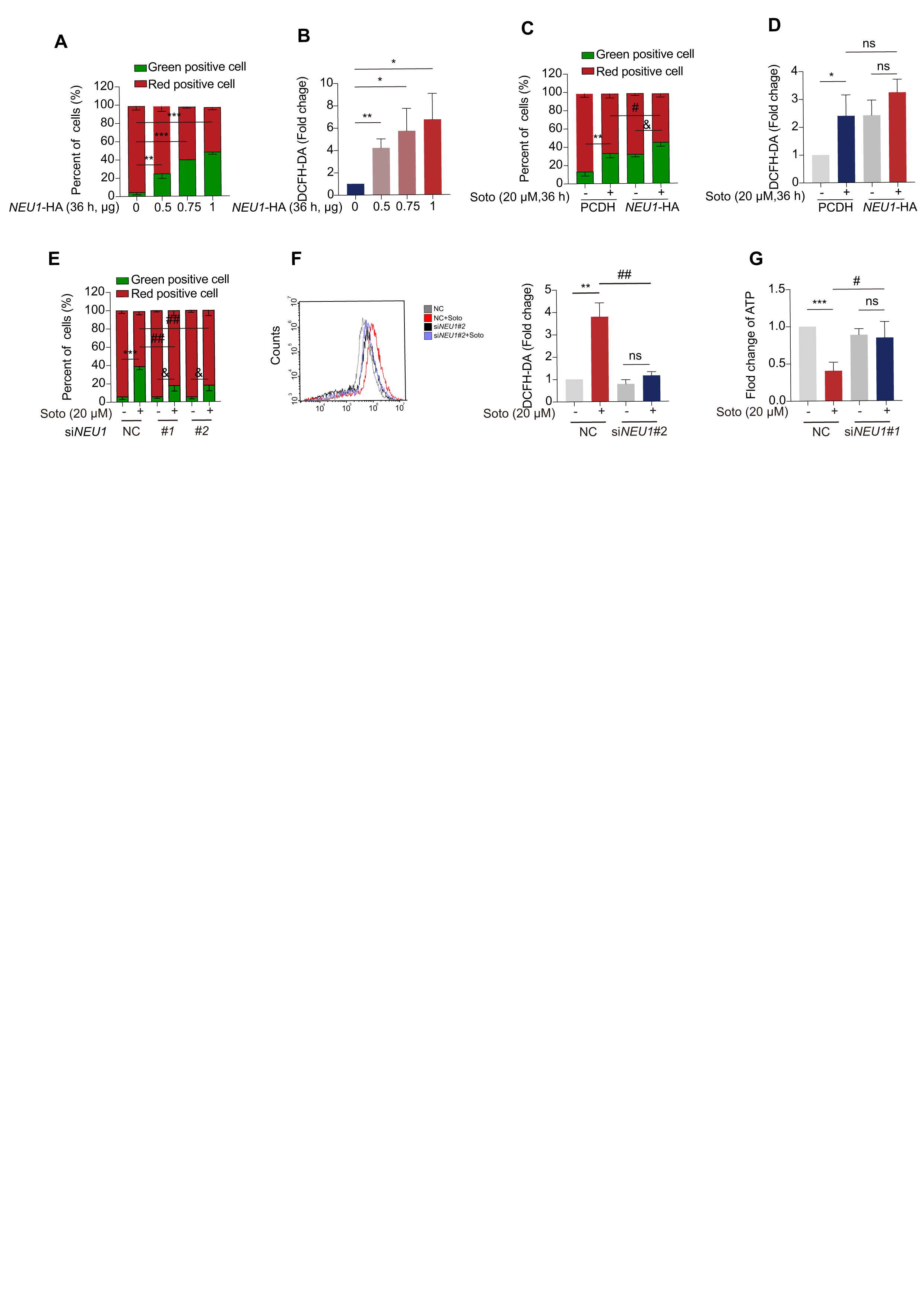


**Fig. S3 Sotorasib induced high expression of NEU1 promotes mitochondrial dysfunction.**

(A) CCC-HEH-2 cells transfected with NC, *NEU1*#1 or *NEU1*#2 knockdown were treated with sotorasib for 48 h. MMP changes in CCC-HEH-2 cells (n = 3). CCC-HEH-2 cells transfected with NC and *NEU1*#1 knockdown were treated with sotorasib for 48 h. (B) ROS levels in CCCHEH-2 cells (n = 3). (C) ATP levels in CCC-HEH-2 cells (n = 3). CCC-HEH-2 cells were transfected with *NEU1*-HA plasmid overexpressing NEU1 at concentrations of 0, 0.5, 0.75 and 1 μg, respectively, for 48 h. (D) MMP changes in CCC-HEH-2 cells (n = 3). (E) ROS levels in CCC-HEH-2 cells (n = 3). CCC-HEH-2 cells were transfected with *NEU1*-HA plasmid overexpressing NEU1 at concentrations of 0, 0.5, and 0.75 μg, respectively, for 36 h. (F) MMP changes in CCC-HEH-2 cells (n = 3). (G) ROS levels in CCC-HEH-2 cells (n = 3). Data expressed as mean ± SD, ***, *p*＜0.0001, **, *p*＜0.01, *, *p*＜0.05 (vs. Vehicle), , ##, *p*＜0.01 #, *p*＜0.05 (*vs*. NC + sotorasib), ns, no significance.


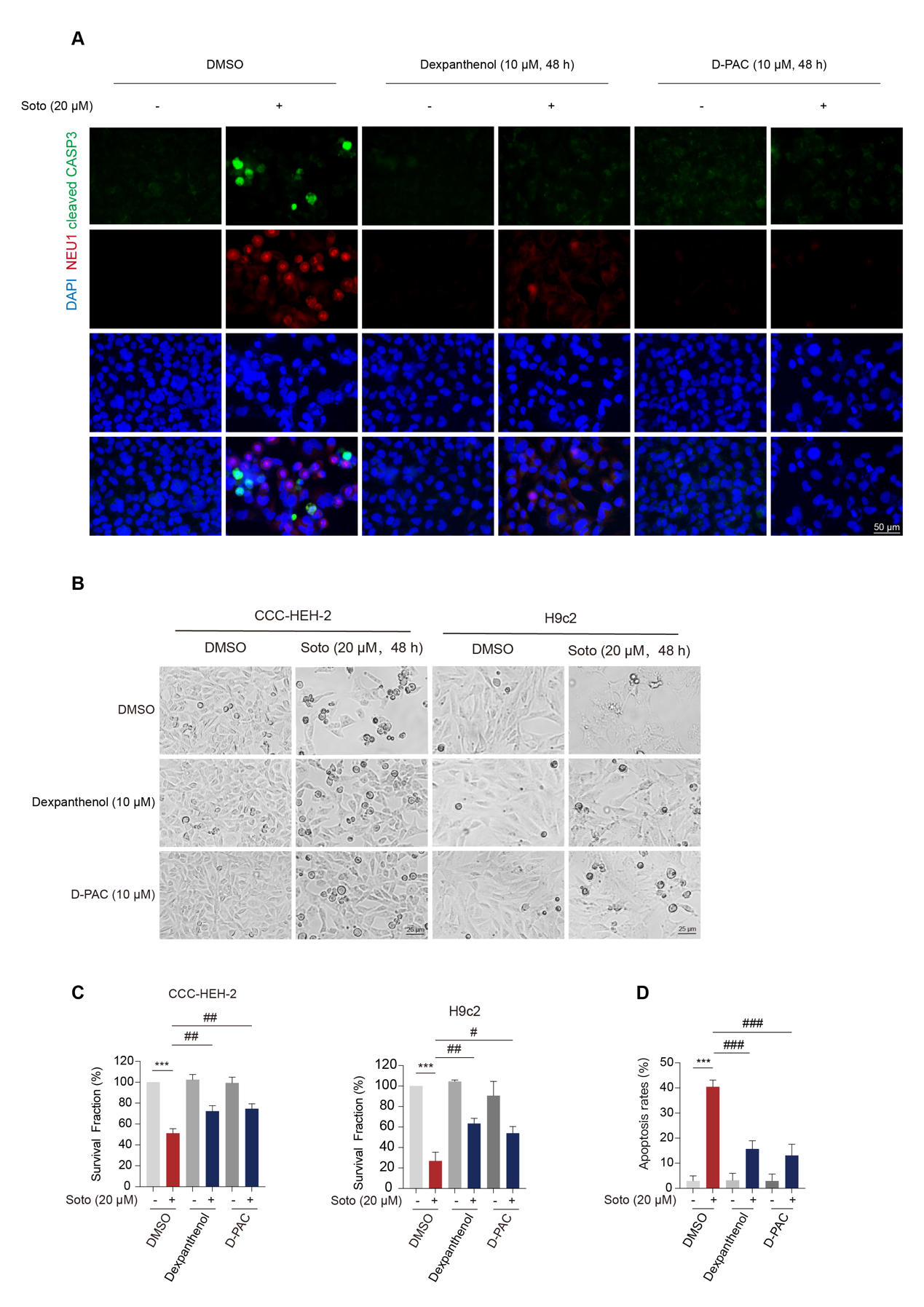


**Fig. S4 D-PAC decreases NEU1 protein expression levels to rescue the cardiotoxicity induced by sotorasib.**

CCC-HEH-2 cells were treated with D-PAC (10 μM) or dexpanthenol (10 μM) in combination with sotorasib 20 μM for 48 h. (A) Representative image of cleaved-CASP3 IF (n = 3). Scale bars: 50 μm. CCC-HEH-2 or H9c2 cells were treated with D-PAC(10 μM) or dexpanthenol (10 μM) in combination with sotorasib 20 μM or 60 μM for 48 h. (B) Representative pictures of CCC-HEH-2 and H9c2 cells (n=3). Scale bar: 25 μm. (C) CCC-HEH-2 and H9c2 cells survival fraction (n=3). (D) CCC-HEH-2 cells were treated with D-PAC (10 μM) or dexpanthenol (10 μM) in combination with sotorasib 20 μM for 48 h. CCC-HEH-2 cells apoptosis rates (n = 3). Data expressed as mean ± SD, ***, *p*＜0.0001 (vs. Vehicle), ###, *p*＜0.0001, ##, *p*＜0.01 #, *p*＜0.05 (*vs*. sotorasib), ns, no significance.


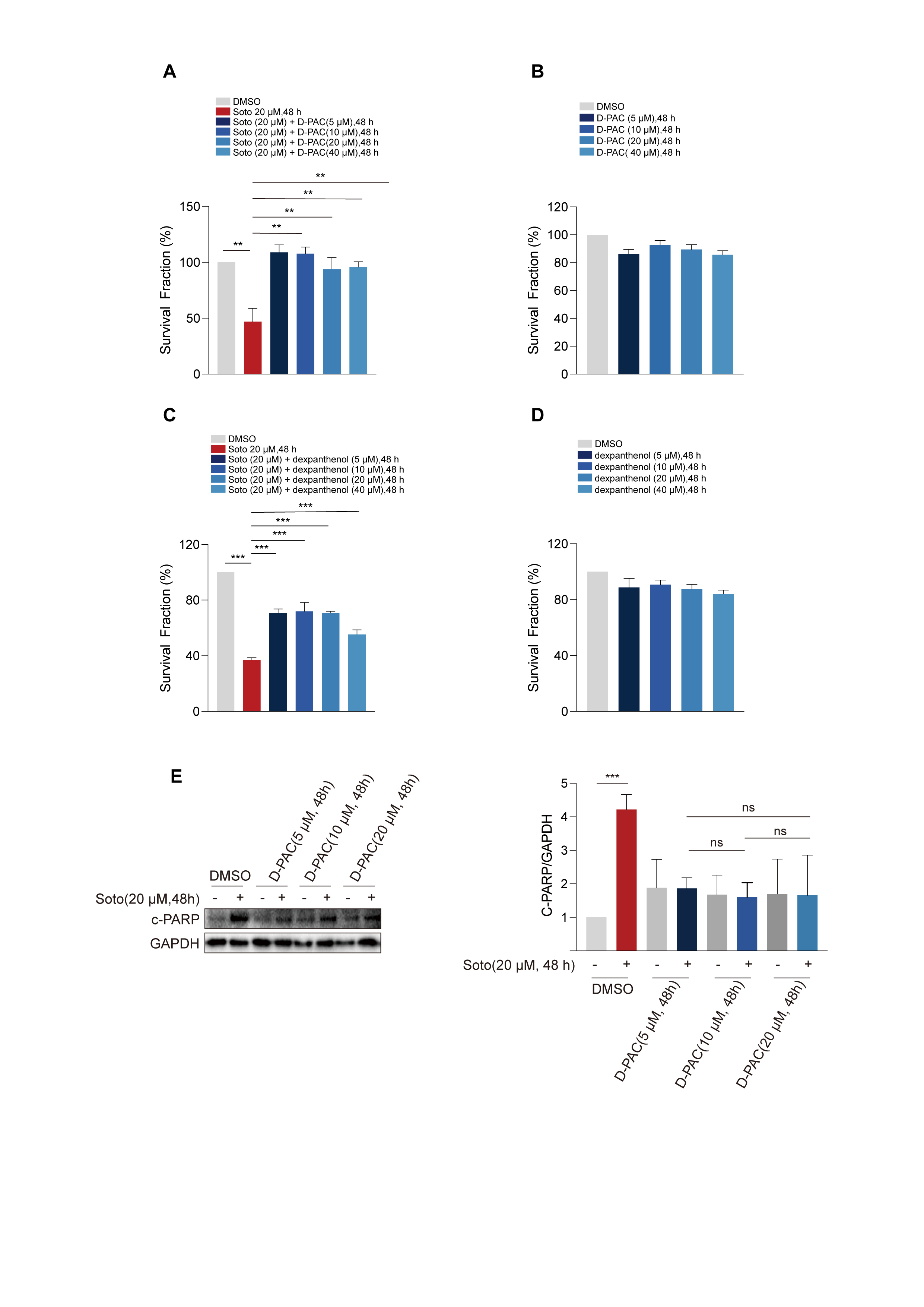


**Fig. S5 D-PAC and dexpanthenol rescues sotorasib-induced cardiomyocyte apoptosis in a concentration-independent manner.**

(A) CCC-HEH-2 cells were treated with D-PAC (5, 10, 20 or 40 μM) in combination with sotorasib 20 μM for 48 h. Survival fraction detection by SRB colorimetric assay (n = 3) (B) CCC-HEH-2 cells were exposed to different concentrations of 0, 5, 10, 20 or 40 μM D-PAC for 48 h. Survival fraction detection by SRB colorimetric assay (n = 3). (C) CCC-HEH-2 cells were treated with dexpanthenol (5, 10, 20 or 40 μM) in combination with sotorasib 20 μM for 48 h. Survival fraction detection by SRB colorimetric assay (n = 3). (D) CCC-HEH-2 cells were exposed to different concentrations of 0, 5, 10, 20 or 40 μM dexpanthenol for 48 h. Survival fraction detection by SRB colorimetric assay (n = 3). (E) CCC-HEH-2 cells were treated with D-PAC (5, 10, or 20 μM) in combination with sotorasib 20 μM for 48 h. Expression levels of c-PARP protein in CCC-HEH-2 cells (n = 3).Data expressed as mean ± SD, **, *p*＜0.01***, *p*＜0.0001 (vs. Vehicle).


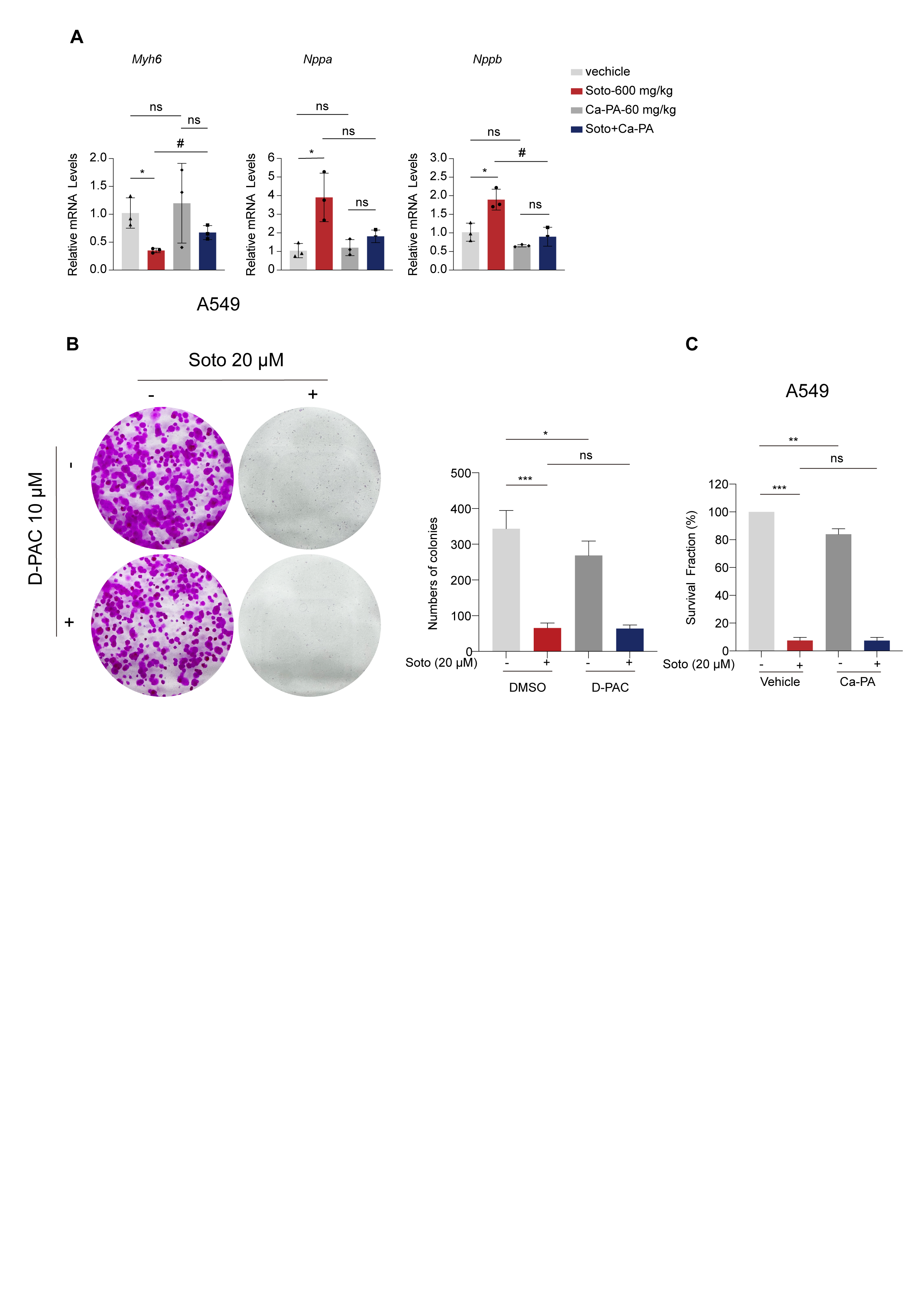


**Fig. S6 Ca-PA ameliorated sotorasib-induced cardiotoxicity without affecting its antitumor activity.**

C57BL/6J male mice were treated with 600 mg/kg/d sotorasib and/or 60 mg/kg/d Ca-PA for 28 days. (A) Myocardial remodeling indicator mRNA expression levels (n = 3). (B) 549 cells were treated with sotorasib 20 μM and/or D-PAC 10 μM for 48h and detection of cell clone formation (n = 3). (C)A549 cells were treated with sotorasib 20 μM and/or D-PAC 10 μM for 48h and the viability of A549 cells was examined (n = 3). Data expressed as mean ± SD, **, p＜0.01, *, p＜0.05 (vs. Vehicle), #, p＜0.05 (vs. sotorasib), ns, no significance.

**Table S1. Enriched proteins in sotorasib treated CCC-HEH-2 cells.**

| Protein | Description | Gene | Fold change |
| --- | --- | --- | --- |
| A0A804F6T5 | Pyruvate kinase | PKM | 0.47 |
| E7ENQ1 | Mitogen-activated protein kinase kinase kinase kinase 4 | MAP4K4 | infinitesimal |
| Q13501 | Sequestosome-1 | SQSTM1 | 2.16 |
| A1C2F0 | Growth/differentiation factor 8 | GDF8 | 0.47 |
| P42025 | Beta-centractin | ACTR1B | 0.46 |
| R4SBI6 | Epoxide hydrolase | EPHX1 | 2.18 |
| A0A384MTI8 | m7GpppX diphosphatase | DCPS | 0.36 |
| P31749 | RAC-alpha serine/threonine-protein kinase | AKT1 | 0.43 |
| P0CG39 | POTE ankyrin domain family member J | POTEJ | 2.40 |
| Q5URX0 | beta-N-acetylhexosaminidase | HEXB | 2.67 |
| A0A024R7X0 | ADP-ribosylation factor guanine nucleotide-exchange factor 1(Brefeldin A-inhibited), isoform CRA_a | ARFGEF1 | 0.30 |
| P27037 | Activin receptor type-2A | ACVR2A | Inf |
| Q15475 | Homeobox protein SIX1 | SIX1 | 0.41 |
| P07305 | Histone H1.0 | H1-0 | 0.32 |
| Q5SNT2 | Transmembrane protein 201 | TMEM201 | 2.53 |
| Q9NWX5 | Ankyrin repeat and SOCS box protein 6 | ASB6 | 2.28 |
| O00458 | Interferon-related developmental regulator 1 | IFRD1 | 2.86 |
| Q96RF0 | Sorting nexin-18 | SNX18 | 0.48 |
| Q8WVC6 | Dephospho-CoA kinase domain-containing protein | DCAKD | 2.44 |
| A0A024R1E4 | Mitochondrial fission process protein 1 | MTP18 | 2.06 |
| B4DJ71 | Phosphate transporter | Inf |  |
| Q5JQI0 | exo-alpha-sialidase | NEU1 | 2.19 |
| Q9Y6K8 | Adenylate kinase isoenzyme 5 | AK5 | 0.50 |
| Q0Z944 | Beta globin (Fragment) | HBB | 5.43 |
| Q9Y6G0 | RNA-binding protein | 2.58 |  |
| Q9H1I8 | Activating signal cointegrator 1 complex subunit 2 | ASCC2 | 0.43 |
| Q96J80 | Mammalian ependymin-related protein 1 | MERP1 | 2.18 |
| A0A024RC97 | Phosphatidylserine synthase | PTDSS2 | 2.16 |
| Q8IU85 | Calcium/calmodulin-dependent protein kinase type 1D | CAMK1D | infinitesimal |
| P04733 | Metallothionein-1F | MT1F | infinitesimal |
| Q9HA77 | Probable cysteine--tRNA ligase, mitochondrial | CARS2 | infinitesimal |
| P78549 | Endonuclease III-like protein 1 | NTHL1 | 0.23 |
| Q9H1E5 | Thioredoxin-related transmembrane protein 4 | TMX4 | Inf |
| Q8N2A8 | Mitochondrial cardiolipin hydrolase | PLD6 | 2.01 |
| Q68CP7 | ABC-type glutathione-S-conjugate transporter | DKFZp781G125 | 2.53 |

**Continued Table S1.**

| Protein | Description | Gene | Fold change |
| --- | --- | --- | --- |
| C9IZD0 | Anoctamin-10 (Fragment) | ANO10 | Inf |
| B4E0C7 | Tripeptidyl-peptidase 1 |  | Inf |
| A0A384NYP3 | Epididymis secretory sperm binding protein |  | infinitesimal |
| Q7Z6I8 | UPF0461 protein C5orf24 | C5orf24 | Inf |
| Protein | Description | Gene | Fold change |
| A0A804F6T5 | Pyruvate kinase | PKM | 0.47 |
| E7ENQ1 | Mitogen-activated protein kinase kinase kinase kinase 4 | MAP4K4 | infinitesimal |
| Q13501 | Sequestosome-1 | SQSTM1 | 2.16 |
| A1C2F0 | Growth/differentiation factor 8 | GDF8 | 0.47 |
| P42025 | Beta-centractin | ACTR1B | 0.46 |
| R4SBI6 | Epoxide hydrolase | EPHX1 | 2.18 |
| A0A384MTI8 | m7GpppX diphosphatase | DCPS | 0.36 |
| P31749 | RAC-alpha serine/threonine-protein kinase | AKT1 | 0.43 |
| P0CG39 | POTE ankyrin domain family member J | POTEJ | 2.40 |
| Q5URX0 | beta-N-acetylhexosaminidase | HEXB | 2.67 |

**Table S2.**

| Antibodies | |
| --- | --- |
| GAPDH | db106, 1:10000, Diagbio, Hangzhou, Zhejiang, China |
| anti-c-PARP | ET1608-10, Huabio, Hangzhou, Zhejiang, China |
| anti-NEU1 | sc-166824, 1:500, Santa Cruz Biotechnology |
| Phospho-Akt1 (Ser473) | D7F10,1:1000, Cell Signaling Technology, Boston, MA, USA |
| Anti-AKT1 | 2938S,1:1000, Cell Signaling Technology, Boston, MA, USA |
| Phospho-AMPKα (Thr172) | 2535S,1:1000, Cell Signaling Technology, Boston, MA, USA |
| Cleaved caspase 3 | #9664, 1;1000, Cell Signaling Technology, Boston, MA, USA |
| anti-FLAG tag | (db7002, 1:1000, Diagbio, Hangzhou, Zhejiang, China) |
| anti-HA tag | db2603, 1:1000, Diagbio, Hangzhou, Zhejiang, China |
| Anti-PGC-1α | sc-518025,1:500, Santa Cruz Biotechnology |

**Table S3.**

| Quantitative real-time PCR (qPCR) | |
| --- | --- |
| Human-*GAPDH* forward | 5'-GGAGCGAGATCCCTCCAAAAT-3ʹ |
| Human-*GAPDH* reverse | 5'-GGCTGTTGTCATACTTCTCATGG-3ʹ |
| Human-*Neu1* forward | 5'-TTCTCCAACCCAGCACATC-3ʹ |
| Human-*Neu1* reverse | 5'-ACTGTCTCTTTCCGCCATG-3ʹ |
| Human -*Neu1* forward | 5'-GCCAAGTTCATCGCCATG-3ʹ |
| Human -*Neu1* reverse  Mouse-*GAPDH* forward | 5'-CCCTGTGTCTACATCGTTCAC-3ʹ  5'-TCAACAGCAACTCCCACTCTTCC-3ʹ |
| Mouse-*GAPDH* reverse | 5'-ACCCTGTTGCTGTAGCCGTATTCA-3ʹ |
| Mouse-*Nppa* reverse | 5ʹ- GTCTAGCAGGTTCTTGAAATC-3ʹ |
| Mouse-*Nppb* forward | 5ʹ-AAGATGGTGCAAGGGTCTG-3ʹ |
| Mouse-*Myh6* forward | 5ʹ- TTGGGAAATTCATCCGAATC-3ʹ |
| Mouse-*Myh7* forward | 5ʹ- AATCCTAATGCAAACAAGGG 3ʹ |
| Mouse-*Myh7* forward | 5ʹ- CAGAAGGTAGGTCTCTATGTC 3ʹ |

Italics indicate primer names

**Table S4.**

| siRNA oligonucleotides | |
| --- | --- |
| *NEU1* siRNA #1 | 5’-GCTTCAGCAATGGTACCTCAT-3’ |
| *NEU1* siRNA #2 | 5’-CCCGGAATCTCTCCCTGGATA-3’ |

**Continued Table S4.**

| siRNA oligonucleotides | |
| --- | --- |
| *REEP1* siRNA #1 | 5’-GACATCTTCCTTTGTTGGTTT-3’ |
| *HMOX1* siRNA #1 | 5’-ACAGTTGCTGTAGGGCTTTAT-3’ |
| *MTP18* siRNA #1 | 5’-GCCATTGACAAAGGCAAGAAG-3’ |
| *AKT1* siRNA #1 | 5’-GGACAAGGACGGGCACATTAA-3’ |

Italics indicate siRNA names
